# Comparison of EWMA, MA, and MQ Under a Unified PBRTQC Framework for Thyroid and Coagulation Tests

**DOI:** 10.3390/diagnostics16020288

**Published:** 2026-01-16

**Authors:** Banjiu Zhaxi, Chaochao Ma, Qian Chen, Yingying Hu, Wenyi Ding, Xiaoqi Li, Ling Qiu

**Affiliations:** 1 Department of Laboratory Medicine, Peking Union Medical College Hospital, Peking Union Medical College & Chinese Academy of Medical Science, Beijing 100730, China; 2 Department of Occupational and Environmental Health Sciences, School of Public Health, Peking University, Beijing 100191, China; 3 State Key Laboratory of Complex Severe and Rare Diseases, Peking Union Medical College Hospital, Peking Union Medical College & Chinese Academy of Medical Science, Beijing 100730, China

**Keywords:** patient-based real-time quality control, moving average, moving quantile, exponentially weighted moving average, transform–truncate–alarm framework

## Abstract

**Background:** Patient-based real-time quality control (PBRTQC) enables continuous analytical monitoring using routine patient results; however, the performance of classical statistical process control (SPC) algorithms varies across analytes, and standardized evaluation and optimization strategies remain limited. To address this gap, this study compared three SPC algorithms—moving average (MA), moving quantile (MQ), and exponentially weighted moving average (EWMA)—within a unified preprocessing framework and proposed a composite performance metric for parameter optimization. **Methods:** Routine patient results from six laboratory analytes were analyzed using a standardized “transform–truncate–alarm” PBRTQC workflow. Simulated systematic biases were introduced for model training, and algorithm-specific parameters were optimized using a composite metric integrating sensitivity, false-positive rate (FPR), and detection delay. Performance was subsequently evaluated on an independent validation dataset. **Results:** For most analytes, all three SPC algorithms demonstrated robust PBRTQC performance, achieving high sensitivity (generally ≥0.85), very low false-positive rates (<0.002), and rapid detection of systematic bias. EWMA showed more balanced performance for thyroid-stimulating hormone (TSH), with improved sensitivity and shorter detection delay compared with MA and MQ. The proposed composite metric effectively facilitated clinically meaningful parameter optimization across algorithms. **Conclusions:** Under a unified preprocessing framework, classical SPC algorithms provided reliable PBRTQC performance across multiple analytes, with EWMA offering advantages for more variable measurements. The proposed composite metric supports standardized, practical, and analyte-adaptive PBRTQC implementation in clinical laboratories.

## 1. Introduction

Clinical laboratories generate large volumes of test results that directly support diagnosis, treatment decisions, and longitudinal monitoring. Because patient testing is continuous, even modest analytical shifts may influence clinical interpretation when results are compared over time. Routine internal quality control is indispensable, yet it is performed at discrete time points and may not fully capture changes occurring between quality control events. In addition, heterogeneous patient populations and biological variation can complicate the early recognition of subtle analytical drift. With increasing automation and informatics capability, it has become feasible to leverage routinely produced patient results for continuous monitoring of analytical stability. These practical needs motivate approaches that detect systematic bias and analytical drift early while maintaining a low false-alarm burden and minimal additional operational cost.

Patient-based real-time quality control (PBRTQC) is an increasingly recognized approach for continuous analytical monitoring that leverages routine patient test results to identify systematic bias and analytical drift in real time [1,2,3]. Compared with traditional internal quality control, PBRTQC offers several advantages, including higher monitoring frequency, minimal additional cost, and the ability to detect reagent lot shifts [4] or instrument malfunctions earlier under certain circumstances [5]. With the rapid expansion of laboratory information systems and improved computational capacity, PBRTQC has become an important area of interest in laboratory quality management [6] and is gaining increasing attention from professional organizations and guideline committees [7,8]. Consistent with this trend, multiple studies have demonstrated PBRTQC feasibility across analytes and laboratory settings, including detection of lot changes, instrument-related bias, and subtle drift that may be less readily identified by conventional internal quality control [3,4,9,10,11,12]. Over the past decade, recommendations and research efforts have also progressively advanced PBRTQC standardization by outlining implementation prerequisites, informatics considerations, and key performance indicators [1,8,13]. However, many laboratories still lack an integrated and operational workflow that systematically combines data preprocessing, simulated error generation, parameterized statistical control algorithms, and unified strategies for parameter optimization and validation.

Methodologically, PBRTQC research primarily focuses on two categories. Classical statistical process control algorithms—such as moving average (MA), moving quantile (MQ), and exponentially weighted moving average (EWMA)—provide transparent structures and low implementation burden. Machine learning models, including random forests, gradient boosting, and neural networks, have also been introduced to capture more complex patterns of analytical error and subtle drift [14,15,16,17]. Despite their simplicity and interpretability, classical algorithms can be sensitive to skewed distributions, extreme values, heteroscedasticity, and analyte-specific biological characteristics, motivating efforts to improve robustness in real-world PBRTQC applications.

To enhance the stability and applicability of conventional PBRTQC workflows, several refinements have been proposed [18,19,20,21]. Box–Cox transformation can mitigate skewness and improve the robustness of moving-window statistics; the two-parameter Box-Cox variant has been reported to perform well in certain settings [22]. Truncation or Winsorizing procedures can reduce undue influence from outliers [12], and consecutive alarm rules can further lower false-positive rates and improve clinical suitability in high-frequency monitoring scenarios [23]. Together, these components suggest a unified “transform-truncate-alarm” framework that can standardize PBRTQC preprocessing and alarm generation. However, it remains unclear whether MA, MQ, and EWMA perform consistently when embedded within the same unified framework, or whether meaningful algorithm-analyte differences emerge.

PBRTQC performance is typically evaluated using metrics that reflect distinct clinical trade-offs, including sensitivity for bias detection, false-positive rate (FPR), and detection delay (e.g., the median number of patients before error detection, MNPed) [24]. Because these metrics operate on different scales and represent different priorities, selecting optimal parameters based on a single metric—or on unweighted combinations—can be inefficient and inconsistent across analytes. A composite performance indicator that simultaneously incorporates sensitivity, FPR, and detection speed may therefore support more coherent and practical parameter optimization.

Accordingly, the present study systematically evaluates three classical algorithms (MA, MQ, and EWMA) within a consistent PBRTQC workflow under a unified transform-truncate-alarm framework and introduces a composite performance metric that jointly considers sensitivity, false-positive rate, and detection delay to support clinically meaningful parameter optimization. Using real patient data supplemented with simulated systematic error segments, we employ a training-validation design to compare algorithmic behavior across multiple analytes and to assess the practical feasibility of this unified framework in routine clinical laboratory settings.

## 2. Materials and Methods

### 2.1. Study Design and Overview

This was a single-center, retrospective methodological study conducted in the Department of Laboratory Medicine at Peking Union Medical College Hospital (Beijing, China). Routine patient test results for six analytes—thyroid-stimulating hormone (TSH), free triiodothyronine (FT3), free thyroxine (FT4), thrombin time (TT), activated partial thromboplastin time (APTT), and prothrombin time (PT)—were extracted from the laboratory information system for the period from August to October 2025. For each analyte, only the numerical test result, the corresponding measurement time, and the analytical instrument identifier were retrieved; no patient identifiers or clinical information were accessed, and all data were fully de-identified prior to analysis.

For each analyte, the eligible records were ordered chronologically, and the dataset was then split into two non-overlapping subsets: a training set and an independent validation set. The training set was used to establish PBRTQC models and to optimize parameter combinations for three SPC algorithms: MA, MQ, and EWMA. Within the training set, a grid-search strategy [25] was applied to identify the “best” parameter configuration for each algorithm–analyte pair, and the corresponding PBRTQC performance was quantified using predefined metrics.

The validation set was subsequently used to assess the out-of-sample performance and robustness of these optimized PBRTQC models without further parameter tuning. Using the same optimized parameter combinations derived from the training phase, MA-, MQ-, and EWMA-based PBRTQC schemes were applied to the validation data for each analyte. The primary objective of the study was to compare the performance of the three SPC algorithms under a common PBRTQC framework, by evaluating and contrasting their detection capability and false-alarm behavior across TSH, FT3, FT4, TT, APTT, and PT.

### 2.2. Analytical Methods and Instruments

Thyroid function tests and coagulation assays were performed in the Department of Laboratory Medicine at Peking Union Medical College Hospital according to the manufacturer’s instructions and routine laboratory procedures.

TSH, FT3, and FT4 were measured on the Atellica IM 1600 Analyzer (Siemens Medical Solutions, Tarrytown, NY, USA). All assays were performed on serum samples using the manufacturer’s reagent kits and calibration protocols. Only results generated on this single analytical platform during the study period were included in the PBRTQC analysis.

APTT, PT, and TT were measured on the Sysmex CS-5100 coagulation analyzer (Sysmex Corporation, Kobe, Japan). Coagulation testing was performed on sodium citrate-anticoagulated plasma, processed according to standard preanalytical and analytical procedures in the laboratory. Only results obtained from this analyzer were used for subsequent PBRTQC model development and evaluation.

### 2.3. Quality Control

#### 2.3.1. Analytical Quality Assurance

All study data were derived from routine clinical testing performed under the laboratory’s established internal and external quality assurance system. For both thyroid function and coagulation assays, three-level commercial internal quality control materials were analyzed at least once per analytical run according to the manufacturers’ instructions. Internal quality control results were monitored using predefined acceptance criteria and Westgard-type rules, and imprecision and bias were required to remain within allowable limits based on internal specifications and external guidelines.

The laboratory also participated regularly in external quality assessment/proficiency testing schemes for the included analytes. During the study period (August–October 2025), all relevant external quality assessment results met the provider’s performance criteria, and no persistent or clinically significant bias was observed. Only results generated on analytically stable systems with acceptable internal quality control/external quality assessment performance were retained for PBRTQC modelling, ensuring the reliability of the underlying measurement data.

#### 2.3.2. Quality Assurance of Data Processing and Analysis

All data preprocessing, bias simulation, and PBRTQC model construction were implemented using custom R scripts developed by the investigators. The analysis pipeline included explicit checks for missing values, non-numeric entries, and out-of-range timestamps, and used fixed random seeds for simulation steps to ensure reproducibility. The full code base and intermediate outputs (training/validation splits, simulated error datasets, performance summaries) were retained to allow complete traceability of the modelling process. To enhance the robustness of the computational implementation, the core scripts were independently reviewed by at least one additional investigator and cross-checked using two large language model-based code assistants (GPT-5.1 and Gemini 3 Pro). These tools were used to automatically screen for logical inconsistencies, edge-case handling problems, and implementation errors in the statistical procedures. Final decisions on model specification and interpretation were made by the research team, providing an additional layer of quality control for the data analysis workflow.

### 2.4. Data Analysis

All statistical analyses were performed using R software (version 4.5.2) [26]. The following packages and versions were used:

readxl 1.4.5 (data import) [27], dplyr 1.1.4 (data manipulation) [27], openxlsx 4.2.8.1 (Excel export) [28], ggplot2 4.0.0 (visualization) [29], zoo 1.8-14 (moving window calculations) [30], parallel 4.5.2 (parallel computing), and forecast 8.24.0 (Box-Cox transformation) [31].

For each analyte (TSH, FT3, FT4, APTT, PT, and TT), the SPC analysis followed a standardized PBRTQC pipeline. First, raw test results were imported from Excel files, non-numeric symbols were removed, and values were converted to numeric format. A Box-Cox transformation was then applied to approximate normality; when the estimated λ was close to zero, a logarithmic transformation was used instead. If non-positive values were present, a small offset was added to ensure all observations were suitable for transformation. Histograms of the original, transformed, and interquartile range-based outlier-trimmed data were generated solely for visual inspection of distributional shape. For all subsequent PBRTQC modelling and SPC analyses, the full transformed dataset was used.

Firstly, for model development, the most recent 7000 records were then selected in chronological order and split into a training set and a validation set in a 1:1 ratio. The training set was used exclusively for parameter optimization, while the validation set was used for independent performance evaluation.

Within the training set, synthetic systematic errors were introduced to evaluate PBRTQC performance. For each analyte, 10 error scenarios were generated per run, including five positive and five negative bias levels (10%, 30%, 50%, 70%, and 90%). Each scenario contained five error segments of 100–300 consecutive results, separated by 550–600 error-free records to mimic realistic bias patterns. Two indicator variables were added to each dataset to label biased observations (error flag) and segment identity (segment ID), and a summary table of the segment structure was produced.

For each analyte, three SPC algorithms—MA, MQ, and EWMA—were applied under the same unified framework of data transformation, truncation with value replacement, and consecutive alarm rules. The parameter space included window width, quantile level (for MQ), smoothing constant λ (for EWMA), truncation factor, upper and lower control limit multipliers, and the required number of consecutive points beyond the control limits to trigger an alarm. The total number of parameters and the specific search ranges for each parameter are detailed in the Supplementary Materials file. Parallel computing was used to perform a grid search across all parameter combinations on the training error datasets. For each configuration, four performance metrics were calculated: sensitivity (true positive rate for detecting biased segments), false-positive rate (FPR) in bias-free data, the median number of patients before error detection (MNPed), and a composite ME_Score that integrates sensitivity and FPR while penalizing delayed detection using a sigmoid penalty function on MNPed. The weights used in the ME_Score were empirically specified to reflect routine laboratory priorities, with greater emphasis placed on minimizing false-positive alarms, and were not derived through a formal weight-optimization procedure. Sensitivity and false-positive rate were first calculated separately for each simulated bias scenario (bias magnitude and direction) and were then aggregated across scenarios (by averaging) to obtain the overall sensitivity and false-positive rate used in the ME_Score and parameter ranking.

ME_Score=0.0005×Sensitivity+0.999×(1−FPR)−0.0005×Sigmoid(MNPed)

where

$$Sigmoid(MNPed)=\frac{1}{1+e^{-0.05\times (MNPed-101)}}$$

A two-stage selection strategy was adopted to identify the optimal parameter combination for each algorithm–analyte pair. In the first stage, all candidate configurations were ranked by ME_Score to retain a high-performing subset. In the second stage, this subset was re-evaluated to prioritize clinically meaningful trade-offs, favoring lower FPR, higher sensitivity, and acceptable MNPed in that order. The final selected parameter set for MA, MQ, and EWMA was then applied without further tuning to the independent validation datasets, and performance metrics were recomputed. All numerical outputs (training and validation performance summaries, optimal parameter tables) were exported as .csv files, and diagnostic figures (histograms, performance curves, and SPC control charts) were exported as .png images for further review and comparison across the three SPC algorithms. To reduce spurious single-point alarms, an alarm was triggered only when the PBRTQC statistic exceeded the control limits for k consecutive patient results. For performance evaluation, this k-point alarm run was retrospectively labeled as beginning at the first point of the consecutive sequence.

To evaluate the robustness of the optimized PBRTQC parameters under diverse and challenging clinical scenarios, we conducted a multi-dimensional sensitivity analysis. First, the stability of the algorithms was tested against variations in error patterns. We constructed alternative simulated error configurations by modifying the number, locations, and inter-segment gaps of the systematic error segments. The optimal parameter sets (MA, MQ, and EWMA) derived from the original training process for each analyte were then applied directly to these alternative validation datasets without further tuning. This allowed us to assess the consistency of key performance metrics, including ME_Score, sensitivity, FPR, and MNPed. Second, to address potential real-world analytical instabilities that evolve over time, we performed a separate sensitivity analysis using gradual drifting bias patterns instead of abrupt stepwise shifts. In this scenario, a dynamic drifting bias was introduced to simulate progressive instrument deterioration or reagent degradation. The systematic error originated from a minor baseline deviation and increased linearly over time until reaching a predefined maximum bias level, after which it remained constant for the remainder of the segment. This three-stage pattern (baseline-drift-plateau) was designed to evaluate the PBRTQC framework’s capability to detect evolving analytical instabilities before they stabilize at a clinically significant magnitude. This analysis aimed to determine whether the framework optimized for step-changes could retain clinically acceptable detection capability and alarm stability when faced with slowly evolving analytical drift.

## 3. Results

### 3.1. Characteristics of the Simulated Error Data

Across all six analytes and both data splits, the simulation procedure generated error datasets with a consistent and well-controlled structure (Table 1). For each analyte, 10 distinct bias scenarios (five positive and five negative) were successfully embedded into the transformed patient result series, and five separate error segments were created within each scenario. The realized segment lengths predominantly fell within the intended range of approximately 100–300 consecutive results, and the intervening gaps between segments were generally close to the target spacing of 250–500 unbiased results. The distributions of segment lengths, inter-segment gaps, and the total number of biased observations were initially designed to be consistent across all six analytes (as shown in Table 1) to ensure a standardized evaluation of algorithmic performance. Furthermore, to conduct a rigorous sensitivity analysis, additional error datasets with varying lengths and positions were specifically configured for each analyte. Detailed summaries of these analyte-specific error patterns are provided in Supplementary Tables S1–S6 for TSH, FT3, FT4, APTT, PT, and TT, respectively. These distributions remained comparable between the training and validation sets within each specific test.

### 3.2. Baseline Distributions of Test Parameters

For all six test parameters, the raw patient results exhibited markedly right-skewed distributions with long upper tails (Supplementary Figure S1). After Box-Cox transformation with analyte-specific λ values (e.g., λ ≈ 0.2 for TSH, −0.25 for FT3, −0.05 for FT4, and −1 for APTT, PT, and TT), the histograms became substantially more symmetric, although mild skewness remained for some tests. Subsequent trimming of extreme values based on the interquartile range, applied only for visualization, further yielded approximately bell-shaped distributions for the transformed data across all test parameters. Additionally, the detailed changes in distributional statistics (including skewness and kurtosis results) for each analyte before and after Box-Cox transformation are summarized in Supplementary Table S7.

### 3.3. Optimized PBRTQC Configurations and Comparative Performance of MA, MQ and EWMA

Within the common transform-truncation-alarm framework, all three SPC algorithms converged on compact and clinically interpretable PBRTQC parameter sets for the six laboratory tests. For the EWMA algorithm, the preferred configurations used a smoothing constant λ of 0.9 for all coagulation and thyroid hormone tests except TSH (λ = 0.4), upper and lower control-limit multipliers between 1.64 and 3, no or minimal truncation (0–0.02), and a requirement of five consecutive alarm points (Table 2). Under these settings, EWMA achieved ME_Scores around 0.99 in both the training and validation datasets for every test, with sensitivities generally ≥0.85 and false-positive rates consistently below 0.002; MNPed were small (0–7) and highly consistent between datasets.

The moving average and moving quantile algorithms showed similarly high overall performance but with slightly different optimal parameter patterns (Supplementary Tables S8 and S9). Across all six tests, both MA and MQ favoured a narrow window width of 3 patient results and five or ten consecutive alarm points, again with modest truncation factors. For FT3, FT4, PT, APTT and TT, these settings yielded near-perfect sensitivities (≈0.99–1.00) with false-positive rates between 0.0004 and 0.0027 and MNPed values close to zero in both training and validation sets, indicating rapid and stable detection of simulated biases without inflation of false alarms.

**Figure 1:**
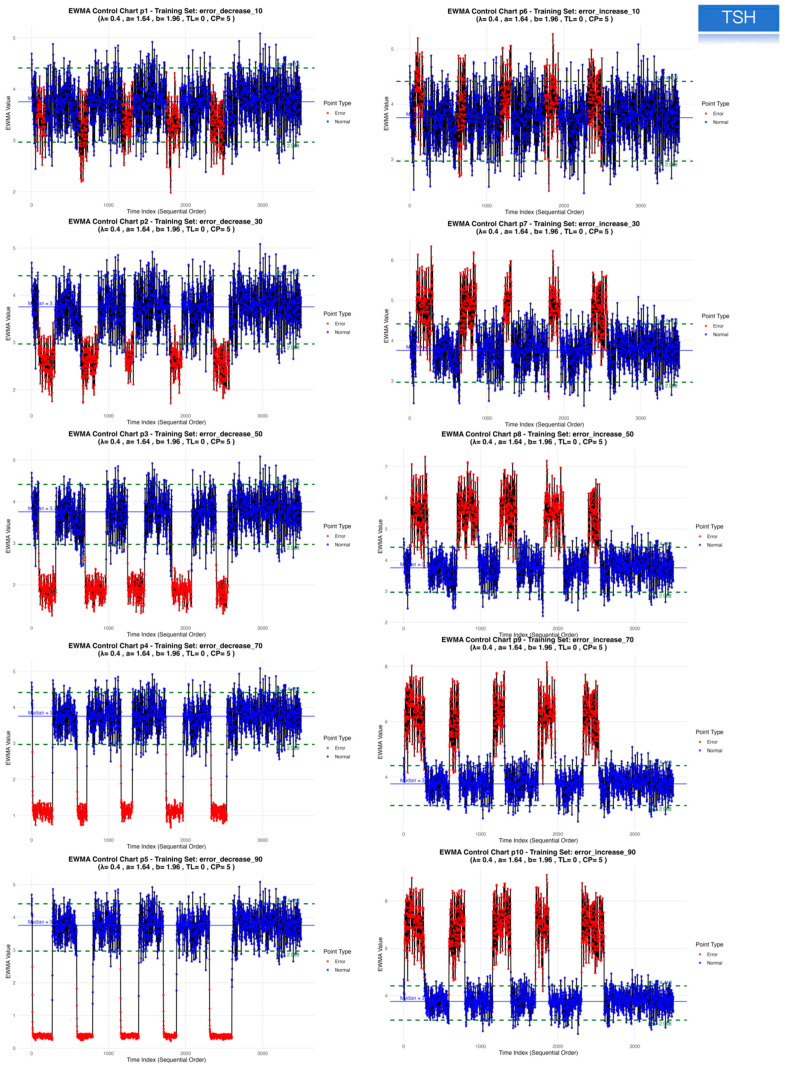
EWMA control charts for TSH in the training dataset under simulated positive and negative bias conditions. This figure shows the EWMA control charts for TSH in the training dataset under ten simulated systematic bias scenarios, including five negative biases (−10%, −30%, −50%, −70%, −90%) and five positive biases (+10%, +30%, +50%, +70%, +90%). Each panel displays the EWMA-transformed patient results in chronological order, with unbiased observations shown in blue and biased observations shown in red. Control limits were constructed using the optimized parameters (smoothing constant λ = 0.4; upper and lower limit multipliers a = 1.64 and b = 1.96; truncation factor = 0; consecutive alarm requirement = 5).

**Figure 2:**
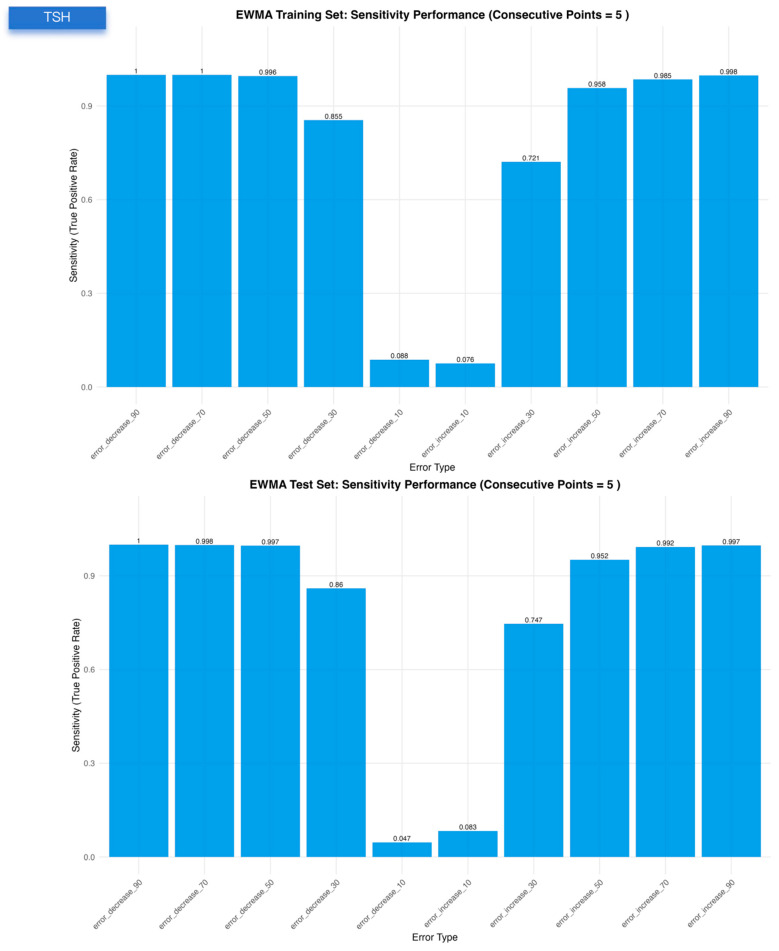

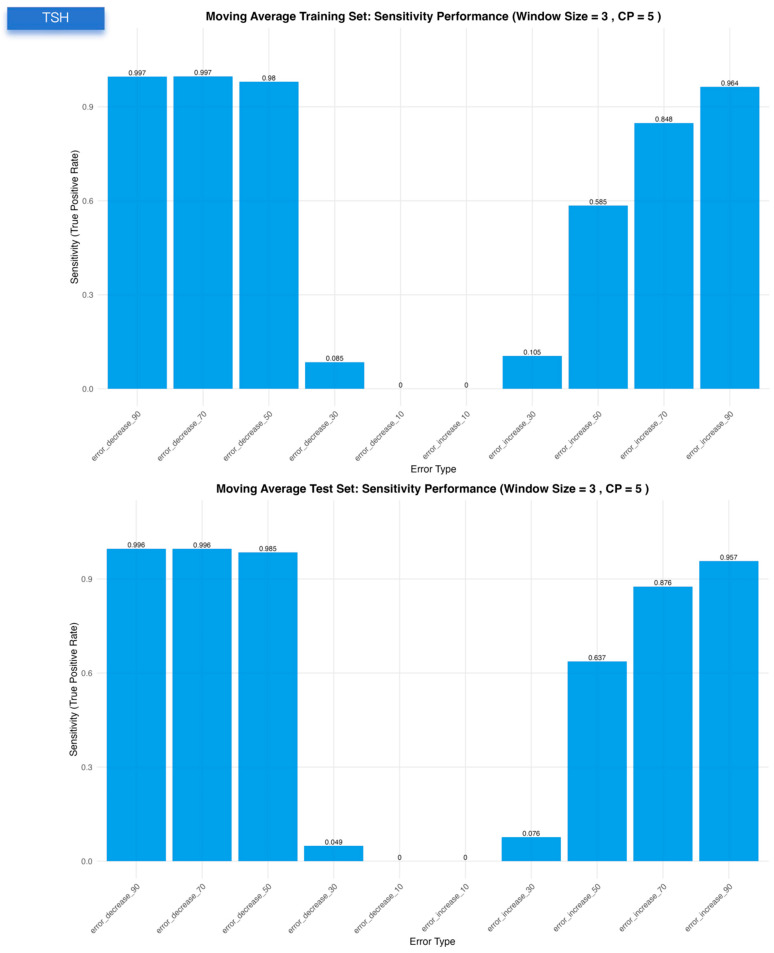

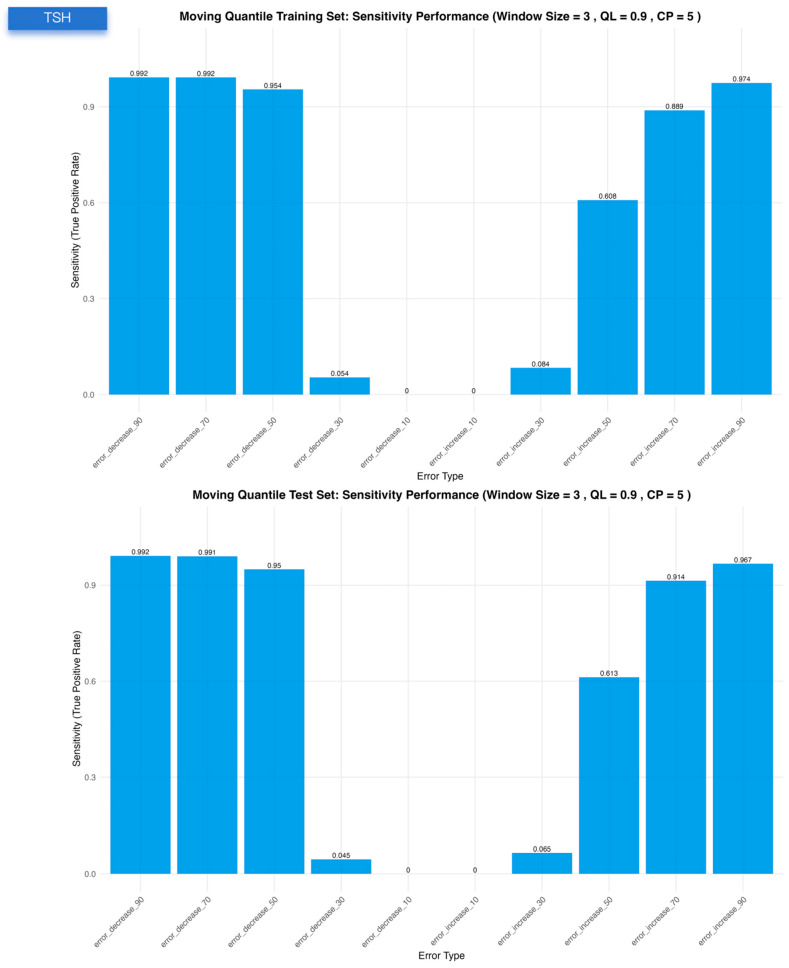
Sensitivity performance of EWMA, moving average, and moving quantile algorithms across ten simulated bias scenarios in the TSH training and validation datasets.

**Figure 3:**
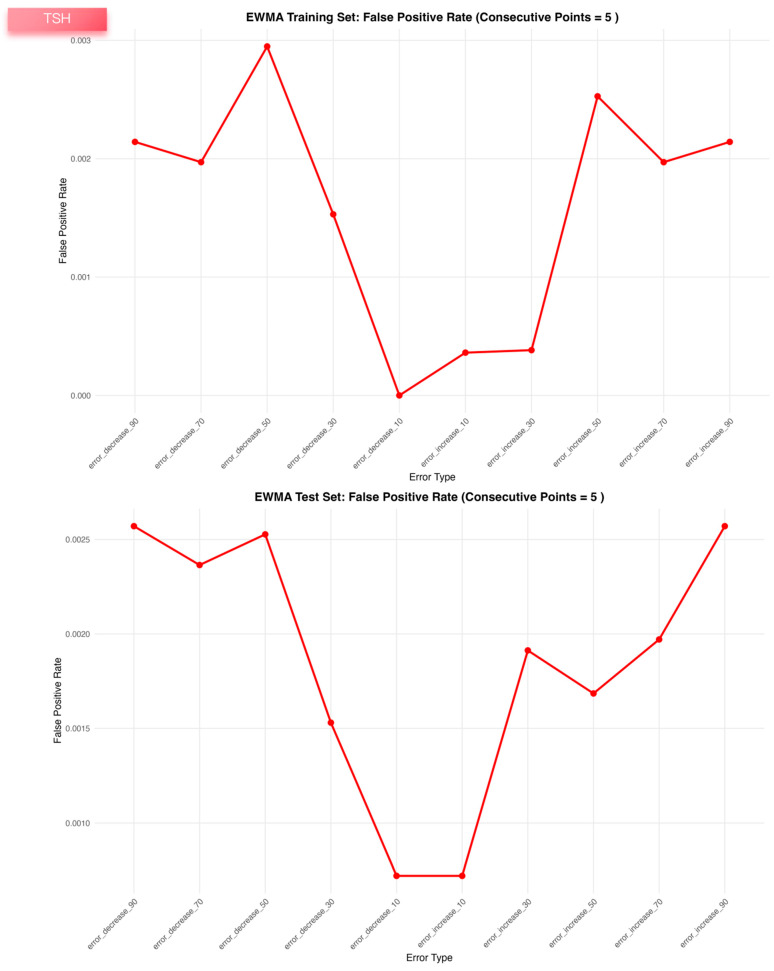

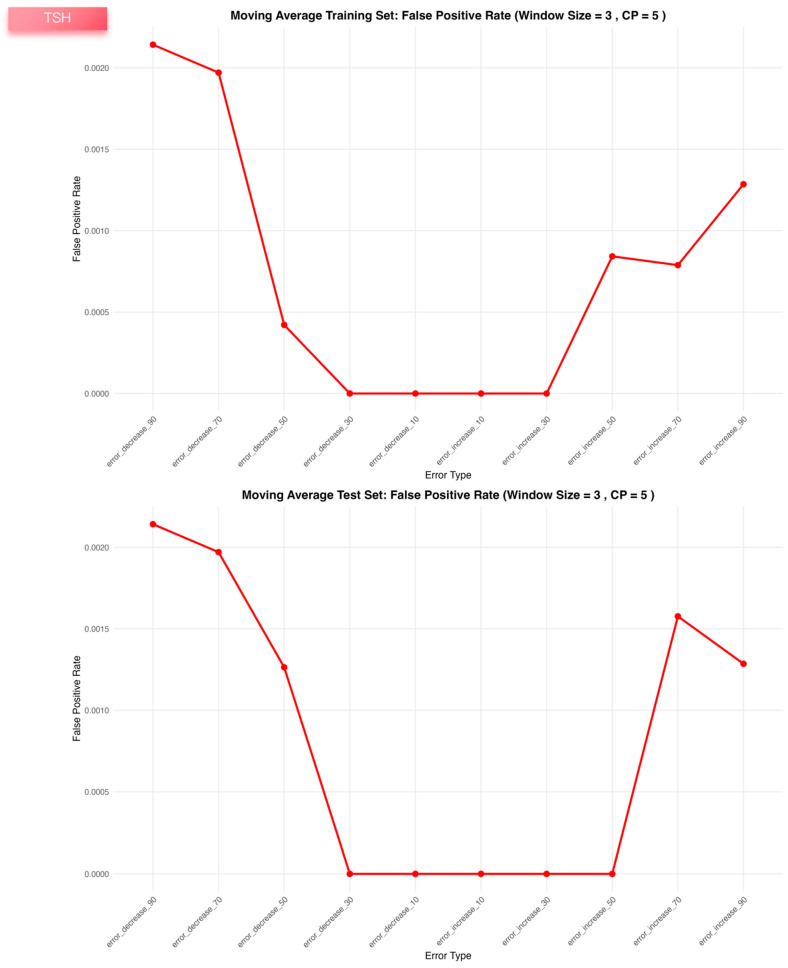

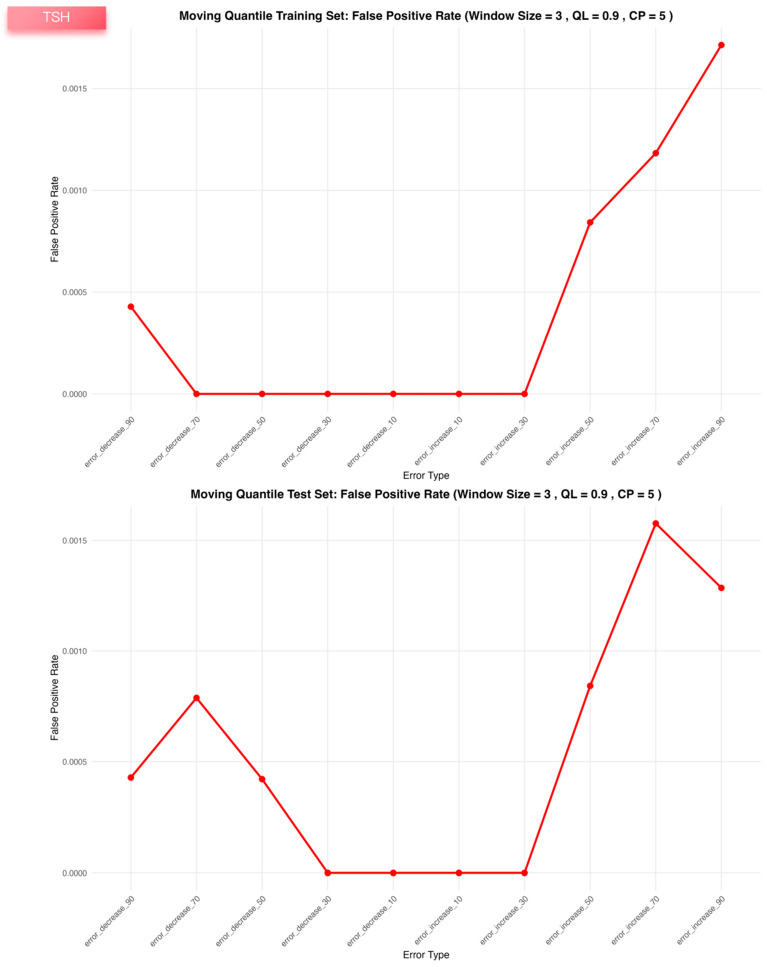
False-positive rates of EWMA, moving average, and moving quantile algorithms across ten simulated bias-free segments in the TSH training and validation datasets.

**Figure 4:**
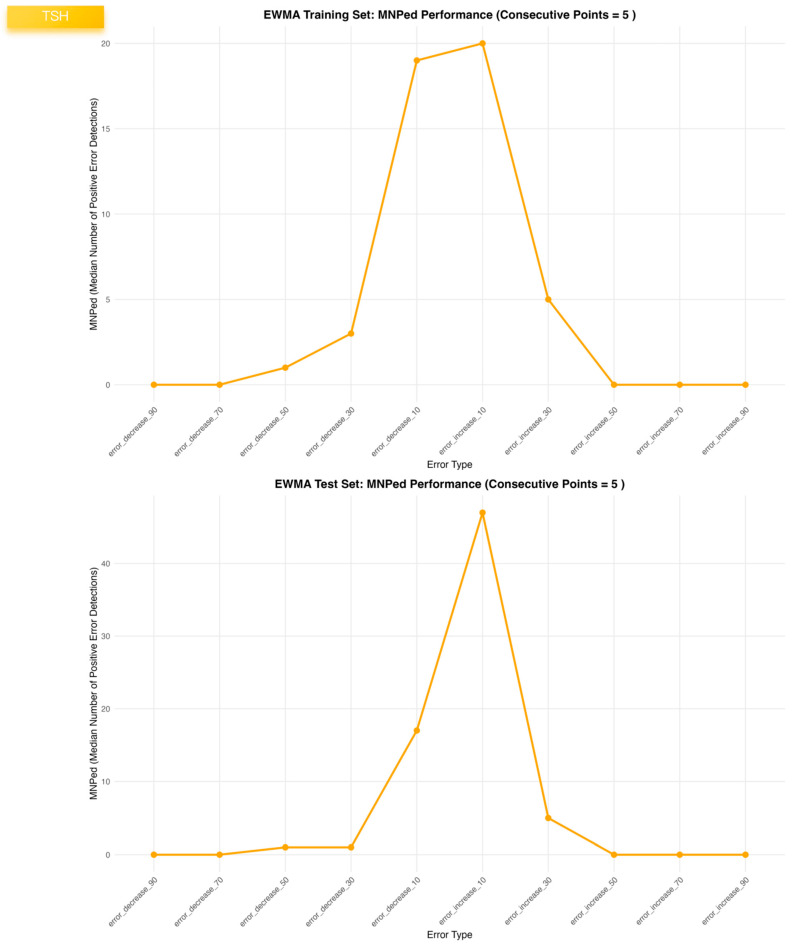

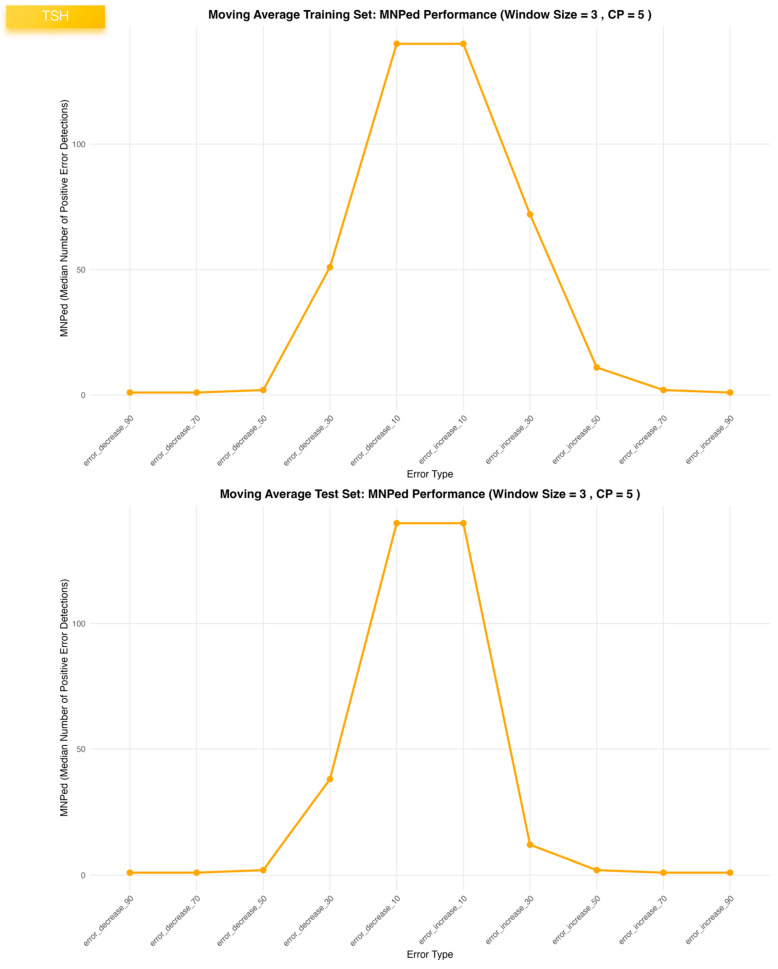

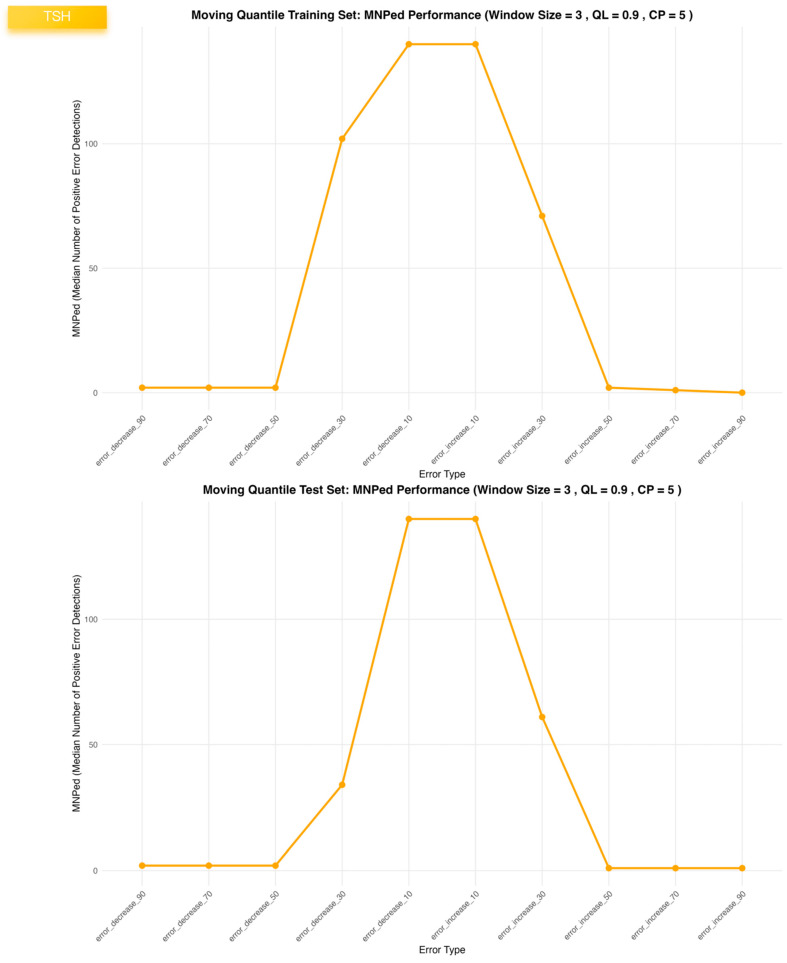
MNPed values of EWMA, moving average, and moving quantile algorithms across ten simulated bias scenarios in the TSH training and validation datasets.

For TSH, the EWMA configuration (λ = 0.4, a = 1.64, b = 1.96, truncation factor 0, five consecutive points) provided a balanced compromise between sensitivity (~0.77 in both datasets), very low false-positive rate (~0.002) and early detection (MNPed 5–7). In contrast, the MA and MQ models achieved slightly higher ME_Scores but at the cost of lower TSH sensitivity (~0.55) and substantially delayed detection (MNPed in the range of 30–40 patient results), despite similarly low false-positive rates. Consistent with these summary metrics, the TSH control charts (Figure 1) show clear segregation of biased segments above or below the control limits for EWMA, and the bias-response curves (Figure 2, Figure 3 and Figure 4) demonstrate that sensitivity approaches 1.0 and MNPed approaches 0 for larger absolute biases (≥50–70%), while 10% biases are detected less frequently and with longer delay. These patterns were qualitatively similar across the remaining five tests, as illustrated by the corresponding control charts and bias–response plots in Supplementary Figures S2–S51.

Each panel displays the sensitivity of a given PBRTQC algorithm under ten predefined bias scenarios for TSH. The upper and lower panels correspond to the training and test datasets, respectively. Bias scenarios include five negative and five positive shifts with increasing magnitudes. Sensitivity was calculated based on successful detection of simulated bias segments under optimized algorithm-specific parameters.

False-positive rates were calculated for the three PBRTQC algorithms evaluated on bias-free data segments for TSH. Results are shown separately for the training (upper panels) and test (lower panels) datasets. False-positive rate was defined as the proportion of unbiased observations triggering false alarms under the same optimized parameter settings used for sensitivity evaluation.

The median number of positive error detections (MNPed) for EWMA, moving average, and moving quantile algorithms was calculated across ten simulated bias scenarios in the TSH datasets. Upper and lower panels represent training and test datasets, respectively. MNPed reflects the cumulative number of alarm signals generated within biased segments and was used as an integrated indicator of detection strength and alarm persistence.

### 3.4. Sensitivity Analysis

Sensitivity analyses were conducted to evaluate the robustness of the optimized PBRTQC parameters under alternative simulated error configurations, in which the locations and numbers of systematic error segments were modified. As shown in Supplementary Tables S10–S12, across all six analytes and for all three SPC algorithms (MA, MQ, and EWMA), the overall performance metrics remained highly consistent with those obtained under the original simulation settings.

In addition, a separate sensitivity analysis was performed using datasets with simulated gradual drift patterns rather than stepwise bias shifts. When the optimized parameter sets derived from the original simulations were applied to these drift-based error datasets without re-optimization, overall PBRTQC performance remained broadly comparable to that observed under step-change scenarios. However, a modest reduction in sensitivity was observed across some analytes, reflecting the increased difficulty of detecting slowly evolving analytical drift compared with abrupt systematic shifts. Importantly, false-positive rates and detection stability were largely preserved, indicating that the optimized parameters retained acceptable robustness under alternative error dynamics (Supplementary Tables S13–S15).

## 4. Discussion

This study systematically evaluated the PBRTQC performance of three classical statistical process control algorithms—MA, MQ, and EWMA—within a unified “transform-truncate-alarm” framework across six commonly used laboratory tests. After applying Box-Cox transformation, truncation-based value replacement, and consecutive alarm rules, all three algorithms demonstrated overall favorable and analyte-dependent performance for FT3, FT4, APTT, PT, and TT, characterized by favorable sensitivity and false-positive rates, and rapid detection of simulated biases with strong consistency across datasets. For TSH, where distributional variability and day-to-day fluctuation were more pronounced, the EWMA method achieved the most balanced performance, exhibiting higher sensitivity and shorter detection delay than MA and MQ. Overall, the proposed preprocessing framework substantially enhanced the robustness of traditional SPC-based PBRTQC methods, enabling near-optimal error detection performance for most analytes.

This study systematically evaluated the PBRTQC performance of three classical statistical process control algorithms—moving average (MA), moving quantile (MQ), and exponentially weighted moving average (EWMA)—within a unified “transform-truncate-alarm” framework across six commonly used laboratory tests. After applying Box-Cox transformation, truncation-based value replacement, and consecutive alarm rules, all three algorithms demonstrated favorable and analyte-dependent performance for FT3, FT4, APTT, PT, and TT, characterized by very high sensitivity, extremely low false-positive rates, and rapid detection of simulated biases with strong consistency across datasets. For TSH, where distributional variability and day-to-day fluctuation were more pronounced, EWMA achieved the most balanced performance, exhibiting higher sensitivity and shorter detection delay than MA and MQ. Overall, the proposed preprocessing framework improved the robustness and practical suitability of SPC-based PBRTQC schemes for most analytes.

A Box-Cox transformation was applied as a pragmatic preprocessing step to improve distributional stability and mitigate the impact of inherent skewness on moving-window statistics. The primary objective was not to impose strict normality or to model the underlying physiological distribution, but to stabilize variance and reduce the influence of skewness so that control limits remain more robust against transient fluctuations in the patient population. This provides a more reliable baseline for the subsequent truncation and consecutive-alarm stages.

PBRTQC performance is fundamentally characterized by three metrics: sensitivity, false-positive rate (FPR), and detection delay (e.g., the median number of patients before error detection, MNPed). These metrics differ not only in scale but also in clinical importance [32], and optimization based on a single indicator or ad hoc combinations may not balance the three dimensions appropriately [33]. Therefore, we developed the ME_Score as a composite target that integrates sensitivity and (1 − FPR) on a common scale while penalizing delayed detection using a nonlinear (sigmoid-type) penalty on MNPed. This design favors parameter sets that maintain very low false-alarm burden, achieve high detection sensitivity, and avoid excessive delay, enabling efficient screening of candidate configurations and facilitating fair comparison across algorithms.

A key rationale of ME_Score is to prioritize low FPR, reflecting the practical reality that excessive alarms can lead to alarm fatigue, increased manual workload, and reduced clinical acceptance in high-frequency PBRTQC monitoring. The dominant weighting of (1 − FPR) implicitly corresponds to a false-positive tolerance on the order of 10^−3^, a level commonly viewed as operationally acceptable in routine laboratories. At the same time, the weighting strategy is context-dependent and can be adapted to different analytes, testing volumes, and risk tolerances. Our results also illustrate why sensitivity-only criteria can be operationally impractical: when EWMA parameters for TSH were optimized using the Youden index, the resulting setting achieved high sensitivity (≈0.91) and short delay (MNPed = 5) but increased the false-positive rate to ~1%, which would generate excessive alarms when deployed across multiple analytes. Accordingly, our optimization strategy prioritizes stringent FPR control and then improves sensitivity and detection speed within this constrained operating space, consistent with PBRTQC as a complementary quality management tool.

Within the proposed transform-truncate-alarm framework, the three algorithms performed consistently well for analytes with relatively stable distributions such as FT3, FT4, APTT, PT, and TT, rapidly detecting moderate-to-large biases and maintaining good sensitivity even for smaller shifts. Notably, the consecutive-alarm rule tended to favor smaller window widths (typically width = 3) in optimal configurations because consecutive alarms filter isolated fluctuations that would otherwise inflate false positives, allowing more responsive settings without compromising FPR. For TSH, EWMA clearly outperformed MA and MQ. Prior work has also reported that PBRTQC may be less effective for TSH than for other thyroid analytes, likely due to greater within-subject variability and instability of mean-based statistics [34]. Our findings extend this observation by showing that EWMA, combined with the transform-truncate-alarm workflow and consecutive alarm rules, can provide faster and more stable detection for TSH. This advantage is plausibly attributable to EWMA’s emphasis on recent observations and its smoother statistic, which helps maintain uninterrupted alarm sequences under noisy conditions. Regarding MNPed = 0, this can occur under consecutive-alarm logic because once k consecutive points exceed limits, the alarm sequence is retrospectively attributed to the first point of that run; therefore, if the run begins at the start of an injected error segment, the calculated delay becomes zero. In practical terms, the observed delay of ~5–7 patients for thyroid-related tests mainly reflects the difficulty of detecting very small shifts (e.g., 10% bias) and is generally acceptable in routine settings, especially when results are reviewed promptly once a signal is generated.

The proposed framework has several strengths: (1) methodological simplicity, transparency, and reproducibility suitable for routine laboratory practice; (2) improved stability of SPC algorithms for skewed or heavy-tailed data through transformation and truncation; (3) substantial reduction of false positives via consecutive alarms; and (4) a unified composite metric that streamlines parameter selection and supports standardized comparison across algorithms. Several limitations should be acknowledged. First, simulated error patterns (with fixed magnitudes and segment structures) may not fully capture the diversity of real-world analytical errors, and performance under retrospective error labeling may optimistically estimate detection timeliness, particularly when MNPed approaches zero. Second, this was a single-center study with one instrument platform per analyte and a limited observation period; differences in patient case mix, analytical systems, and lot-to-lot variability could influence baseline distributions and detection behavior. Third, while the study focused on classical SPC algorithms for transparency and ease of deployment, we did not directly benchmark advanced machine learning-based PBRTQC models within the same framework. Future work incorporating real-world error events, multicenter data, and broader platform/lot variability will be important to evaluate generalizability and refine analyte-adaptive parameterization.

Beyond methodological performance, practical implementation is critical for PBRTQC adoption. The transform-truncate-alarm framework relies on simple moving-window calculations and summary statistics and can be implemented using standard laboratory computing infrastructure without specialized hardware. From an integration standpoint, it requires only routinely available patient results and timestamps and can be deployed alongside existing laboratory information systems or middleware. Importantly, it is intended to complement rather than replace conventional internal quality control. In future studies, we also plan to extend the unified framework to additional classical monitoring approaches, such as cumulative sum control charts, which can be sensitive to small persistent shifts but require careful parameterization for routine use [35,36]. Overall, our findings support a practical pathway toward more standardized, analyte-adaptive, and clinically acceptable PBRTQC implementation in routine clinical laboratories.

## 5. Conclusions

This study evaluated three classical SPC algorithms—MA, MQ, and EWMA—within a unified “transform–truncate–alarm” PBRTQC framework and found that all three algorithms provided consistently high performance for most laboratory tests, with EWMA showing the most balanced behavior for TSH. The proposed ME_Score enabled coherent parameter selection by jointly considering sensitivity, false-positive rate, and detection delay. These results support the feasibility of using a standardized preprocessing workflow to compare and optimize PBRTQC methods across different analytes.

## Figures and Tables

**Table 1:** Error segment lengths and gaps summary table.

Data	Error Type	Gap-1	Segment 1 Count	Gap 1–2	Segment 2 Count	Gap 2–3	Segment 3 Count	Gap 3–4	Segment 4 Count	Gap 4–5	Segment 5 Count	Gap 5-
Training Set	error_decrease_10	64	114	438	111	451	140	431	183	386	185	997
	error_increase_10	64	114	438	111	451	140	431	183	386	185	997
	error_decrease_30	86	216	348	213	354	104	483	146	409	207	934
	error_increase_30	86	216	348	213	354	104	483	146	409	207	934
	error_decrease_50	92	221	379	275	278	221	354	256	319	153	952
	error_increase_50	92	221	379	275	278	221	354	256	319	153	952
	error_decrease_70	11	261	319	126	443	148	436	223	361	205	967
	error_increase_70	11	261	319	126	443	148	436	223	361	205	967
	error_decrease_90	9	259	318	207	361	236	319	172	427	292	900
	error_increase_90	9	259	318	207	361	236	319	172	427	292	900
Test Set	error_decrease_10	64	114	438	111	451	140	431	183	386	185	997
	error_increase_10	64	114	438	111	451	140	431	183	386	185	997
	error_decrease_30	86	216	348	213	354	104	483	146	409	207	934
	error_increase_30	86	216	348	213	354	104	483	146	409	207	934
	error_decrease_50	92	221	379	275	278	221	354	256	319	153	952
	error_increase_50	92	221	379	275	278	221	354	256	319	153	952
	error_decrease_70	11	261	319	126	443	148	436	223	361	205	967
	error_increase_70	11	261	319	126	443	148	436	223	361	205	967
	error_decrease_90	9	259	318	207	361	236	319	172	427	292	900
	error_increase_90	9	259	318	207	361	236	319	172	427	292	900

This table summarizes the structure of the simulated error segments used for PBRTQC model development and validation. For each analyte, ten bias scenarios (five positive and five negative) were generated, with five discrete error segments embedded in each scenario. The lengths of individual error segments generally fell within the predefined range of approximately 100–300 consecutive patient results, and the numbers of unbiased observations between adjacent segments were close to the intended spacing. Note: Gap X-Y count: Represents the number of non-error data points (i.e., unaffected by simulated errors) found between the X-th and Y-th error segments. Gap-1: Refers to the number of non-error data points before the first simulated error segment. Segment X count: Denotes the number of data points within the X-th simulated error segment (i.e., the contiguous block where an error was injected). Gap 5-: Represents the number of non-error data points after the fifth (and final) simulated error segment.

**Table 2:** Recommended PBRTQC parameters and performance metrics for the exponentially weighted moving average algorithm.

Analytes	Lambda	Upper limit Multiplier (a)	Lower limit Multiplier (b)	Truncation Factor	Consecutive Alarm Points	Data	ME_Score	Sensitivity	False-Positive Rate	MNPed
TSH	0.4	1.64	1.96	0	5	Training Set	0.9922	0.7677	0.0016	5
						Test Set	0.9919	0.7673	0.0019	7
FT3	0.9	1.96	3	0	5	Training Set	0.9940	0.9623	0.0008	0
						Test Set	0.9941	0.9631	0.0007	0
FT4	0.9	1.96	1.64	0.02	5	Training Set	0.9936	0.8622	0.0007	3
						Test Set	0.9936	0.8559	0.0006	7
PT	0.9	3	3	0	5	Training Set	0.9933	1.0000	0.0017	0
						Test Set	0.9933	1.0000	0.0017	0
APTT	0.9	3	3	0	5	Training Set	0.9932	1.0000	0.0018	0
						Test Set	0.9932	1.0000	0.0018	0
TT	0.9	3	3	0	5	Training Set	0.9932	1.0000	0.0018	0
						Test Set	0.9932	1.0000	0.0018	0

This table summarizes the optimized PBRTQC parameters and corresponding performance metrics for the EWMA algorithm across the six analytes in both the training and validation datasets.

## Data Availability

The original contributions presented in this study are included in the article/Supplementary Materials. Further inquiries can be directed to the corresponding authors. All computational scripts developed for this study are openly available on GitHub at: https://github.com/512800245/PBRTQC_pipeline (accessed on 2 December 2025). The repository contains complete R scripts for automated PBRTQC analysis, including modules for MA, MQ, and EWMA algorithms, as well as a user dispatcher script for unified execution. An example dataset (exampledata.xls) is also provided for demonstration and reproducibility testing.

## Supplementary Materials

The following supporting information can be downloaded at: https://www.mdpi.com/article/10.3390/diagnostics16020288/s1.

## Abbreviations

APTT	activated partial thromboplastin time
Box-Cox	Box-Cox transformation
EWMA	exponentially weighted moving average
FPR	false-positive rate
FT3	free triiodothyronine
FT4	free thyroxine
MA	moving average
ME_Score	model evaluation score
MNPed	median number of patients before error detection
MQ	moving quantile
PBRTQC	patient-based real-time quality control
PT	prothrombin time
SPC	statistical process control
TSH	thyroid-stimulating hormone
TT	thrombin time
