# Supplementary material for: Comparison of EWMA, MA, and MQ Under a Unified PBRTQC Framework for Thyroid and Coagulation Tests
Source: Diagnostics (Basel). 2026 Jan 16;16(2):288. doi: 10.3390/diagnostics16020288 (PMC12839619; doi:10.3390/diagnostics16020288)
Supplement: Supplementary file 1 [file diagnostics-16-00288-s001.zip › Supplemental Figure S1.pdf]

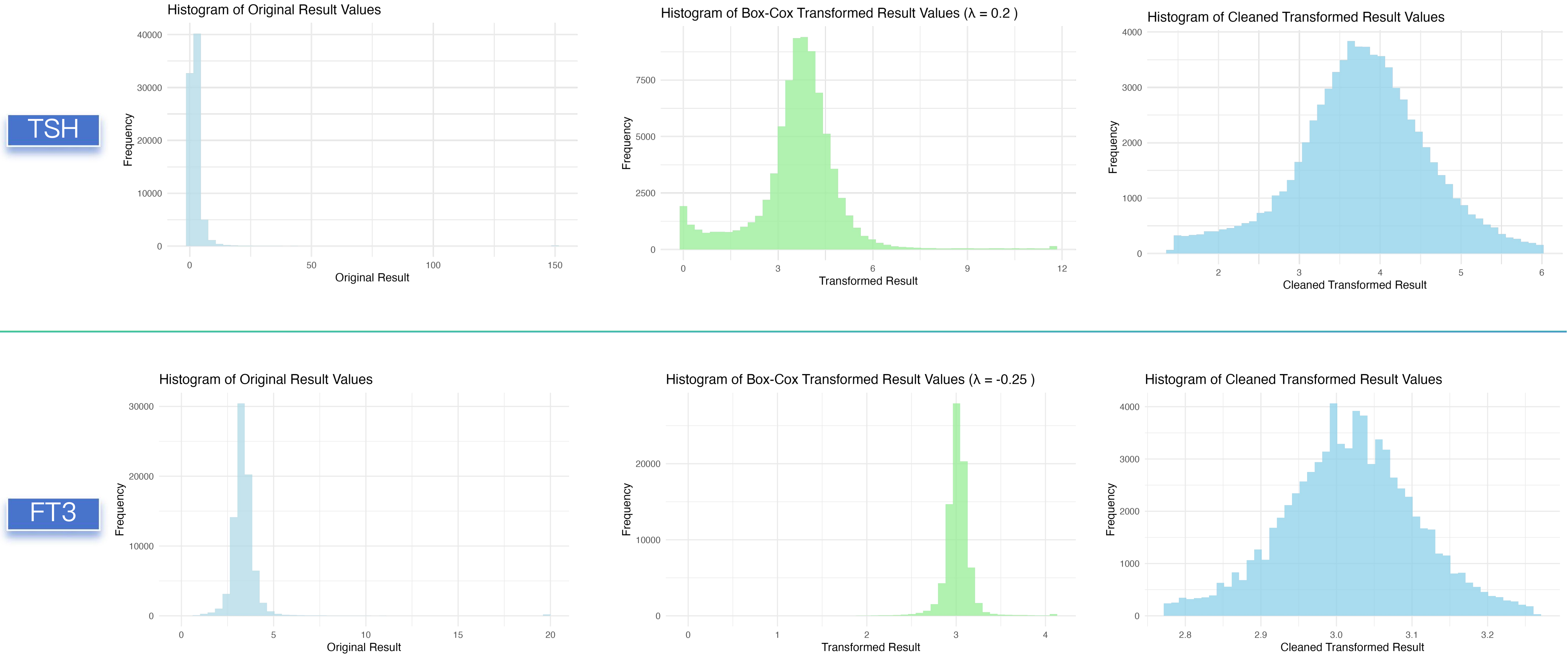
TSH
Histogram of Original Result Values
Frequency
Original Result
0
10000
20000
30000
40000
0
50
100
150
Histogram of Box-Cox Transformed Result Values (λ = 0.2)
Frequency
Transformed Result
0
2500
5000
7500
0
3
6
9
12
Histogram of Cleaned Transformed Result Values
Frequency
Cleaned Transformed Result
0
1000
2000
3000
4000
2
3
4
5
6
FT3
Histogram of Original Result Values
Frequency
Original Result
0
10000
20000
30000
0
5
10
15
20
Histogram of Box-Cox Transformed Result Values (λ = -0.25)
Frequency
Transformed Result
0
10000
20000
0
1
2
3
4
Histogram of Cleaned Transformed Result Values
Frequency
Cleaned Transformed Result
0
1000
2000
3000
4000
2.8
2.9
3.0
3.1
3.2

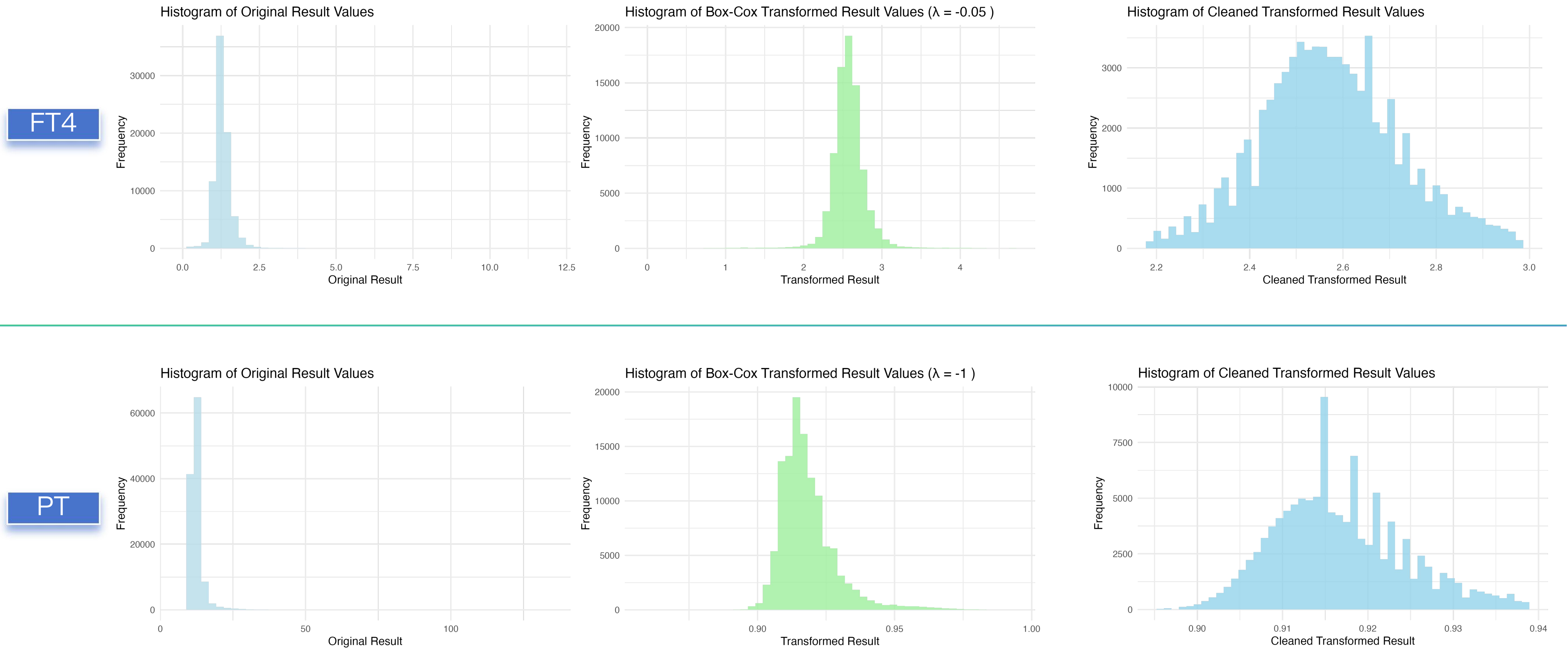
FT4
Histogram of Original Result Values
Frequency
Original Result
Histogram of Box-Cox Transformed Result Values (λ = -0.05)
Transformed Result
Histogram of Cleaned Transformed Result Values
Cleaned Transformed Result
PT
Histogram of Original Result Values
Frequency
Original Result
Histogram of Box-Cox Transformed Result Values (λ = -1)
Transformed Result
Histogram of Cleaned Transformed Result Values
Cleaned Transformed Result

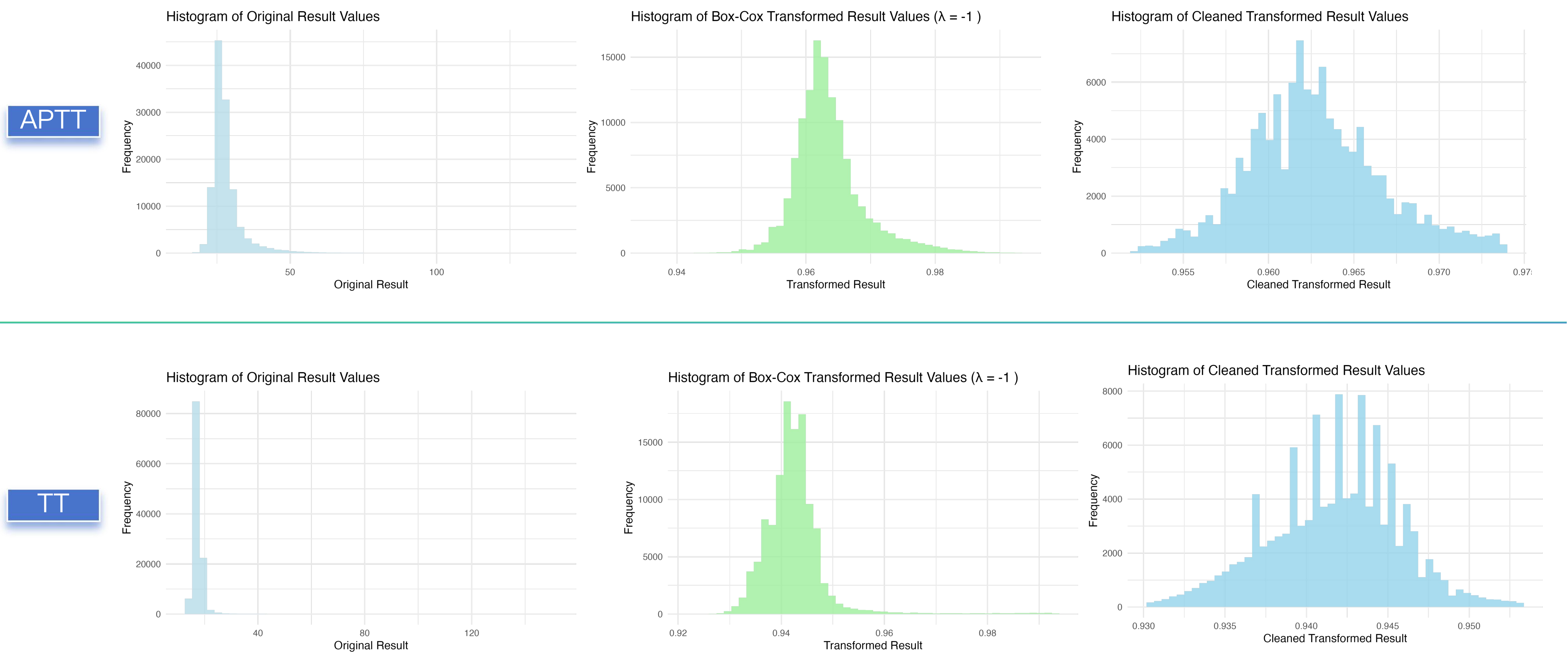
APTT
Histogram of Original Result Values
Frequency
Original Result
Histogram of Box-Cox Transformed Result Values (λ = -1)
Frequency
Transformed Result
Histogram of Cleaned Transformed Result Values
Frequency
Cleaned Transformed Result
TT
Histogram of Original Result Values
Frequency
Original Result
Histogram of Box-Cox Transformed Result Values (λ = -1)
Frequency
Transformed Result
Histogram of Cleaned Transformed Result Values
Frequency
Cleaned Transformed Result
