# Supplementary material for: Comparison of EWMA, MA, and MQ Under a Unified PBRTQC Framework for Thyroid and Coagulation Tests
Source: Diagnostics (Basel). 2026 Jan 16;16(2):288. doi: 10.3390/diagnostics16020288 (PMC12839619; doi:10.3390/diagnostics16020288)
Supplement: Supplementary file 1 [file diagnostics-16-00288-s001.zip › Supplementary Methods.pdf]

Supplementary Methods

Supplementary Methods Statistical process control algorithms for PBRTQC

S1.Moving average (MA)

The moving average (MA) algorithm monitors analytical stability by computing the arithmetic mean of consecutive patient results within a sliding window. At each time point *t*, the MA statistic is calculated as:

$$MA_t = \frac{1}{w} \sum_{i=t-w+1}^{t} X_i$$

where Xi denotes the Box–Cox–transformed patient result and w is the window width. To derive control limits, the distribution of all MA_t values obtained from the training dataset is summarized using robust statistics. The upper and lower control limits are defined as:

UL=Median(MA)+a×MAD(MA), LL=Median(MA)−b×MAD(MA)

where a and b are scaling multipliers for the upper and lower limits, respectively, and MAD denotes the median absolute deviation.

A systematic bias is signaled when a predefined number of consecutive MA_t values exceed the upper control limit or fall below the lower control limit. The MA parameter space explored in this study included multiple window widths, control-limit multipliers, truncation factors, and consecutive-alarm requirements. The algorithm outputs optimized parameters together with detection performance metrics and corresponding SPC control charts.

S2. Moving quantile (MQ)

The moving quantile (MQ) algorithm extends the MA concept by replacing the arithmetic mean with a quantile-based statistic, thereby improving robustness to skewed distributions and extreme observations. For each sliding window of width w, the MQ statistic at time point t is defined as:

$$MQ_t = Q_p(X_{t-w+1}, \cdots, X_t)$$

where $Q_p(\cdot)$ represents the empirical quantile at probability level p. Control limits are constructed using the robust summary statistics of the full sequence of MQt values derived from the training data:

UL=Median(MQ)+a×MAD(MQ), LL=Median(MQ)−b×MAD(MQ)

with a and b denoting the upper and lower scaling multipliers.

Bias detection is triggered when the specified number of consecutive MQ statistics crosses either control limit. The MQ parameter space included window width, quantile level, control-limit multipliers, truncation factor, and the number of consecutive alarm points. The output consists of optimized MQ parameters, detection performance summaries, and graphical SPC representations.

S1.3. Exponentially weighted moving average (EWMA)

The exponentially weighted moving average (EWMA) algorithm assigns exponentially decreasing weights to historical observations, allowing enhanced sensitivity to small and gradual analytical shifts. The EWMA statistic is computed recursively as:

$$Z_t = \lambda X_t + (1 - \lambda) Z_{t-1}$$

where *Xt* denotes the Box–Cox–transformed patient result at time t, λ is the smoothing constant ($0<\lambda\leq1$), and *Zt* is the EWMA statistic. The initial value is defined as $Z_1=X_1$. Control limits are established using the median and MAD of the EWMA statistics calculated from the training dataset:

UL=Median(Z)+a×MAD(Z), LL=Median(Z)−b×MAD(Z)

where a and b are the scaling factors for the upper and lower limits.

A bias is considered detected when a predefined number of consecutive EWMA statistics exceed the control limits. The EWMA parameter space explored in this study included the smoothing constant λ, control-limit multipliers, truncation factor, and consecutive-alarm requirement. The algorithm outputs the optimized EWMA configuration, detection performance metrics, and corresponding SPC charts.

Parameter space for PBRTQC algorithm optimization

The following parameter spaces were defined for parameter optimization of different SPC-based PBRTQC algorithms. For each algorithm, a set of commonly used and practically relevant parameters was specified, and all parameters were optimized jointly within the unified transform–truncate–alarm framework.

Parameter space for moving average (MA)–based PBRTQC

Parameter space:

- Window width: 3, 5, 7, 10, 15, 20, 25, 30
- Upper limit multiplier (a): 1.64, 1.96, 3
- Lower limit multiplier (b): 1.64, 1.96, 3
- Truncation factor (tl): 0 (none), 0.02, 0.05, 0.1
- Consecutive alarm points: 1, 5, 10 (Consecutive alarm points refer to the number of consecutive data points (patient results) that must exceed the predefined control limit before the system triggers an alarm signal. This parameter helps to balance sensitivity (ability to detect true errors quickly) and specificity (avoidance of false alarms).)

Parameter space for exponentially weighted moving average (EWMA)–based PBRTQC

Parameter space

- Smoothing constant (λ): 0.1, 0.2, 0.3, 0.4, 0.5, 0.6, 0.7, 0.8, 0.9
- Upper limit multiplier (a): 1.64, 1.96, 3
- Lower limit multiplier (b): 1.64, 1.96, 3
- Truncation factor (tl): 0 (none), 0.02, 0.05, 0.1
- Consecutive alarm points: 1, 5, 10

Parameter space for moving quantile (MQ)–based PBRTQC

Parameter space:

- Window width: 3, 5, 7, 10, 15, 20, 25, 30
- Quantile levels: 0.1–0.9 (in increments of 0.1)
- Upper limit multiplier (a): 1.64, 1.96, 3
- Lower limit multiplier (b): 1.64, 1.96, 3
- Truncation factor (tl): 0 (none), 0.02, 0.05, 0.1
- Consecutive alarm points: 1, 5, 10

Using the investigators’ computational environment as an example (Mac mini, Apple M4 Pro chip, 48 GB RAM, macOS Tahoe 26.1), the pipeline was implemented with multi-processing enabled. Under this setting, the average computational time required

to complete grid-search optimization for a single analyte was approximately 3 minutes for EWMA, 3 minutes for MA, and around 20 minutes for MQ
