# Supplementary material for: Comparison of EWMA, MA, and MQ Under a Unified PBRTQC Framework for Thyroid and Coagulation Tests
Source: Diagnostics (Basel). 2026 Jan 16;16(2):288. doi: 10.3390/diagnostics16020288 (PMC12839619; doi:10.3390/diagnostics16020288)
Supplement: Supplementary file 1 [file diagnostics-16-00288-s001.zip › Supplementary Table S1.pdf]

Supplementary Table S1 Error segment lengths and gaps summary table for TSH

Data	Error type	Gap -1	Segment 1 count	Gap 1-2	Segment 2 count	Gap 2-3	Segment 3 count	Gap 3-4	Segment 4 count	Gap 4-5	Segment 5 count	Gap 5-
Training Set	error_decrease_10	70	232	349	204	356	239	338	260	303	149	1000
Training Set	error_increase_10	70	232	349	204	356	239	338	260	303	149	1000
Training Set	error_decrease_30	11	166	420	281	281	219	377	157	420	211	957
Training Set	error_increase_30	11	166	420	281	281	219	377	157	420	211	957
Training Set	error_decrease_50	78	171	425	237	353	235	318	282	278	261	862
Training Set	error_increase_50	78	171	425	237	353	235	318	282	278	261	862
Training Set	error_decrease_70	68	291	285	119	450	251	344	145	420	131	996
Training Set	error_increase_70	68	291	285	119	450	251	344	145	420	131	996
Training Set	error_decrease_90	72	233	367	129	462	268	286	194	358	282	849
Training Set	error_increase_90	72	233	367	129	462	268	286	194	358	282	849
Test Set	error_decrease_10	70	232	349	204	356	239	338	260	303	149	1000
Test Set	error_increase_1	70	232	349	204	356	239	338	260	303	149	1000

	0											
Test Set	error_decrease_30	11	166	420	281	281	219	377	157	420	211	957
Test Set	error_increase_30	11	166	420	281	281	219	377	157	420	211	957
Test Set	error_decrease_50	78	171	425	237	353	235	318	282	278	261	862
Test Set	error_increase_50	78	171	425	237	353	235	318	282	278	261	862
Test Set	error_decrease_70	68	291	285	119	450	251	344	145	420	131	996
Test Set	error_increase_70	68	291	285	119	450	251	344	145	420	131	996
Test Set	error_decrease_90	72	233	367	129	462	268	286	194	358	282	849
Test Set	error_increase_90	72	233	367	129	462	268	286	194	358	282	849
