# Supplementary material for: Comparison of EWMA, MA, and MQ Under a Unified PBRTQC Framework for Thyroid and Coagulation Tests
Source: Diagnostics (Basel). 2026 Jan 16;16(2):288. doi: 10.3390/diagnostics16020288 (PMC12839619; doi:10.3390/diagnostics16020288)
Supplement: Supplementary file 1 [file diagnostics-16-00288-s001.zip › Supplementary Table S10.pdf]

Supplementary Table S10. Sensitivity analysis of PBRTQC performance for EWMA under varying simulated error locations and segment numbers

Analytes	Lambda	Upper limit multiplier (a)	Lower limit multiplier (b)	Truncation factor	Consecutive alarm points	ME_Score	Sensitivity	False positive rate	MNPed
TSH	0.4	1.64	1.96	0	5	0.9920	0.7577	0.0015	16.4
FT3	0.9	1.96	3	0	5	0.9938	0.9661	0.0009	0.4
FT4	0.9	1.96	1.64	0.02	5	0.9937	0.8618	0.0006	5
PT	0.9	3	3	0	5	0.9935	1.0000	0.0015	0
APTT	0.9	3	3	0	5	0.9932	1.0000	0.0018	0
TT	0.9	3	3	0	5	0.9932	1.0000	0.0018	0
