# Supplementary material for: Comparison of EWMA, MA, and MQ Under a Unified PBRTQC Framework for Thyroid and Coagulation Tests
Source: Diagnostics (Basel). 2026 Jan 16;16(2):288. doi: 10.3390/diagnostics16020288 (PMC12839619; doi:10.3390/diagnostics16020288)
Supplement: Supplementary file 1 [file diagnostics-16-00288-s001.zip › Supplementary Table S11.pdf]

Supplementary Table S11. Sensitivity analysis of PBRTQC performance for MA under varying simulated error locations and segment numbers

Analytes	Window width	Upper limit multiplier (a)	Lower limit multiplier (b)	Truncation factor	Consecutive alarm points	ME_Score	Sensitivity	False positive rate	MNPed
TSH	3	3	3	0.05	5	0.9984	0.5617	0.0008	59
FT3	3	1.64	1.64	0.1	10	0.9975	0.9997	0.0020	0
FT4	3	3	3	0.05	5	0.9978	0.8662	0.0016	2
PT	3	1.64	1.64	0	10	0.9975	1.0000	0.0020	0
APTT	3	1.96	1.64	0	10	0.9975	1.0000	0.0020	0
TT	3	1.64	1.96	0	10	0.9975	1.0000	0.0020	0
