# Supplementary material for: Comparison of EWMA, MA, and MQ Under a Unified PBRTQC Framework for Thyroid and Coagulation Tests
Source: Diagnostics (Basel). 2026 Jan 16;16(2):288. doi: 10.3390/diagnostics16020288 (PMC12839619; doi:10.3390/diagnostics16020288)
Supplement: Supplementary file 1 [file diagnostics-16-00288-s001.zip › Supplementary Table S13.pdf]

Supplementary Table S13. Sensitivity analysis of EWMA-based PBRTQC under drifting systematic bias

Analytes	Lambda	Upper limit multiplier (a)	Lower limit multiplier (b)	Truncation factor	Consecutive alarm points	ME_Score	Sensitivity	False positive rate	MNPed
TSH	0.4	1.64	1.96	0	5	0.9920	0.7491	0.0016	7
FT3	0.9	1.96	3	0	5	0.9940	0.9585	0.0008	2
FT4	0.9	1.96	1.64	0.02	5	0.9936	0.8526	0.0006	5
PT	0.9	3	3	0	5	0.9933	0.9992	0.0016	0.1
APTT	0.9	3	3	0	5	0.9932	0.9999	0.0018	0
TT	0.9	3	3	0	5	0.9931	1.0000	0.0019	0
