# Supplementary material for: Comparison of EWMA, MA, and MQ Under a Unified PBRTQC Framework for Thyroid and Coagulation Tests
Source: Diagnostics (Basel). 2026 Jan 16;16(2):288. doi: 10.3390/diagnostics16020288 (PMC12839619; doi:10.3390/diagnostics16020288)
Supplement: Supplementary file 1 [file diagnostics-16-00288-s001.zip › Supplementary Table S14.pdf]

Supplementary Table S14. Sensitivity analysis of MA-based PBRTQC under drifting systematic bias

Analytes	Window width	Upper limit multiplier (a)	Lower limit multiplier (b)	Truncation factor	Consecutive alarm points	ME_Score	Sensitivity	False positive rate	MNPed
TSH	3	3	3	0.05	5	0.9984	0.5386	0.0007	49
FT3	3	1.64	1.64	0.1	10	0.9975	0.9999	0.0020	0
FT4	3	3	3	0.05	5	0.9979	0.8531	0.0016	5
PT	3	1.64	1.64	0	10	0.9976	0.9991	0.0019	0.2
APTT	3	1.96	1.64	0	10	0.9975	0.9999	0.0020	0
TT	3	1.64	1.96	0	10	0.9974	0.9999	0.0021	0
