# Supplementary material for: Comparison of EWMA, MA, and MQ Under a Unified PBRTQC Framework for Thyroid and Coagulation Tests
Source: Diagnostics (Basel). 2026 Jan 16;16(2):288. doi: 10.3390/diagnostics16020288 (PMC12839619; doi:10.3390/diagnostics16020288)
Supplement: Supplementary file 1 [file diagnostics-16-00288-s001.zip › Supplementary Table S15.pdf]

Supplementary Table S15. Sensitivity analysis of MQ-based PBRTQC under drifting systematic bias

Analytes	Window width	Quantile Level	Upper limit multiplier (a)	Lower limit multiplier (b)	Truncation factor	Consecutive alarm points	ME_Score	Sensitivity	False positive rate	MNPed
TSH	3	0.9	3	3	0	5	0.9987	0.5365	0.0004	51
FT3	3	0.1	3	3	0	5	0.9975	0.9965	0.0020	0.6
FT4	3	0.9	1.96	3	0	5	0.9981	0.8571	0.0013	6
PT	3	0.9	1.64	3	0	10	0.9977	0.9951	0.0018	0.8
APTT	3	0.6	3	1.64	0	10	0.9975	0.9973	0.0020	0.6
TT	3	0.6	1.64	1.96	0	10	0.9974	0.9977	0.0021	0.5
