# Supplementary material for: Comparison of EWMA, MA, and MQ Under a Unified PBRTQC Framework for Thyroid and Coagulation Tests
Source: Diagnostics (Basel). 2026 Jan 16;16(2):288. doi: 10.3390/diagnostics16020288 (PMC12839619; doi:10.3390/diagnostics16020288)
Supplement: Supplementary file 1 [file diagnostics-16-00288-s001.zip › Supplementary Table S2.pdf]

Supplementary Table S2 Error segment lengths and gaps summary table for FT3

Data	Error type	Gap -1	Segment 1 count	Gap 1-2	Segment 2 count	Gap 2-3	Segment 3 count	Gap 3-4	Segment 4 count	Gap 4-5	Segment 5 count	Gap 5-
Training Set	error_decrease_10	86	286	283	168	400	262	334	171	403	125	982
Training Set	error_increase_10	86	286	283	168	400	262	334	171	403	125	982
Training Set	error_decrease_30	57	235	359	177	382	189	396	124	472	251	858
Training Set	error_increase_30	57	235	359	177	382	189	396	124	472	251	858
Training Set	error_decrease_50	56	100	498	236	320	162	408	139	425	124	1032
Training Set	error_increase_50	56	100	498	236	320	162	408	139	425	124	1032
Training Set	error_decrease_70	43	124	432	288	297	279	298	167	420	297	855
Training Set	error_increase_70	43	124	432	288	297	279	298	167	420	297	855
Training Set	error_decrease_90	38	163	391	140	446	191	364	205	380	285	897
Training Set	error_increase_90	38	163	391	140	446	191	364	205	380	285	897
Test Set	error_decrease_10	86	286	283	168	400	262	334	171	403	125	982
Test Set	error_increase_1	86	286	283	168	400	262	334	171	403	125	982

	0											
Test Set	error_decrease_30	57	235	359	177	382	189	396	124	472	251	858
Test Set	error_increase_30	57	235	359	177	382	189	396	124	472	251	858
Test Set	error_decrease_50	56	100	498	236	320	162	408	139	425	124	1032
Test Set	error_increase_50	56	100	498	236	320	162	408	139	425	124	1032
Test Set	error_decrease_70	43	124	432	288	297	279	298	167	420	297	855
Test Set	error_increase_70	43	124	432	288	297	279	298	167	420	297	855
Test Set	error_decrease_90	38	163	391	140	446	191	364	205	380	285	897
Test Set	error_increase_90	38	163	391	140	446	191	364	205	380	285	897
