# Supplementary material for: Comparison of EWMA, MA, and MQ Under a Unified PBRTQC Framework for Thyroid and Coagulation Tests
Source: Diagnostics (Basel). 2026 Jan 16;16(2):288. doi: 10.3390/diagnostics16020288 (PMC12839619; doi:10.3390/diagnostics16020288)
Supplement: Supplementary file 1 [file diagnostics-16-00288-s001.zip › Supplementary Table S3.pdf]

Supplementary Table S3 Error segment lengths and gaps summary table for FT4

Data	Error type	Gap -1	Segment 1 count	Gap 1-2	Segment 2 count	Gap 2-3	Segment 3 count	Gap 3-4	Segment 4 count	Gap 4-5	Segment 5 count	Gap 5-
Training Set	error_decrease _10	87	221	346	279	293	108	465	109	462	268	862
Training Set	error_increase _10	87	221	346	279	293	108	465	109	462	268	862
Training Set	error_decrease _30	58	106	476	204	369	207	388	240	335	243	874
Training Set	error_increase _30	58	106	476	204	369	207	388	240	335	243	874
Training Set	error_decrease _50	45	204	354	142	408	108	446	291	280	296	926
Training Set	error_increase _50	45	204	354	142	408	108	446	291	280	296	926
Training Set	error_decrease _70	76	136	427	274	325	100	488	178	409	192	895
Training Set	error_increase _70	76	136	427	274	325	100	488	178	409	192	895
Training Set	error_decrease _90	94	231	357	239	356	221	351	186	391	129	945
Training Set	error_increase _90	94	231	357	239	356	221	351	186	391	129	945
Test Set	error_decrease _10	87	221	346	279	293	108	465	109	462	268	862
Test Set	error_increase	87	221	346	279	293	108	465	109	462	268	862

	_10											
Test Set	error_decrease_30	58	106	476	204	369	207	388	240	335	243	874
Test Set	error_increase_30	58	106	476	204	369	207	388	240	335	243	874
Test Set	error_decrease_50	45	204	354	142	408	108	446	291	280	296	926
Test Set	error_increase_50	45	204	354	142	408	108	446	291	280	296	926
Test Set	error_decrease_70	76	136	427	274	325	100	488	178	409	192	895
Test Set	error_increase_70	76	136	427	274	325	100	488	178	409	192	895
Test Set	error_decrease_90	94	231	357	239	356	221	351	186	391	129	945
Test Set	error_increase_90	94	231	357	239	356	221	351	186	391	129	945
