# Supplementary material for: Comparison of EWMA, MA, and MQ Under a Unified PBRTQC Framework for Thyroid and Coagulation Tests
Source: Diagnostics (Basel). 2026 Jan 16;16(2):288. doi: 10.3390/diagnostics16020288 (PMC12839619; doi:10.3390/diagnostics16020288)
Supplement: Supplementary file 1 [file diagnostics-16-00288-s001.zip › Supplementary Table S4.pdf]

Supplementary Table S4 Error segment lengths and gaps summary table for PT

Data	Error type	Gap -1	Segment 1 count	Gap 1-2	Segment 2 count	Gap 2-3	Segment 3 count	Gap 3-4	Segment 4 count	Gap 4-5	Segment 5 count	Gap 5-
Training Set	error_decrease _10	22	233	357	166	385	143	445	202	349	139	1059
Training Set	error_increase _10	22	233	357	166	385	143	445	202	349	139	1059
Training Set	error_decrease _30	71	258	324	169	403	192	368	177	398	233	907
Training Set	error_increase _30	71	258	324	169	403	192	368	177	398	233	907
Training Set	error_decrease _50	65	169	402	134	450	212	343	223	342	216	944
Training Set	error_increase _50	65	169	402	134	450	212	343	223	342	216	944
Training Set	error_decrease _70	34	176	383	134	431	108	445	152	446	259	932
Training Set	error_increase _70	34	176	383	134	431	108	445	152	446	259	932
Training Set	error_decrease _90	49	107	473	271	319	129	439	284	277	285	867
Training Set	error_increase _90	49	107	473	271	319	129	439	284	277	285	867
Test Set	error_decrease _10	22	233	357	166	385	143	445	202	349	139	1059
Test Set	error_increase	22	233	357	166	385	143	445	202	349	139	1059

	_10											
Test Set	error_decrease_30	71	258	324	169	403	192	368	177	398	233	907
Test Set	error_increase_30	71	258	324	169	403	192	368	177	398	233	907
Test Set	error_decrease_50	65	169	402	134	450	212	343	223	342	216	944
Test Set	error_increase_50	65	169	402	134	450	212	343	223	342	216	944
Test Set	error_decrease_70	34	176	383	134	431	108	445	152	446	259	932
Test Set	error_increase_70	34	176	383	134	431	108	445	152	446	259	932
Test Set	error_decrease_90	49	107	473	271	319	129	439	284	277	285	867
Test Set	error_increase_90	49	107	473	271	319	129	439	284	277	285	867
