# Supplementary material for: Comparison of EWMA, MA, and MQ Under a Unified PBRTQC Framework for Thyroid and Coagulation Tests
Source: Diagnostics (Basel). 2026 Jan 16;16(2):288. doi: 10.3390/diagnostics16020288 (PMC12839619; doi:10.3390/diagnostics16020288)
Supplement: Supplementary file 1 [file diagnostics-16-00288-s001.zip › Supplementary Table S5.pdf]

Supplementary Table S5 Error segment lengths and gaps summary table for APTT

Data	Error type	Gap -1	Segment 1 count	Gap 1-2	Segment 2 count	Gap 2-3	Segment 3 count	Gap 3-4	Segment 4 count	Gap 4-5	Segment 5 count	Gap 5-
Training Set	error_decrease_10	14	269	315	155	397	187	379	192	360	222	1010
Training Set	error_increase_10	14	269	315	155	397	187	379	192	360	222	1010
Training Set	error_decrease_30	79	145	435	206	388	223	356	171	427	259	811
Training Set	error_increase_30	79	145	435	206	388	223	356	171	427	259	811
Training Set	error_decrease_50	18	230	370	190	366	283	310	109	487	176	961
Training Set	error_increase_50	18	230	370	190	366	283	310	109	487	176	961
Training Set	error_decrease_70	98	206	368	256	295	165	391	281	292	194	954
Training Set	error_increase_70	98	206	368	256	295	165	391	281	292	194	954
Training Set	error_decrease_90	93	131	425	265	294	161	437	239	337	129	989
Training Set	error_increase_90	93	131	425	265	294	161	437	239	337	129	989
Test Set	error_decrease_10	14	269	315	155	397	187	379	192	360	222	1010
Test Set	error_increase_1	14	269	315	155	397	187	379	192	360	222	1010

	0											
Test Set	error_decrease_30	79	145	435	206	388	223	356	171	427	259	811
Test Set	error_increase_30	79	145	435	206	388	223	356	171	427	259	811
Test Set	error_decrease_50	18	230	370	190	366	283	310	109	487	176	961
Test Set	error_increase_50	18	230	370	190	366	283	310	109	487	176	961
Test Set	error_decrease_70	98	206	368	256	295	165	391	281	292	194	954
Test Set	error_increase_70	98	206	368	256	295	165	391	281	292	194	954
Test Set	error_decrease_90	93	131	425	265	294	161	437	239	337	129	989
Test Set	error_increase_90	93	131	425	265	294	161	437	239	337	129	989
