# Supplementary material for: Comparison of EWMA, MA, and MQ Under a Unified PBRTQC Framework for Thyroid and Coagulation Tests
Source: Diagnostics (Basel). 2026 Jan 16;16(2):288. doi: 10.3390/diagnostics16020288 (PMC12839619; doi:10.3390/diagnostics16020288)
Supplement: Supplementary file 1 [file diagnostics-16-00288-s001.zip › Supplementary Table S6.pdf]

Supplementary Table S6 Error segment lengths and gaps summary table for TT

Data	Error type	Gap -1	Segment 1 count	Gap 1-2	Segment 2 count	Gap 2-3	Segment 3 count	Gap 3-4	Segment 4 count	Gap 4-5	Segment 5 count	Gap 5-
Training Set	error_decrease_10	96	256	309	295	283	259	339	113	440	223	887
Training Set	error_increase_10	96	256	309	295	283	259	339	113	440	223	887
Training Set	error_decrease_30	70	228	370	252	326	236	326	270	318	279	825
Training Set	error_increase_30	70	228	370	252	326	236	326	270	318	279	825
Training Set	error_decrease_50	70	111	486	235	362	277	319	110	457	188	885
Training Set	error_increase_50	70	111	486	235	362	277	319	110	457	188	885
Training Set	error_decrease_70	68	149	415	259	322	129	469	233	367	178	911
Training Set	error_increase_70	68	149	415	259	322	129	469	233	367	178	911
Training Set	error_decrease_90	91	213	380	149	408	123	456	210	341	220	909
Training Set	error_increase_90	91	213	380	149	408	123	456	210	341	220	909
Test Set	error_decrease_10	96	256	309	295	283	259	339	113	440	223	887
Test Set	error_increase_10	96	256	309	295	283	259	339	113	440	223	887
Test Set	error_decrease_30	70	228	370	252	326	236	326	270	318	279	825

Test Set	error_increase_30	70	228	370	252	326	236	326	270	318	279	825
Test Set	error_decrease_50	70	111	486	235	362	277	319	110	457	188	885
Test Set	error_increase_50	70	111	486	235	362	277	319	110	457	188	885
Test Set	error_decrease_70	68	149	415	259	322	129	469	233	367	178	911
Test Set	error_increase_70	68	149	415	259	322	129	469	233	367	178	911
Test Set	error_decrease_90	91	213	380	149	408	123	456	210	341	220	909
Test Set	error_increase_90	91	213	380	149	408	123	456	210	341	220	909
