# Supplementary material for: Comparison of EWMA, MA, and MQ Under a Unified PBRTQC Framework for Thyroid and Coagulation Tests
Source: Diagnostics (Basel). 2026 Jan 16;16(2):288. doi: 10.3390/diagnostics16020288 (PMC12839619; doi:10.3390/diagnostics16020288)
Supplement: Supplementary file 1 [file diagnostics-16-00288-s001.zip › Supplementary Table S7.pdf]

Supplementary Table S7. Changes in distributional statistics before and after Box–Cox transformation

Analytes	stage	mean	sd	skewness	kurtosis
TSH	Before_BoxCox	3.236	10.15	10.917	136.199
	After_BoxCox	3.624	1.427	0.467	8.389
FT3	Before_BoxCox	3.43	1.241	8.947	108.502
	After_BoxCox	3.015	0.164	-0.359	26.232
FT4	Before_BoxCox	1.321	0.456	9.978	172.44
	After_BoxCox	2.577	0.238	0.002	18.986
PT	Before_BoxCox	12.532	2.795	8.626	163.286
	After_BoxCox	0.918	0.01	1.768	8.449
APTT	Before_BoxCox	28.489	8.268	7.762	95.34
	After_BoxCox	0.964	0.006	1.271	6.747
TT	Before_BoxCox	17.947	6.534	12.227	179.889
	After_BoxCox	0.942	0.006	3.281	22.699
