# Supplementary material for: Comparison of EWMA, MA, and MQ Under a Unified PBRTQC Framework for Thyroid and Coagulation Tests
Source: Diagnostics (Basel). 2026 Jan 16;16(2):288. doi: 10.3390/diagnostics16020288 (PMC12839619; doi:10.3390/diagnostics16020288)
Supplement: Supplementary file 1 [file diagnostics-16-00288-s001.zip › Supplementary Table S8.pdf]

Supplementary Table S8. Recommended PBRTQC parameters and average performance for the moving average algorithm across training and test datasets

Analytes	Window width	Upper limit multiplier (a)	Lower limit multiplier (b)	Truncation factor	Consecutive alarm points	Data	ME_Score	Sensitivity	False positive rate	MNPed
TSH	3	3	3	0.05	5	Training set	0.9984	0.5561	0.0007	42
						Test set	0.9984	0.5573	0.0008	34
FT3	3	1.64	1.64	0.1	10	Training set	0.9975	0.9997	0.0020	0
						Test set	0.9975	0.9994	0.0020	0
FT4	3	3	3	0.05	5	Training set	0.9978	0.8660	0.0016	3
						Test set	0.9977	0.8631	0.0017	7
PT	3	1.64	1.64	0	10	Training set	0.9975	1.0000	0.0020	0
						Test set	0.9968	1.0000	0.0027	0
APTT	3	1.96	1.64	0	10	Training set	0.9975	1.0000	0.0020	0
						Test set	0.9975	1.0000	0.0020	0
TT	3	1.64	1.96	0	10	Training set	0.9975	1.0000	0.0020	0
						Test set	0.9975	1.0000	0.0020	0
