# Supplementary material for: Comparison of EWMA, MA, and MQ Under a Unified PBRTQC Framework for Thyroid and Coagulation Tests
Source: Diagnostics (Basel). 2026 Jan 16;16(2):288. doi: 10.3390/diagnostics16020288 (PMC12839619; doi:10.3390/diagnostics16020288)
Supplement: Supplementary file 1 [file diagnostics-16-00288-s001.zip › Supplementary Table S9.pdf]

Supplementary Table S9. Recommended PBRTQC parameters and performance metrics for the moving quantile algorithm

Analytes	Window width	Quantile Level	Upper limit multiplier (a)	Lower limit multiplier (b)	Truncation factor	Consecutive alarm points	Data	ME_Score	Sensitivity	False positive rate	MNPed
TSH	3	0.9	3	3	0	5	Training set	0.9987	0.5548	0.0004	46
							Test set	0.9986	0.5537	0.0005	38
FT3	3	0.1	3	3	0	5	Training set	0.9977	0.9906	0.0018	1
							Test set	0.9977	0.9890	0.0018	1
FT4	3	0.9	1.96	3	0	5	Training set	0.9981	0.8670	0.0013	5
							Test set	0.998	0.8630	0.0014	3
PT	3	0.9	1.64	3	0	10	Training set	0.9977	0.9969	0.0018	1
							Test set	0.9976	0.9967	0.0019	1
APTT	3	0.6	3	1.64	0	10	Training set	0.9975	0.9980	0.0020	1
							Test set	0.9975	0.9976	0.0020	1
TT	3	0.6	1.64	1.96	0	10	Training set	0.9975	0.9981	0.0020	1
							Test set	0.9975	0.9980	0.0020	0
