# Supplementary material for: Comparison of EWMA, MA, and MQ Under a Unified PBRTQC Framework for Thyroid and Coagulation Tests
Source: Diagnostics (Basel). 2026 Jan 16;16(2):288. doi: 10.3390/diagnostics16020288 (PMC12839619; doi:10.3390/diagnostics16020288)

TSH_outputMQ - Training (p1-p10)

Moving Quantile Control Chart p1 - Training Set: error_decrease_10
(Window= 3 , QL= 0.9 , a= 3 , b= 3 , TL= 0 , CP= 5)

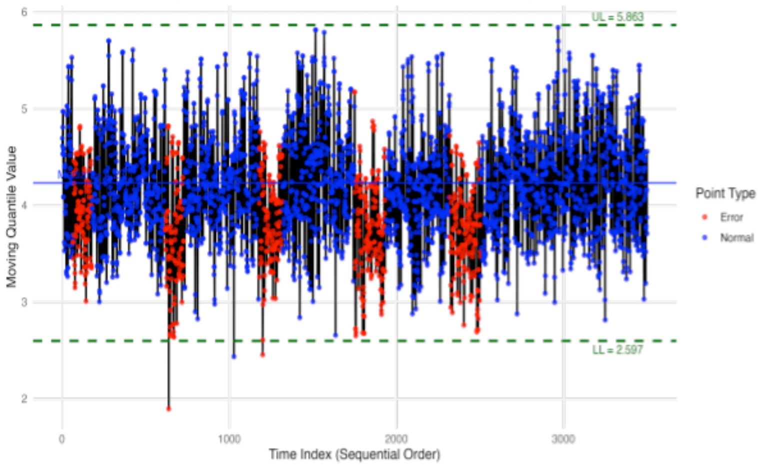

Moving Quantile Control Chart p2 - Training Set: error_decrease_30
(Window= 3 , QL= 0.9 , a= 3 , b= 3 , TL= 0 , CP= 5)

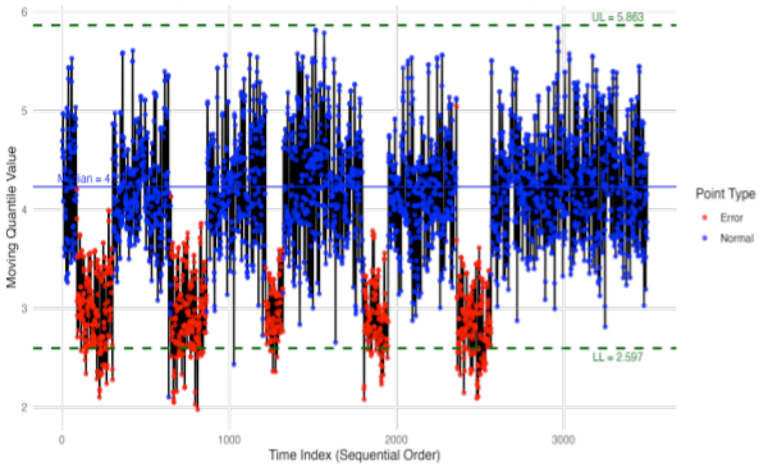

Moving Quantile Control Chart p3 - Training Set: error_decrease_50
(Window= 3 , QL= 0.9 , a= 3 , b= 3 , TL= 0 , CP= 5)

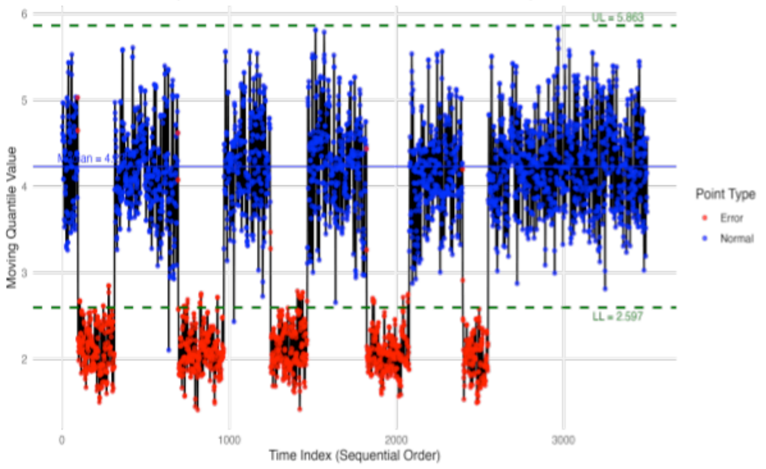

Moving Quantile Control Chart p4 - Training Set: error_decrease_70
(Window= 3 , QL= 0.9 , a= 3 , b= 3 , TL= 0 , CP= 5)

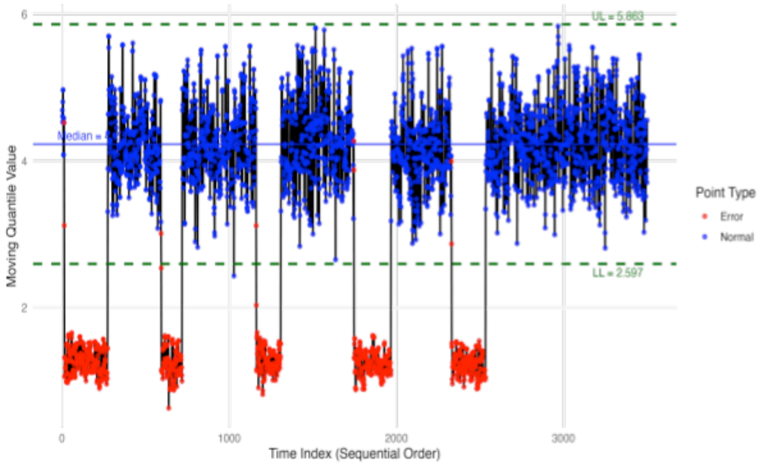

Moving Quantile Control Chart p5 - Training Set: error_decrease_90
(Window= 3 , QL= 0.9 , a= 3 , b= 3 , TL= 0 , CP= 5)

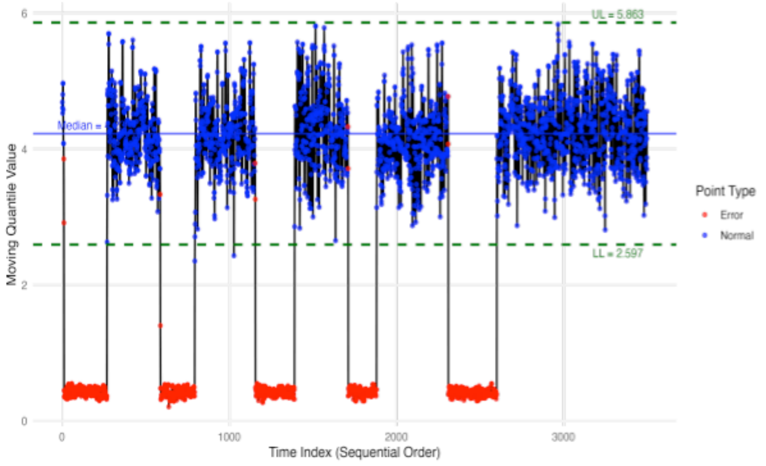

Moving Quantile Control Chart p6 - Training Set: error_increase_10
(Window= 3 , QL= 0.9 , a= 3 , b= 3 , TL= 0 , CP= 5)

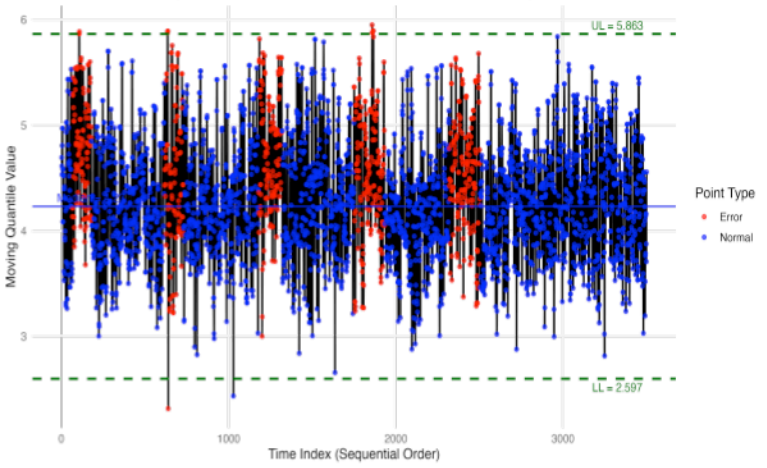

Moving Quantile Control Chart p7 - Training Set: error_increase_30
(Window= 3 , QL= 0.9 , a= 3 , b= 3 , TL= 0 , CP= 5)

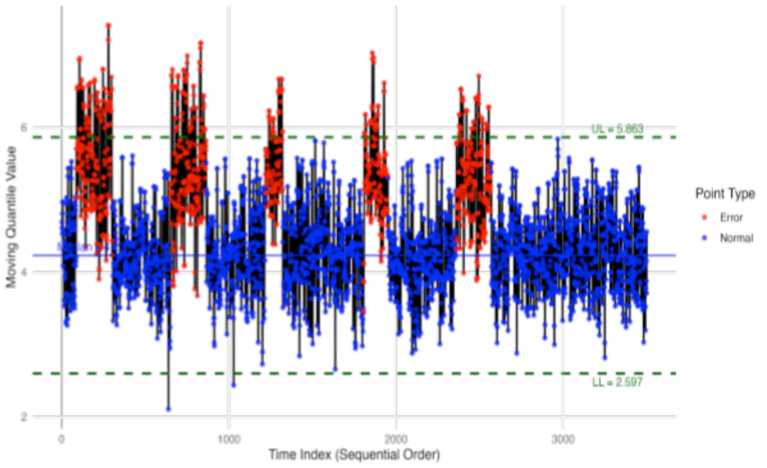

Moving Quantile Control Chart p8 - Training Set: error_increase_50
(Window= 3 , QL= 0.9 , a= 3 , b= 3 , TL= 0 , CP= 5)

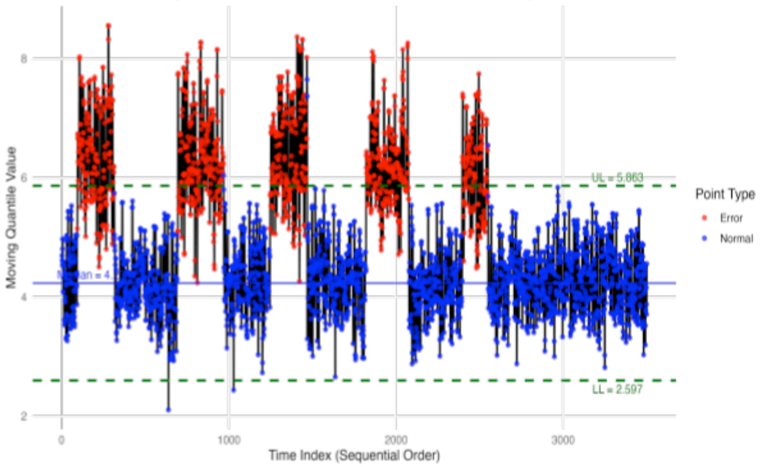

Moving Quantile Control Chart p9 - Training Set: error_increase_70
(Window= 3 , QL= 0.9 , a= 3 , b= 3 , TL= 0 , CP= 5)

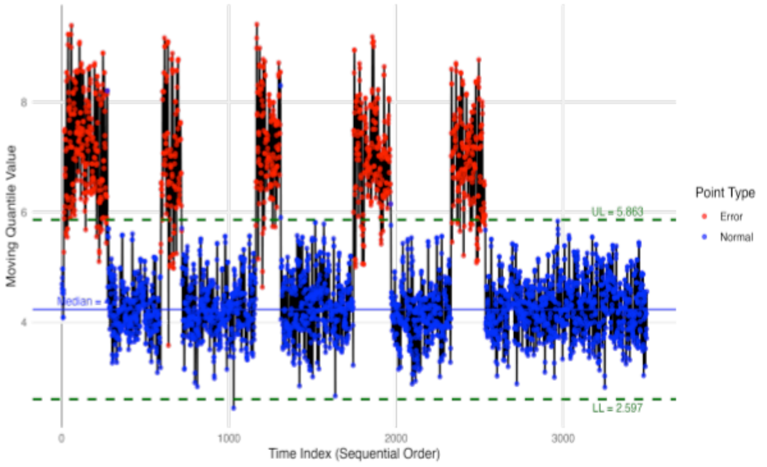

Moving Quantile Control Chart p10 - Training Set: error_increase_90
(Window= 3 , QL= 0.9 , a= 3 , b= 3 , TL= 0 , CP= 5)

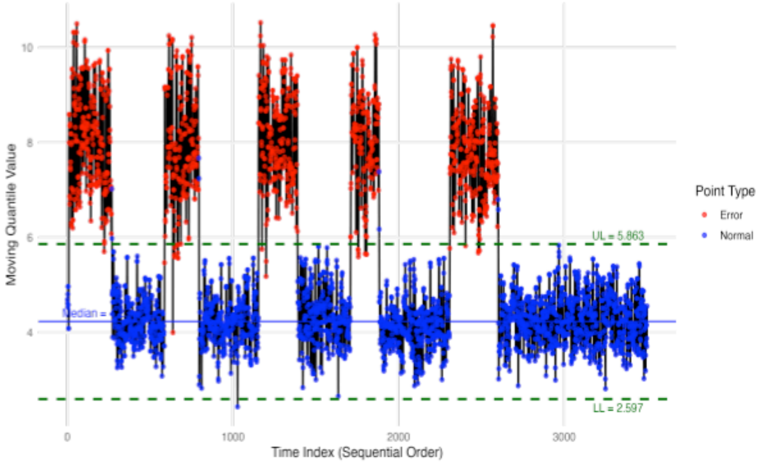

TSH_outputMQ - Test (p11-p20)

Moving Quantile Control Chart p11 - Test Set: error_decrease_10
(Window= 3 , QL= 0.9 , a= 3 , b= 3 , TL= 0 , CP= 5)

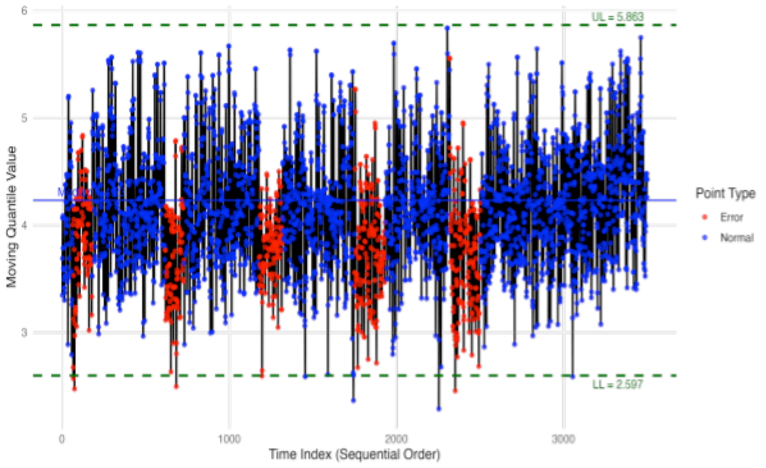

Moving Quantile Control Chart p12 - Test Set: error_decrease_30
(Window= 3 , QL= 0.9 , a= 3 , b= 3 , TL= 0 , CP= 5)

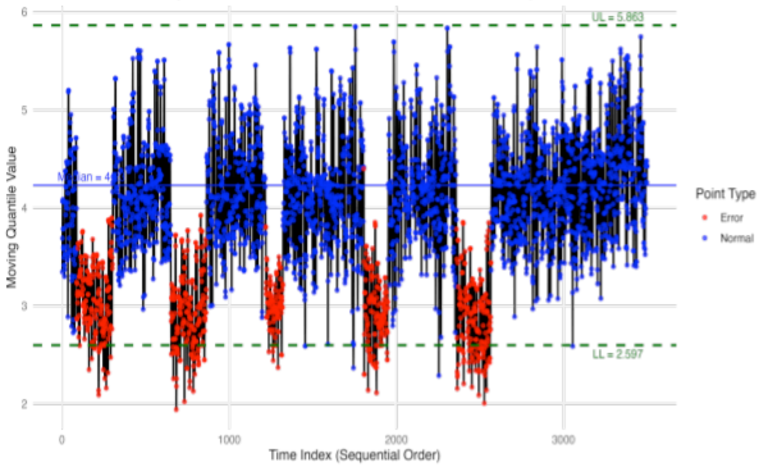

Moving Quantile Control Chart p13 - Test Set: error_decrease_50
(Window= 3 , QL= 0.9 , a= 3 , b= 3 , TL= 0 , CP= 5)

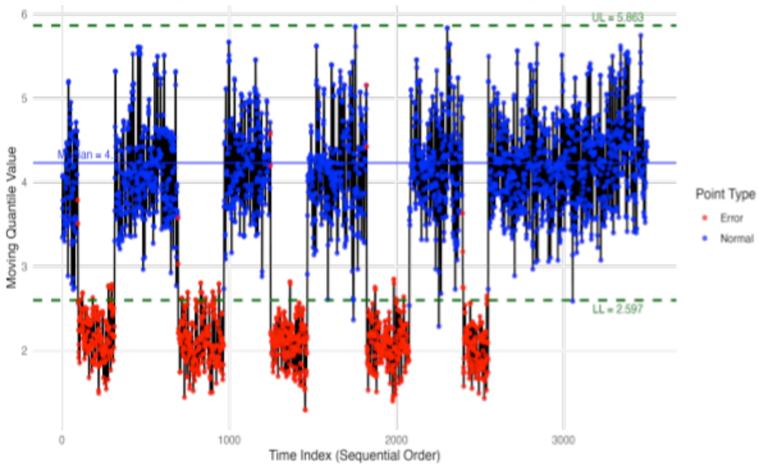

Moving Quantile Control Chart p14 - Test Set: error_decrease_70
(Window= 3 , QL= 0.9 , a= 3 , b= 3 , TL= 0 , CP= 5)

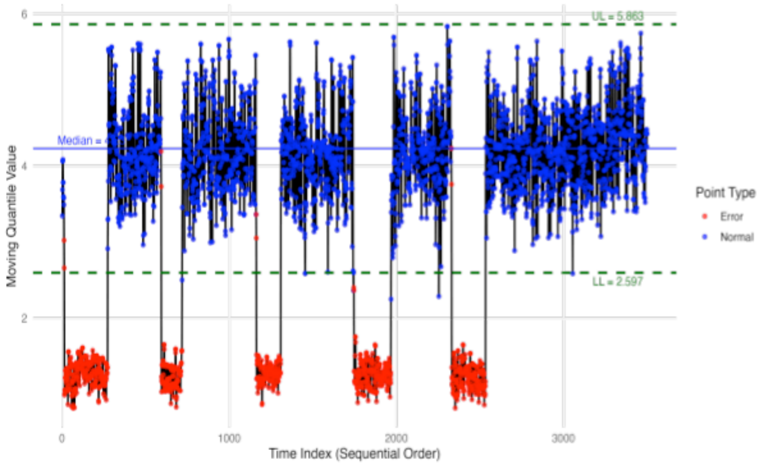

Moving Quantile Control Chart p15 - Test Set: error_decrease_90
(Window= 3 , QL= 0.9 , a= 3 , b= 3 , TL= 0 , CP= 5)

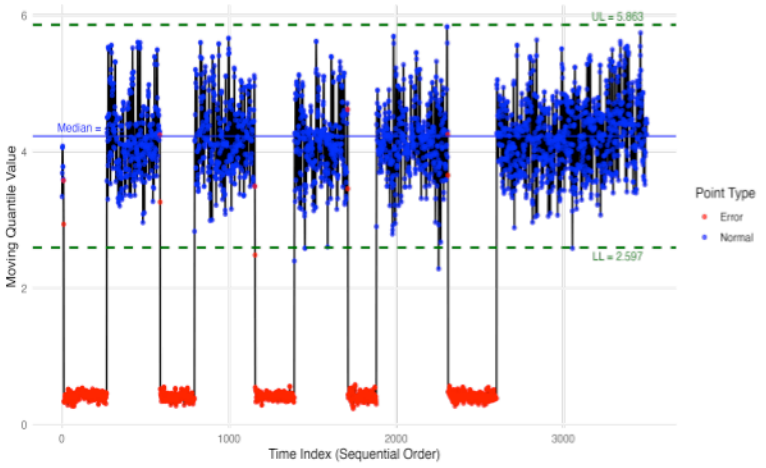

Moving Quantile Control Chart p16 - Test Set: error_increase_10
(Window= 3 , QL= 0.9 , a= 3 , b= 3 , TL= 0 , CP= 5)

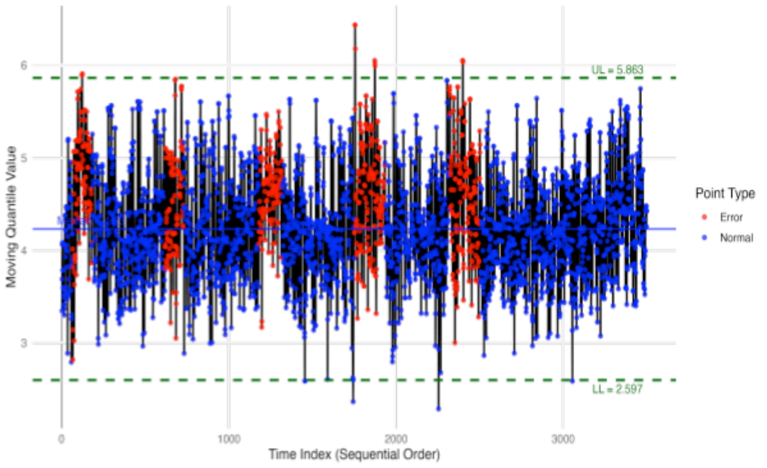

Moving Quantile Control Chart p17 - Test Set: error_increase_30
(Window= 3 , QL= 0.9 , a= 3 , b= 3 , TL= 0 , CP= 5)

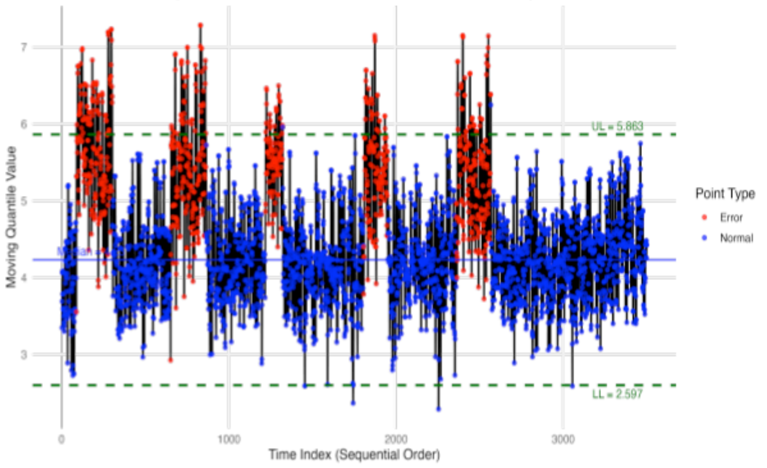

Moving Quantile Control Chart p18 - Test Set: error_increase_50
(Window= 3 , QL= 0.9 , a= 3 , b= 3 , TL= 0 , CP= 5)

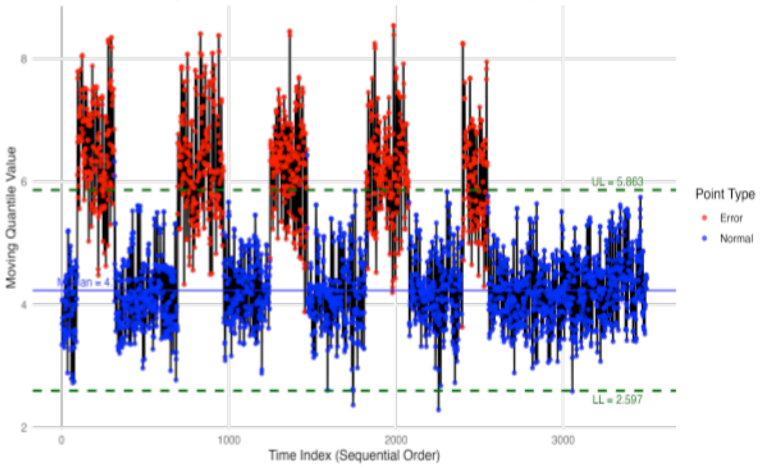

Moving Quantile Control Chart p19 - Test Set: error_increase_70
(Window= 3 , QL= 0.9 , a= 3 , b= 3 , TL= 0 , CP= 5)

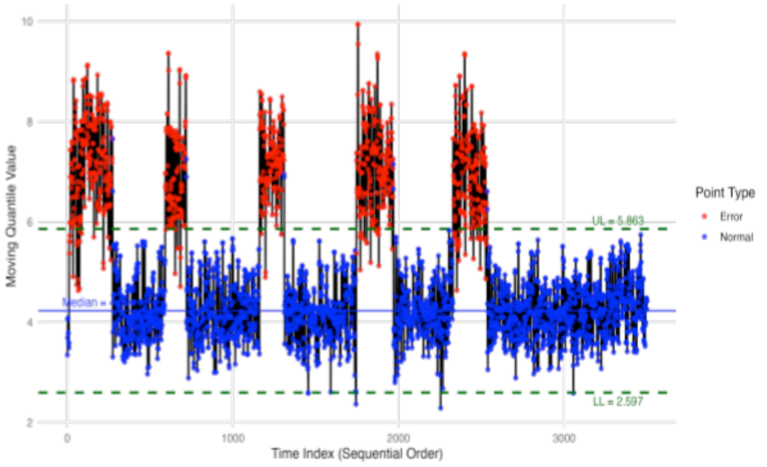

Moving Quantile Control Chart p20 - Test Set: error_increase_90
(Window= 3 , QL= 0.9 , a= 3 , b= 3 , TL= 0 , CP= 5)

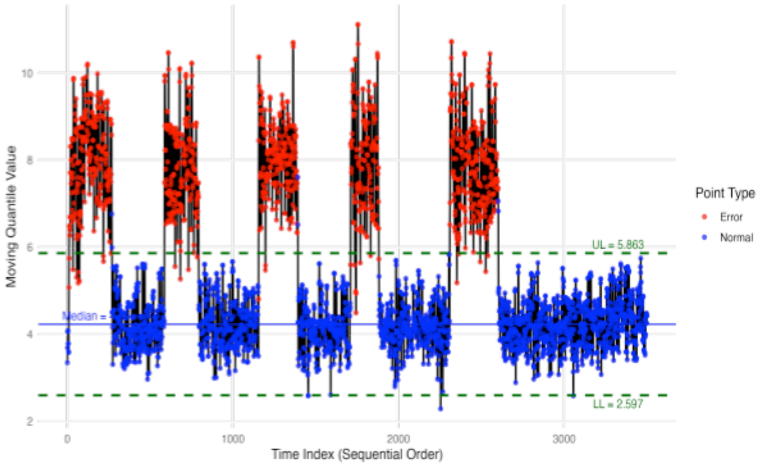

TSH_outputEWMA - Test (p11-p20)

EWMA Control Chart p11 - Test Set: error_decrease_10
(λ= 0.4 , a= 1.64 , b= 1.96 , TL= 0 , CP= 5)

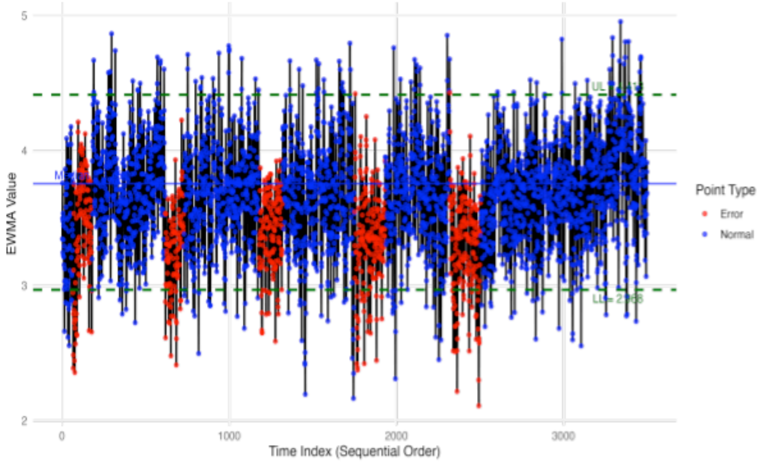

EWMA Control Chart p16 - Test Set: error_increase_10
(λ= 0.4 , a= 1.64 , b= 1.96 , TL= 0 , CP= 5)

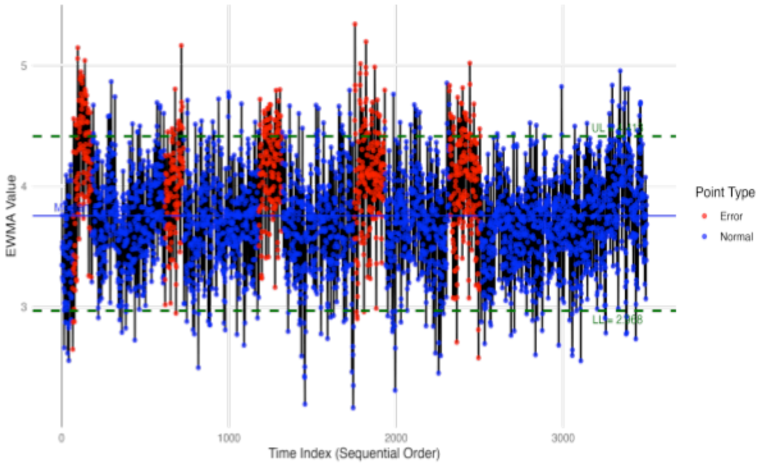

EWMA Control Chart p12 - Test Set: error_decrease_30
(λ= 0.4 , a= 1.64 , b= 1.96 , TL= 0 , CP= 5)

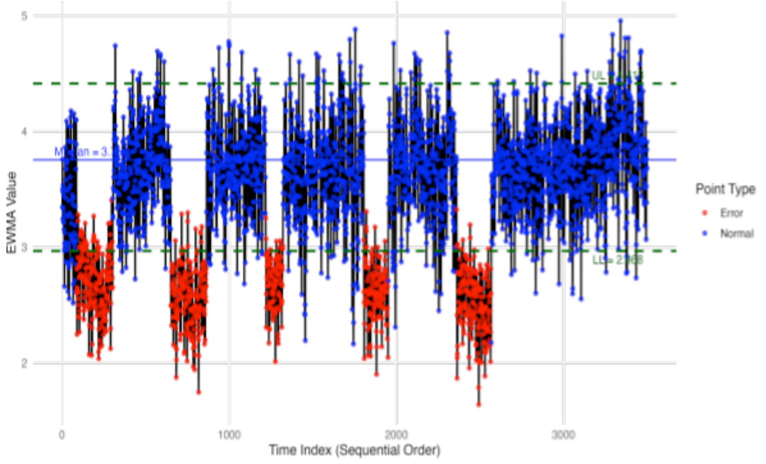

EWMA Control Chart p17 - Test Set: error_increase_30
(λ= 0.4 , a= 1.64 , b= 1.96 , TL= 0 , CP= 5)

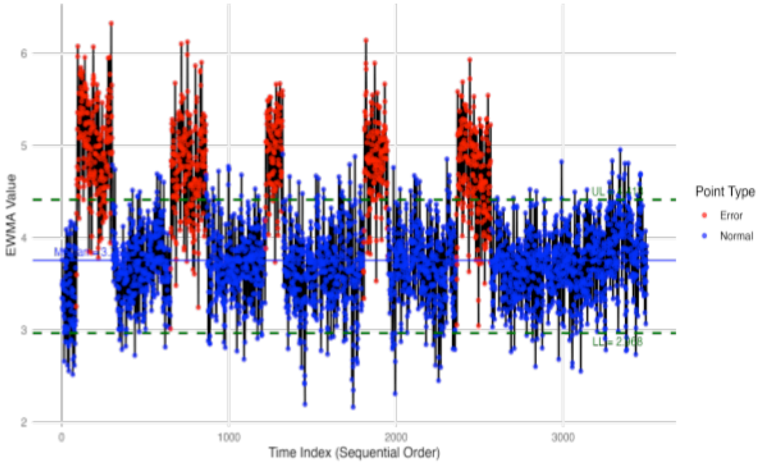

EWMA Control Chart p13 - Test Set: error_decrease_50
(λ= 0.4 , a= 1.64 , b= 1.96 , TL= 0 , CP= 5)

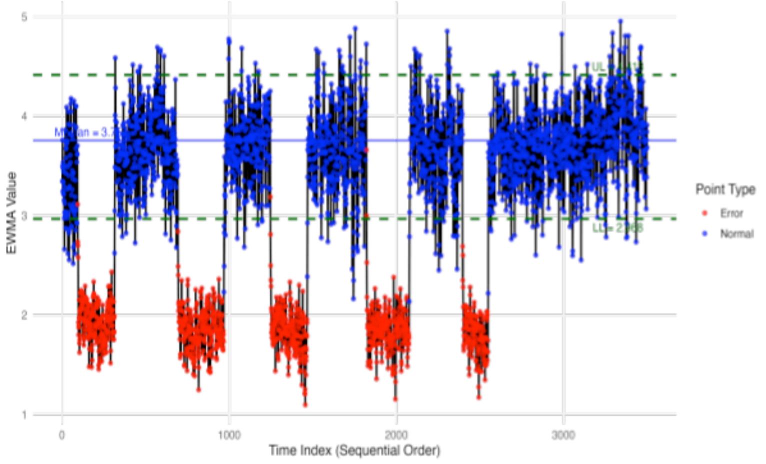

EWMA Control Chart p18 - Test Set: error_increase_50
(λ= 0.4 , a= 1.64 , b= 1.96 , TL= 0 , CP= 5)

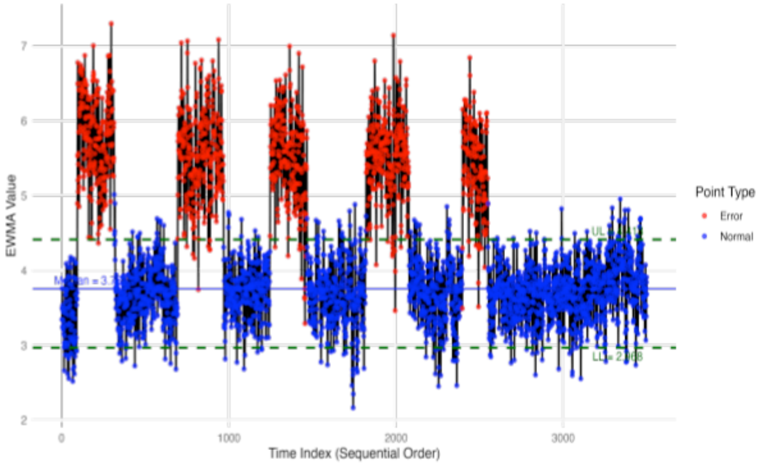

EWMA Control Chart p14 - Test Set: error_decrease_70
(λ= 0.4 , a= 1.64 , b= 1.96 , TL= 0 , CP= 5)

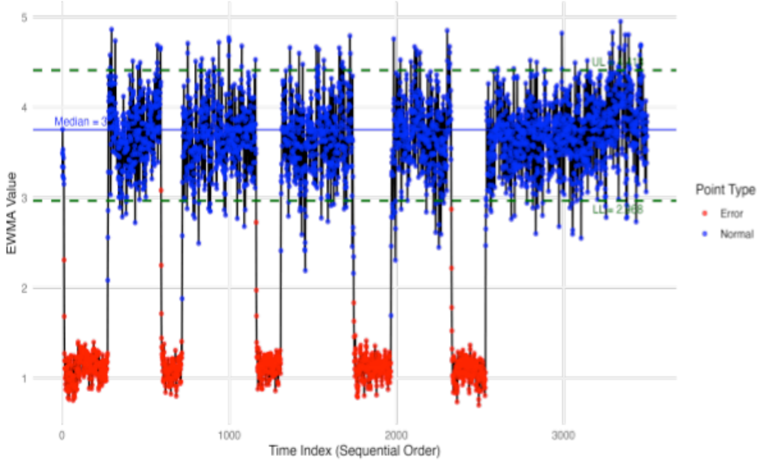

EWMA Control Chart p19 - Test Set: error_increase_70
(λ= 0.4 , a= 1.64 , b= 1.96 , TL= 0 , CP= 5)

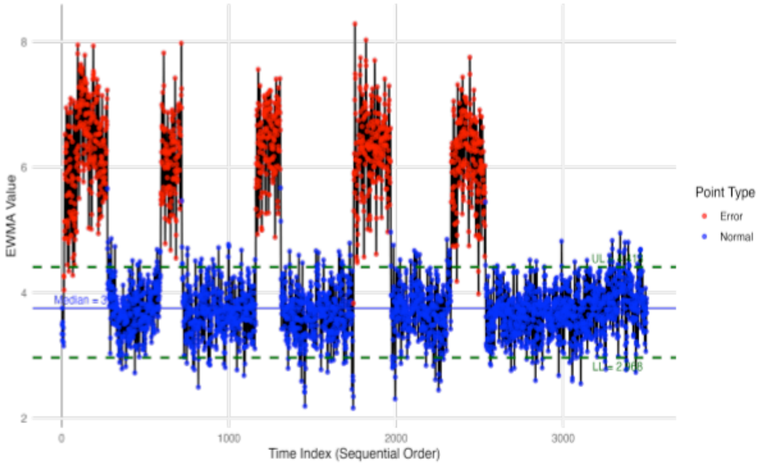

EWMA Control Chart p15 - Test Set: error_decrease_90
(λ= 0.4 , a= 1.64 , b= 1.96 , TL= 0 , CP= 5)

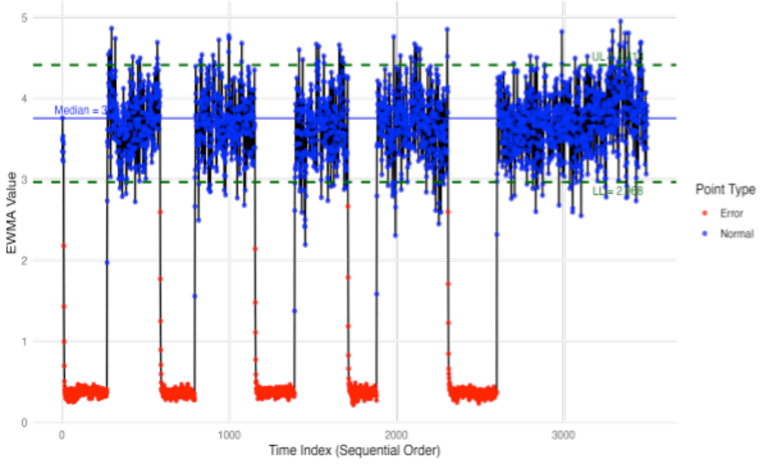

EWMA Control Chart p20 - Test Set: error_increase_90
(λ= 0.4 , a= 1.64 , b= 1.96 , TL= 0 , CP= 5)

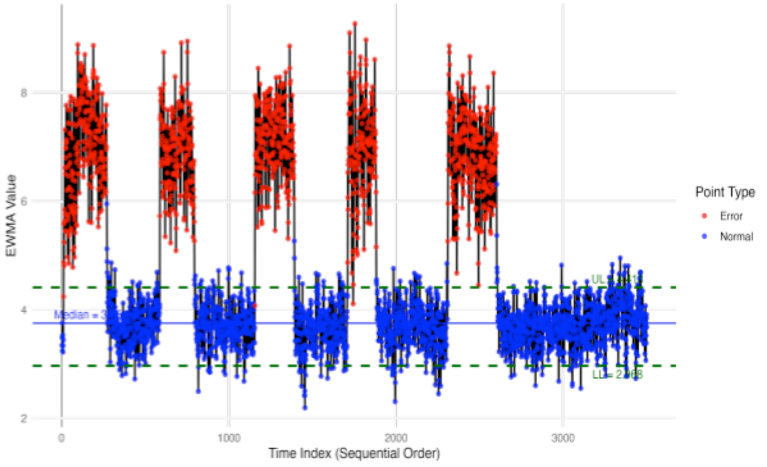

TSH_outputMA - Training (p1-p10)

Moving Average Control Chart p1 - Training Set: error_decrease_10
(Window= 3 , a= 3 , b= 3 , TL= 0.05 , CP= 5)

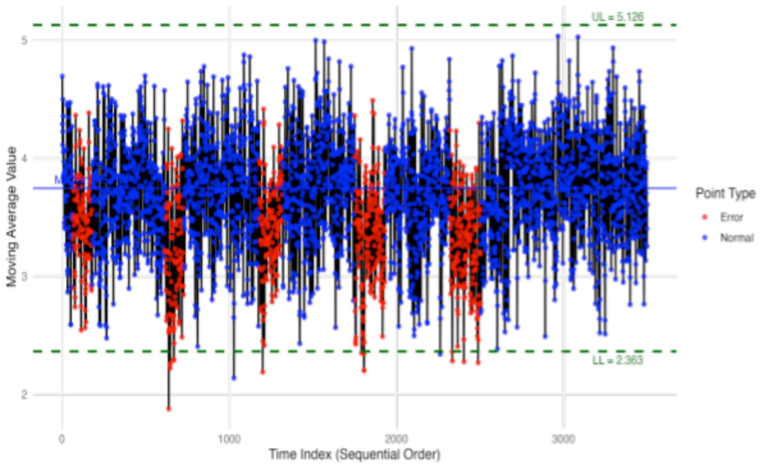

Moving Average Control Chart p2 - Training Set: error_decrease_30
(Window= 3 , a= 3 , b= 3 , TL= 0.05 , CP= 5)

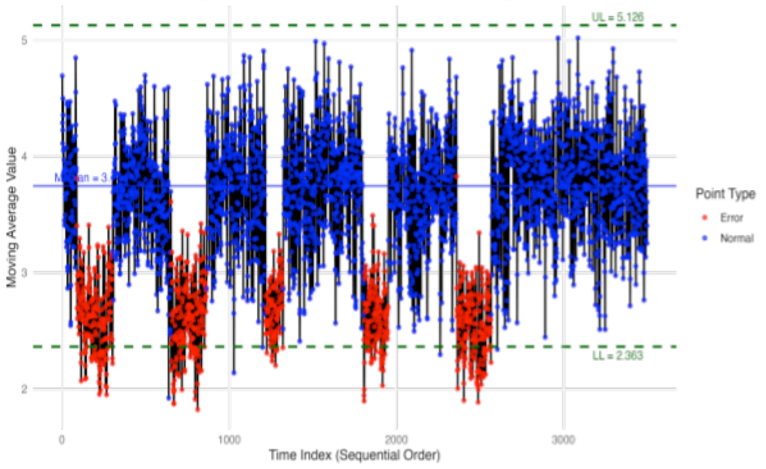

Moving Average Control Chart p3 - Training Set: error_decrease_50
(Window= 3 , a= 3 , b= 3 , TL= 0.05 , CP= 5)

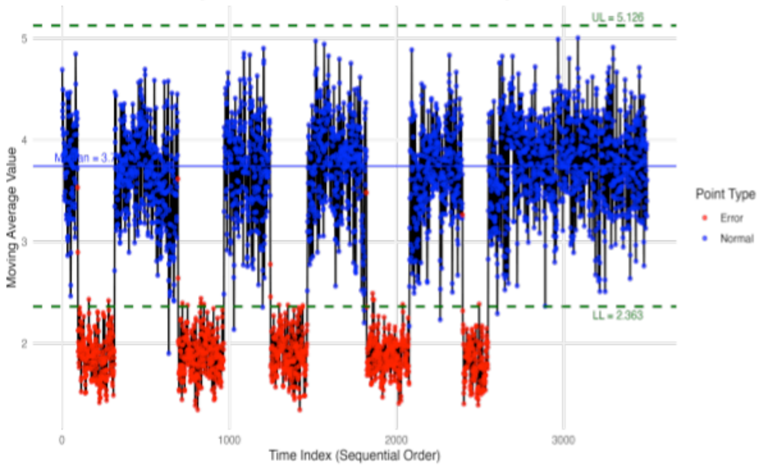

Moving Average Control Chart p4 - Training Set: error_decrease_70
(Window= 3 , a= 3 , b= 3 , TL= 0.05 , CP= 5)

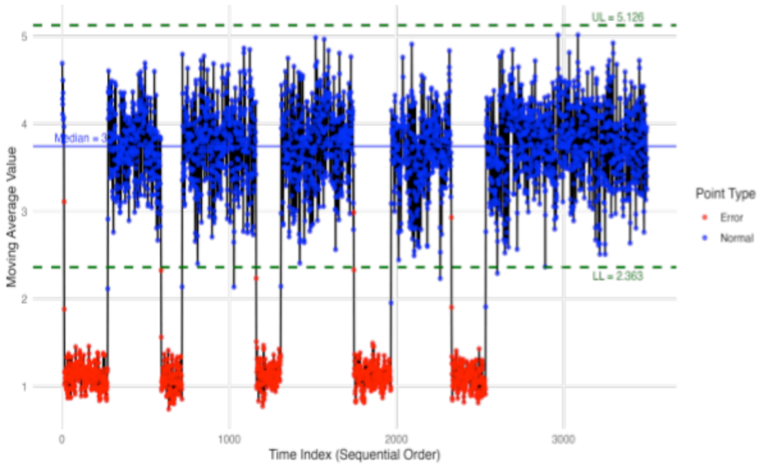

Moving Average Control Chart p5 - Training Set: error_decrease_90
(Window= 3 , a= 3 , b= 3 , TL= 0.05 , CP= 5)

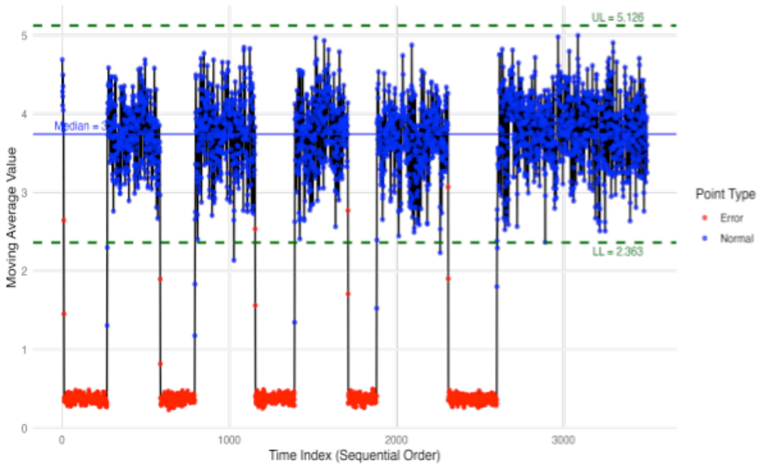

Moving Average Control Chart p6 - Training Set: error_increase_10
(Window= 3 , a= 3 , b= 3 , TL= 0.05 , CP= 5)

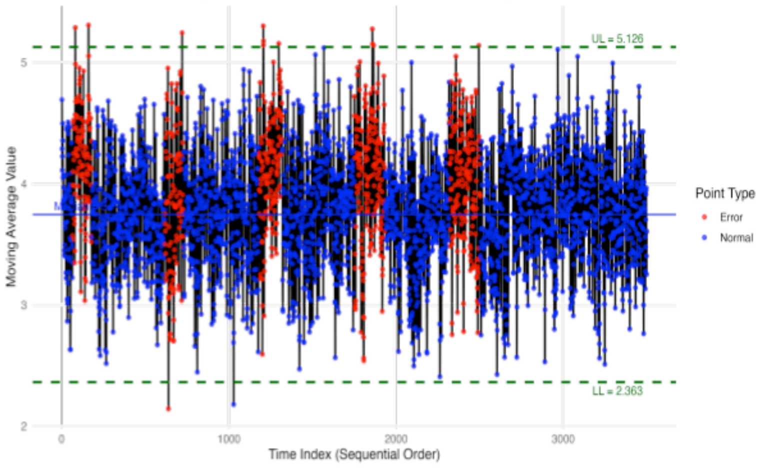

Moving Average Control Chart p7 - Training Set: error_increase_30
(Window= 3 , a= 3 , b= 3 , TL= 0.05 , CP= 5)

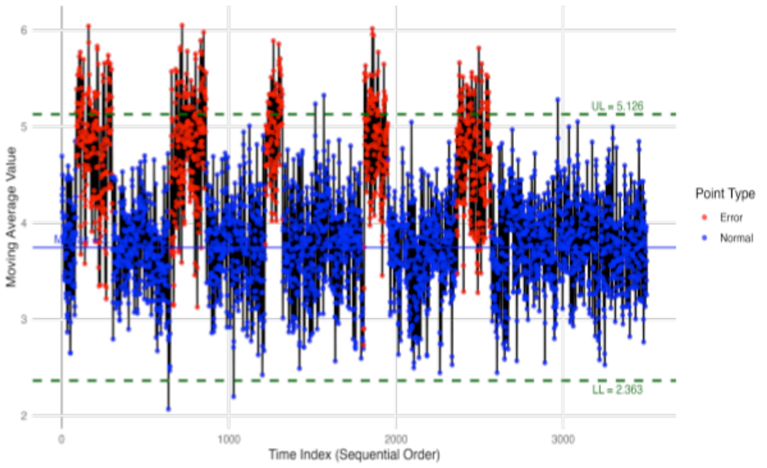

Moving Average Control Chart p8 - Training Set: error_increase_50
(Window= 3 , a= 3 , b= 3 , TL= 0.05 , CP= 5)

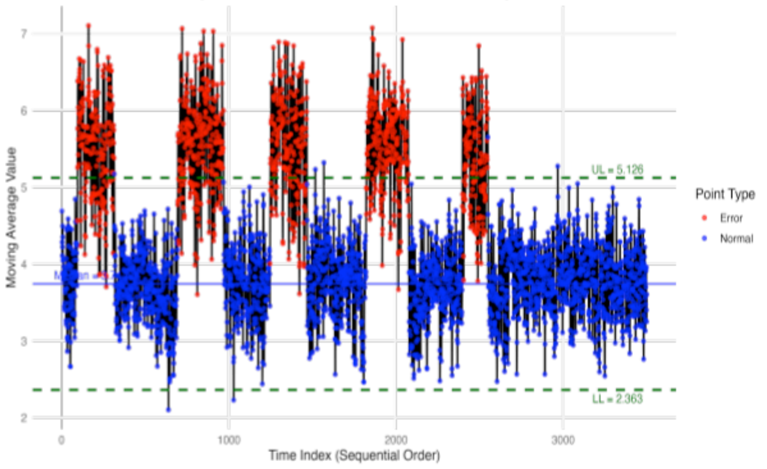

Moving Average Control Chart p9 - Training Set: error_increase_70
(Window= 3 , a= 3 , b= 3 , TL= 0.05 , CP= 5)

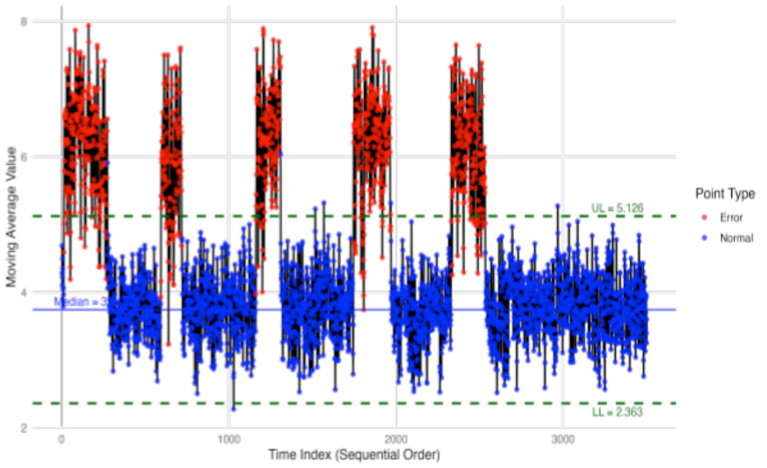

Moving Average Control Chart p10 - Training Set: error_increase_90
(Window= 3 , a= 3 , b= 3 , TL= 0.05 , CP= 5)

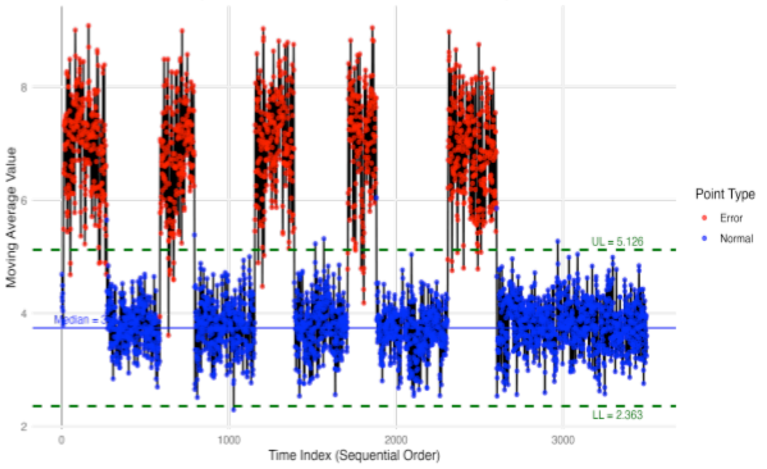

TSH_outputMA - Test (p11-p20)

Moving Average Control Chart p11 - Test Set: error_decrease_10
(Window= 3 , a= 3 , b= 3 , TL= 0.05 , CP= 5)

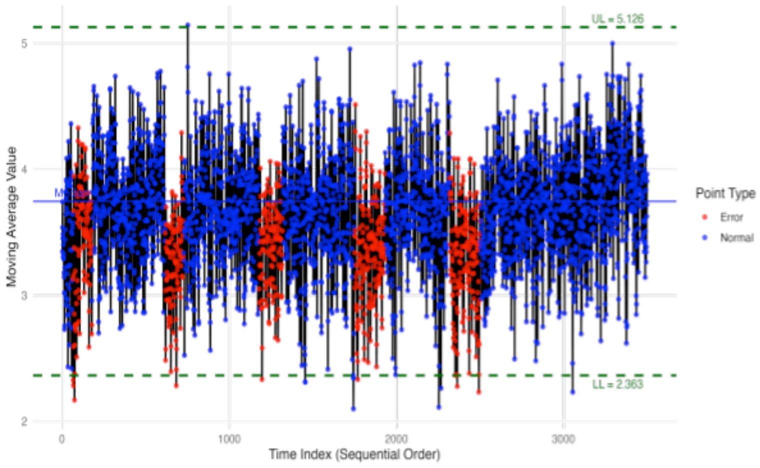

Moving Average Control Chart p12 - Test Set: error_decrease_30
(Window= 3 , a= 3 , b= 3 , TL= 0.05 , CP= 5)

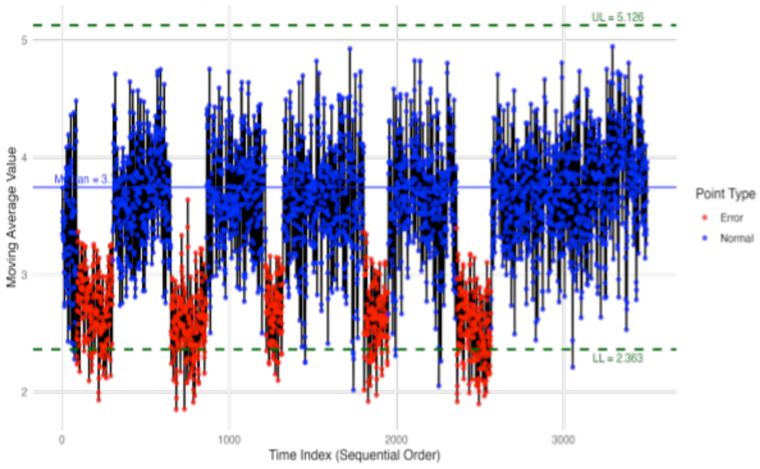

Moving Average Control Chart p13 - Test Set: error_decrease_50
(Window= 3 , a= 3 , b= 3 , TL= 0.05 , CP= 5)

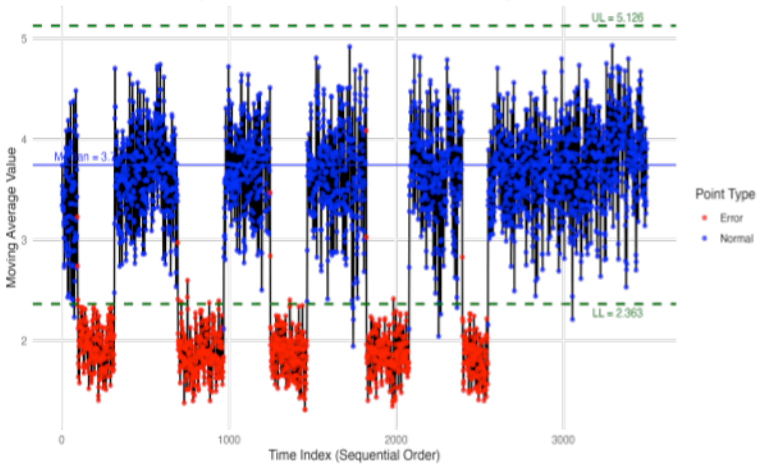

Moving Average Control Chart p14 - Test Set: error_decrease_70
(Window= 3 , a= 3 , b= 3 , TL= 0.05 , CP= 5)

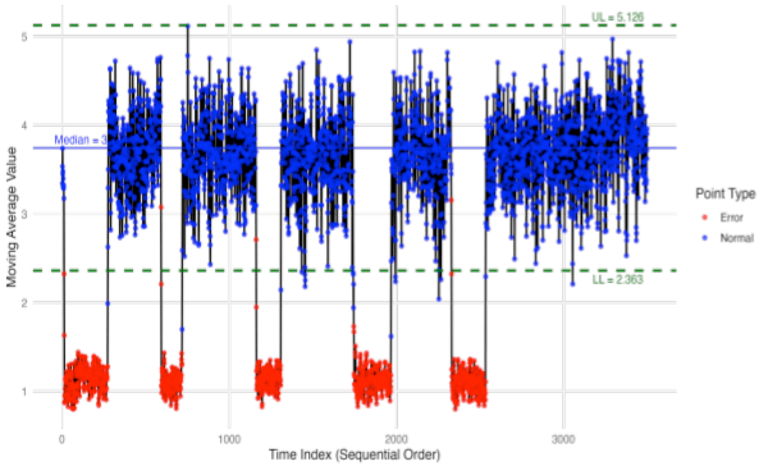

Moving Average Control Chart p15 - Test Set: error_decrease_90
(Window= 3 , a= 3 , b= 3 , TL= 0.05 , CP= 5)

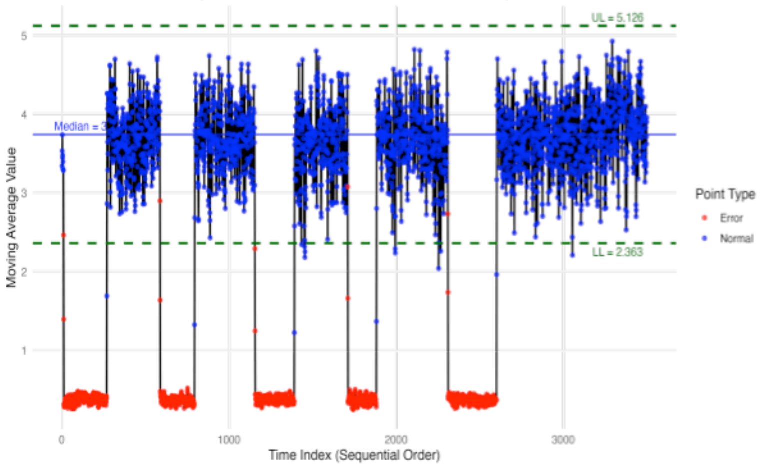

Moving Average Control Chart p16 - Test Set: error_increase_10
(Window= 3 , a= 3 , b= 3 , TL= 0.05 , CP= 5)

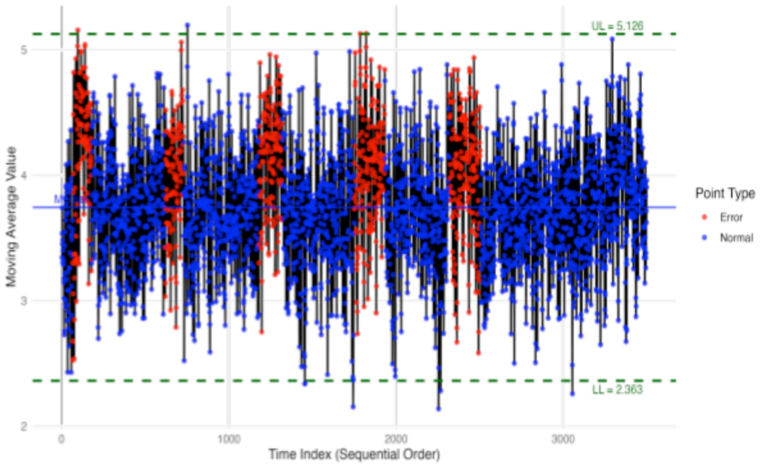

Moving Average Control Chart p17 - Test Set: error_increase_30
(Window= 3 , a= 3 , b= 3 , TL= 0.05 , CP= 5)

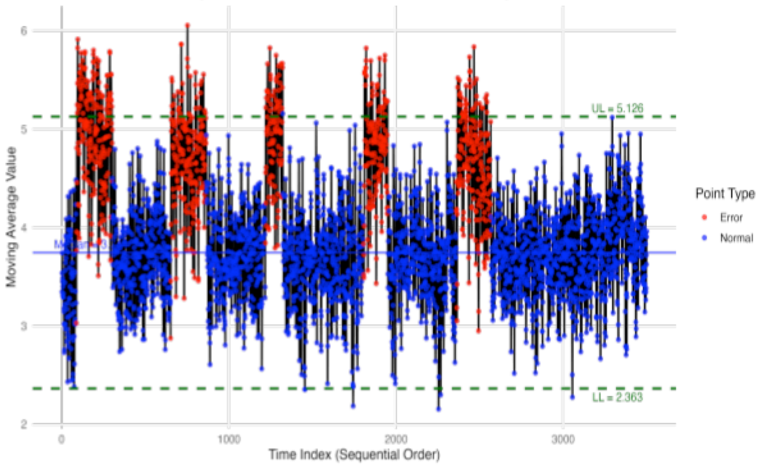

Moving Average Control Chart p18 - Test Set: error_increase_50
(Window= 3 , a= 3 , b= 3 , TL= 0.05 , CP= 5)

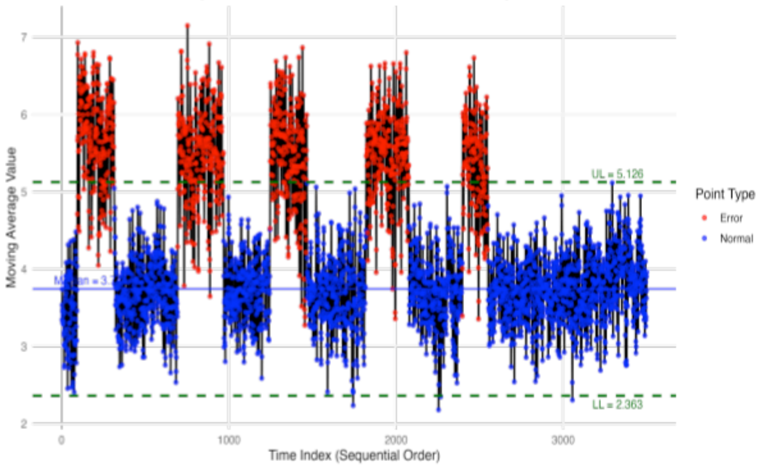

Moving Average Control Chart p19 - Test Set: error_increase_70
(Window= 3 , a= 3 , b= 3 , TL= 0.05 , CP= 5)

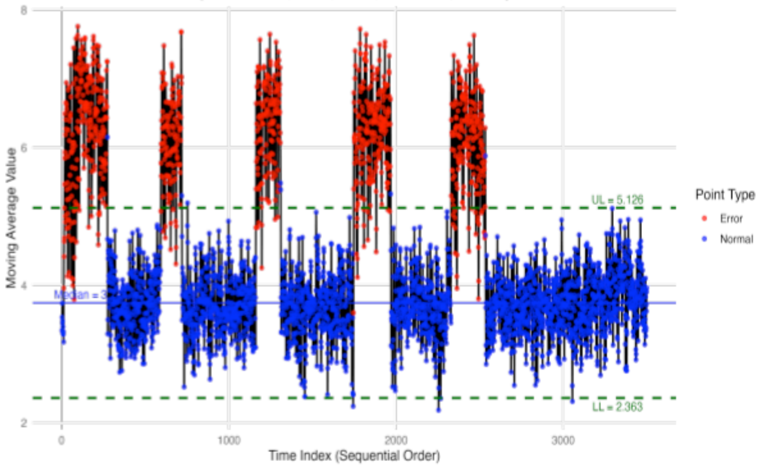

Moving Average Control Chart p20 - Test Set: error_increase_90
(Window= 3 , a= 3 , b= 3 , TL= 0.05 , CP= 5)

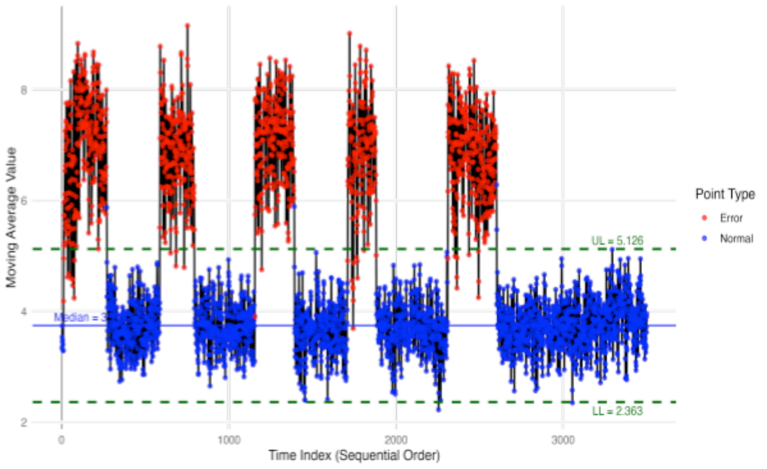

FT3_outputMQ - Training (p1-p10)

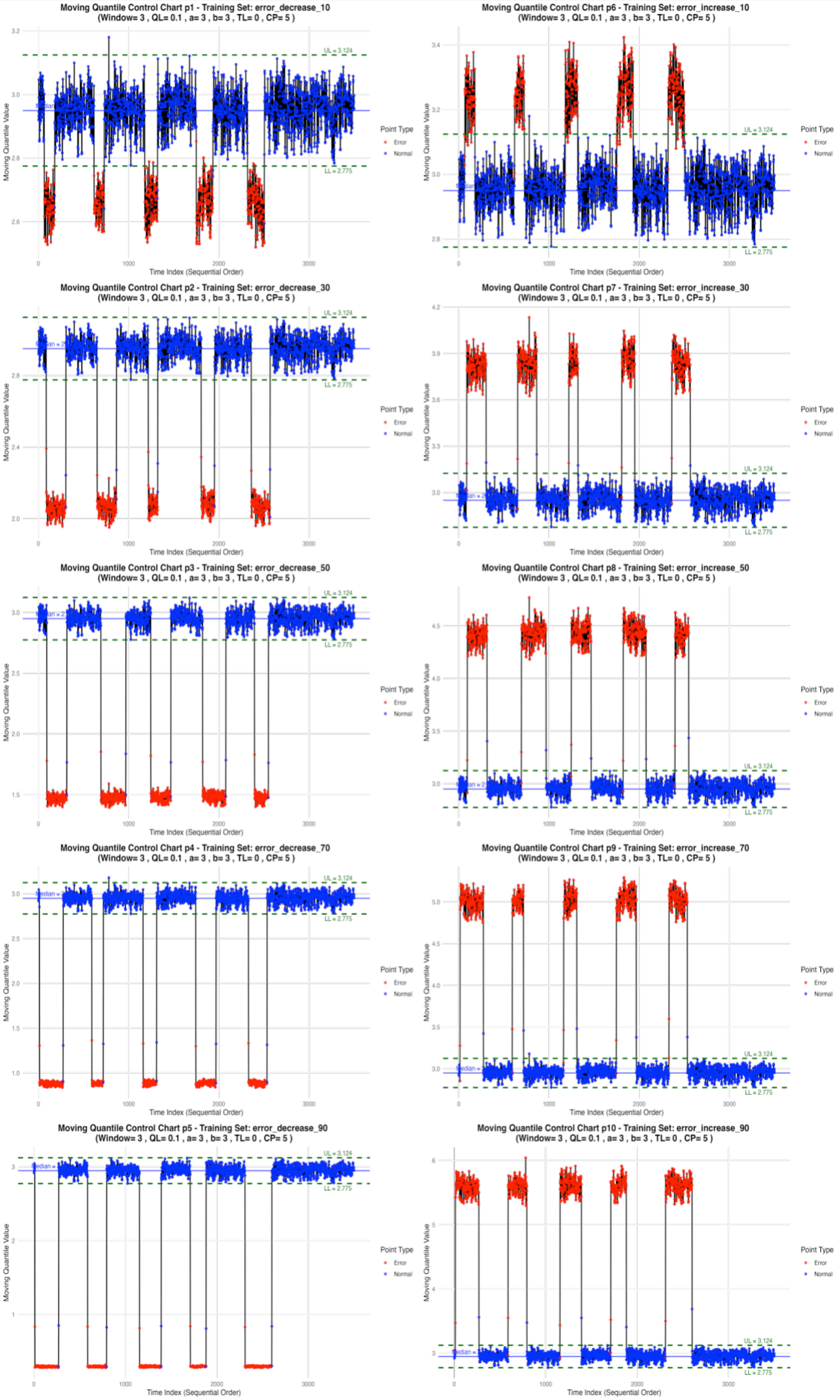

FT3_outputMQ - Test (p11-p20)

Moving Quantile Control Chart p11 - Test Set: error_decrease_10
(Window= 3 , QL= 0.1 , a= 3 , b= 3 , TL= 0 , CP= 5)

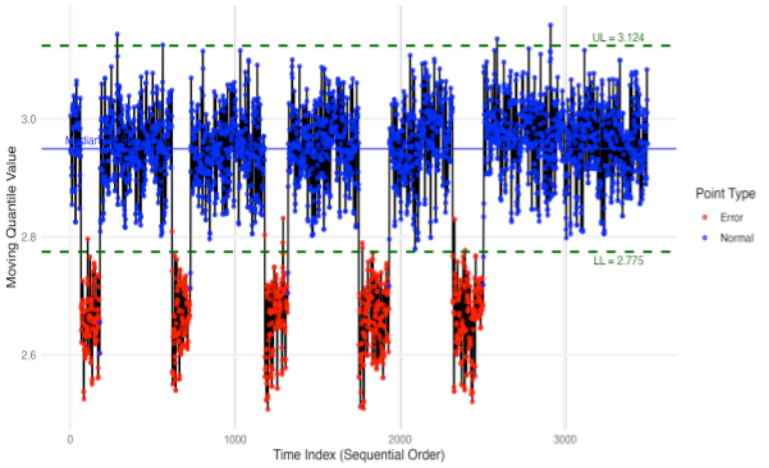

Moving Quantile Control Chart p16 - Test Set: error_increase_10
(Window= 3 , QL= 0.1 , a= 3 , b= 3 , TL= 0 , CP= 5)

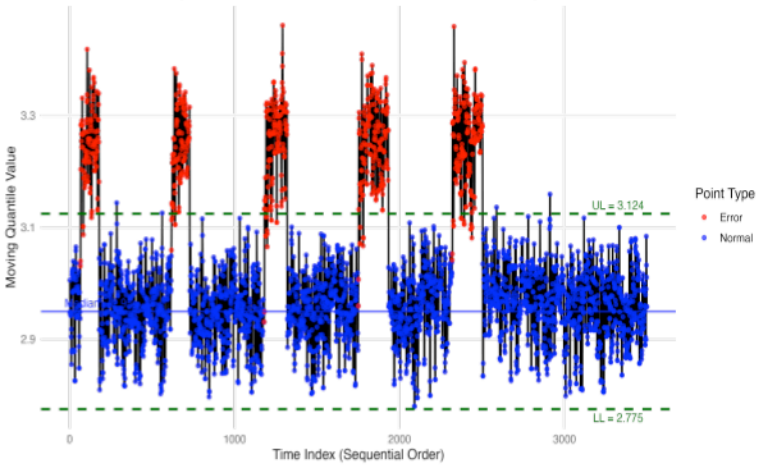

Moving Quantile Control Chart p12 - Test Set: error_decrease_30
(Window= 3 , QL= 0.1 , a= 3 , b= 3 , TL= 0 , CP= 5)

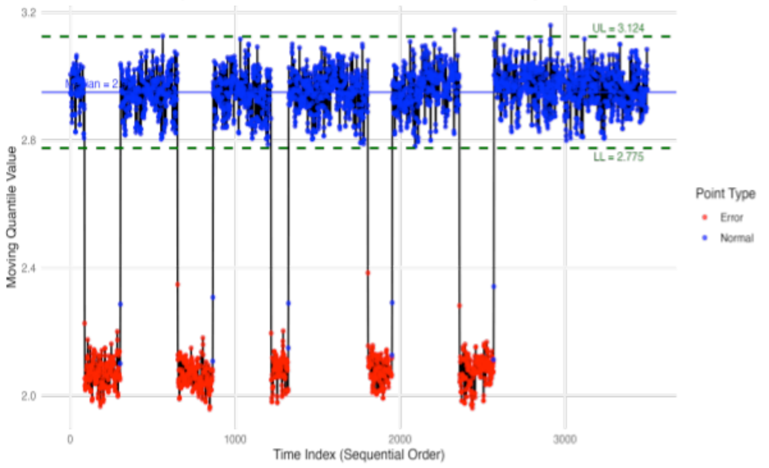

Moving Quantile Control Chart p17 - Test Set: error_increase_30
(Window= 3 , QL= 0.1 , a= 3 , b= 3 , TL= 0 , CP= 5)

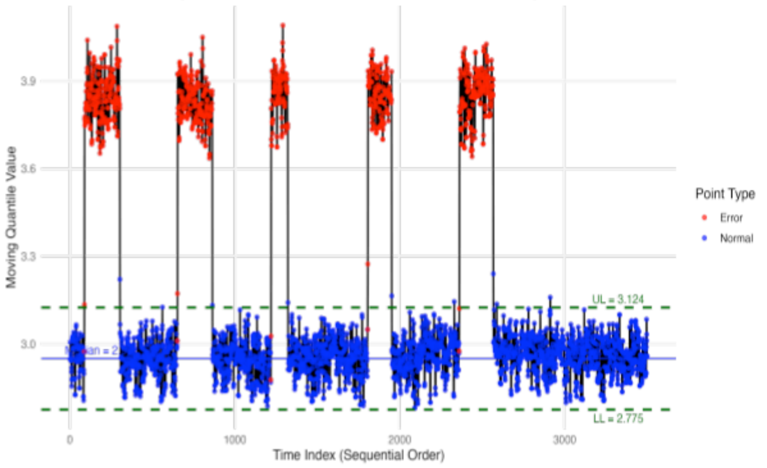

Moving Quantile Control Chart p13 - Test Set: error_decrease_50
(Window= 3 , QL= 0.1 , a= 3 , b= 3 , TL= 0 , CP= 5)

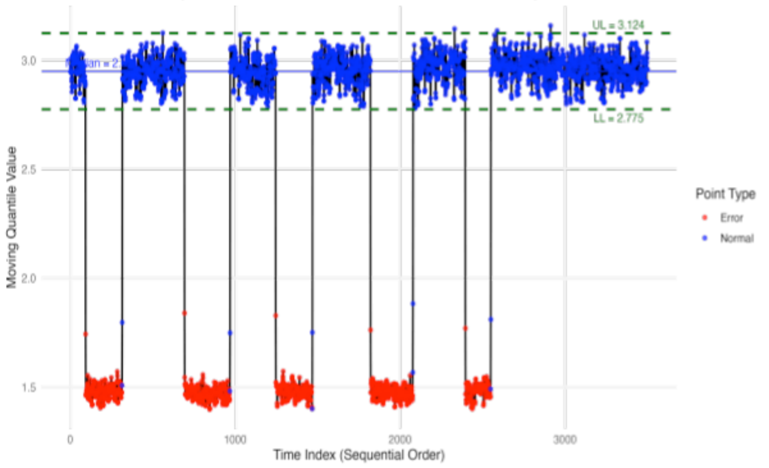

Moving Quantile Control Chart p18 - Test Set: error_increase_50
(Window= 3 , QL= 0.1 , a= 3 , b= 3 , TL= 0 , CP= 5)

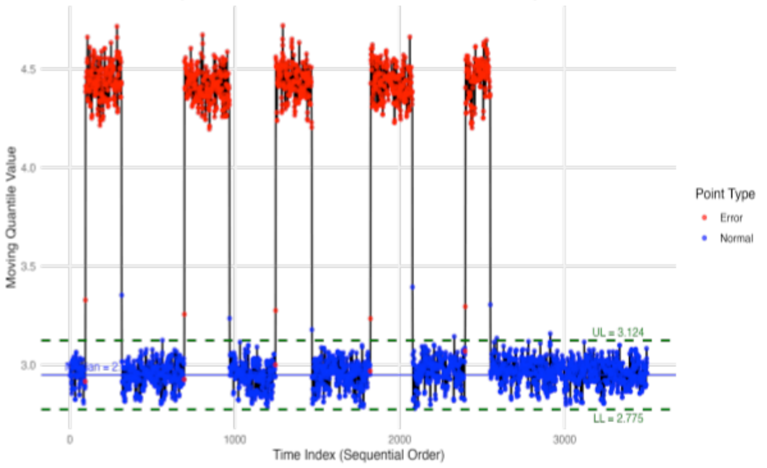

Moving Quantile Control Chart p14 - Test Set: error_decrease_70
(Window= 3 , QL= 0.1 , a= 3 , b= 3 , TL= 0 , CP= 5)

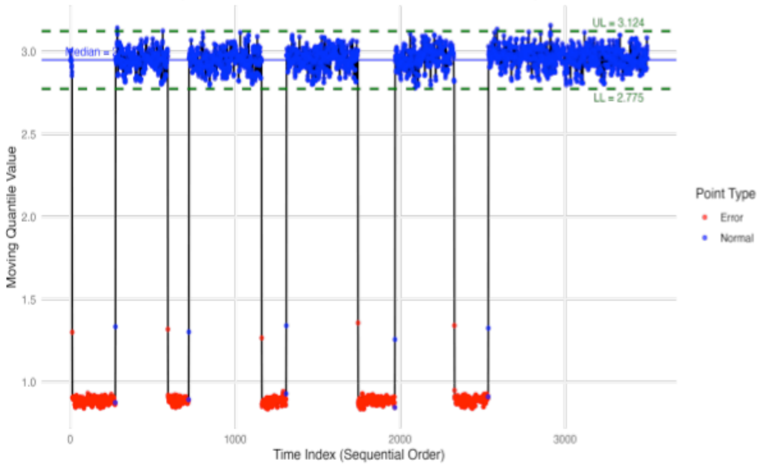

Moving Quantile Control Chart p19 - Test Set: error_increase_70
(Window= 3 , QL= 0.1 , a= 3 , b= 3 , TL= 0 , CP= 5)

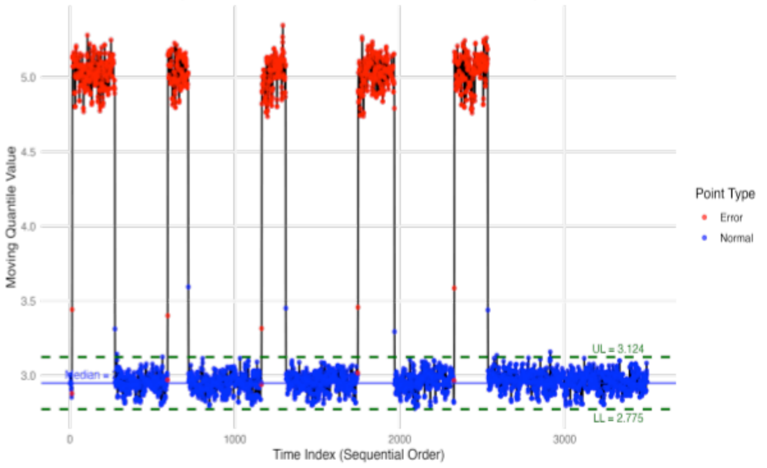

Moving Quantile Control Chart p15 - Test Set: error_decrease_90
(Window= 3 , QL= 0.1 , a= 3 , b= 3 , TL= 0 , CP= 5)

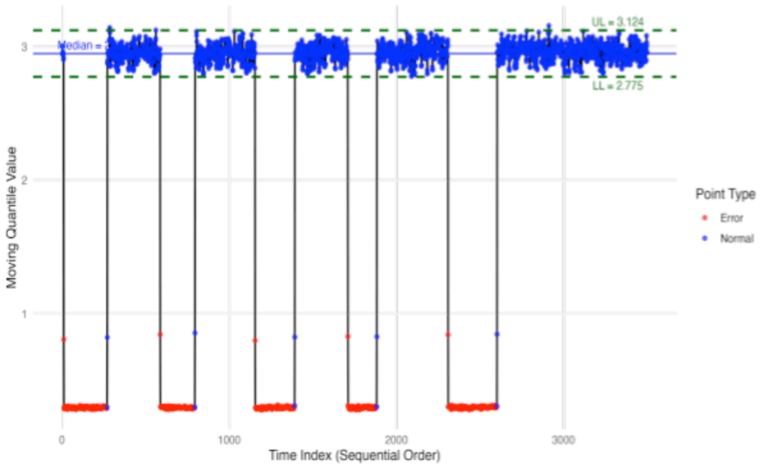

Moving Quantile Control Chart p20 - Test Set: error_increase_90
(Window= 3 , QL= 0.1 , a= 3 , b= 3 , TL= 0 , CP= 5)

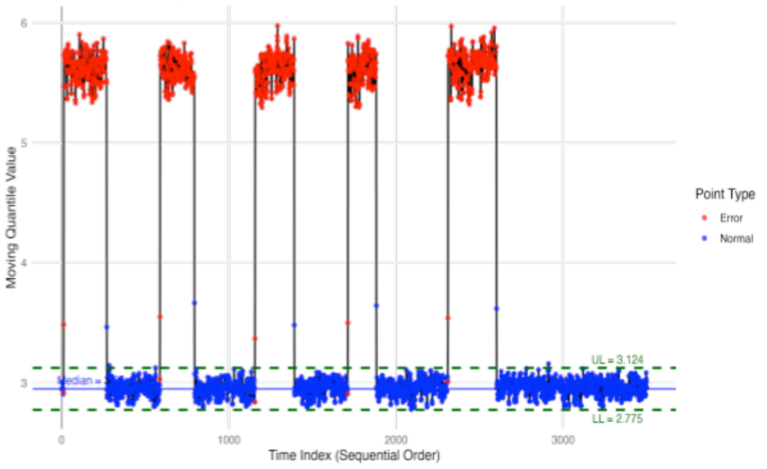

FT3_outputEWMA - Training (p1-p10)

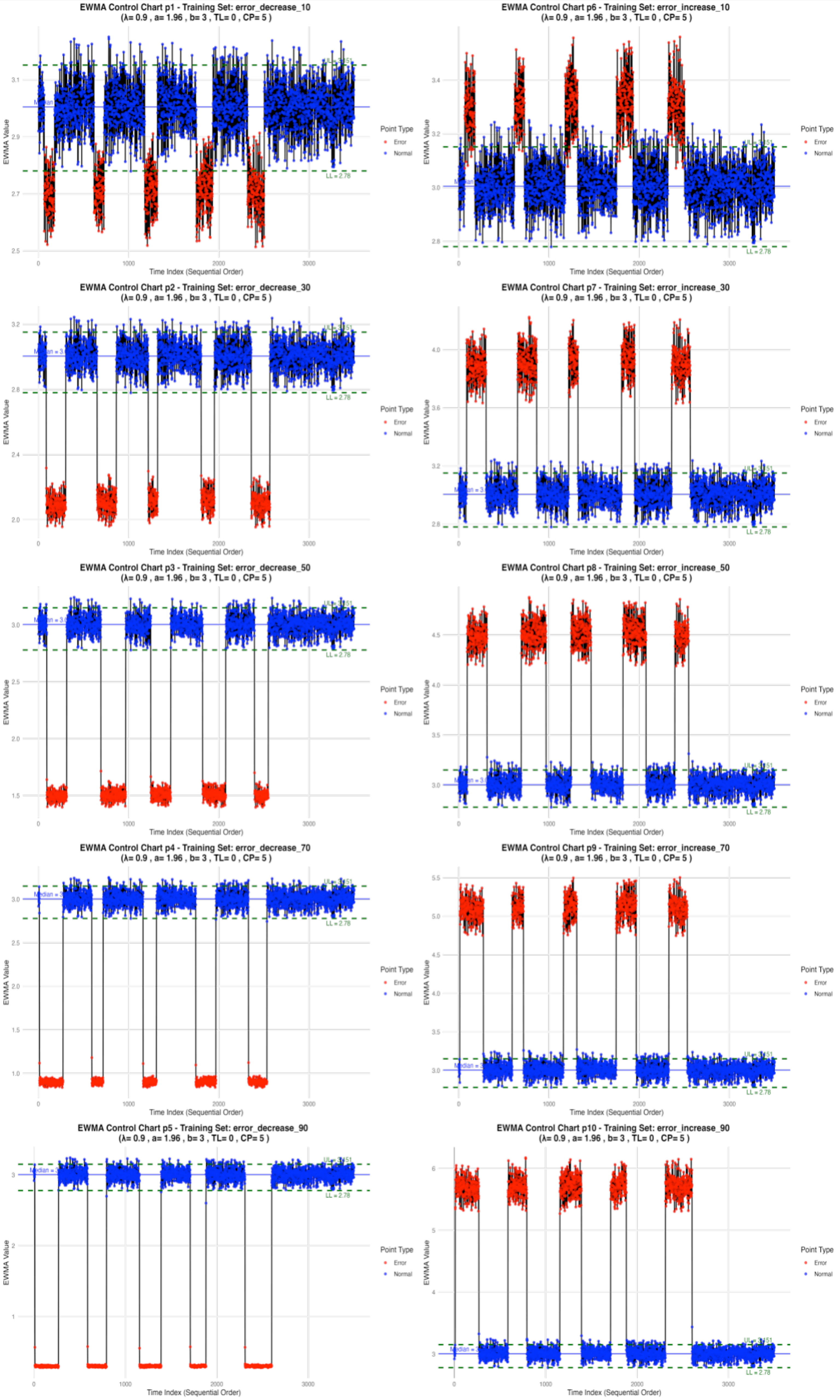

FT3_outputEWMA - Test (p11-p20)

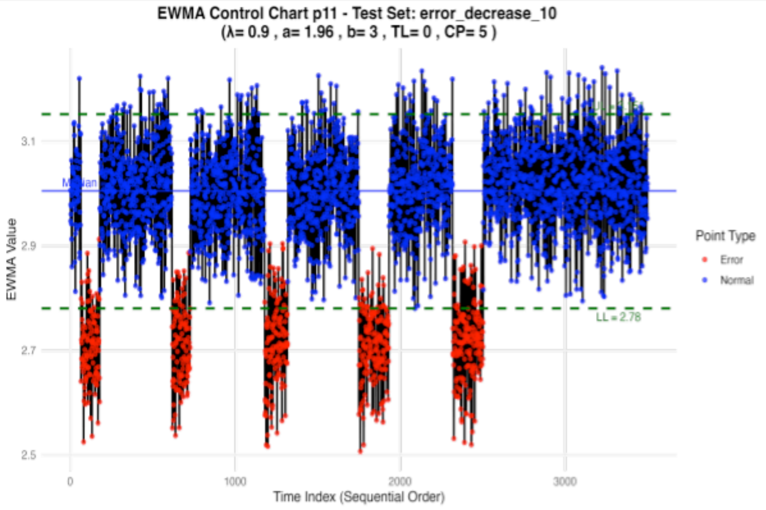

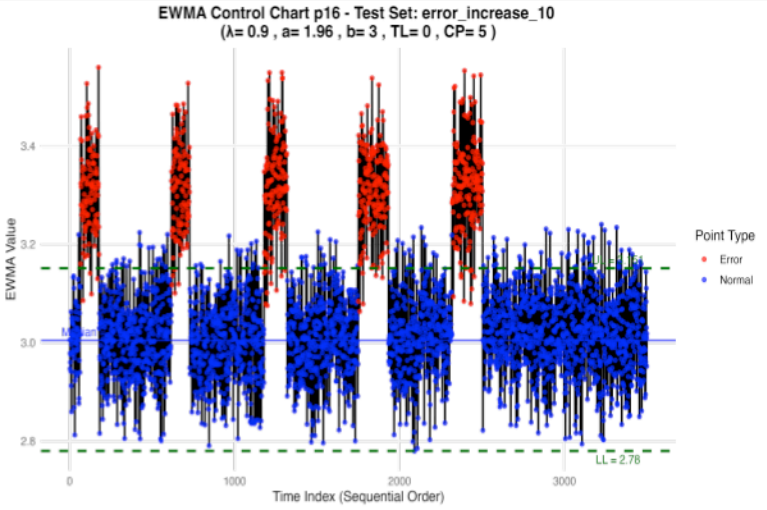

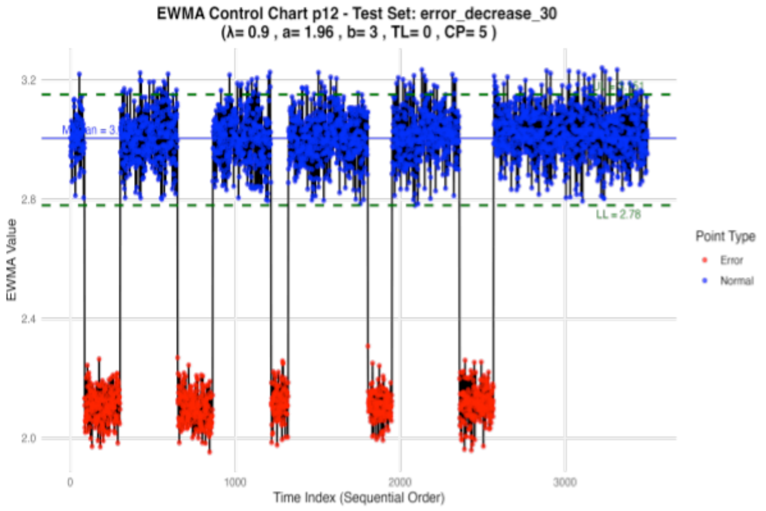

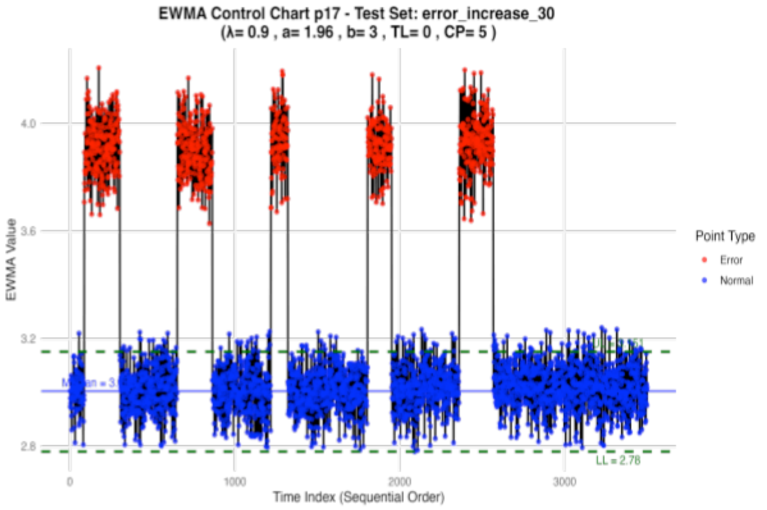

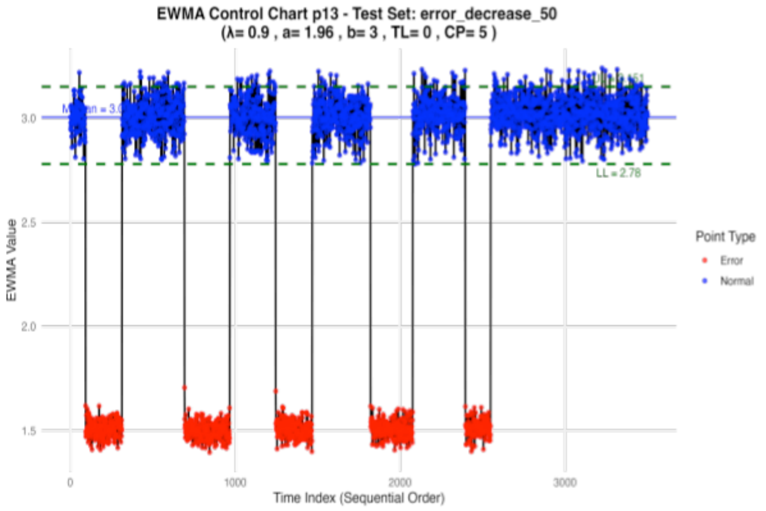

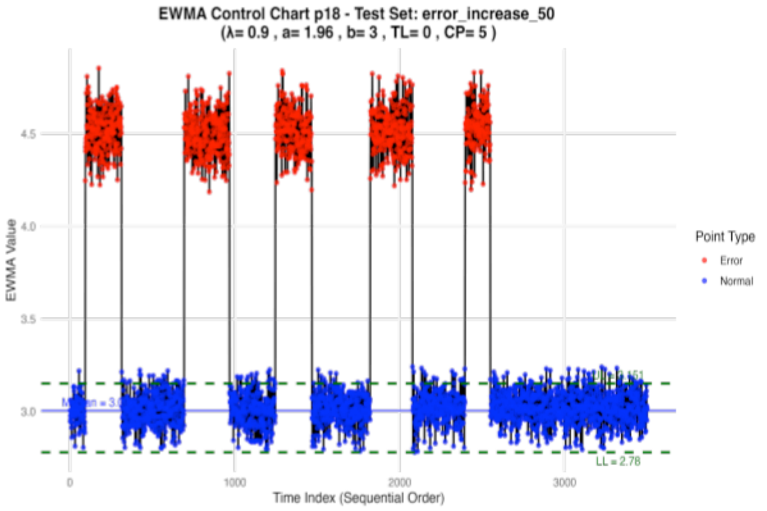

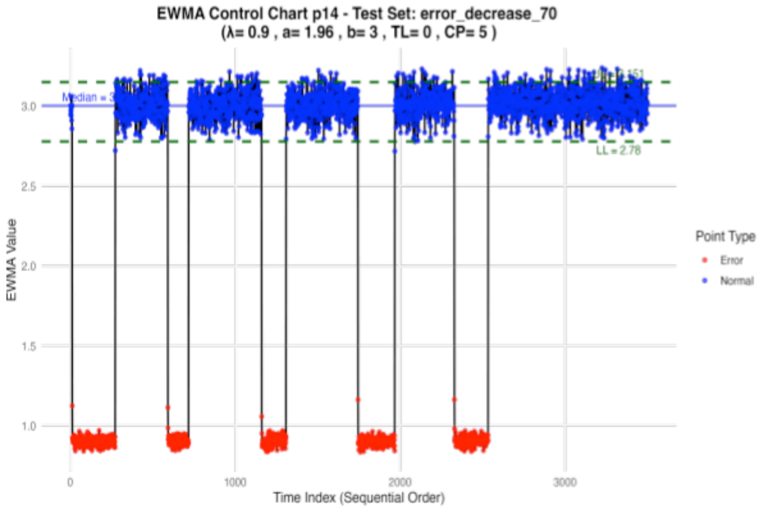

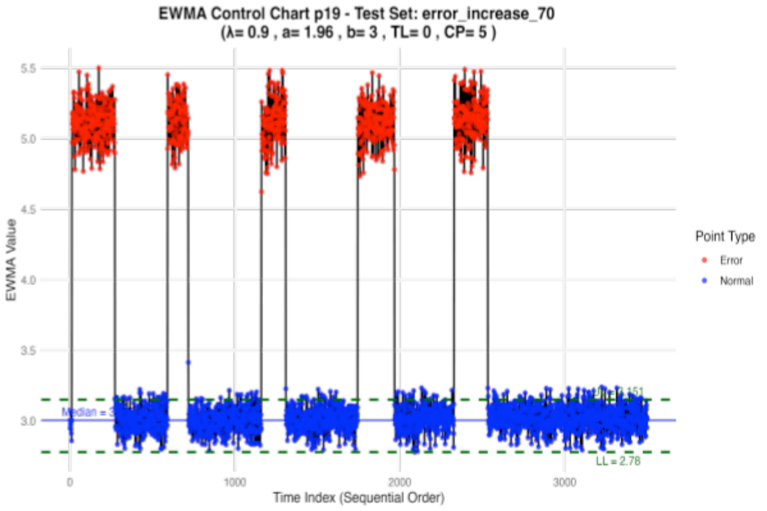

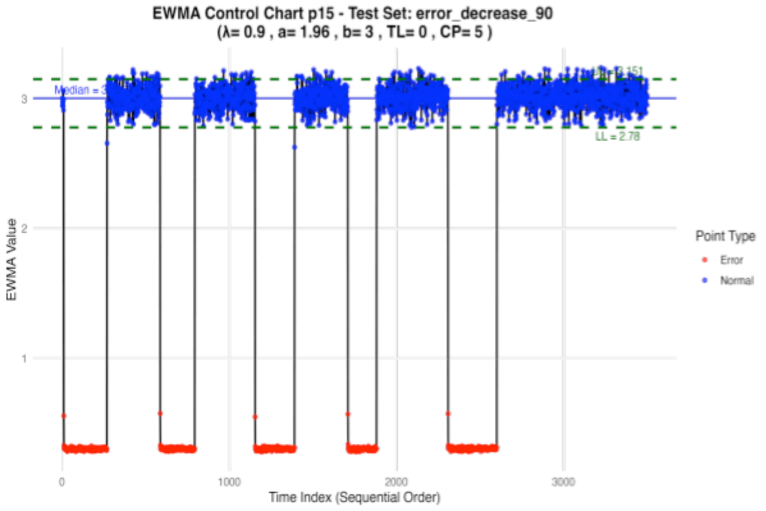

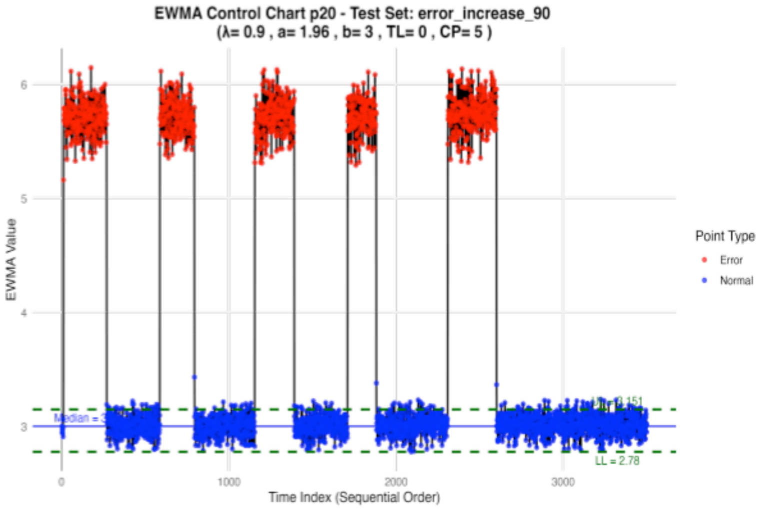

FT3_outputMA - Training (p1-p10)

Moving Average Control Chart p1 - Training Set: error_decrease_10
(Window= 3 , a= 1.64 , b= 1.64 , TL= 0.1 , CP= 10)

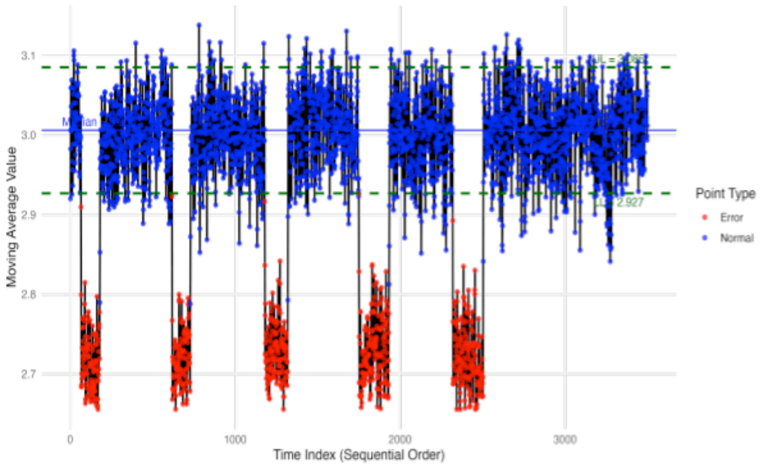

Moving Average Control Chart p6 - Training Set: error_increase_10
(Window= 3 , a= 1.64 , b= 1.64 , TL= 0.1 , CP= 10)

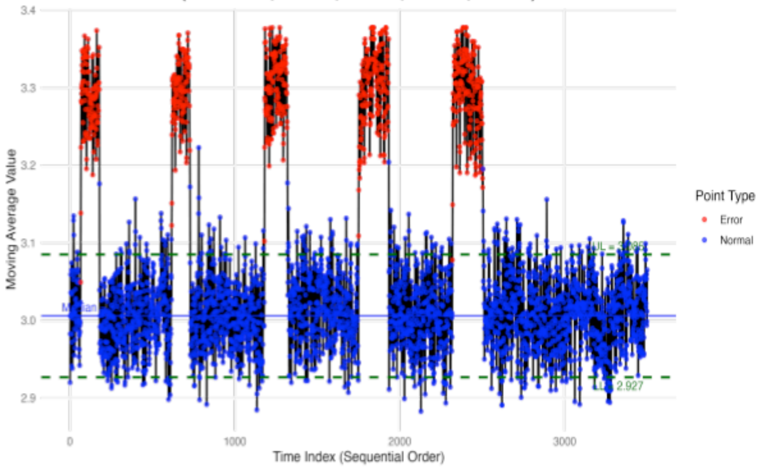

Moving Average Control Chart p2 - Training Set: error_decrease_30
(Window= 3 , a= 1.64 , b= 1.64 , TL= 0.1 , CP= 10)

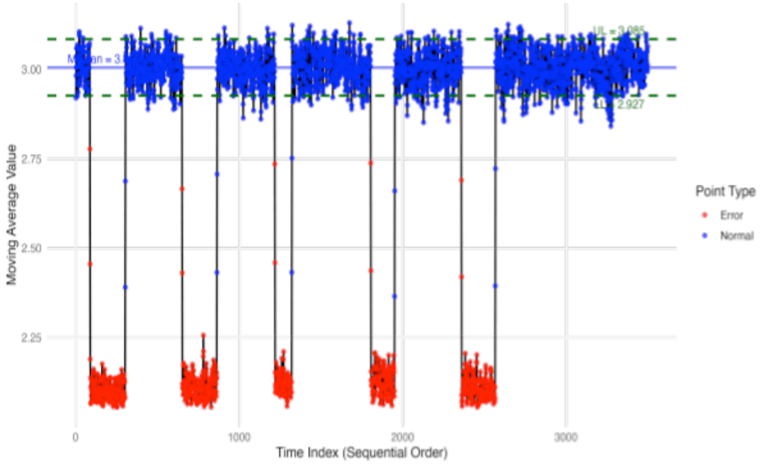

Moving Average Control Chart p7 - Training Set: error_increase_30
(Window= 3 , a= 1.64 , b= 1.64 , TL= 0.1 , CP= 10)

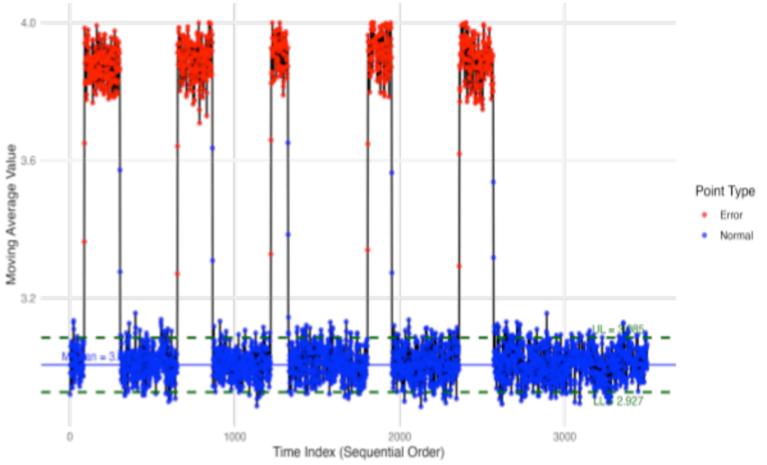

Moving Average Control Chart p3 - Training Set: error_decrease_50
(Window= 3 , a= 1.64 , b= 1.64 , TL= 0.1 , CP= 10)

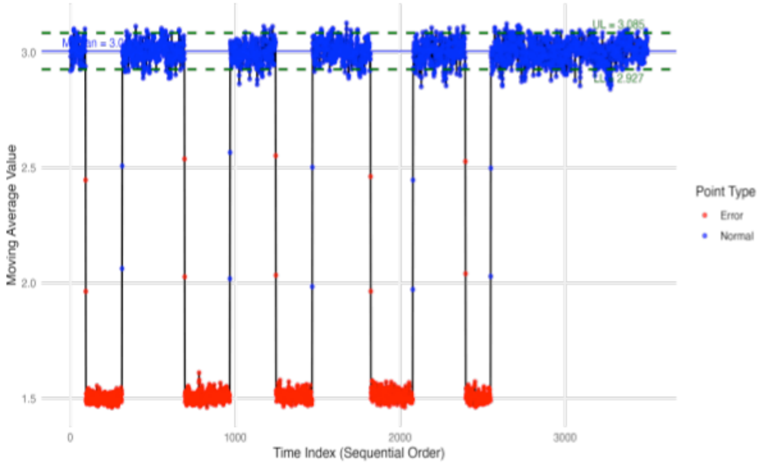

Moving Average Control Chart p8 - Training Set: error_increase_50
(Window= 3 , a= 1.64 , b= 1.64 , TL= 0.1 , CP= 10)

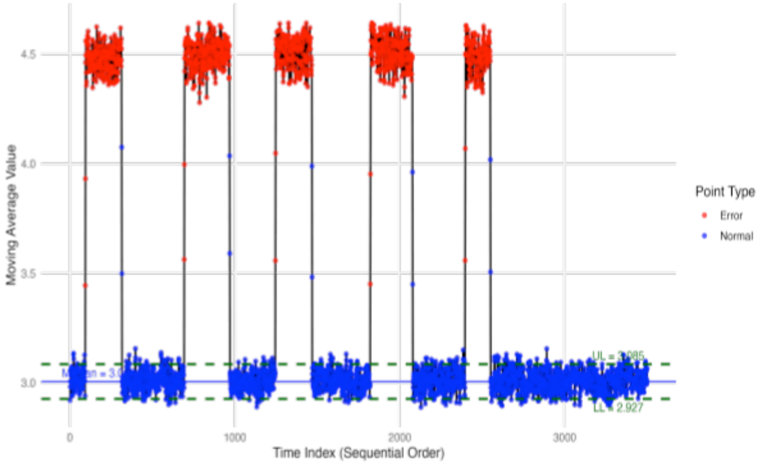

Moving Average Control Chart p4 - Training Set: error_decrease_70
(Window= 3 , a= 1.64 , b= 1.64 , TL= 0.1 , CP= 10)

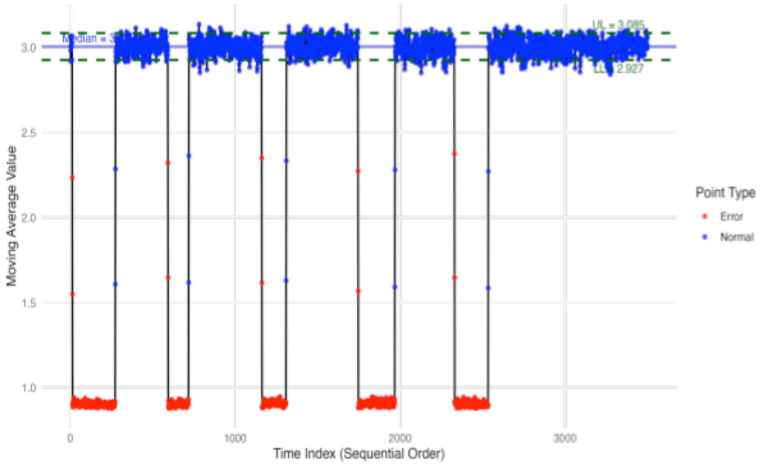

Moving Average Control Chart p9 - Training Set: error_increase_70
(Window= 3 , a= 1.64 , b= 1.64 , TL= 0.1 , CP= 10)

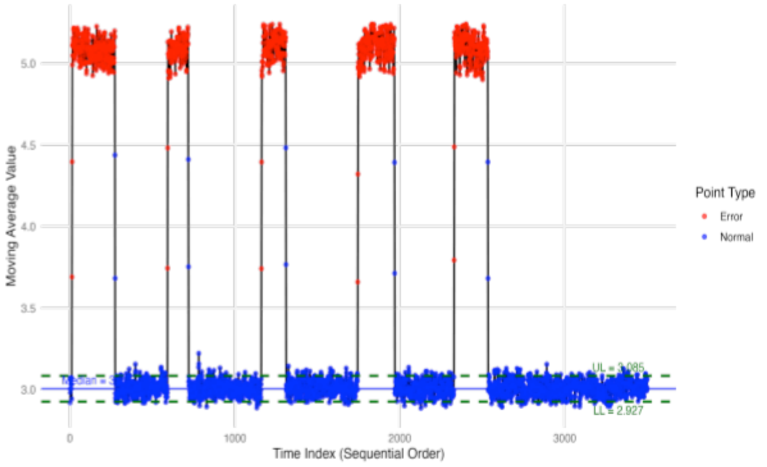

Moving Average Control Chart p5 - Training Set: error_decrease_90
(Window= 3 , a= 1.64 , b= 1.64 , TL= 0.1 , CP= 10)

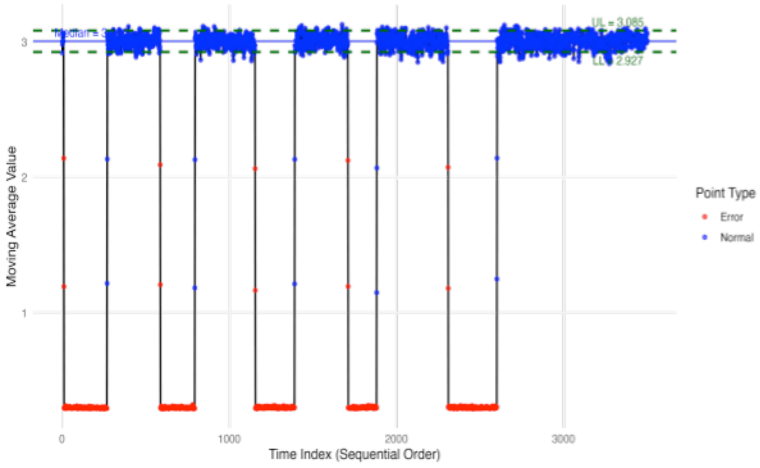

Moving Average Control Chart p10 - Training Set: error_increase_90
(Window= 3 , a= 1.64 , b= 1.64 , TL= 0.1 , CP= 10)

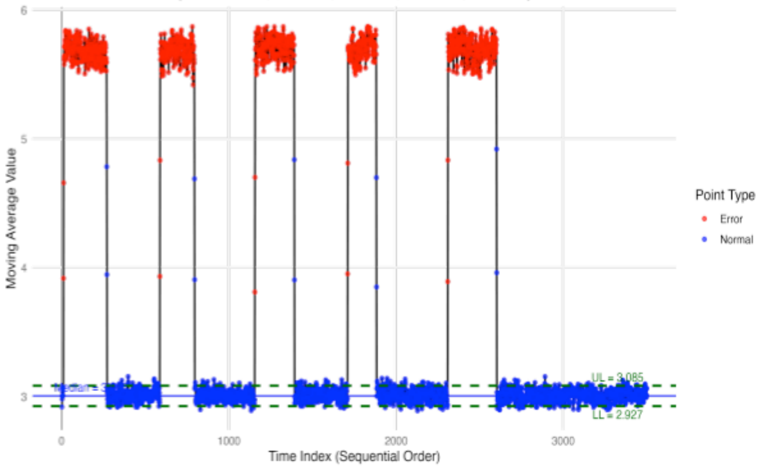

FT3_outputMA - Test (p11-p20)

Moving Average Control Chart p11 - Test Set: error_decrease_10
(Window= 3 , a= 1.64 , b= 1.64 , TL= 0.1 , CP= 10)

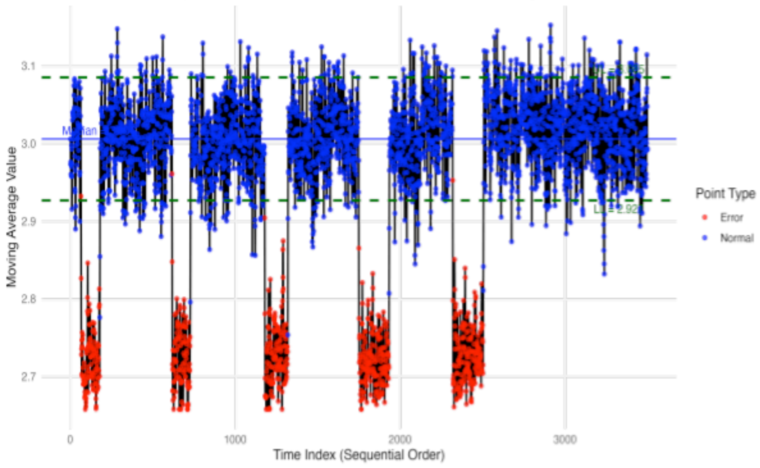

Moving Average Control Chart p16 - Test Set: error_increase_10
(Window= 3 , a= 1.64 , b= 1.64 , TL= 0.1 , CP= 10)

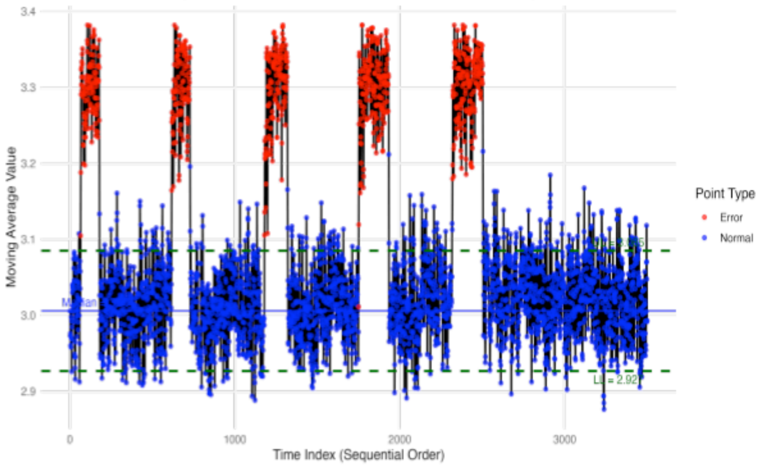

Moving Average Control Chart p12 - Test Set: error_decrease_30
(Window= 3 , a= 1.64 , b= 1.64 , TL= 0.1 , CP= 10)

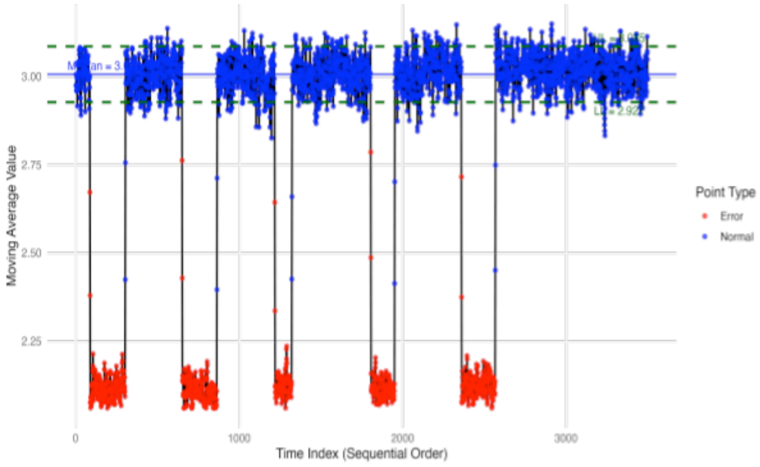

Moving Average Control Chart p17 - Test Set: error_increase_30
(Window= 3 , a= 1.64 , b= 1.64 , TL= 0.1 , CP= 10)

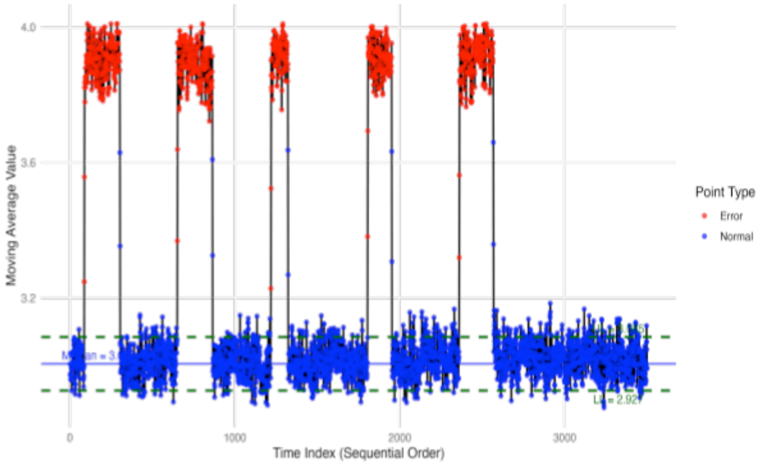

Moving Average Control Chart p13 - Test Set: error_decrease_50
(Window= 3 , a= 1.64 , b= 1.64 , TL= 0.1 , CP= 10)

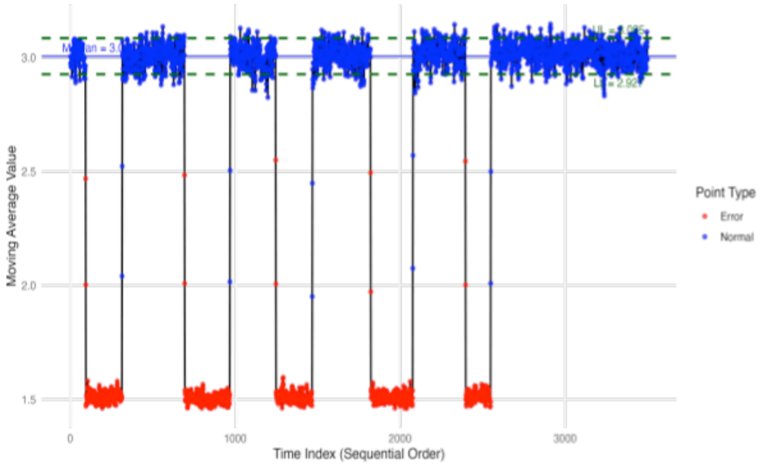

Moving Average Control Chart p18 - Test Set: error_increase_50
(Window= 3 , a= 1.64 , b= 1.64 , TL= 0.1 , CP= 10)

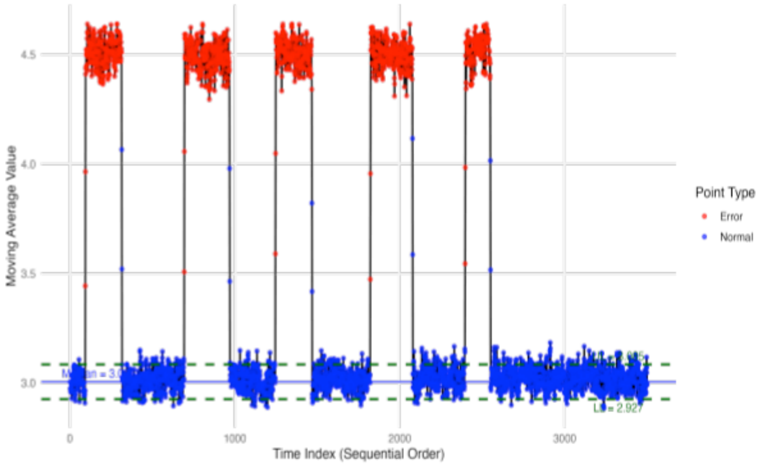

Moving Average Control Chart p14 - Test Set: error_decrease_70
(Window= 3 , a= 1.64 , b= 1.64 , TL= 0.1 , CP= 10)

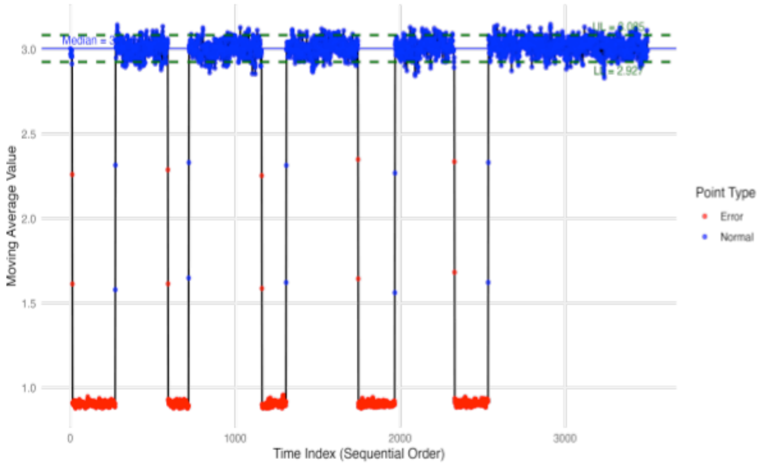

Moving Average Control Chart p19 - Test Set: error_increase_70
(Window= 3 , a= 1.64 , b= 1.64 , TL= 0.1 , CP= 10)

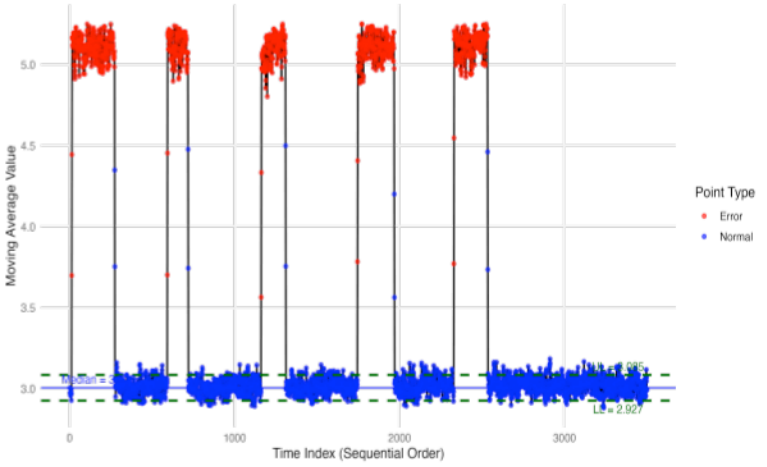

Moving Average Control Chart p15 - Test Set: error_decrease_90
(Window= 3 , a= 1.64 , b= 1.64 , TL= 0.1 , CP= 10)

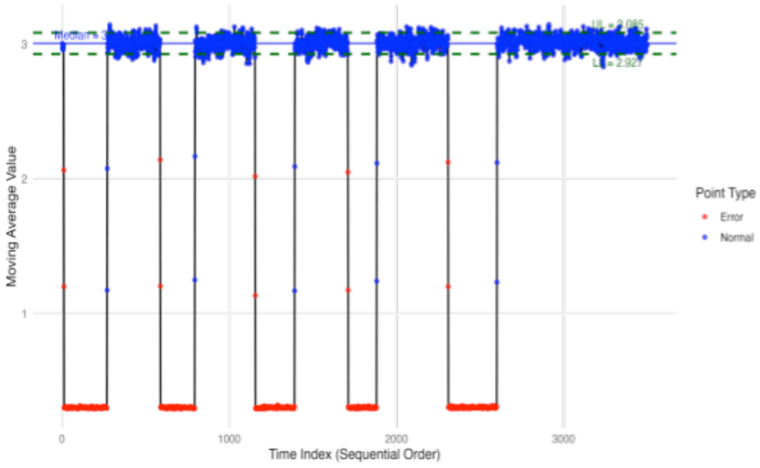

Moving Average Control Chart p20 - Test Set: error_increase_90
(Window= 3 , a= 1.64 , b= 1.64 , TL= 0.1 , CP= 10)

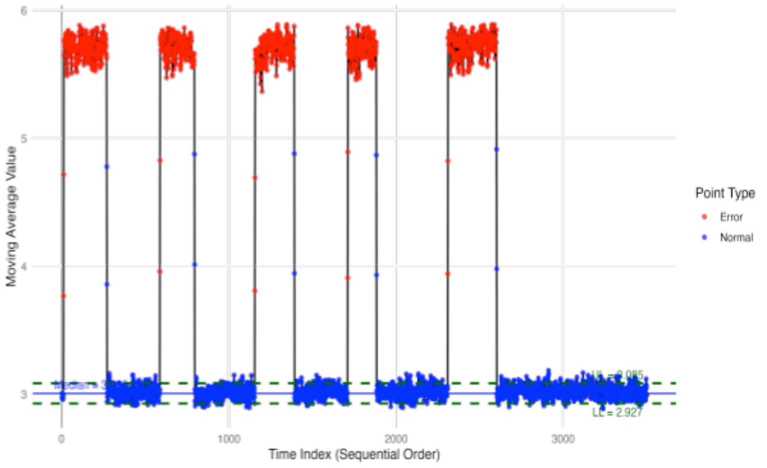

FT4_outputMQ - Training (p1-p10)

Moving Quantile Control Chart p1 - Training Set: error_decrease_10
(Window= 3 , QL= 0.9 , a= 1.96 , b= 3 , TL= 0 , CP= 5)

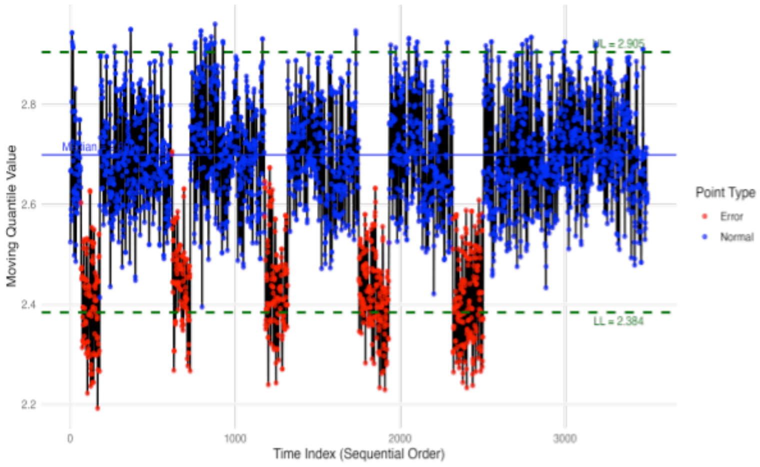

Moving Quantile Control Chart p6 - Training Set: error_increase_10
(Window= 3 , QL= 0.9 , a= 1.96 , b= 3 , TL= 0 , CP= 5)

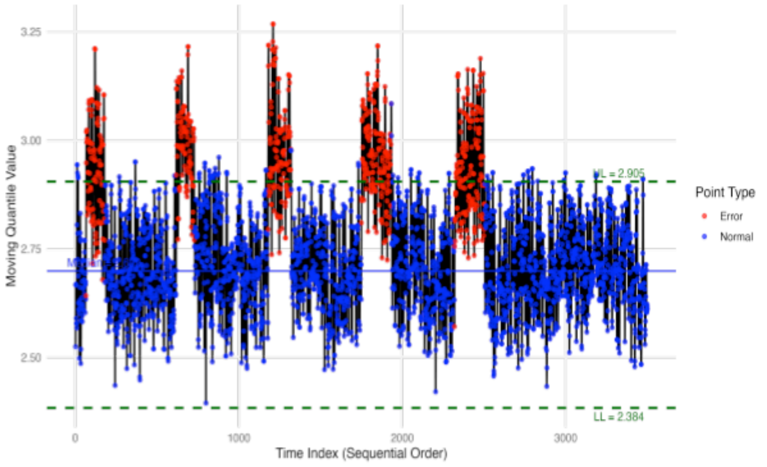

Moving Quantile Control Chart p2 - Training Set: error_decrease_30
(Window= 3 , QL= 0.9 , a= 1.96 , b= 3 , TL= 0 , CP= 5)

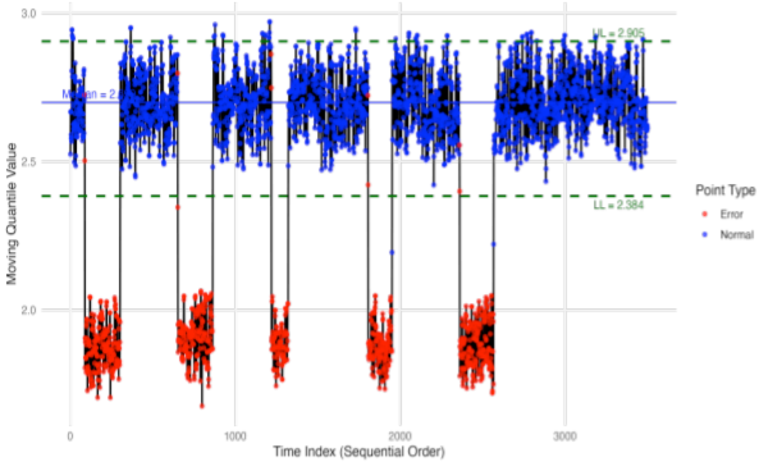

Moving Quantile Control Chart p7 - Training Set: error_increase_30
(Window= 3 , QL= 0.9 , a= 1.96 , b= 3 , TL= 0 , CP= 5)

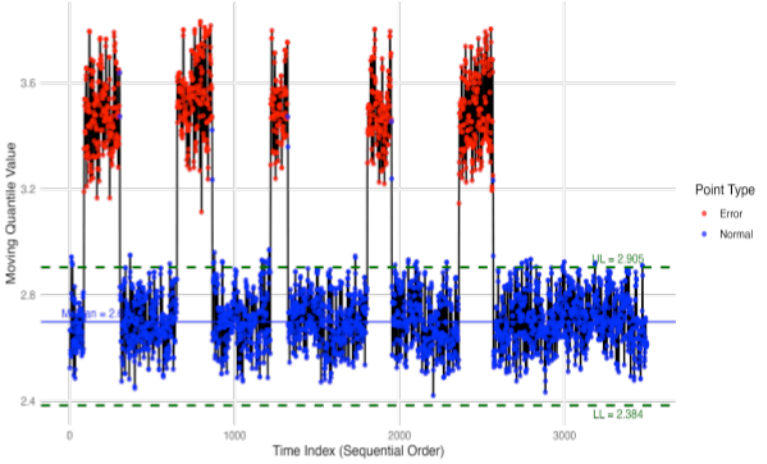

Moving Quantile Control Chart p3 - Training Set: error_decrease_50
(Window= 3 , QL= 0.9 , a= 1.96 , b= 3 , TL= 0 , CP= 5)

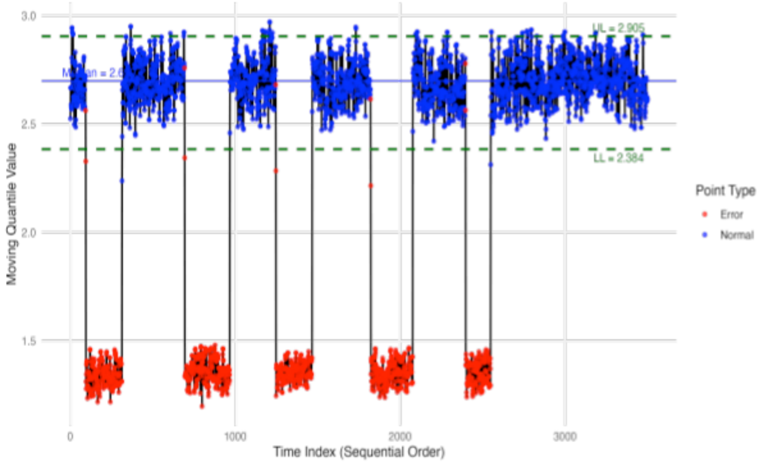

Moving Quantile Control Chart p8 - Training Set: error_increase_50
(Window= 3 , QL= 0.9 , a= 1.96 , b= 3 , TL= 0 , CP= 5)

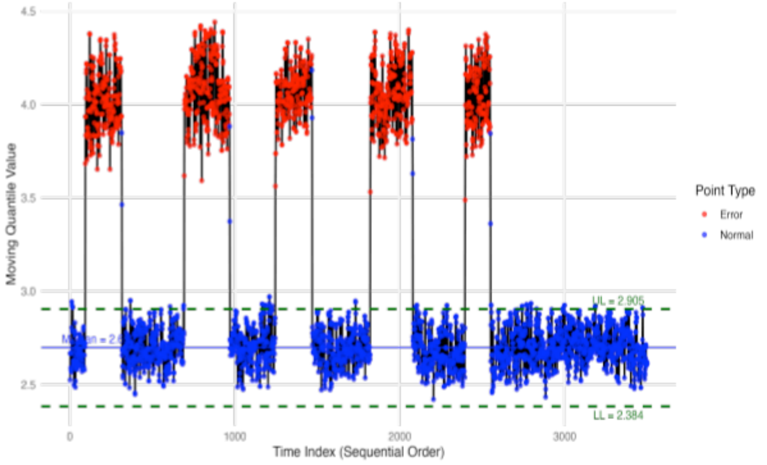

Moving Quantile Control Chart p4 - Training Set: error_decrease_70
(Window= 3 , QL= 0.9 , a= 1.96 , b= 3 , TL= 0 , CP= 5)

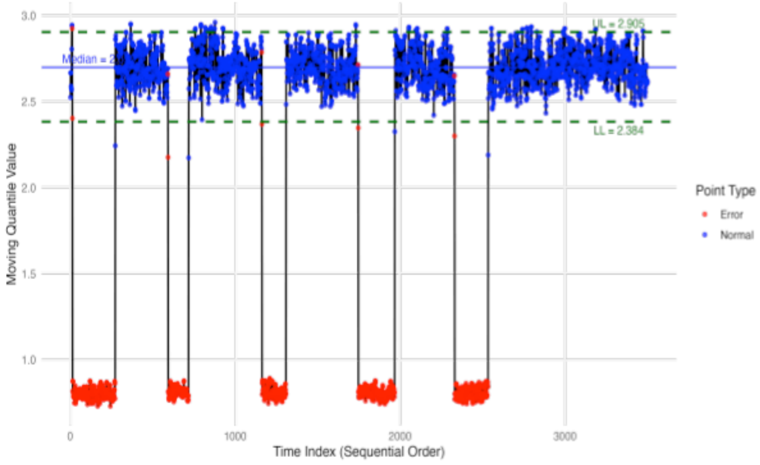

Moving Quantile Control Chart p9 - Training Set: error_increase_70
(Window= 3 , QL= 0.9 , a= 1.96 , b= 3 , TL= 0 , CP= 5)

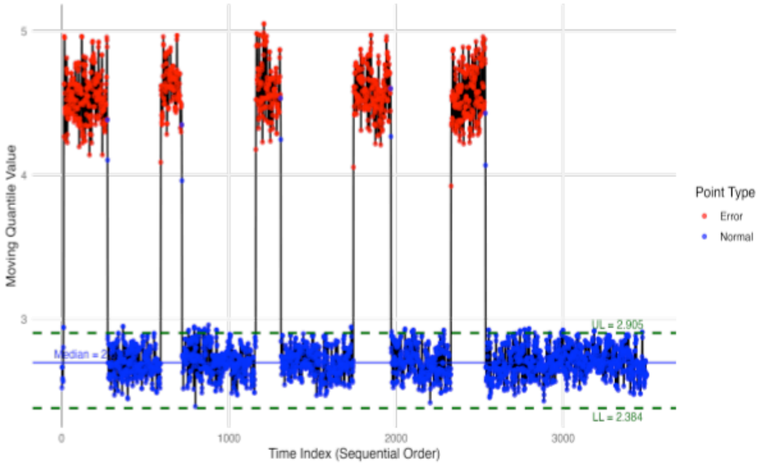

Moving Quantile Control Chart p5 - Training Set: error_decrease_90
(Window= 3 , QL= 0.9 , a= 1.96 , b= 3 , TL= 0 , CP= 5)

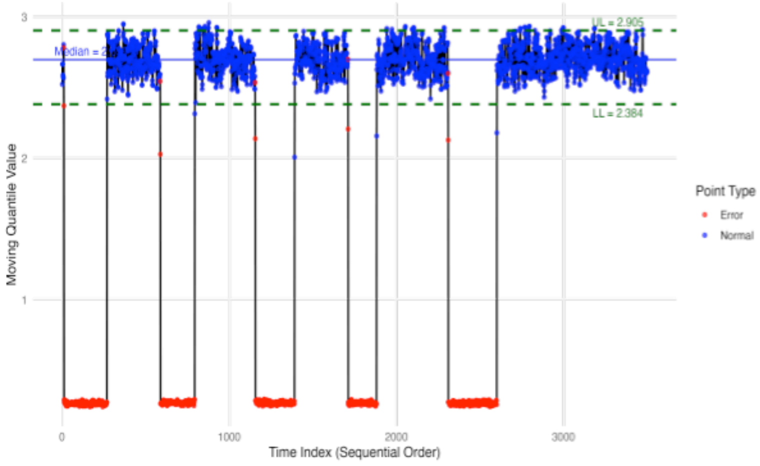

Moving Quantile Control Chart p10 - Training Set: error_increase_90
(Window= 3 , QL= 0.9 , a= 1.96 , b= 3 , TL= 0 , CP= 5)

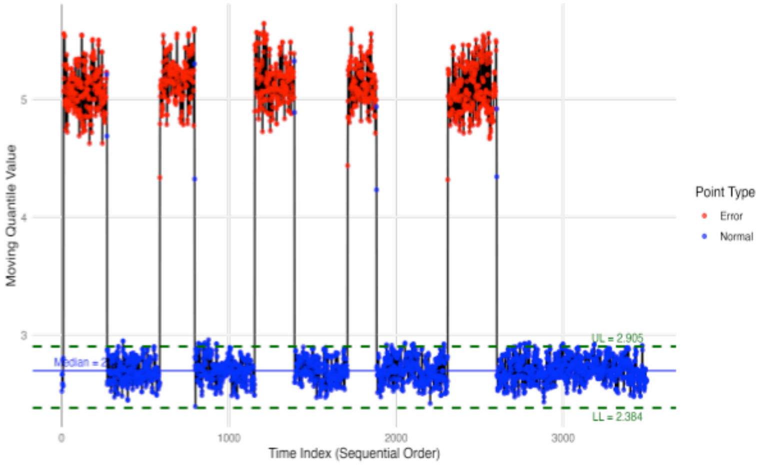

FT4_outputMQ - Test (p11-p20)

Moving Quantile Control Chart p11 - Test Set: error_decrease_10
(Window= 3 , QL= 0.9 , a= 1.96 , b= 3 , TL= 0 , CP= 5)

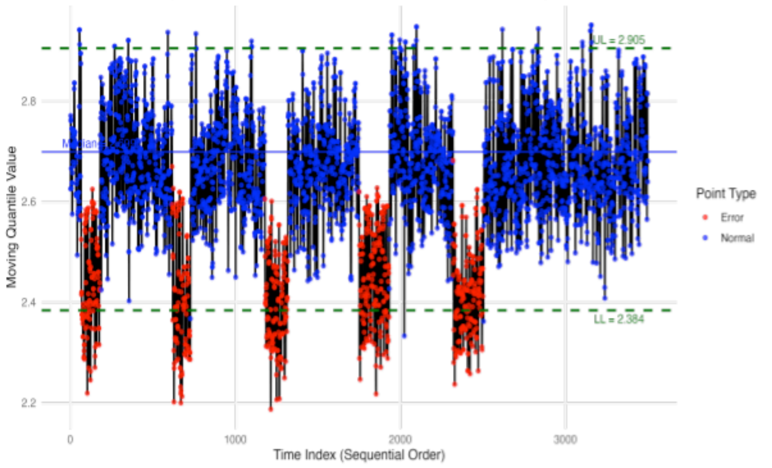

Moving Quantile Control Chart p16 - Test Set: error_increase_10
(Window= 3 , QL= 0.9 , a= 1.96 , b= 3 , TL= 0 , CP= 5)

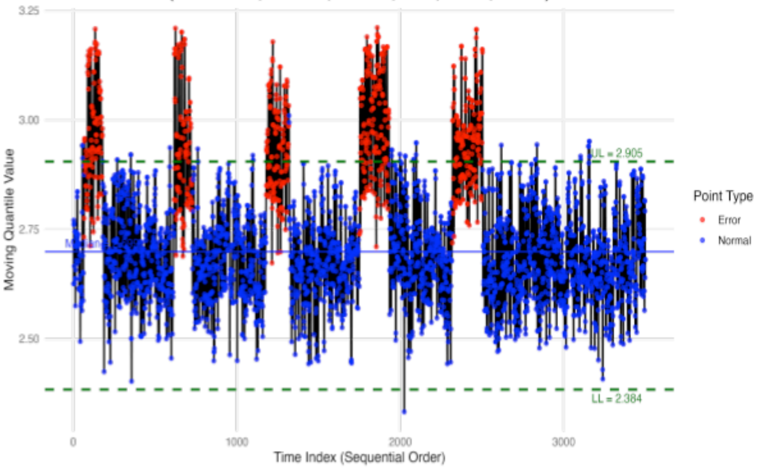

Moving Quantile Control Chart p12 - Test Set: error_decrease_30
(Window= 3 , QL= 0.9 , a= 1.96 , b= 3 , TL= 0 , CP= 5)

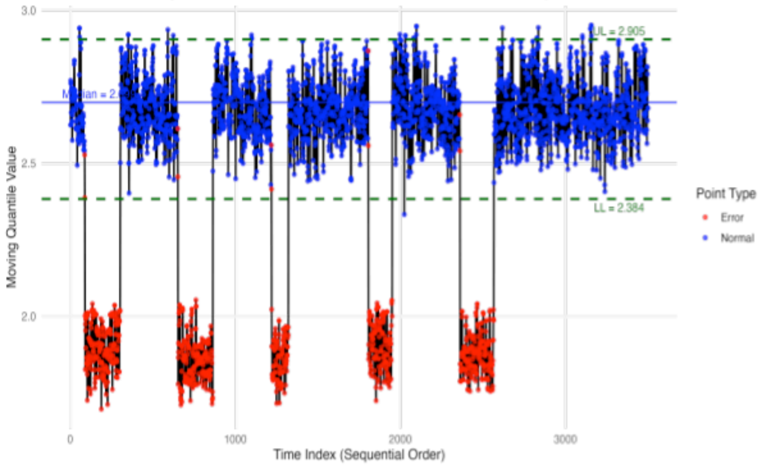

Moving Quantile Control Chart p17 - Test Set: error_increase_30
(Window= 3 , QL= 0.9 , a= 1.96 , b= 3 , TL= 0 , CP= 5)

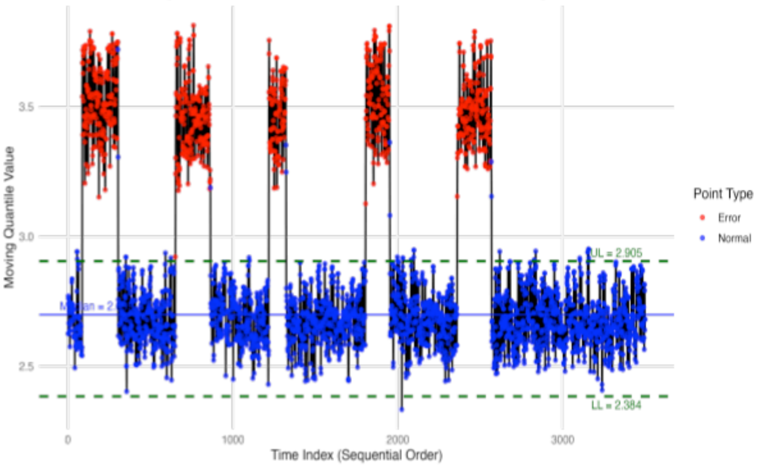

Moving Quantile Control Chart p13 - Test Set: error_decrease_50
(Window= 3 , QL= 0.9 , a= 1.96 , b= 3 , TL= 0 , CP= 5)

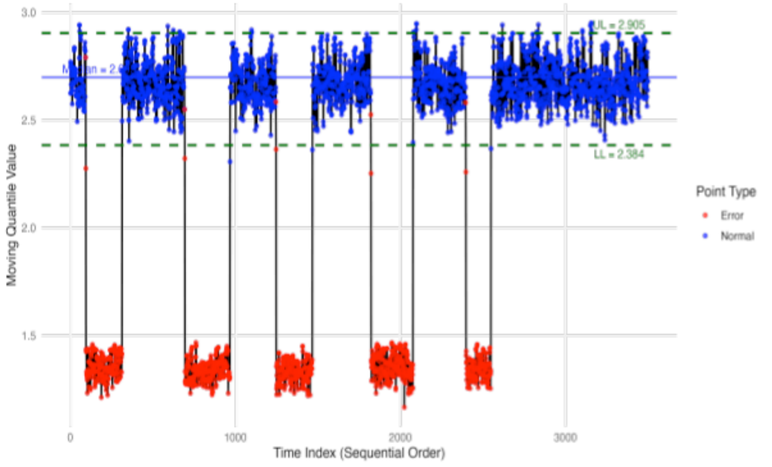

Moving Quantile Control Chart p18 - Test Set: error_increase_50
(Window= 3 , QL= 0.9 , a= 1.96 , b= 3 , TL= 0 , CP= 5)

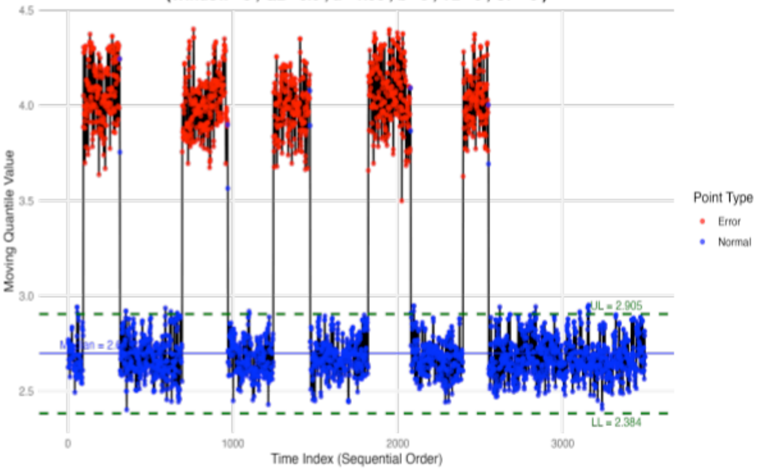

Moving Quantile Control Chart p14 - Test Set: error_decrease_70
(Window= 3 , QL= 0.9 , a= 1.96 , b= 3 , TL= 0 , CP= 5)

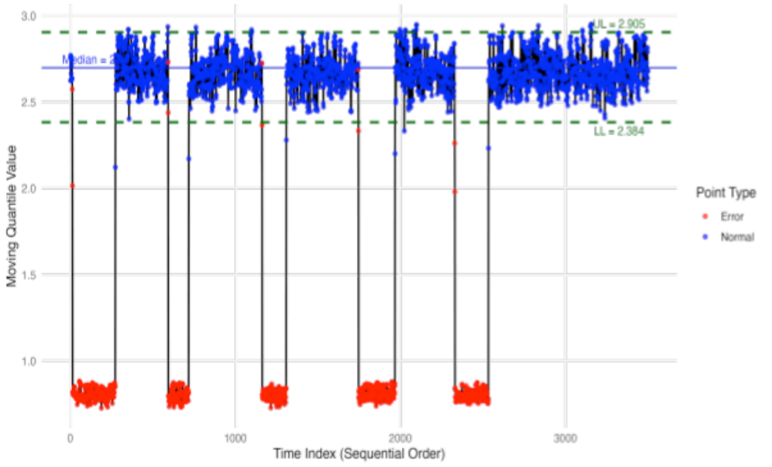

Moving Quantile Control Chart p19 - Test Set: error_increase_70
(Window= 3 , QL= 0.9 , a= 1.96 , b= 3 , TL= 0 , CP= 5)

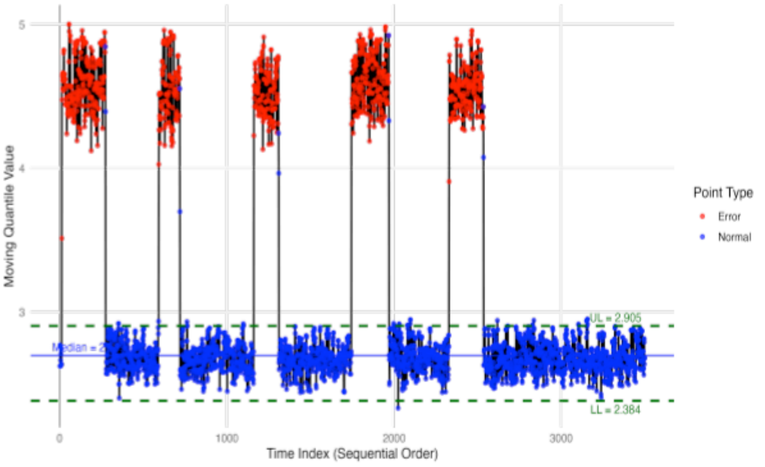

Moving Quantile Control Chart p15 - Test Set: error_decrease_90
(Window= 3 , QL= 0.9 , a= 1.96 , b= 3 , TL= 0 , CP= 5)

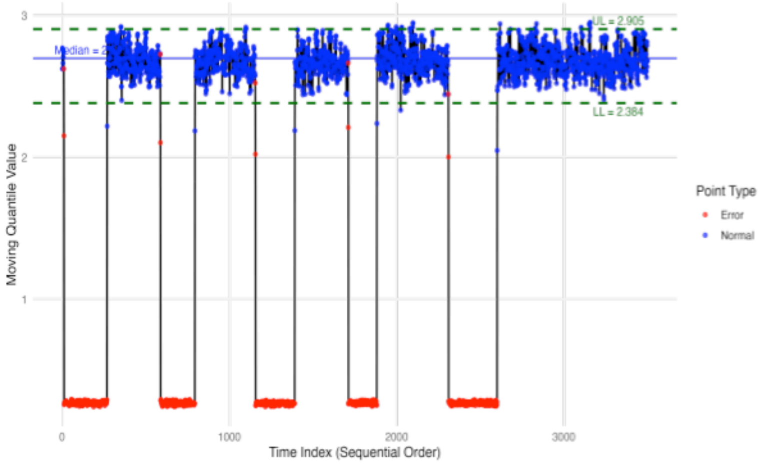

Moving Quantile Control Chart p20 - Test Set: error_increase_90
(Window= 3 , QL= 0.9 , a= 1.96 , b= 3 , TL= 0 , CP= 5)

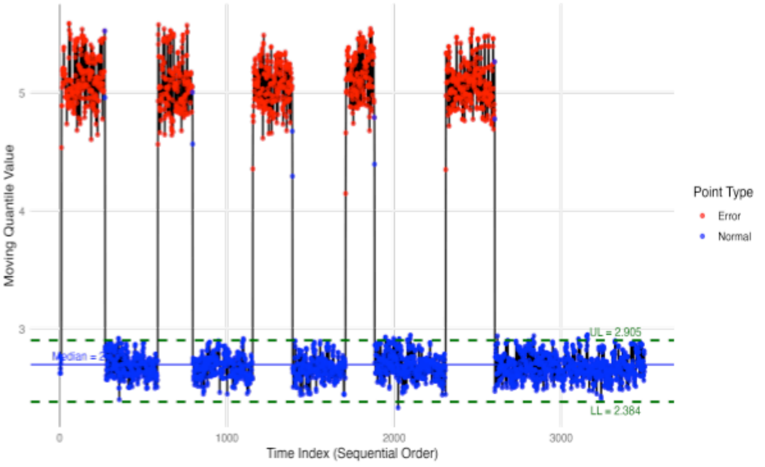

FT4_outputEWMA - Training (p1-p10)

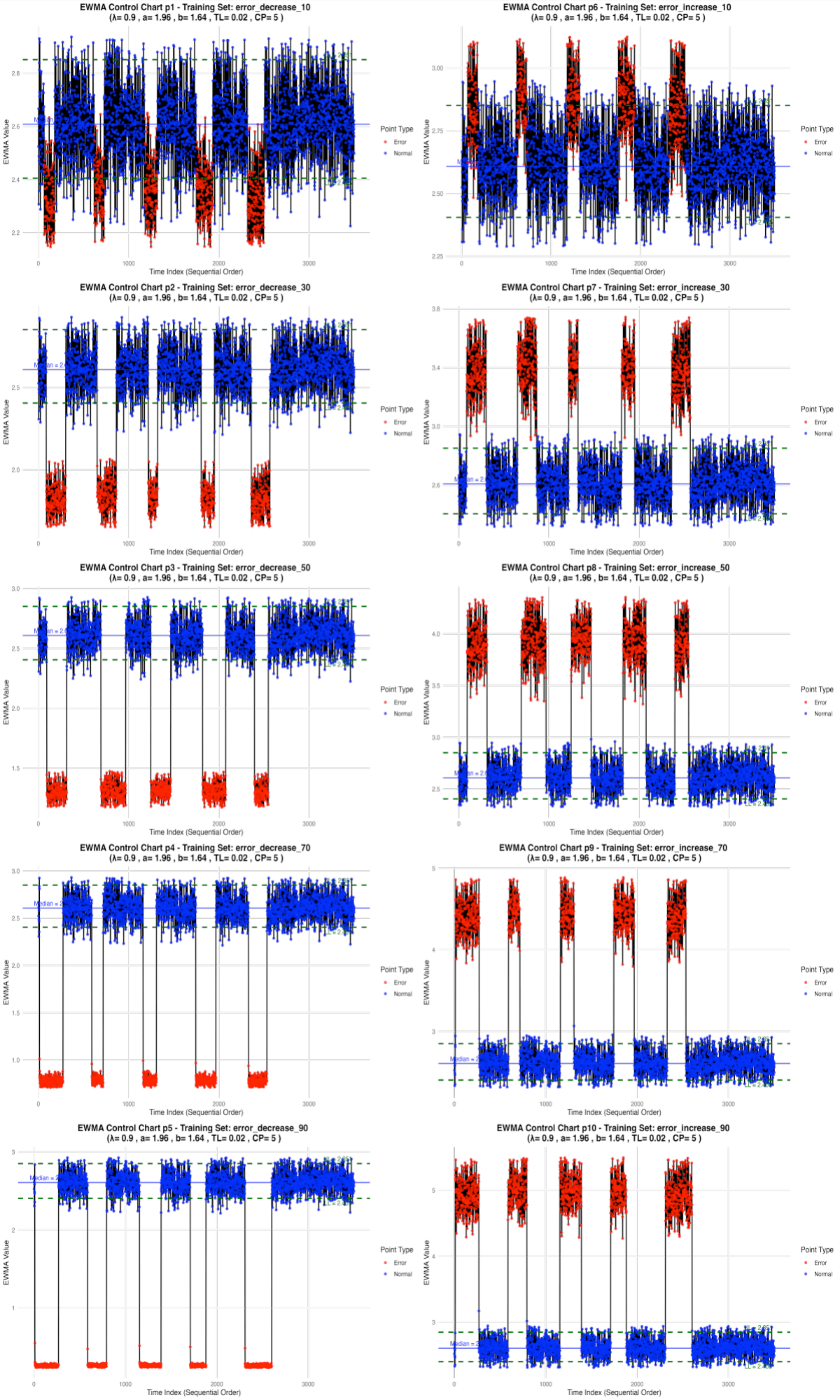

FT4_outputEWMA - Test (p11-p20)

EWMA Control Chart p11 - Test Set: error_decrease_10
(λ= 0.9 , a= 1.96 , b= 1.64 , TL= 0.02 , CP= 5)

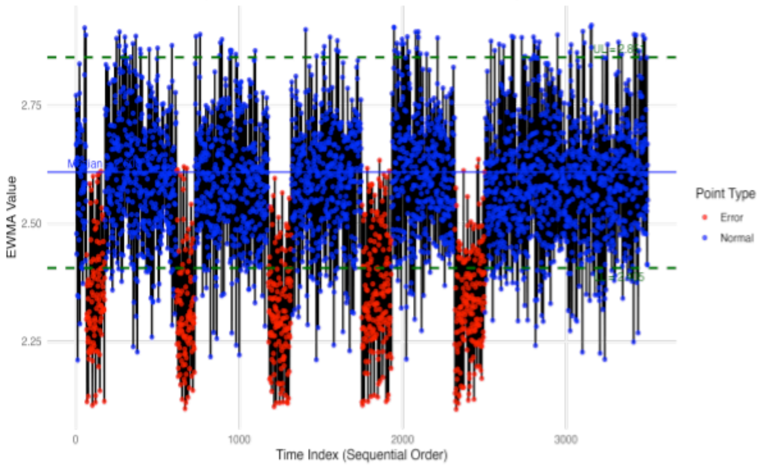

EWMA Control Chart p16 - Test Set: error_increase_10
(λ= 0.9 , a= 1.96 , b= 1.64 , TL= 0.02 , CP= 5)

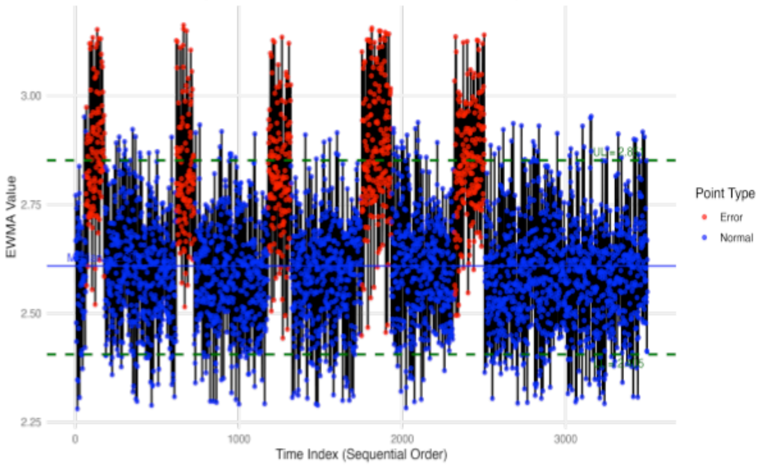

EWMA Control Chart p12 - Test Set: error_decrease_30
(λ= 0.9 , a= 1.96 , b= 1.64 , TL= 0.02 , CP= 5)

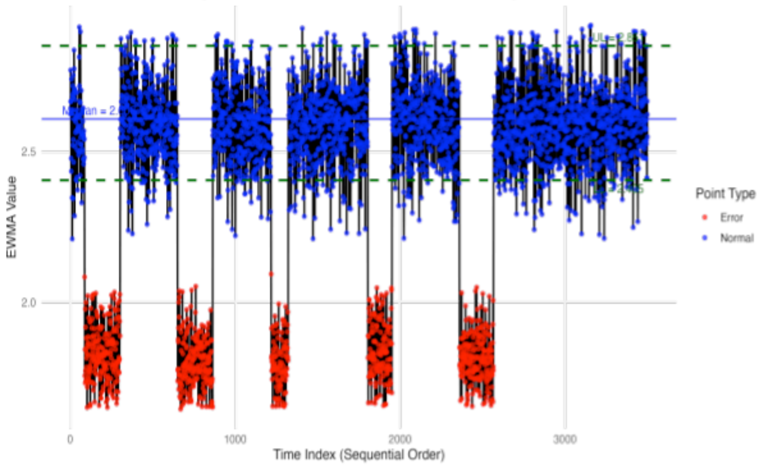

EWMA Control Chart p17 - Test Set: error_increase_30
(λ= 0.9 , a= 1.96 , b= 1.64 , TL= 0.02 , CP= 5)

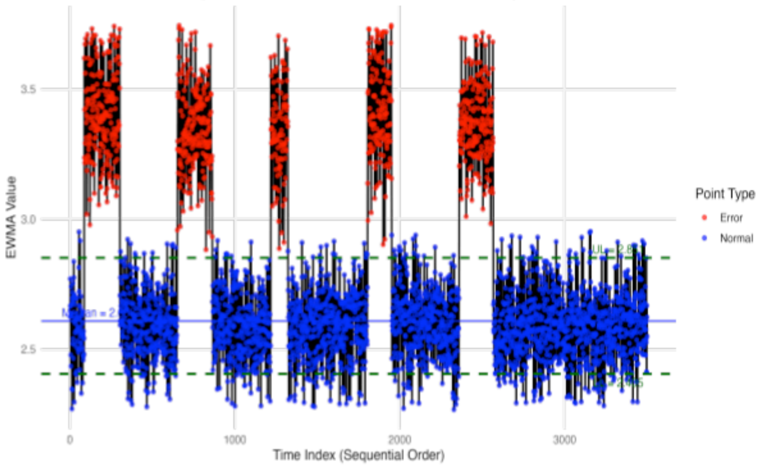

EWMA Control Chart p13 - Test Set: error_decrease_50
(λ= 0.9 , a= 1.96 , b= 1.64 , TL= 0.02 , CP= 5)

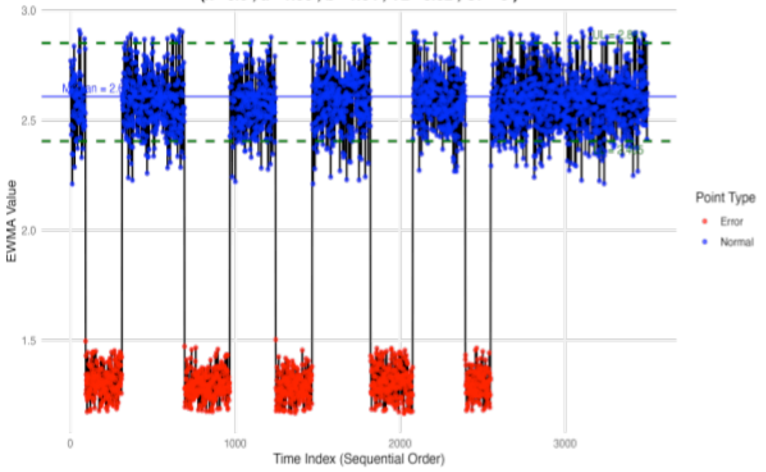

EWMA Control Chart p18 - Test Set: error_increase_50
(λ= 0.9 , a= 1.96 , b= 1.64 , TL= 0.02 , CP= 5)

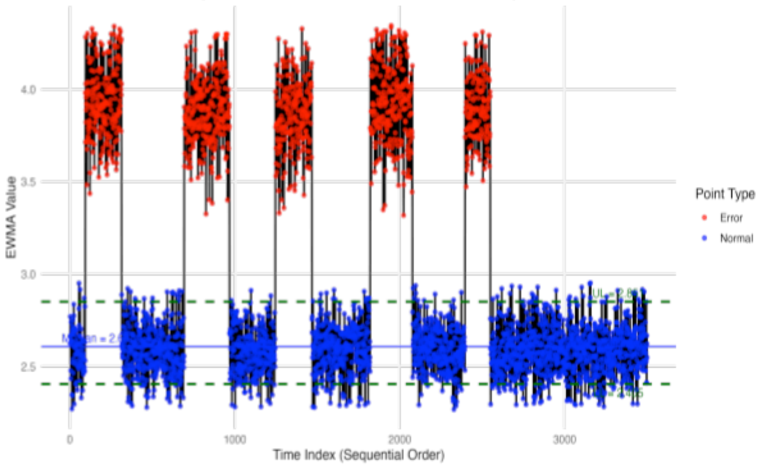

EWMA Control Chart p14 - Test Set: error_decrease_70
(λ= 0.9 , a= 1.96 , b= 1.64 , TL= 0.02 , CP= 5)

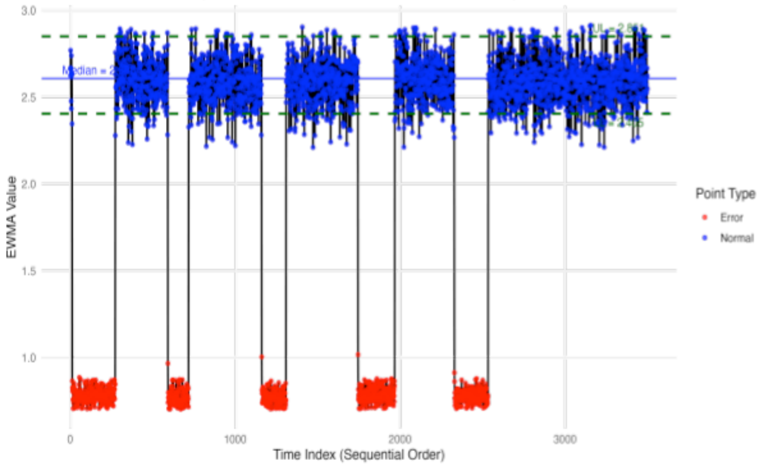

EWMA Control Chart p19 - Test Set: error_increase_70
(λ= 0.9 , a= 1.96 , b= 1.64 , TL= 0.02 , CP= 5)

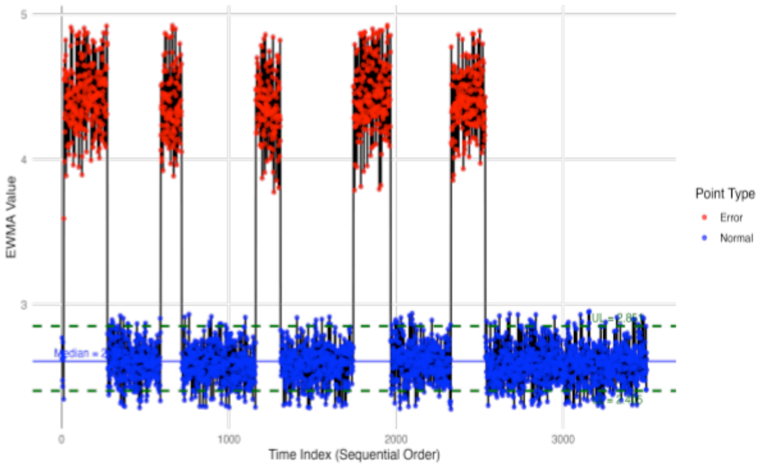

EWMA Control Chart p15 - Test Set: error_decrease_90
(λ= 0.9 , a= 1.96 , b= 1.64 , TL= 0.02 , CP= 5)

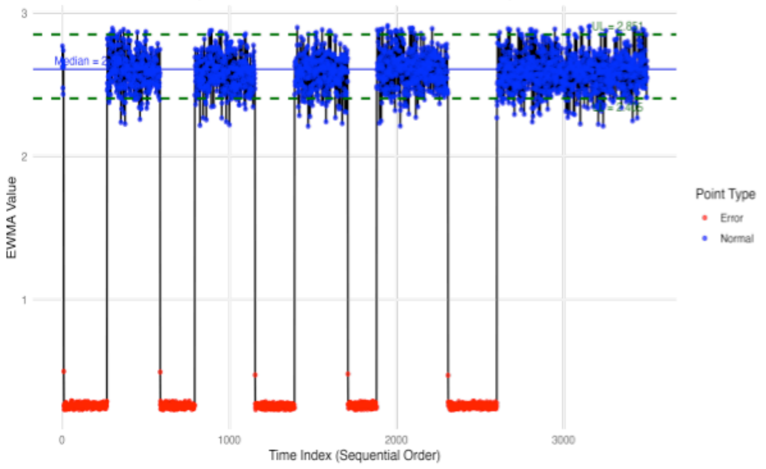

EWMA Control Chart p20 - Test Set: error_increase_90
(λ= 0.9 , a= 1.96 , b= 1.64 , TL= 0.02 , CP= 5)

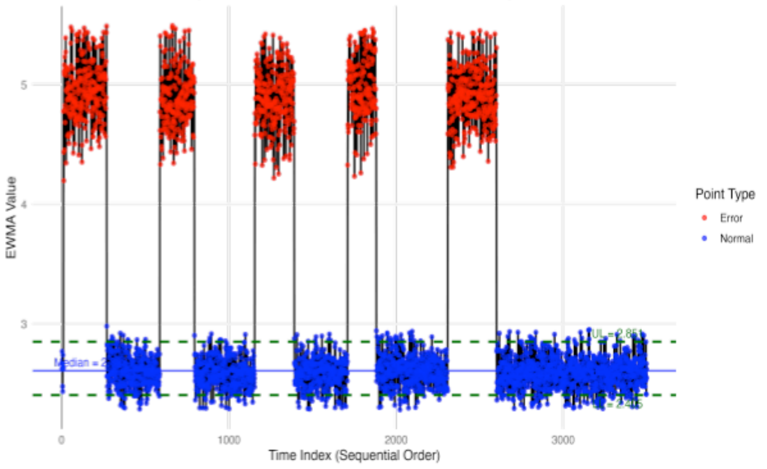

FT4_outputMA - Training (p1-p10)

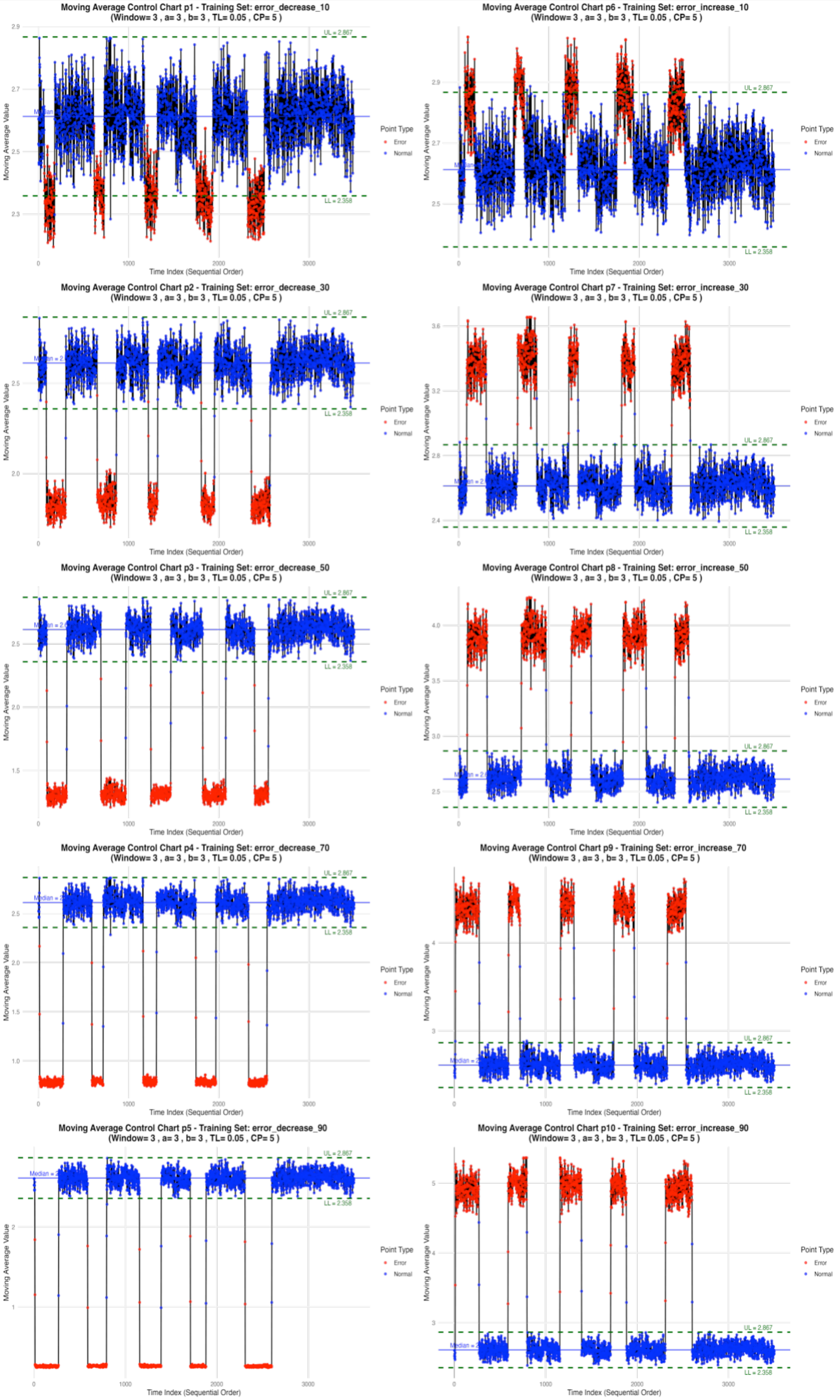

FT4_outputMA - Test (p11-p20)

Moving Average Control Chart p11 - Test Set: error_decrease_10
(Window= 3 , a= 3 , b= 3 , TL= 0.05 , CP= 5)

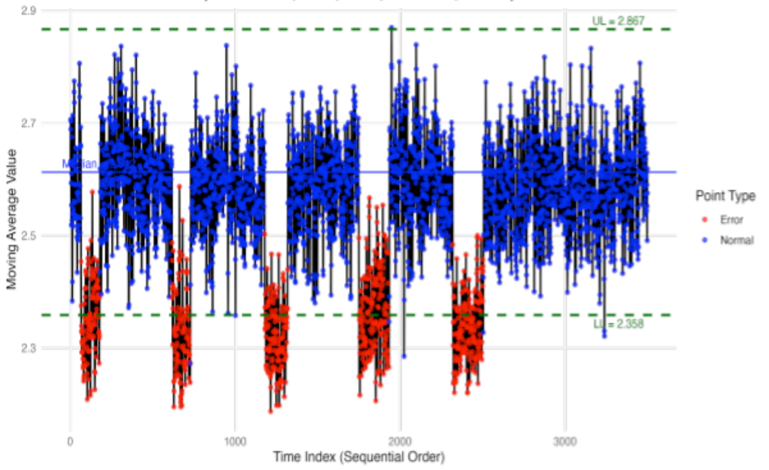

Moving Average Control Chart p16 - Test Set: error_increase_10
(Window= 3 , a= 3 , b= 3 , TL= 0.05 , CP= 5)

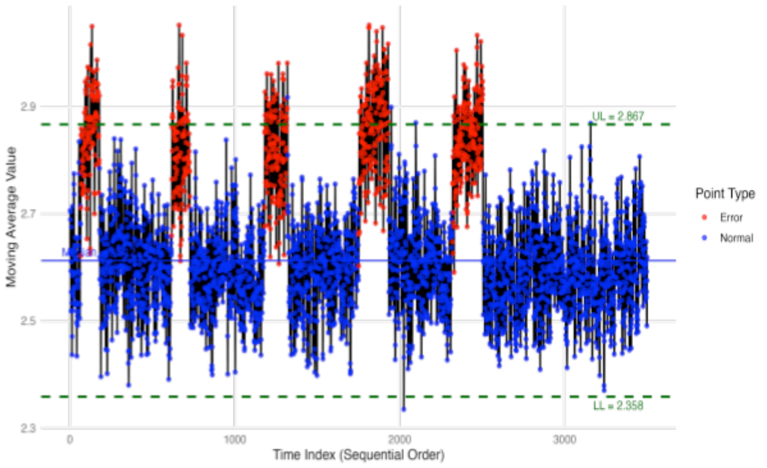

Moving Average Control Chart p12 - Test Set: error_decrease_30
(Window= 3 , a= 3 , b= 3 , TL= 0.05 , CP= 5)

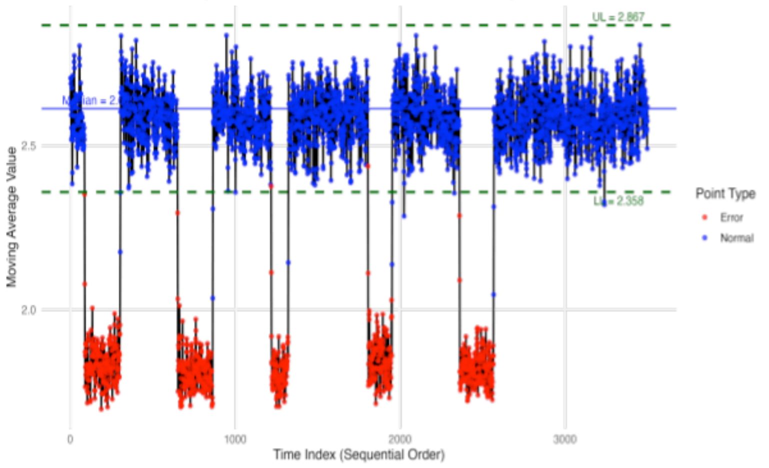

Moving Average Control Chart p17 - Test Set: error_increase_30
(Window= 3 , a= 3 , b= 3 , TL= 0.05 , CP= 5)

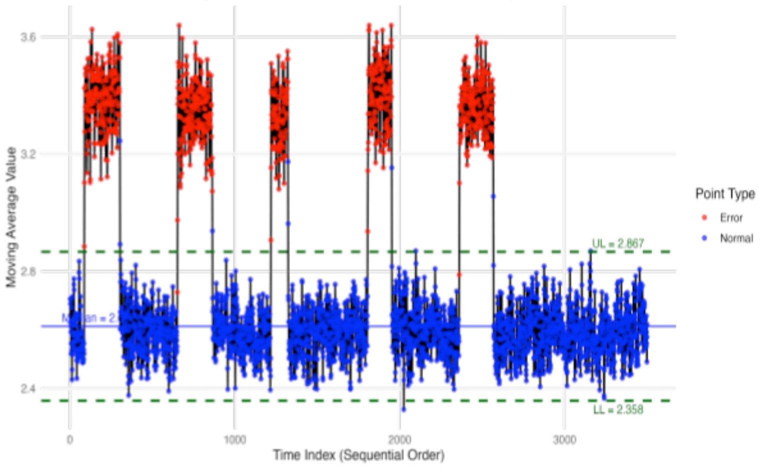

Moving Average Control Chart p13 - Test Set: error_decrease_50
(Window= 3 , a= 3 , b= 3 , TL= 0.05 , CP= 5)

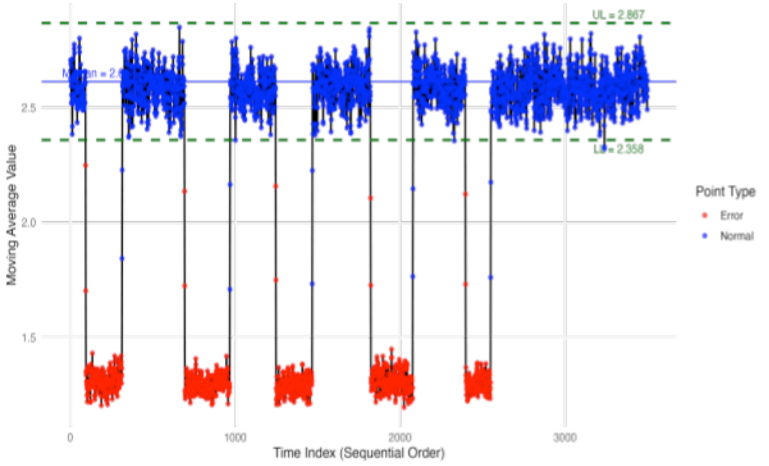

Moving Average Control Chart p18 - Test Set: error_increase_50
(Window= 3 , a= 3 , b= 3 , TL= 0.05 , CP= 5)

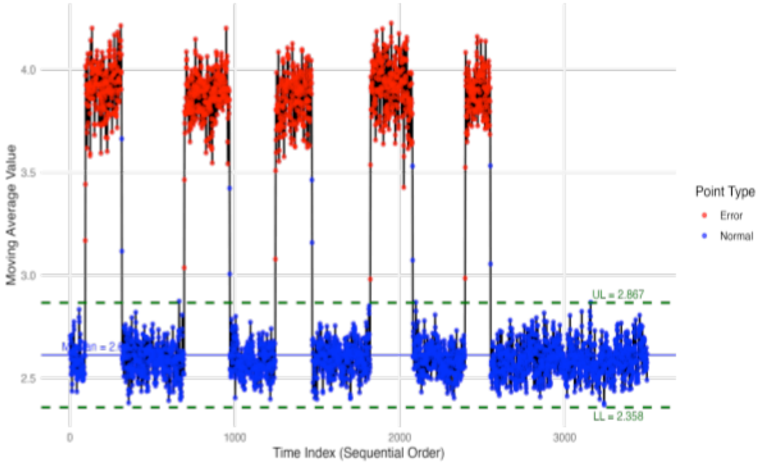

Moving Average Control Chart p14 - Test Set: error_decrease_70
(Window= 3 , a= 3 , b= 3 , TL= 0.05 , CP= 5)

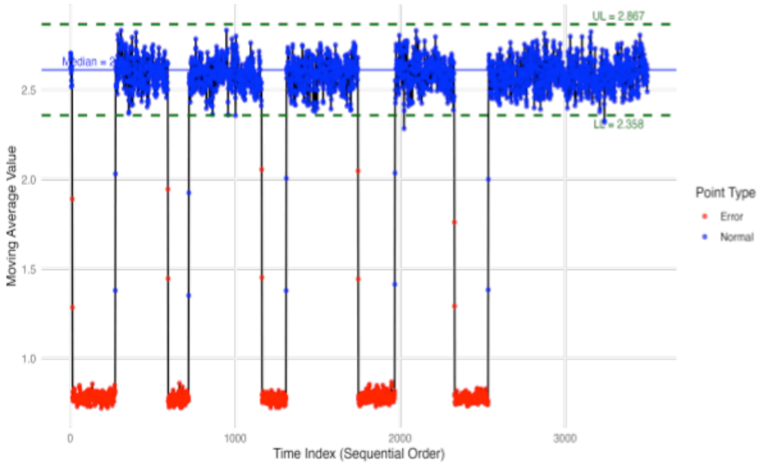

Moving Average Control Chart p19 - Test Set: error_increase_70
(Window= 3 , a= 3 , b= 3 , TL= 0.05 , CP= 5)

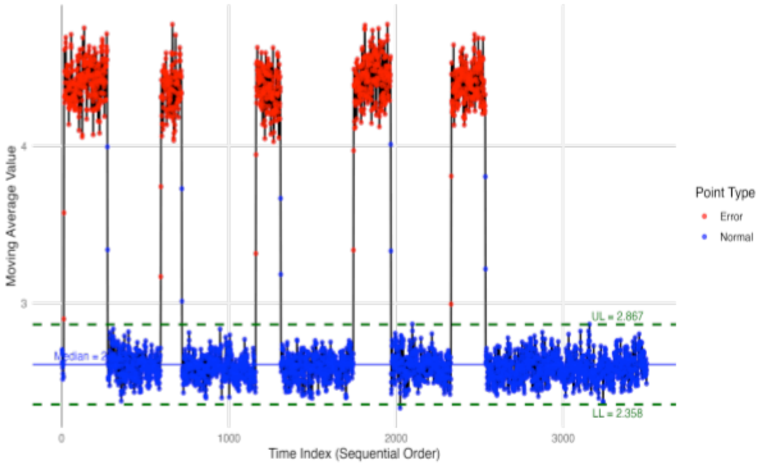

Moving Average Control Chart p15 - Test Set: error_decrease_90
(Window= 3 , a= 3 , b= 3 , TL= 0.05 , CP= 5)

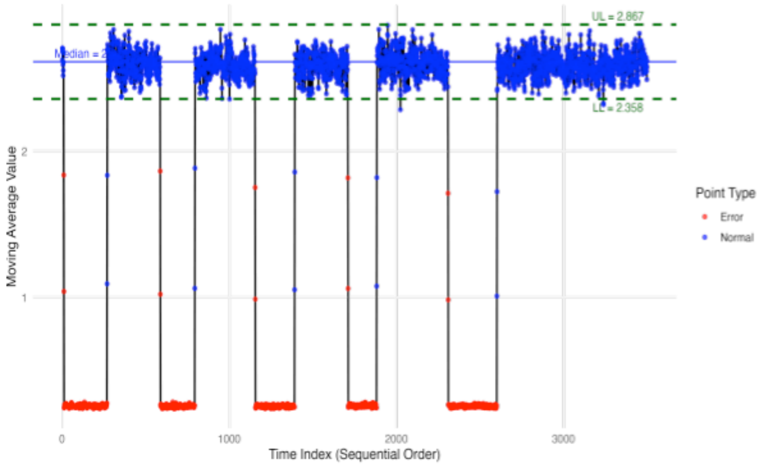

Moving Average Control Chart p20 - Test Set: error_increase_90
(Window= 3 , a= 3 , b= 3 , TL= 0.05 , CP= 5)

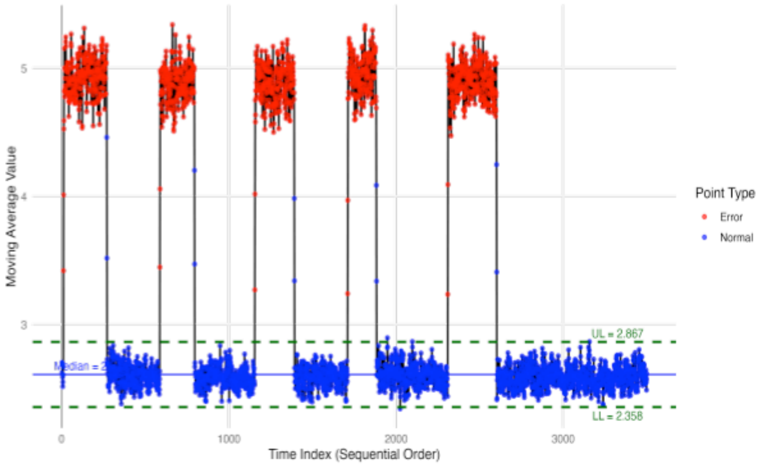

PT_outputMQ - Training (p1-p10)

Moving Quantile Control Chart p1 - Training Set: error_decrease_10
(Window= 3 , QL= 0.9 , a= 1.64 , b= 3 , TL= 0 , CP= 10)

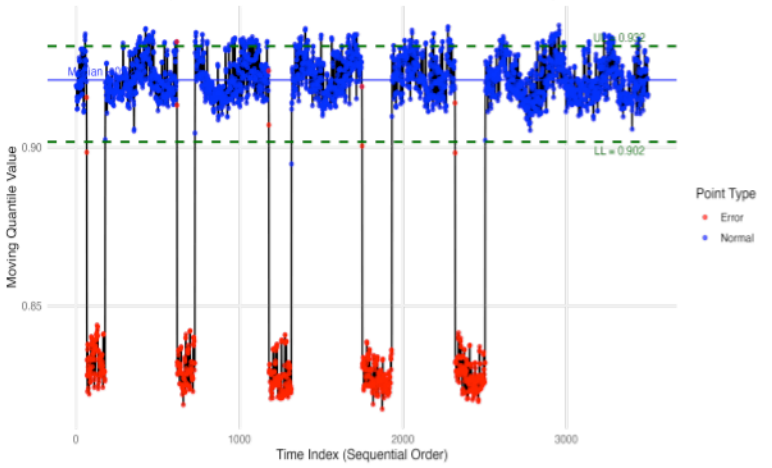

Moving Quantile Control Chart p2 - Training Set: error_decrease_30
(Window= 3 , QL= 0.9 , a= 1.64 , b= 3 , TL= 0 , CP= 10)

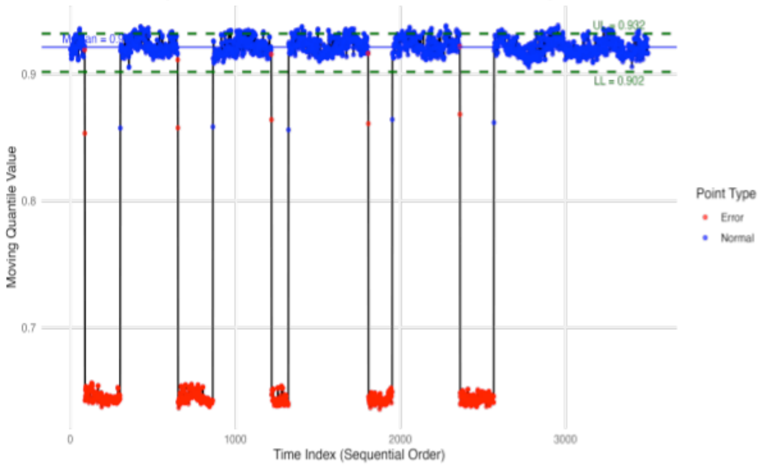

Moving Quantile Control Chart p3 - Training Set: error_decrease_50
(Window= 3 , QL= 0.9 , a= 1.64 , b= 3 , TL= 0 , CP= 10)

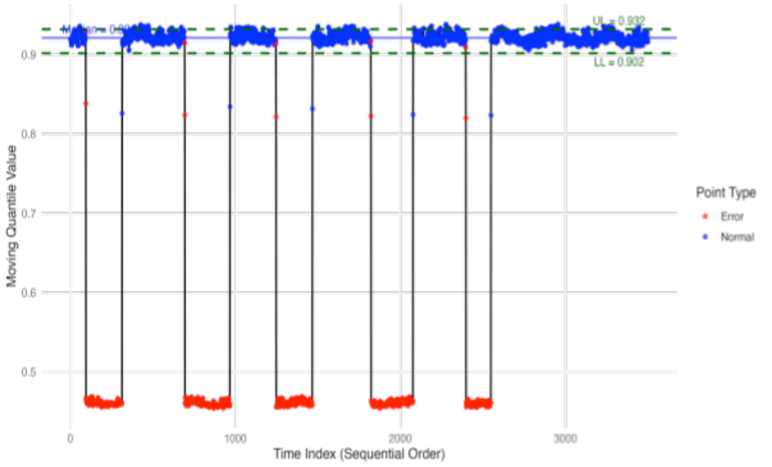

Moving Quantile Control Chart p4 - Training Set: error_decrease_70
(Window= 3 , QL= 0.9 , a= 1.64 , b= 3 , TL= 0 , CP= 10)

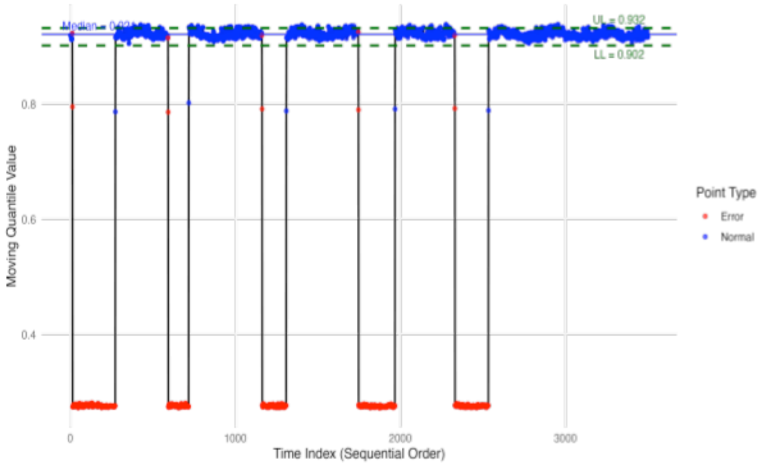

Moving Quantile Control Chart p5 - Training Set: error_decrease_90
(Window= 3 , QL= 0.9 , a= 1.64 , b= 3 , TL= 0 , CP= 10)

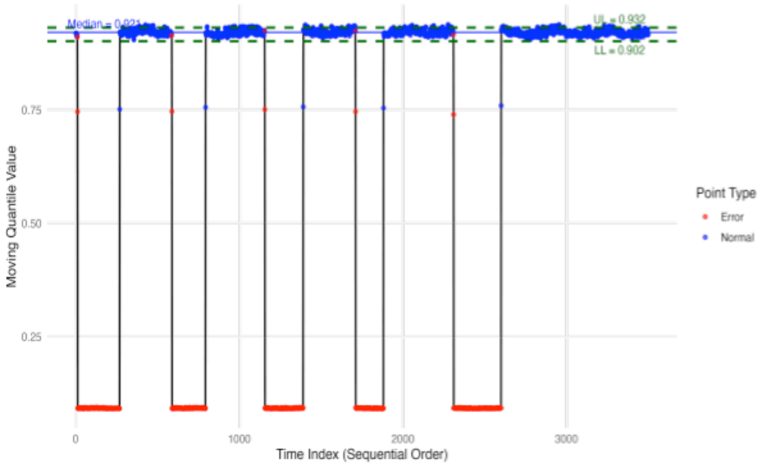

Moving Quantile Control Chart p6 - Training Set: error_increase_10
(Window= 3 , QL= 0.9 , a= 1.64 , b= 3 , TL= 0 , CP= 10)

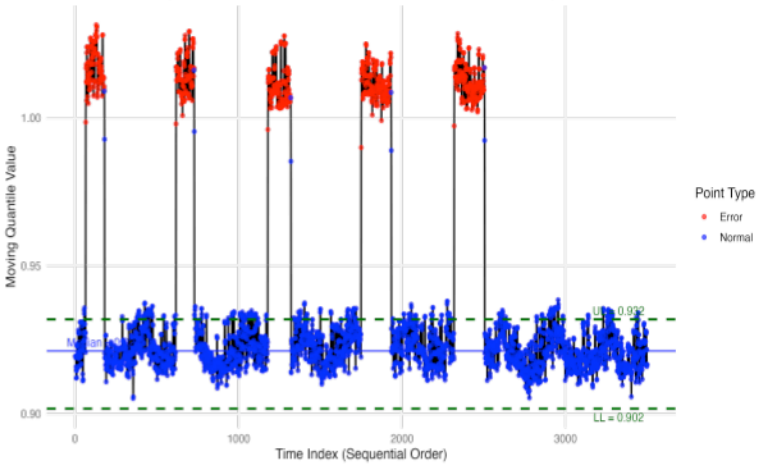

Moving Quantile Control Chart p7 - Training Set: error_increase_30
(Window= 3 , QL= 0.9 , a= 1.64 , b= 3 , TL= 0 , CP= 10)

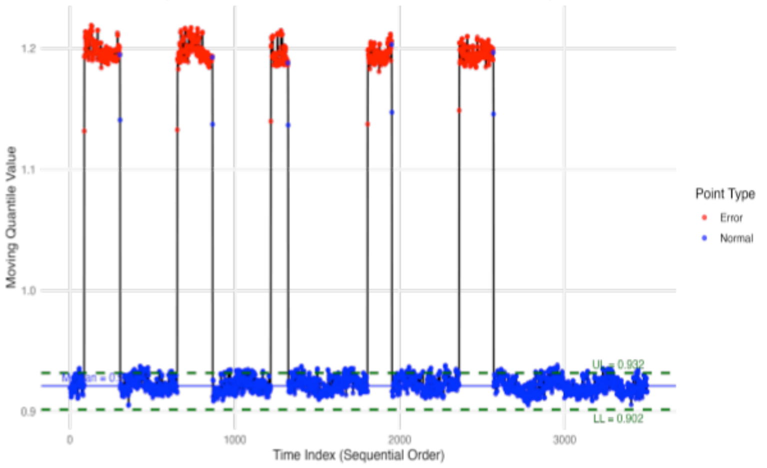

Moving Quantile Control Chart p8 - Training Set: error_increase_50
(Window= 3 , QL= 0.9 , a= 1.64 , b= 3 , TL= 0 , CP= 10)

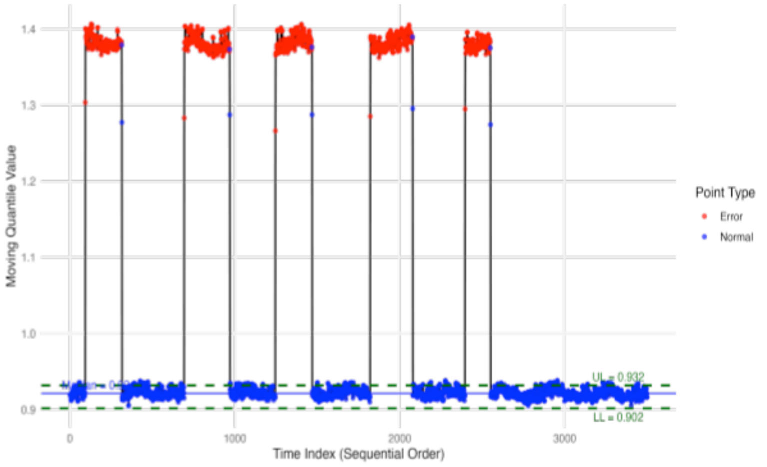

Moving Quantile Control Chart p9 - Training Set: error_increase_70
(Window= 3 , QL= 0.9 , a= 1.64 , b= 3 , TL= 0 , CP= 10)

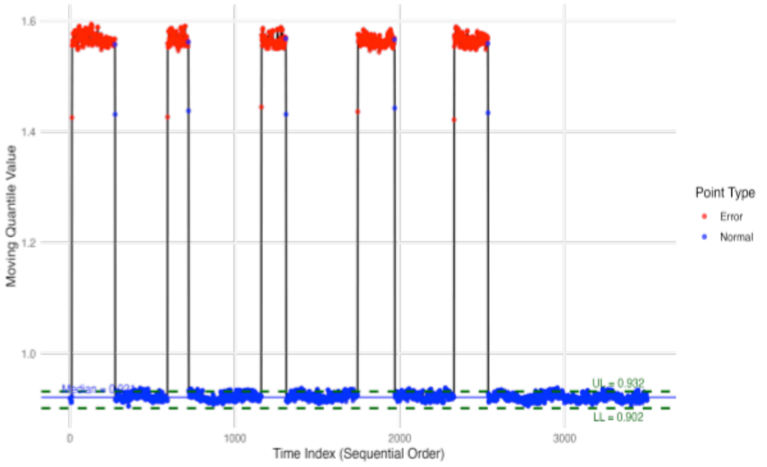

Moving Quantile Control Chart p10 - Training Set: error_increase_90
(Window= 3 , QL= 0.9 , a= 1.64 , b= 3 , TL= 0 , CP= 10)

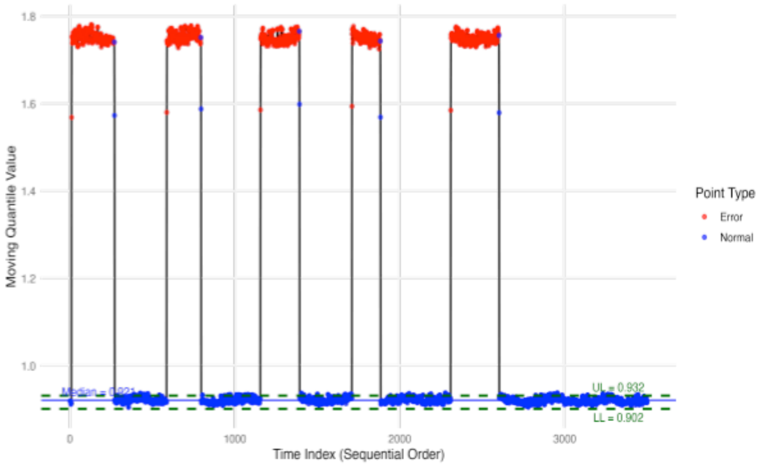

PT_outputMQ - Test (p11-p20)

Moving Quantile Control Chart p11 - Test Set: error_decrease_10
(Window= 3 , QL= 0.9 , a= 1.64 , b= 3 , TL= 0 , CP= 10)

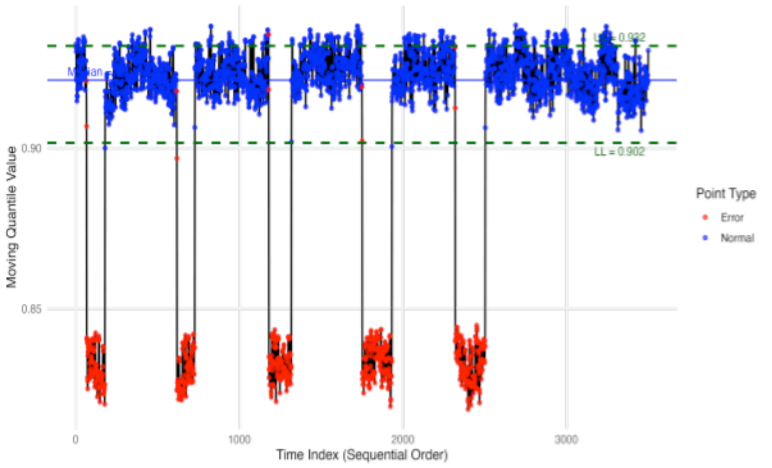

Moving Quantile Control Chart p16 - Test Set: error_increase_10
(Window= 3 , QL= 0.9 , a= 1.64 , b= 3 , TL= 0 , CP= 10)

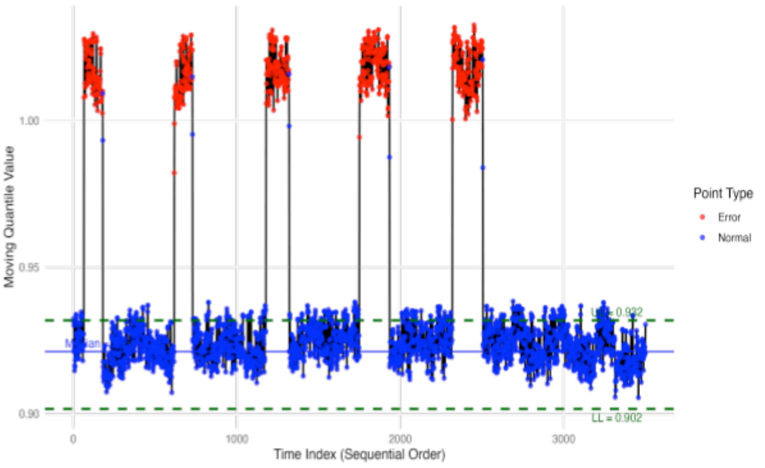

Moving Quantile Control Chart p12 - Test Set: error_decrease_30
(Window= 3 , QL= 0.9 , a= 1.64 , b= 3 , TL= 0 , CP= 10)

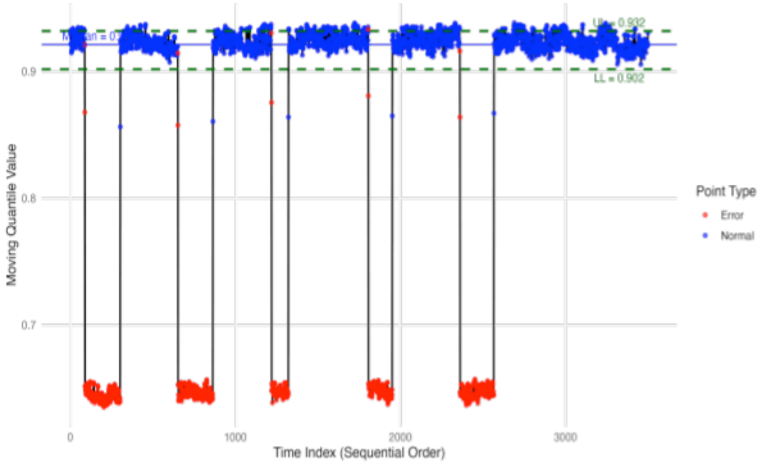

Moving Quantile Control Chart p17 - Test Set: error_increase_30
(Window= 3 , QL= 0.9 , a= 1.64 , b= 3 , TL= 0 , CP= 10)

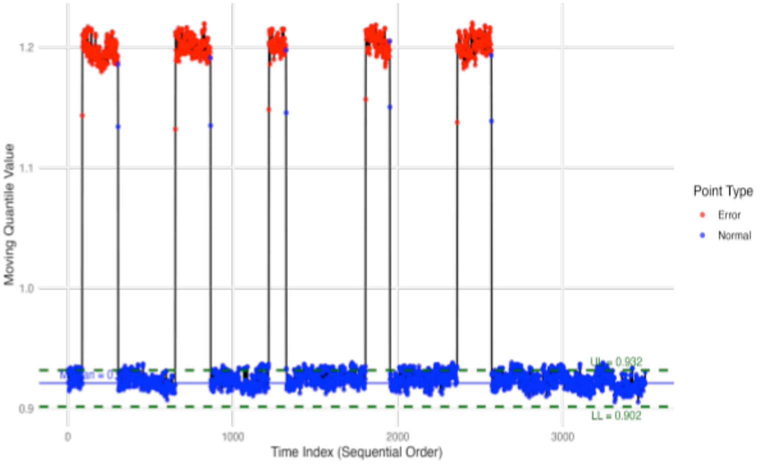

Moving Quantile Control Chart p13 - Test Set: error_decrease_50
(Window= 3 , QL= 0.9 , a= 1.64 , b= 3 , TL= 0 , CP= 10)

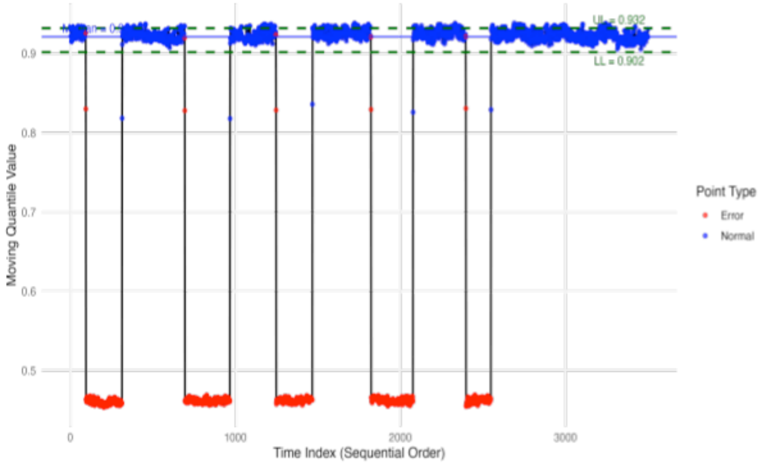

Moving Quantile Control Chart p18 - Test Set: error_increase_50
(Window= 3 , QL= 0.9 , a= 1.64 , b= 3 , TL= 0 , CP= 10)

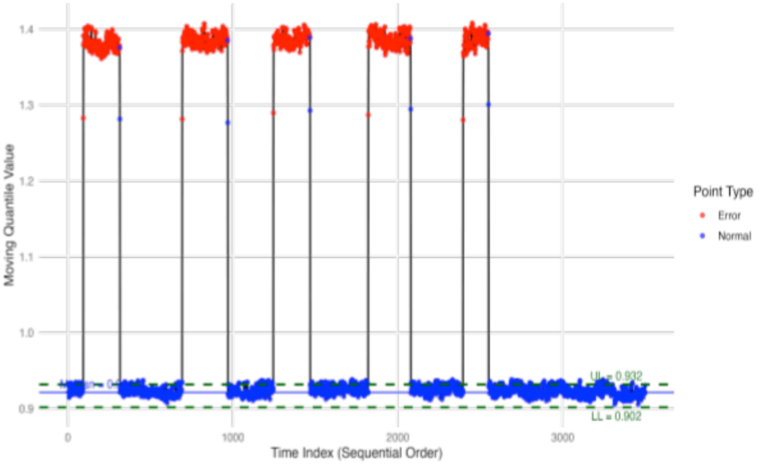

Moving Quantile Control Chart p14 - Test Set: error_decrease_70
(Window= 3 , QL= 0.9 , a= 1.64 , b= 3 , TL= 0 , CP= 10)

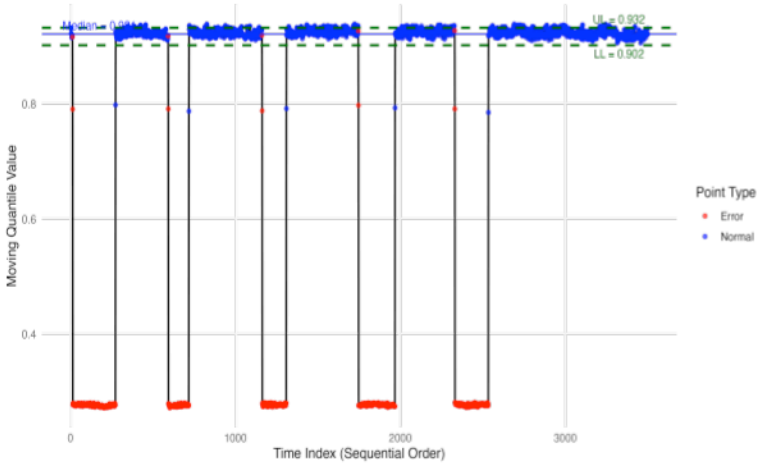

Moving Quantile Control Chart p19 - Test Set: error_increase_70
(Window= 3 , QL= 0.9 , a= 1.64 , b= 3 , TL= 0 , CP= 10)

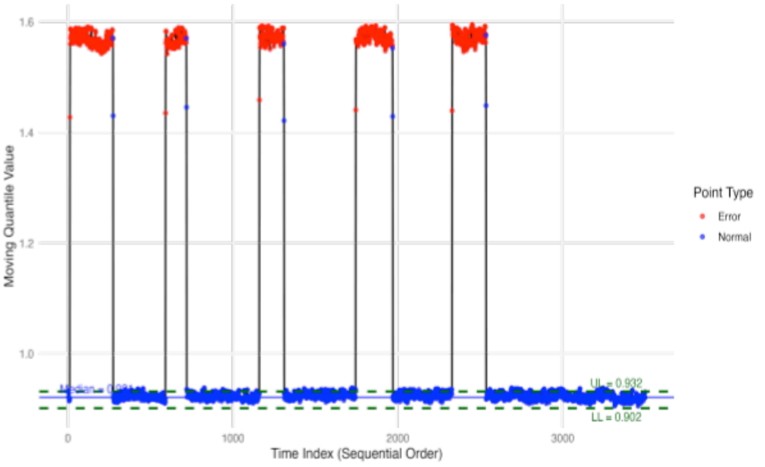

Moving Quantile Control Chart p15 - Test Set: error_decrease_90
(Window= 3 , QL= 0.9 , a= 1.64 , b= 3 , TL= 0 , CP= 10)

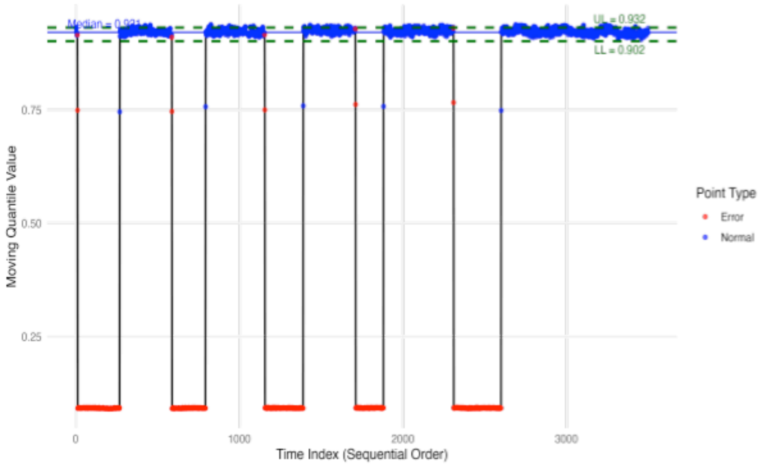

Moving Quantile Control Chart p20 - Test Set: error_increase_90
(Window= 3 , QL= 0.9 , a= 1.64 , b= 3 , TL= 0 , CP= 10)

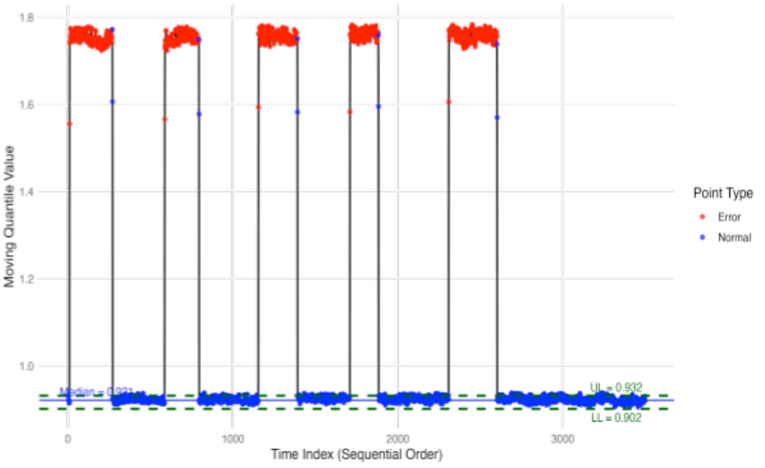

PT_outputEWMA - Training (p1-p10)

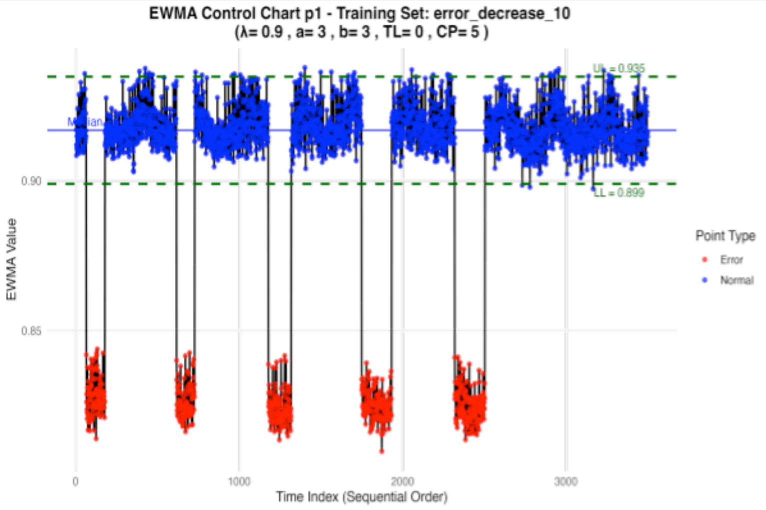

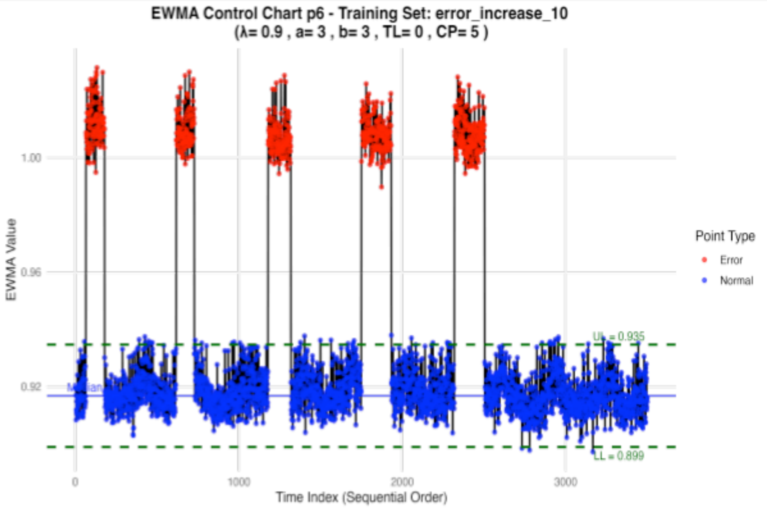

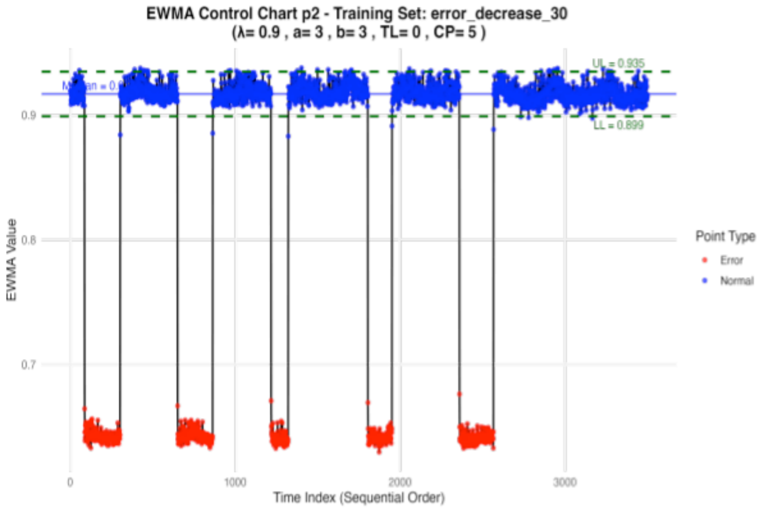

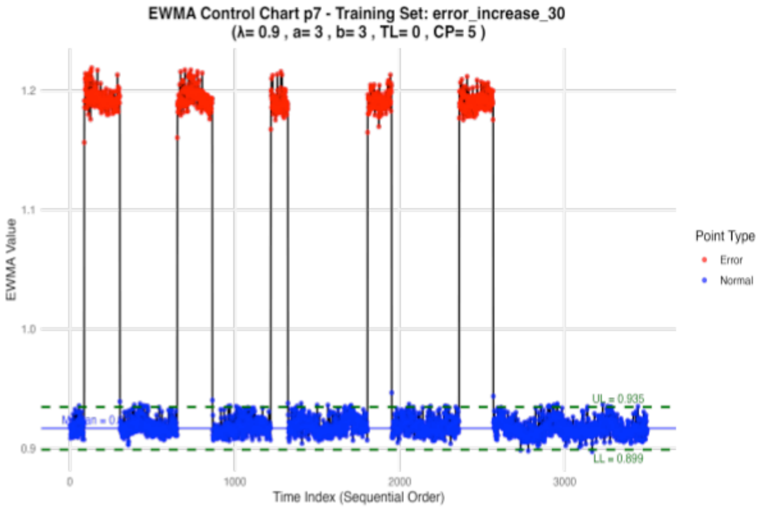

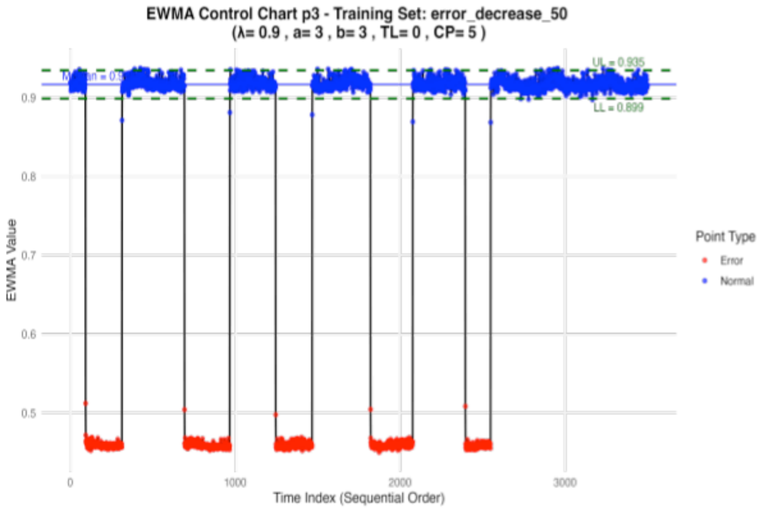

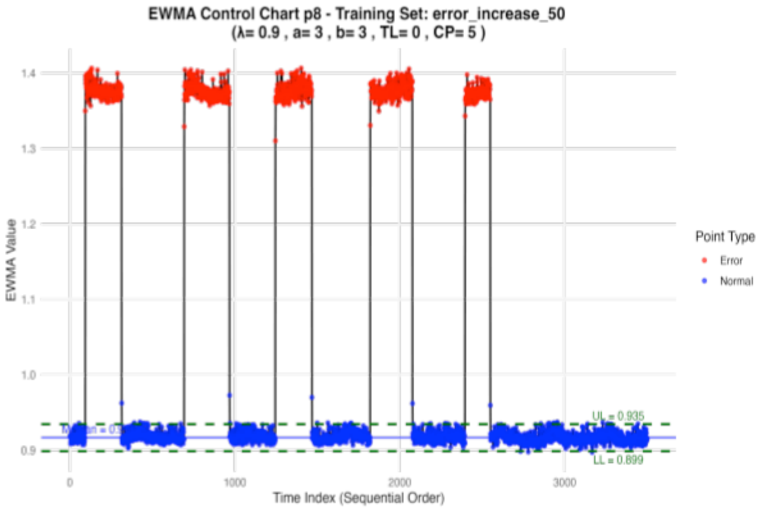

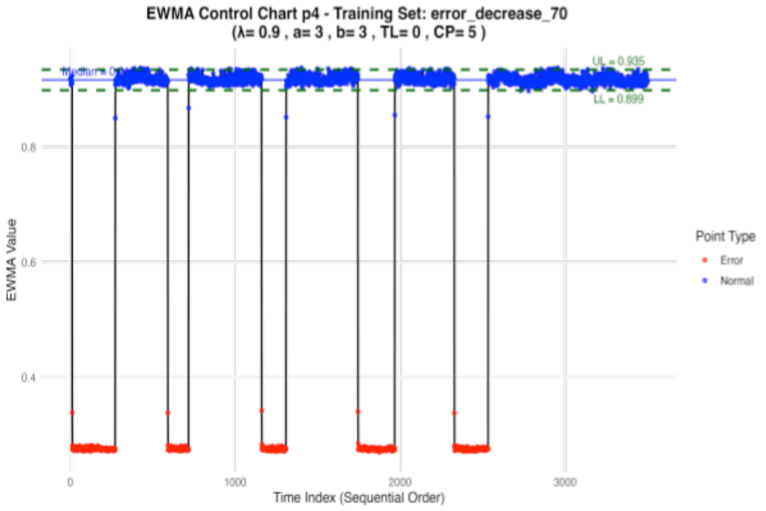

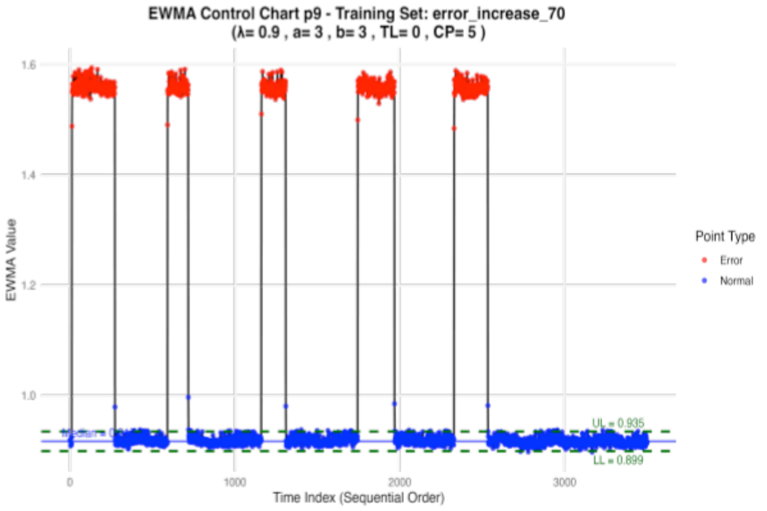

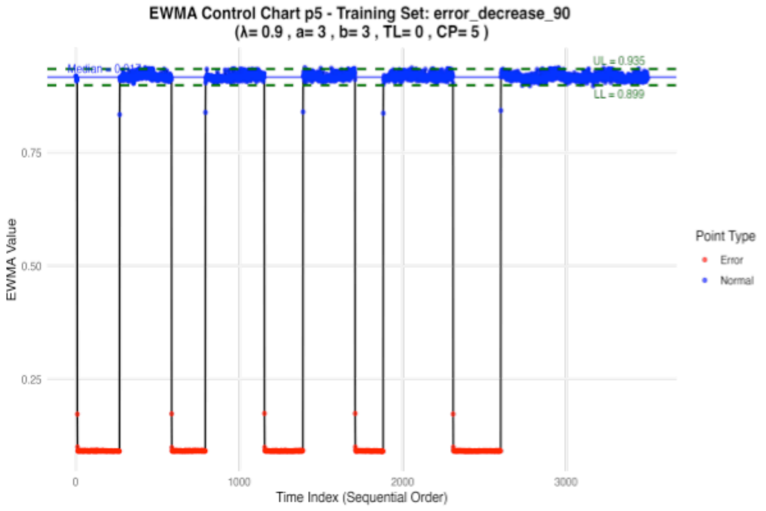

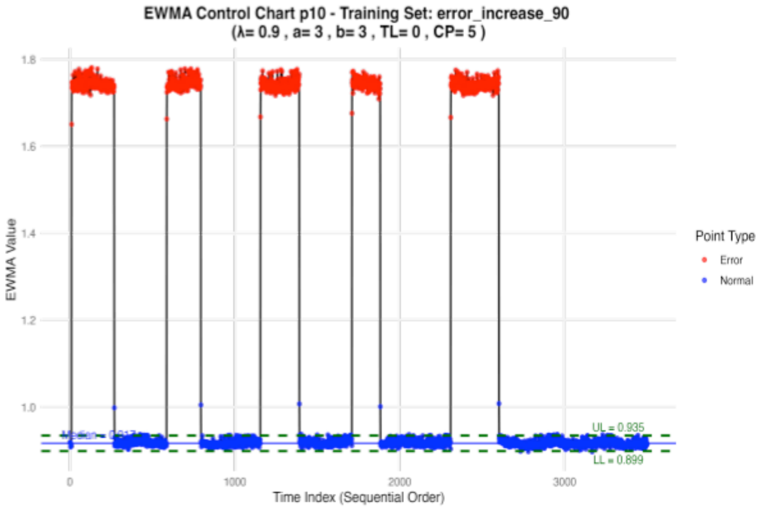

PT_outputEWMA - Test (p11-p20)

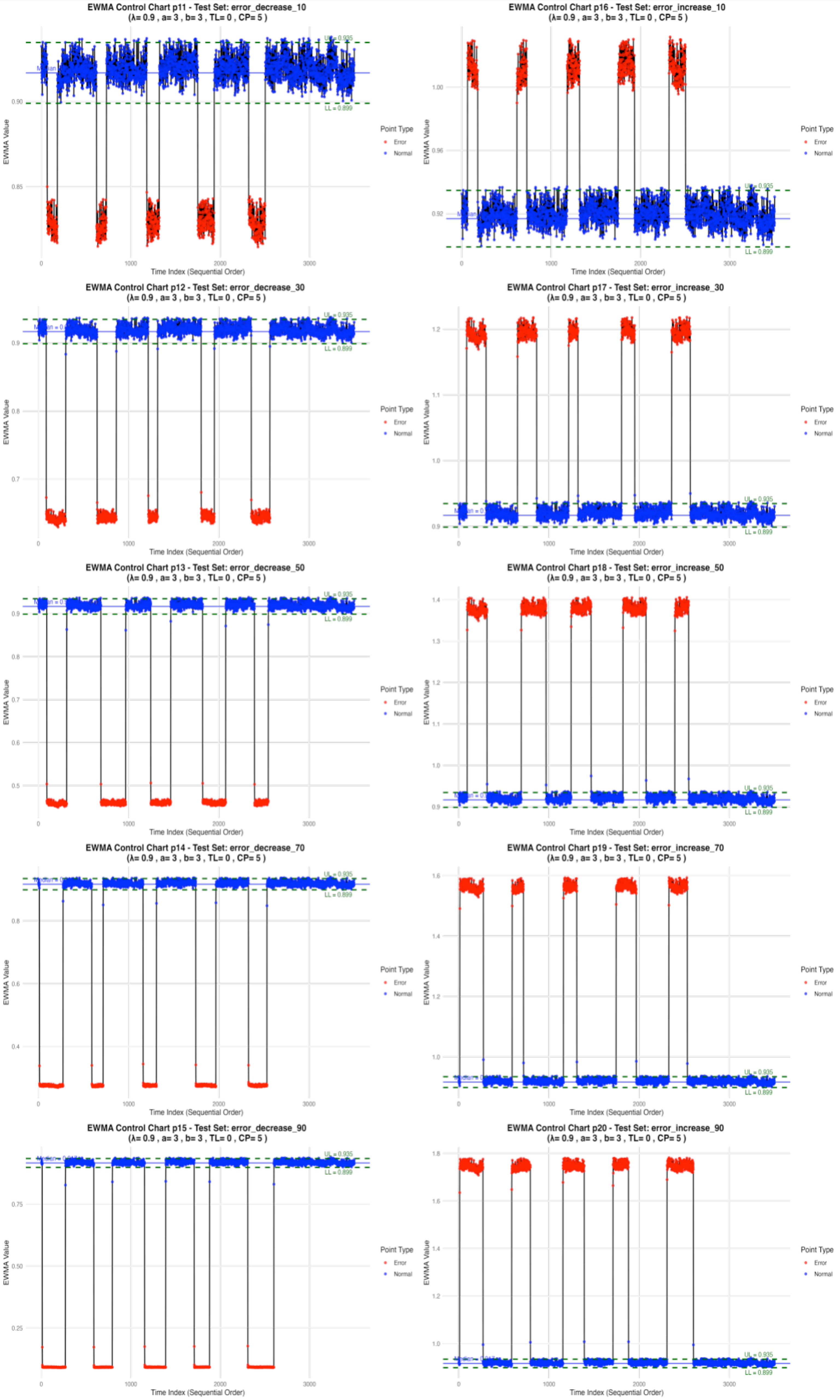

PT_outputMA - Training (p1-p10)

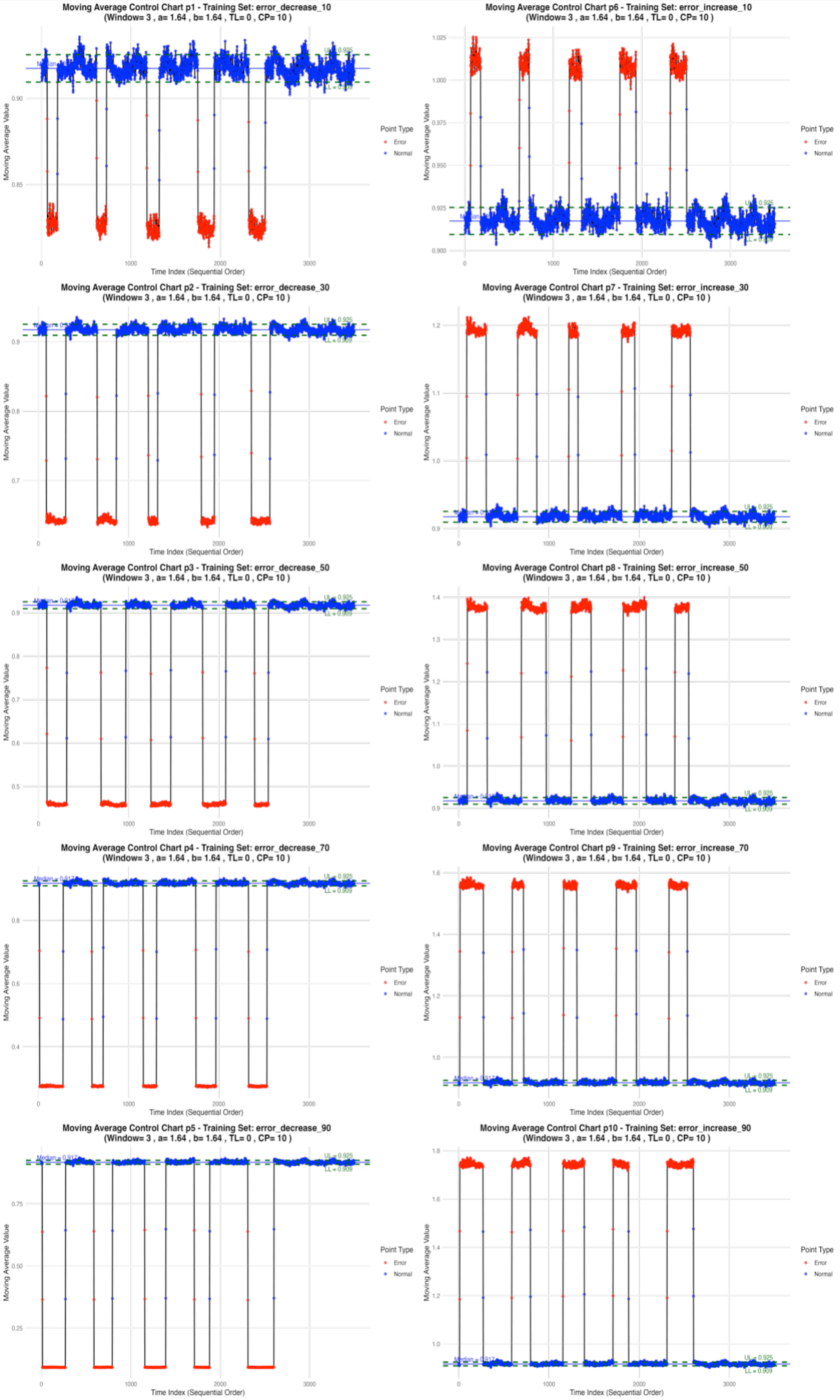

PT_outputMA - Test (p11-p20)

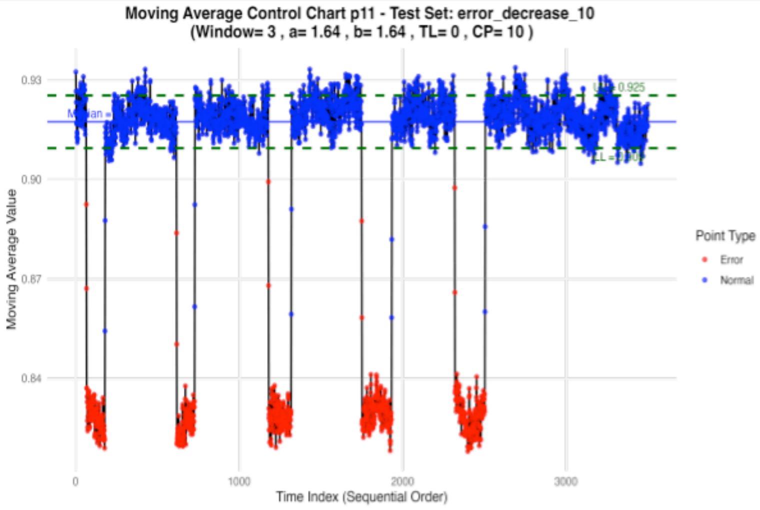

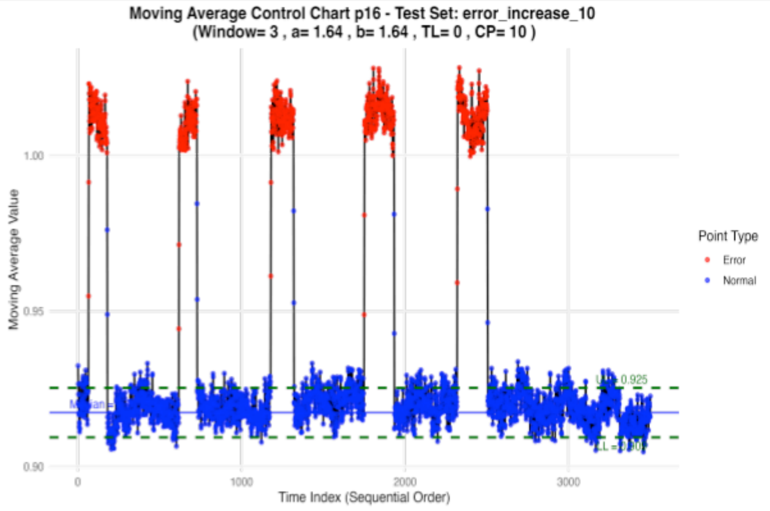

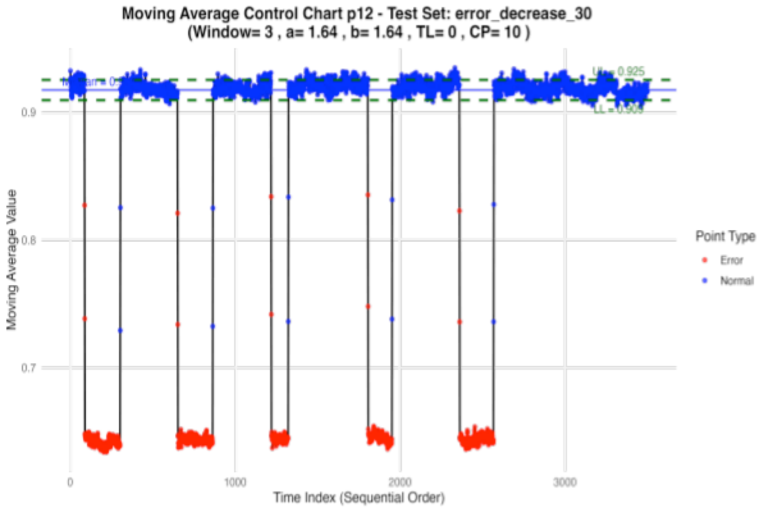

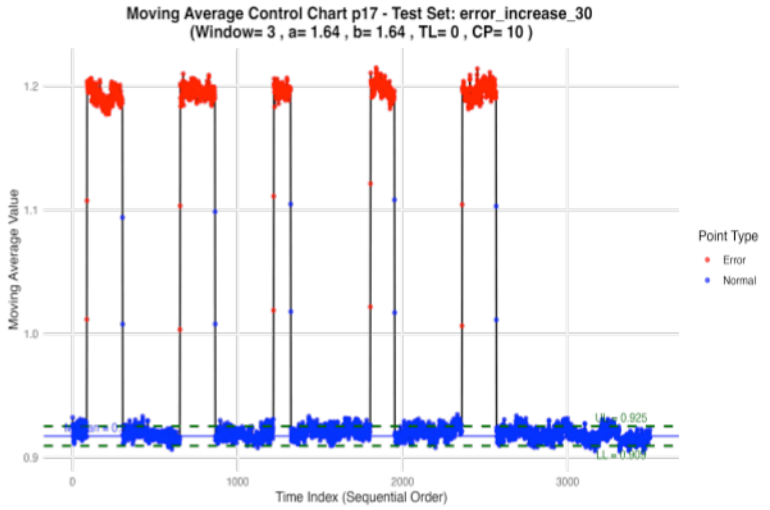

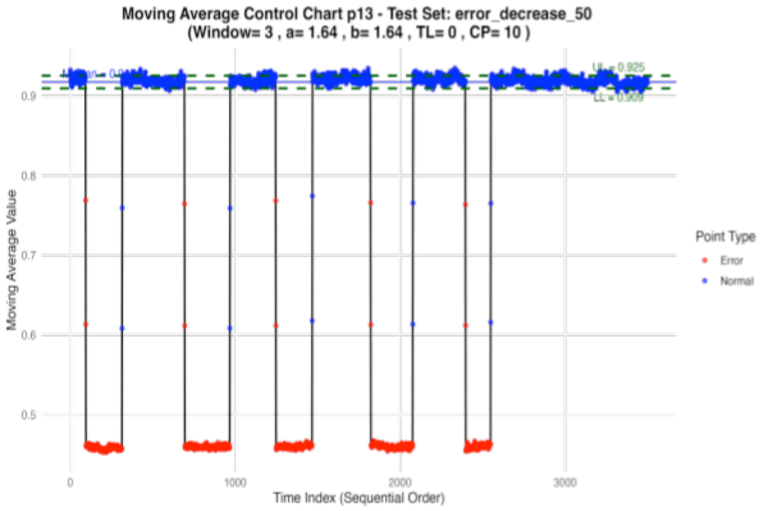

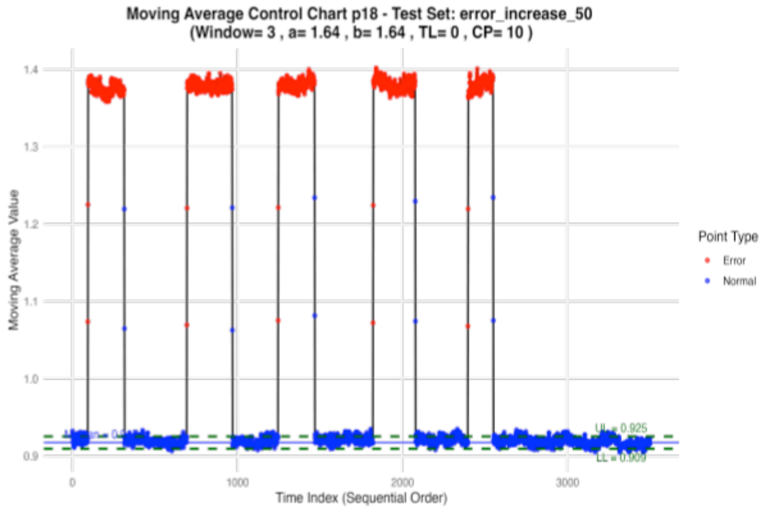

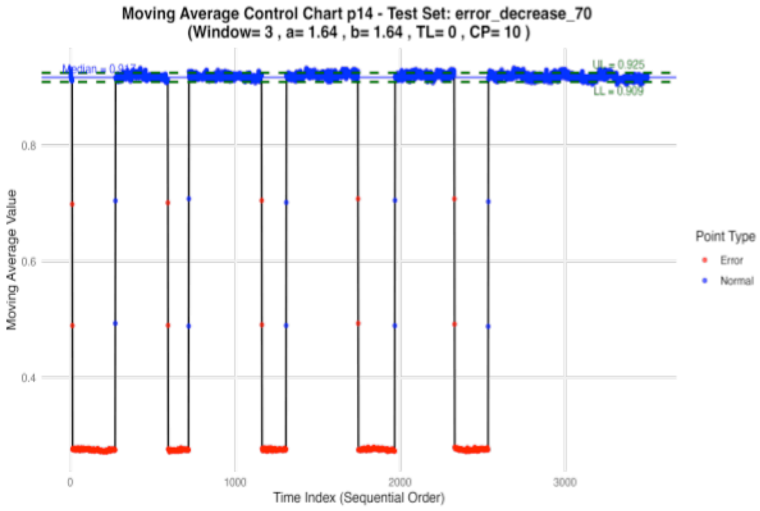

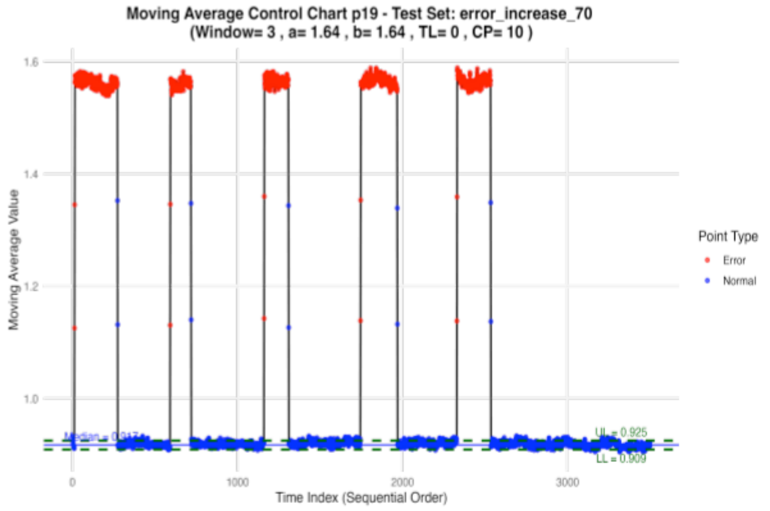

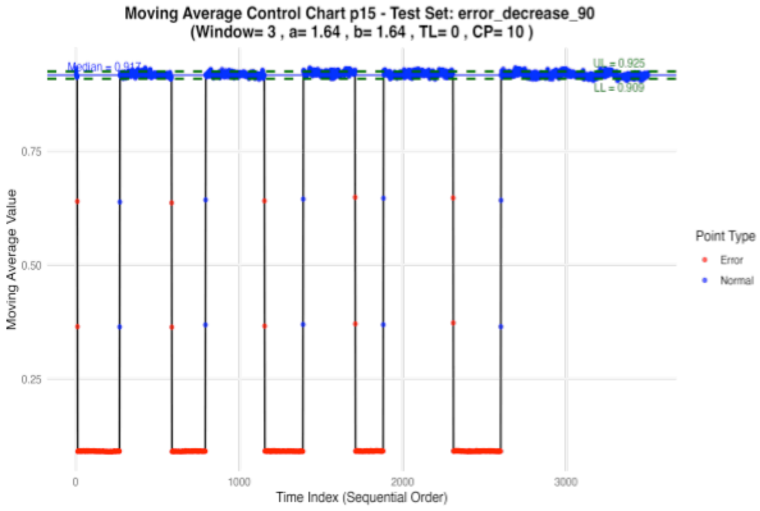

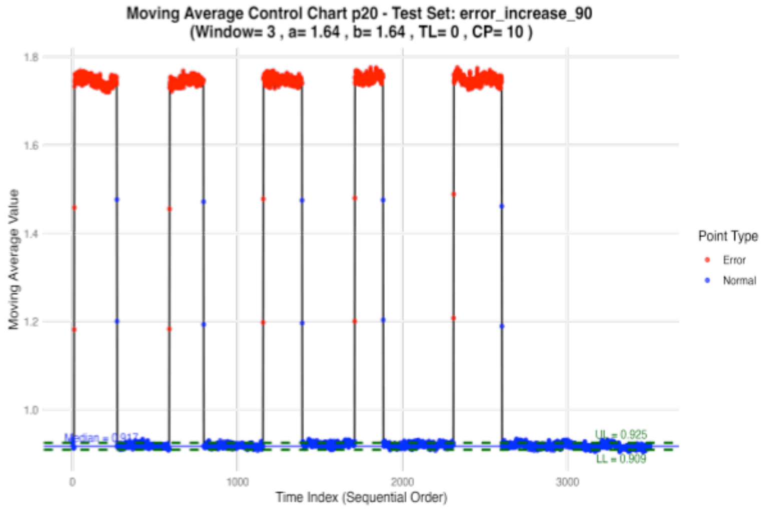

APTT_outputMQ - Training (p1-p10)

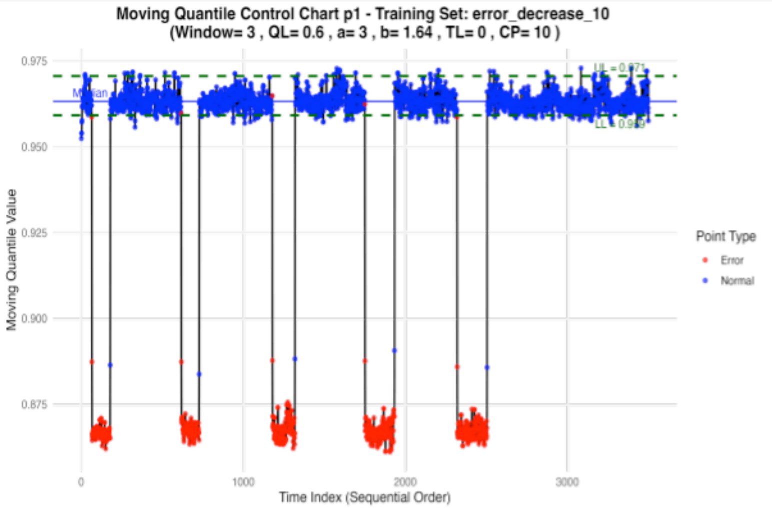

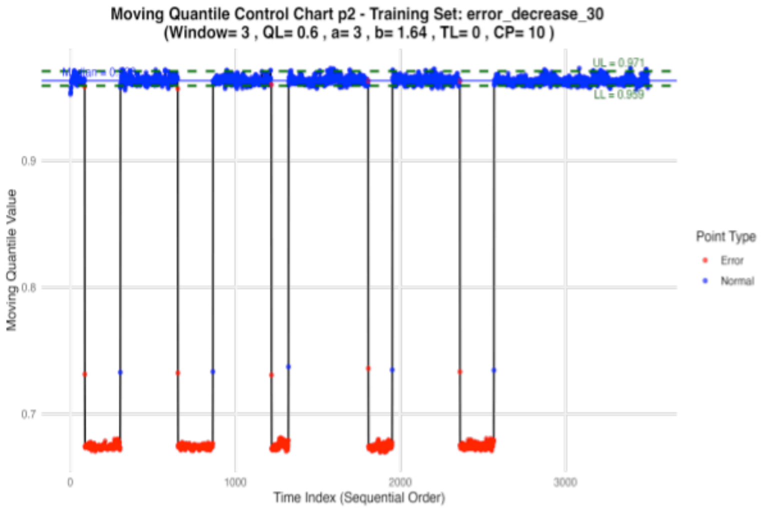

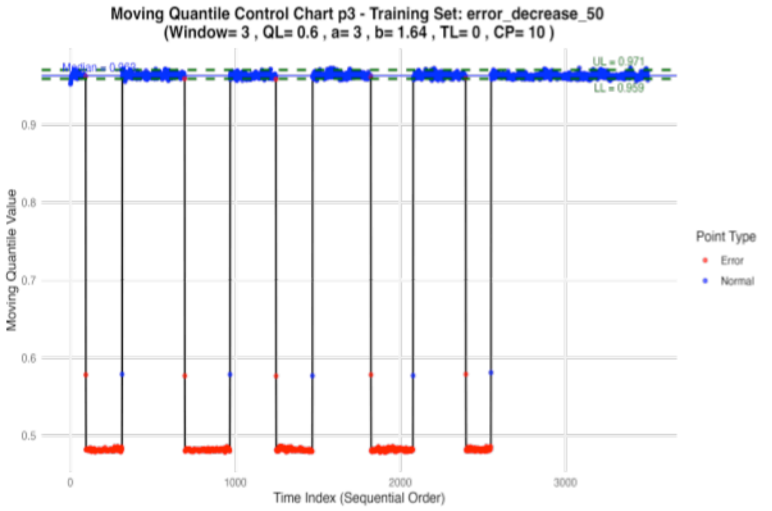

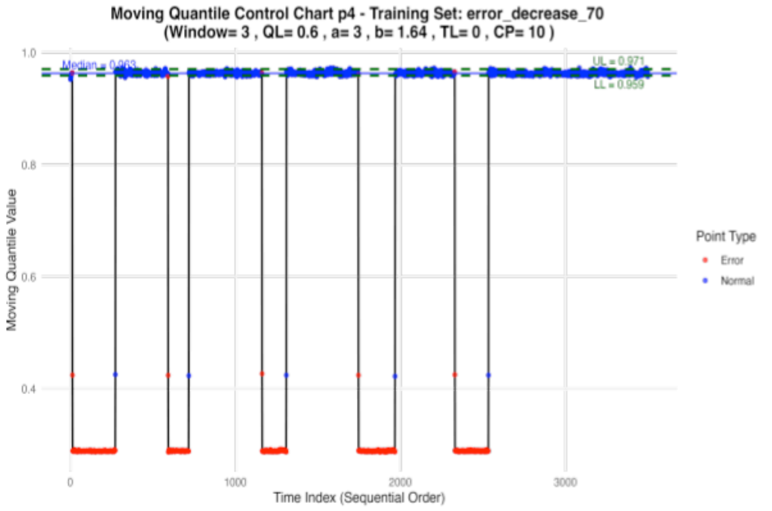

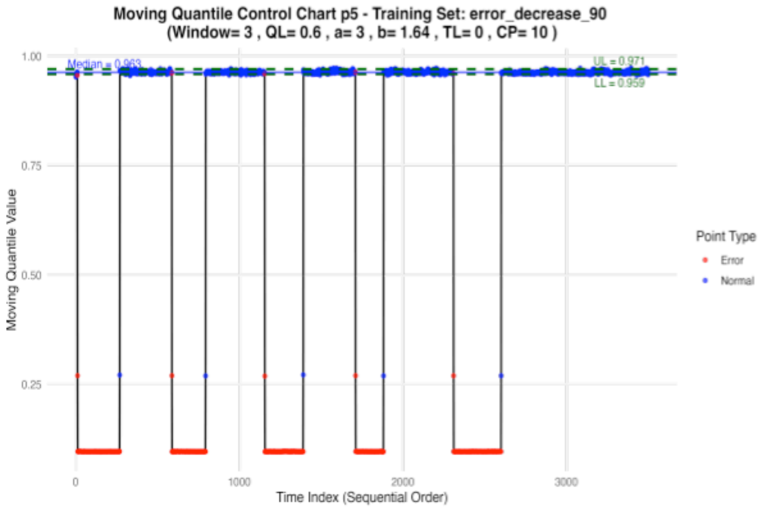

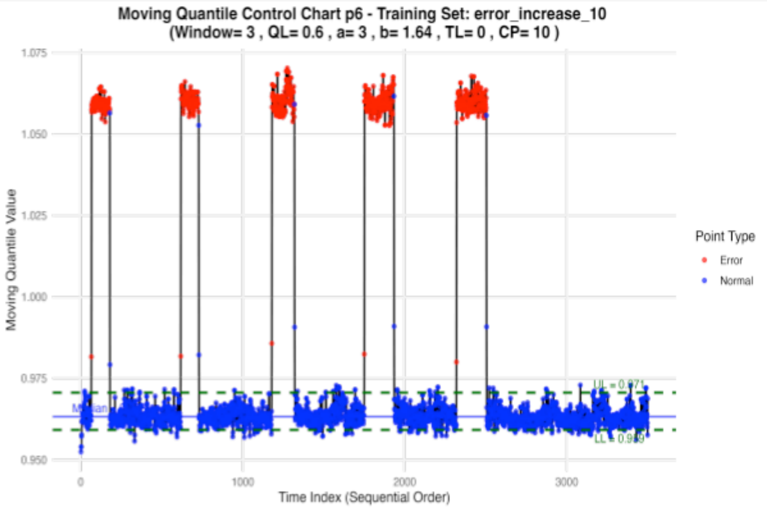

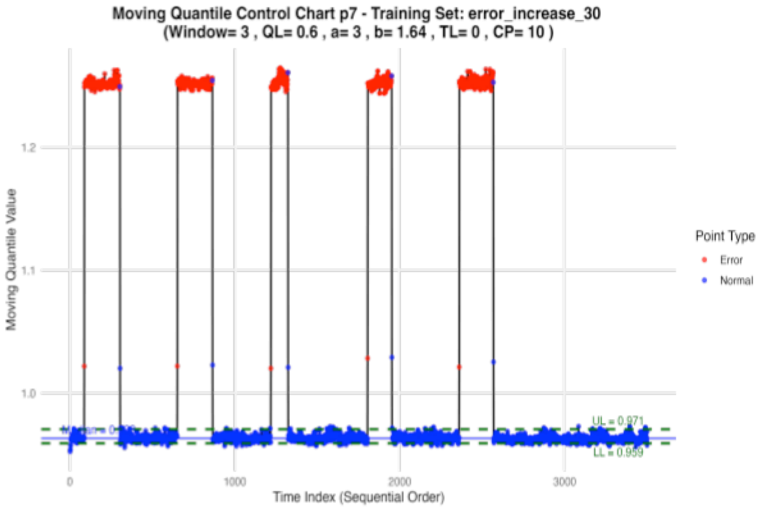

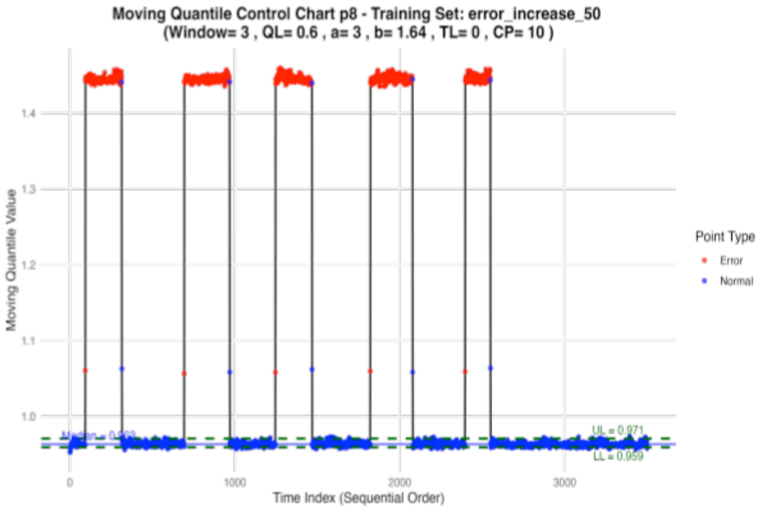

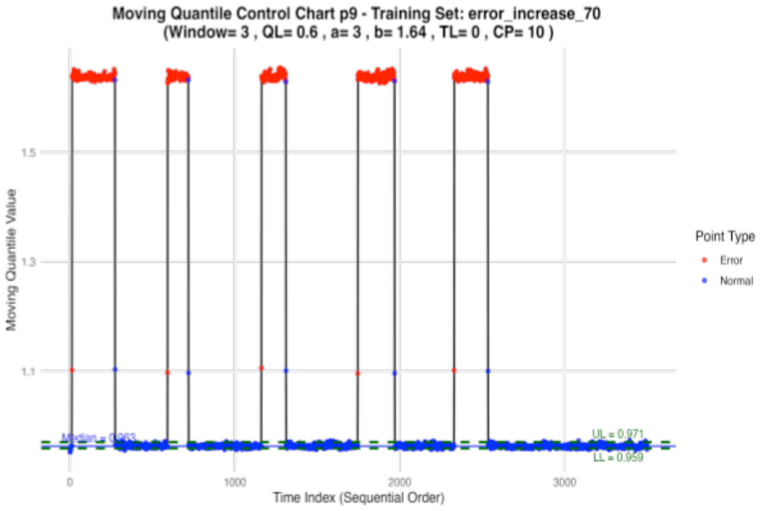

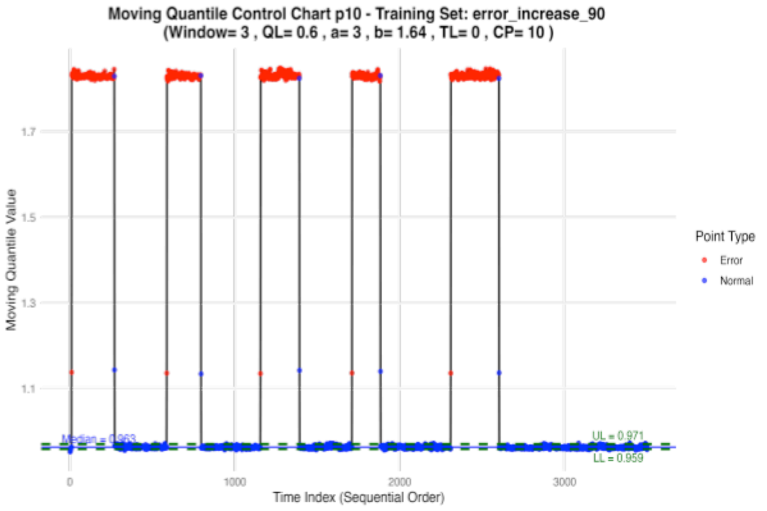

APTT_outputMQ - Test (p11-p20)

Moving Quantile Control Chart p11 - Test Set: error_decrease_10
(Window= 3 , QL= 0.6 , a= 3 , b= 1.64 , TL= 0 , CP= 10)

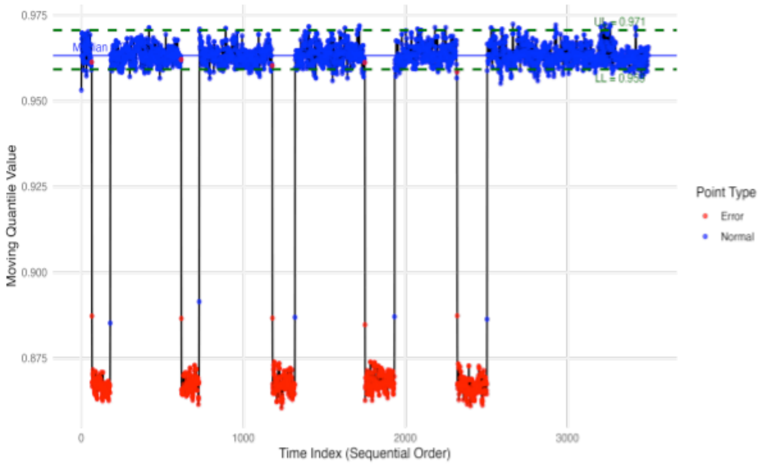

Moving Quantile Control Chart p16 - Test Set: error_increase_10
(Window= 3 , QL= 0.6 , a= 3 , b= 1.64 , TL= 0 , CP= 10)

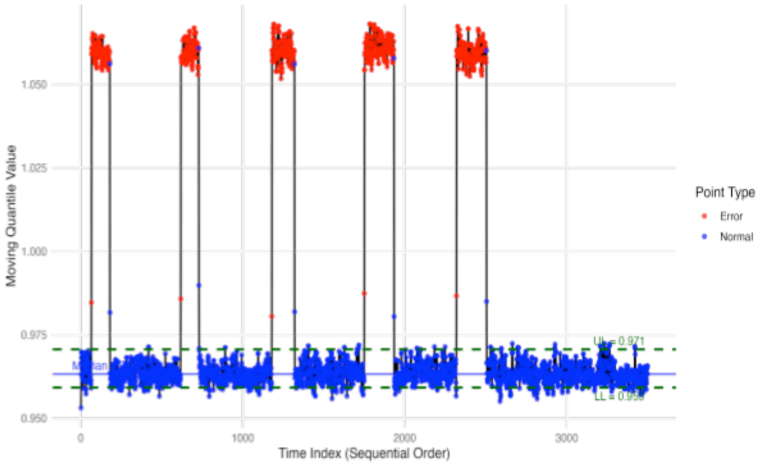

Moving Quantile Control Chart p12 - Test Set: error_decrease_30
(Window= 3 , QL= 0.6 , a= 3 , b= 1.64 , TL= 0 , CP= 10)

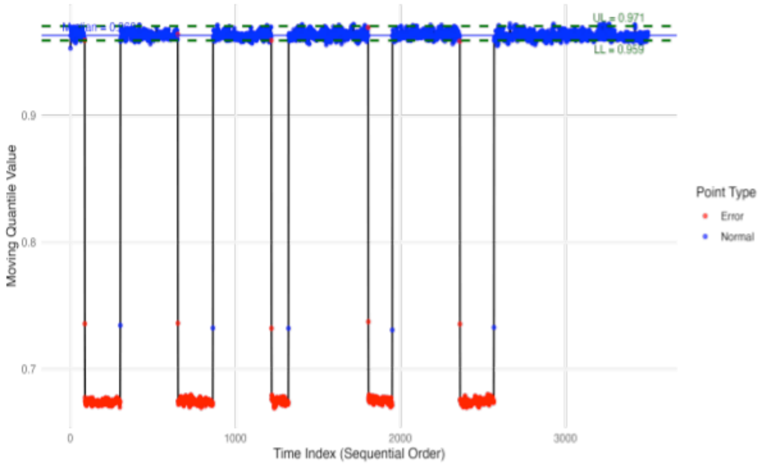

Moving Quantile Control Chart p17 - Test Set: error_increase_30
(Window= 3 , QL= 0.6 , a= 3 , b= 1.64 , TL= 0 , CP= 10)

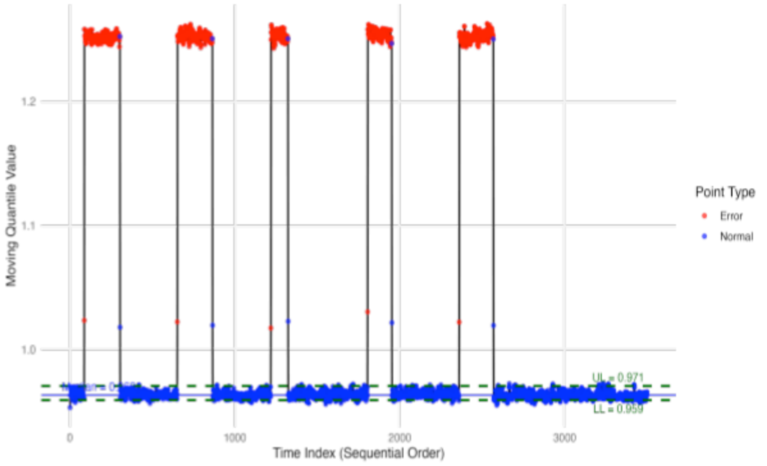

Moving Quantile Control Chart p13 - Test Set: error_decrease_50
(Window= 3 , QL= 0.6 , a= 3 , b= 1.64 , TL= 0 , CP= 10)

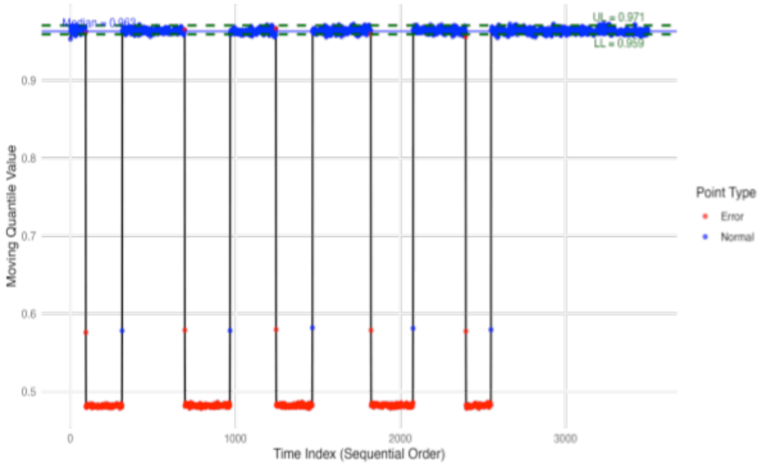

Moving Quantile Control Chart p18 - Test Set: error_increase_50
(Window= 3 , QL= 0.6 , a= 3 , b= 1.64 , TL= 0 , CP= 10)

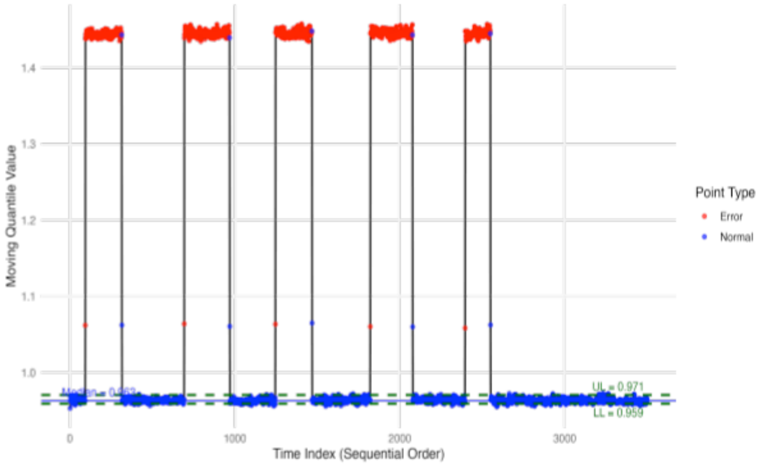

Moving Quantile Control Chart p14 - Test Set: error_decrease_70
(Window= 3 , QL= 0.6 , a= 3 , b= 1.64 , TL= 0 , CP= 10)

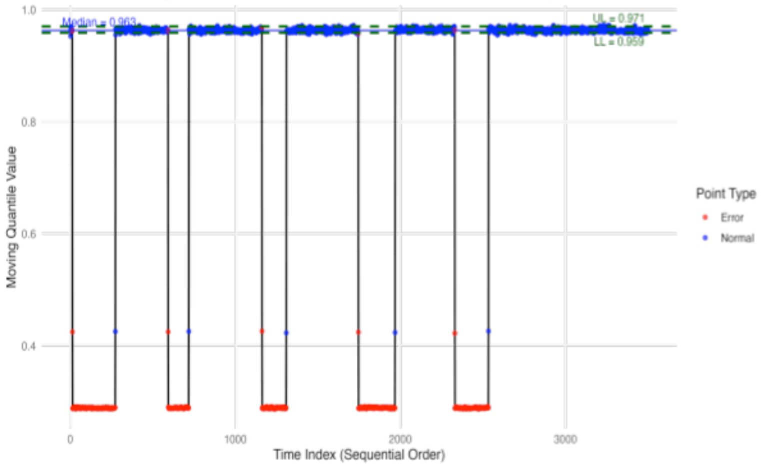

Moving Quantile Control Chart p19 - Test Set: error_increase_70
(Window= 3 , QL= 0.6 , a= 3 , b= 1.64 , TL= 0 , CP= 10)

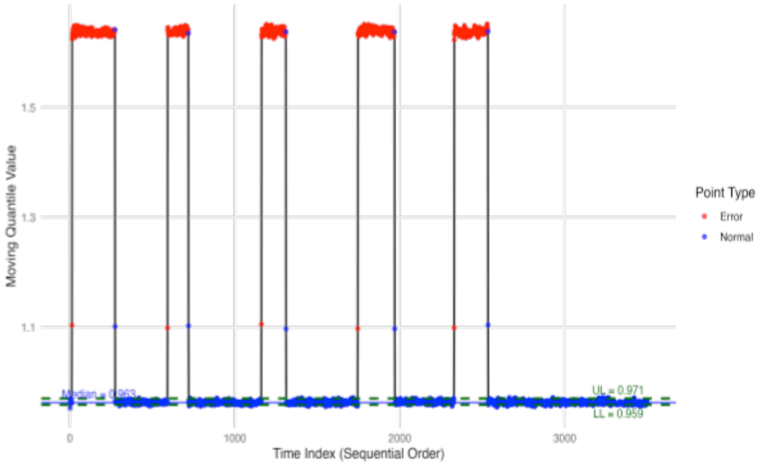

Moving Quantile Control Chart p15 - Test Set: error_decrease_90
(Window= 3 , QL= 0.6 , a= 3 , b= 1.64 , TL= 0 , CP= 10)

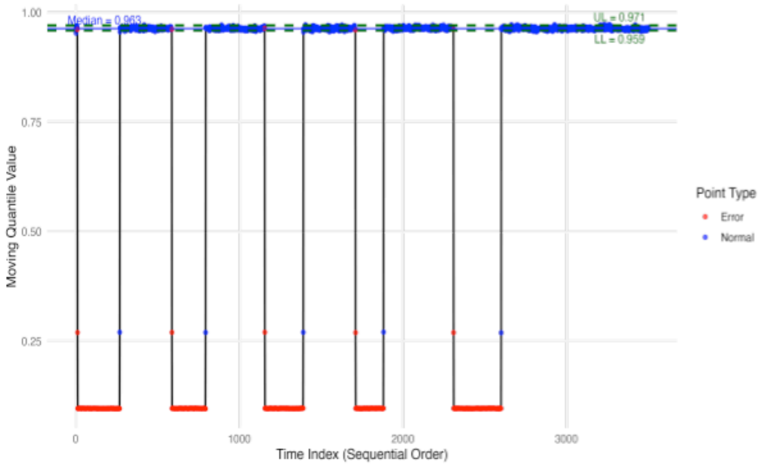

Moving Quantile Control Chart p20 - Test Set: error_increase_90
(Window= 3 , QL= 0.6 , a= 3 , b= 1.64 , TL= 0 , CP= 10)

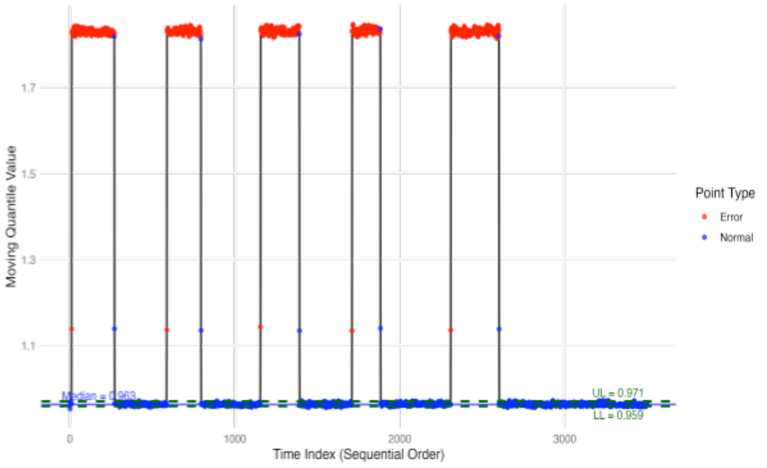

APTT_outputEWMA - Training (p1-p10)

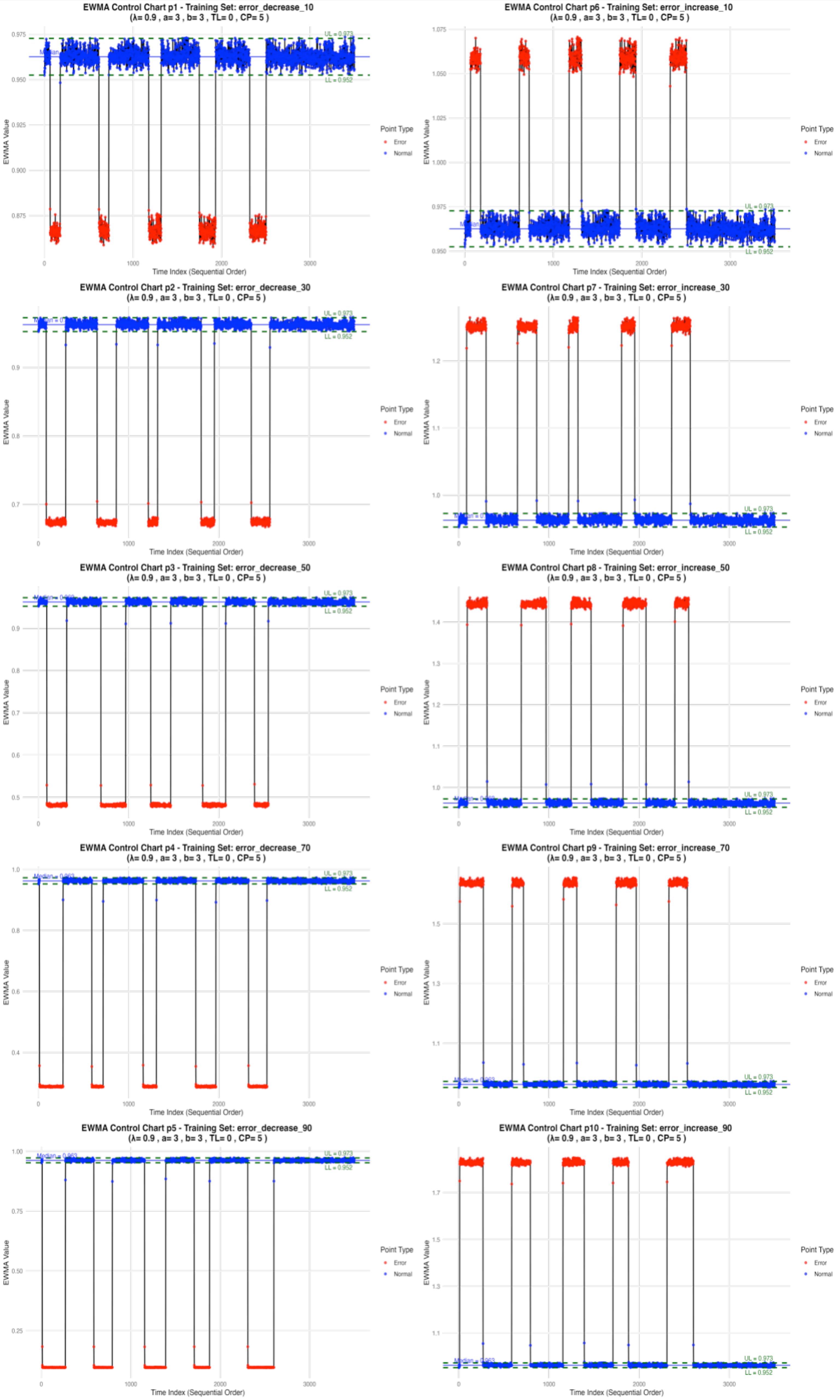

APTT_outputEWMA - Test (p11-p20)

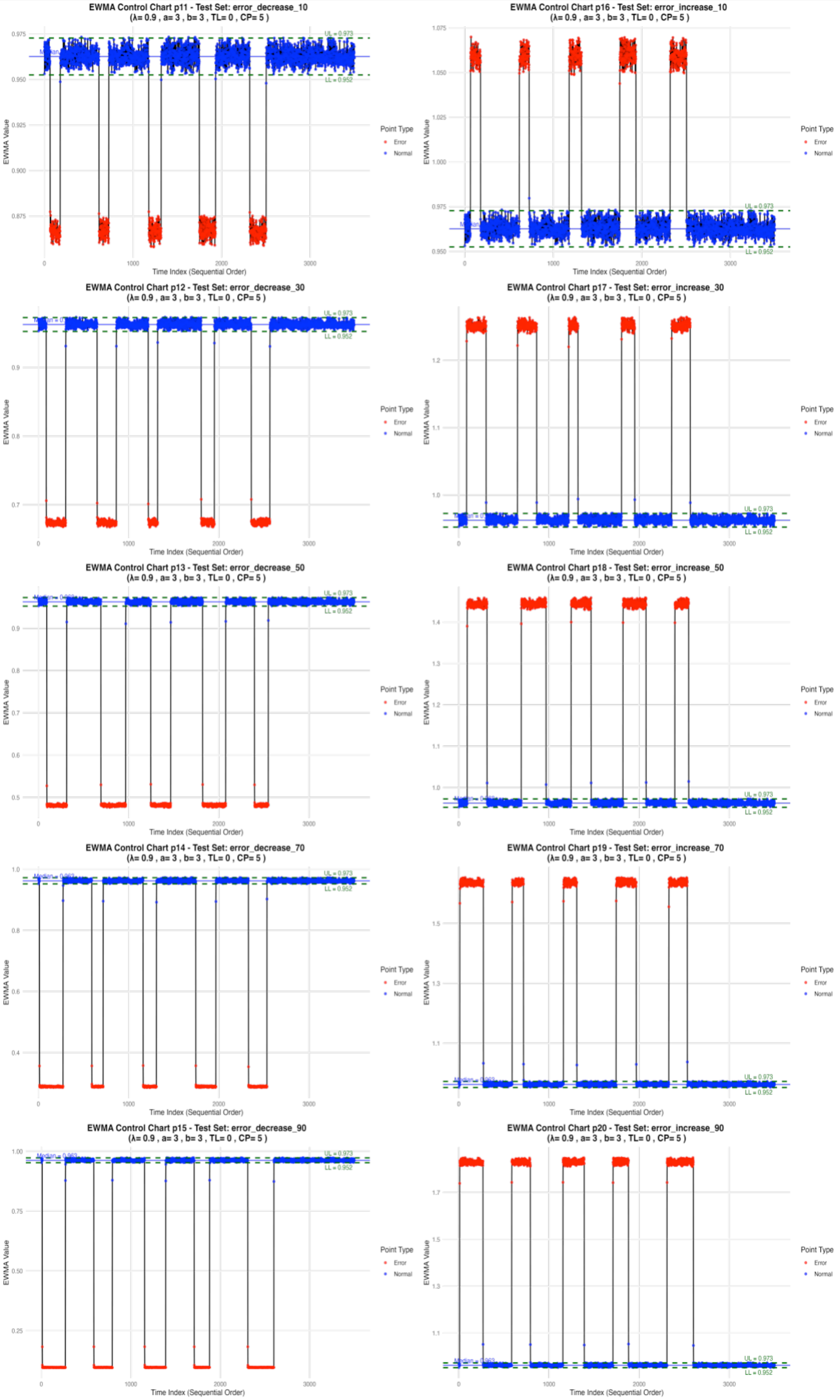

APTT_outputMA - Training (p1-p10)

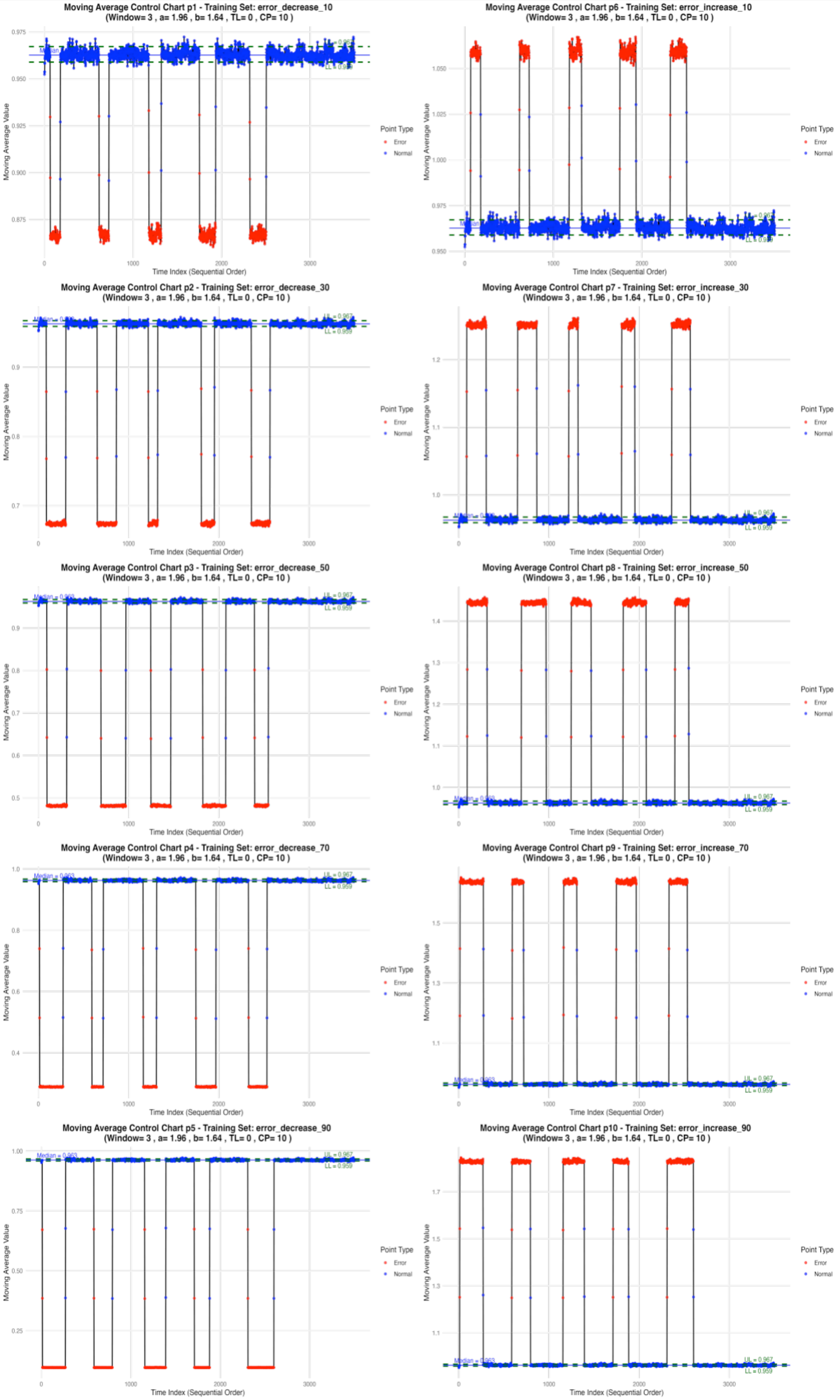

APTT_outputMA - Test (p11-p20)

Moving Average Control Chart p11 - Test Set: error_decrease_10
(Window= 3 , a= 1.96 , b= 1.64 , TL= 0 , CP= 10)

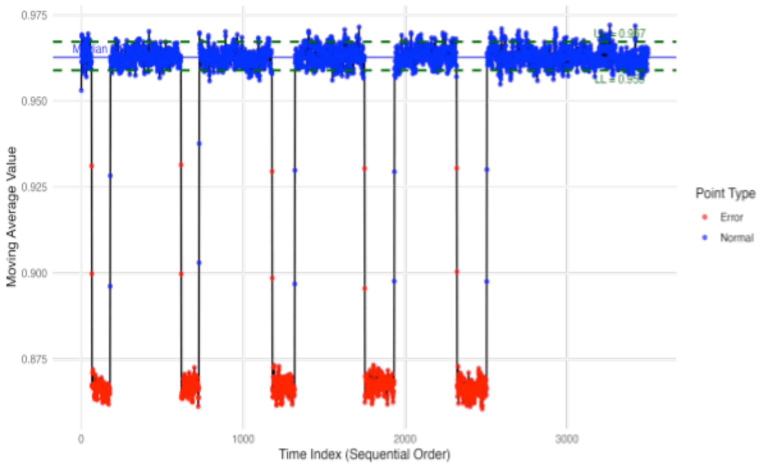

Moving Average Control Chart p16 - Test Set: error_increase_10
(Window= 3 , a= 1.96 , b= 1.64 , TL= 0 , CP= 10)

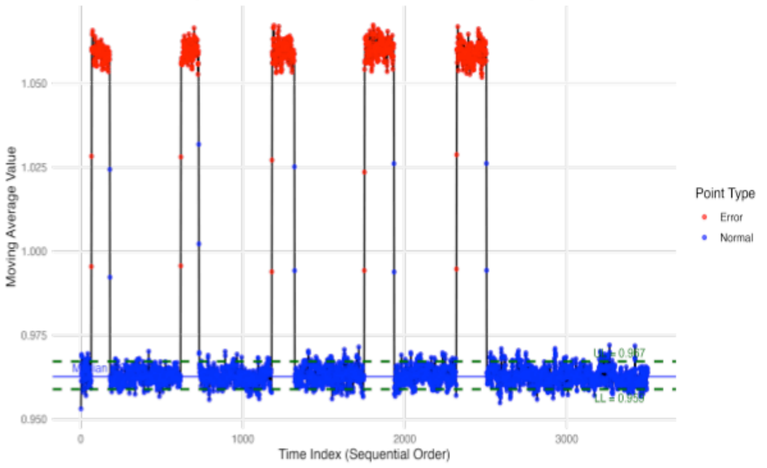

Moving Average Control Chart p12 - Test Set: error_decrease_30
(Window= 3 , a= 1.96 , b= 1.64 , TL= 0 , CP= 10)

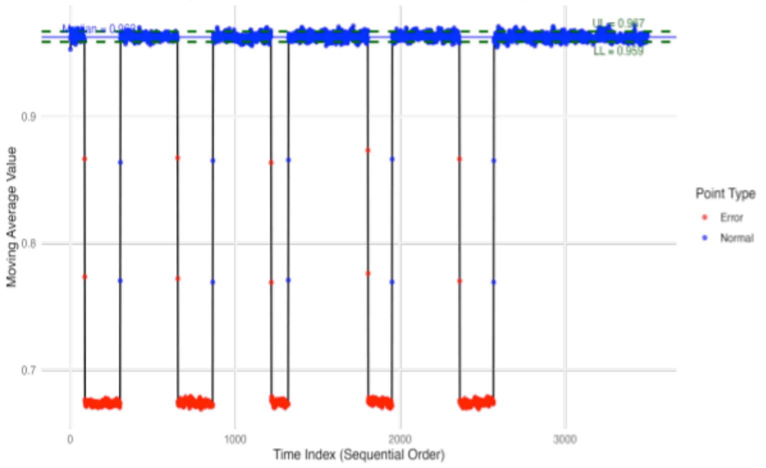

Moving Average Control Chart p17 - Test Set: error_increase_30
(Window= 3 , a= 1.96 , b= 1.64 , TL= 0 , CP= 10)

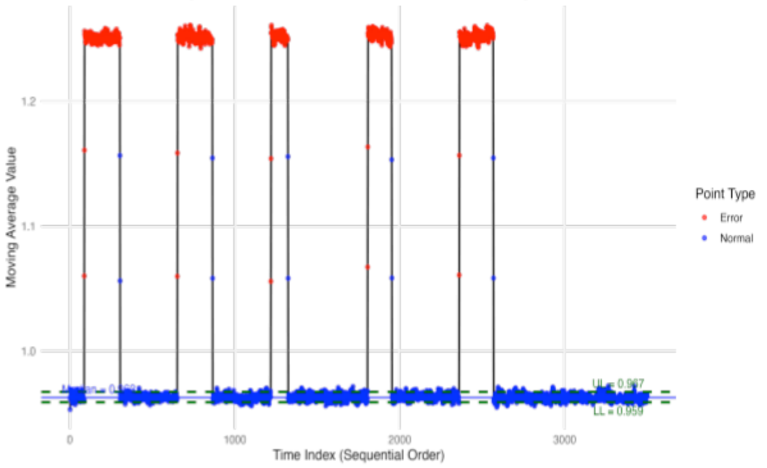

Moving Average Control Chart p13 - Test Set: error_decrease_50
(Window= 3 , a= 1.96 , b= 1.64 , TL= 0 , CP= 10)

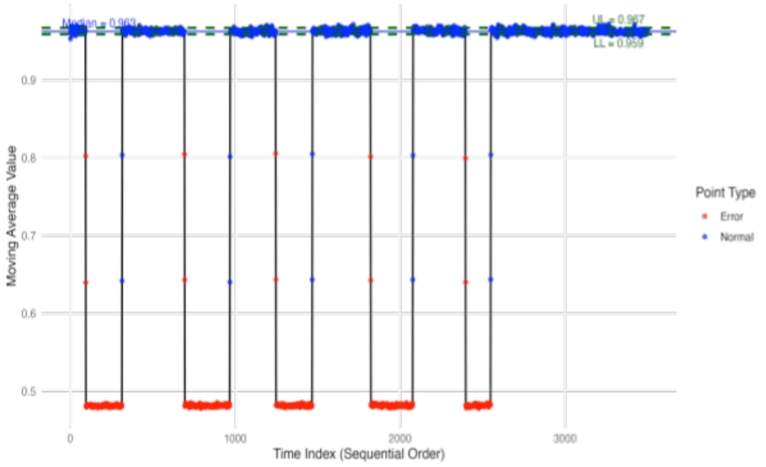

Moving Average Control Chart p18 - Test Set: error_increase_50
(Window= 3 , a= 1.96 , b= 1.64 , TL= 0 , CP= 10)

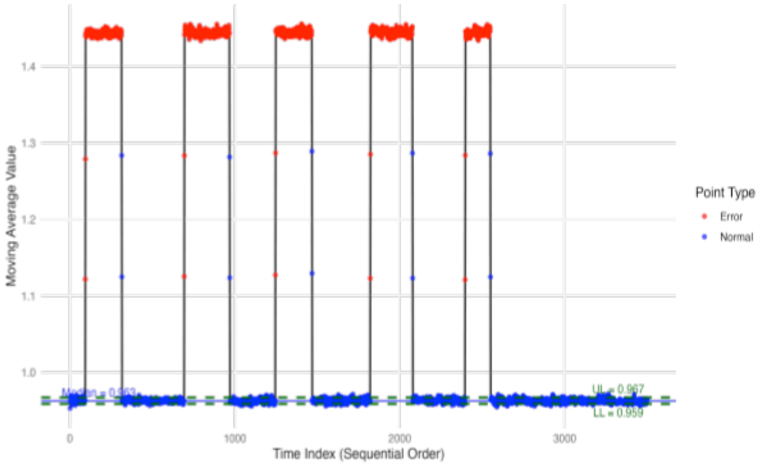

Moving Average Control Chart p14 - Test Set: error_decrease_70
(Window= 3 , a= 1.96 , b= 1.64 , TL= 0 , CP= 10)

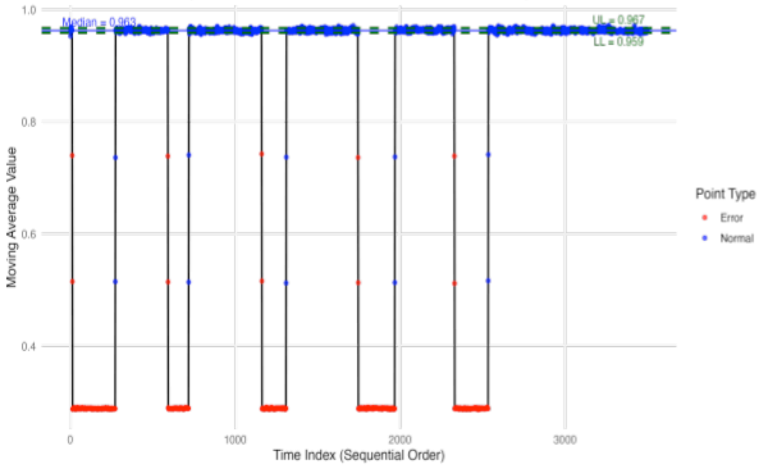

Moving Average Control Chart p19 - Test Set: error_increase_70
(Window= 3 , a= 1.96 , b= 1.64 , TL= 0 , CP= 10)

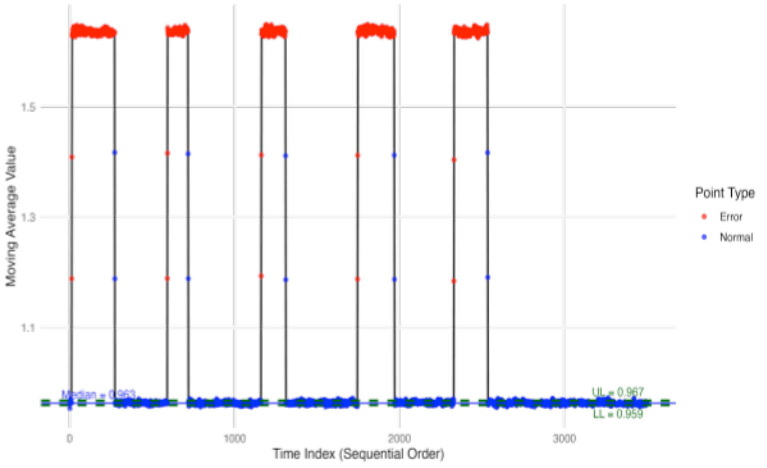

Moving Average Control Chart p15 - Test Set: error_decrease_90
(Window= 3 , a= 1.96 , b= 1.64 , TL= 0 , CP= 10)

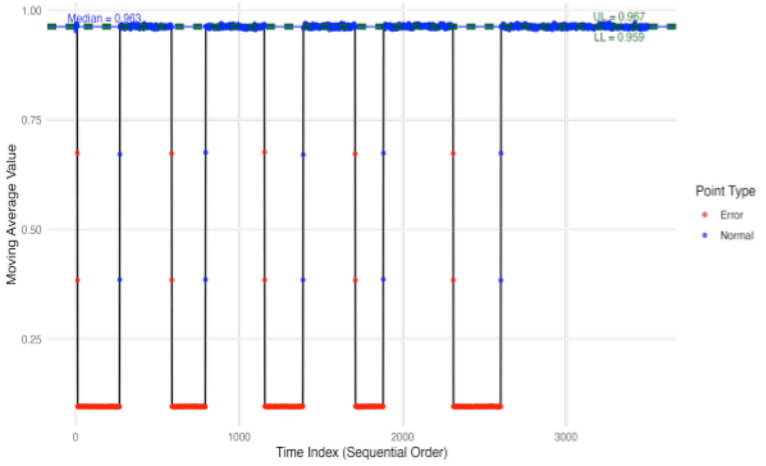

Moving Average Control Chart p20 - Test Set: error_increase_90
(Window= 3 , a= 1.96 , b= 1.64 , TL= 0 , CP= 10)

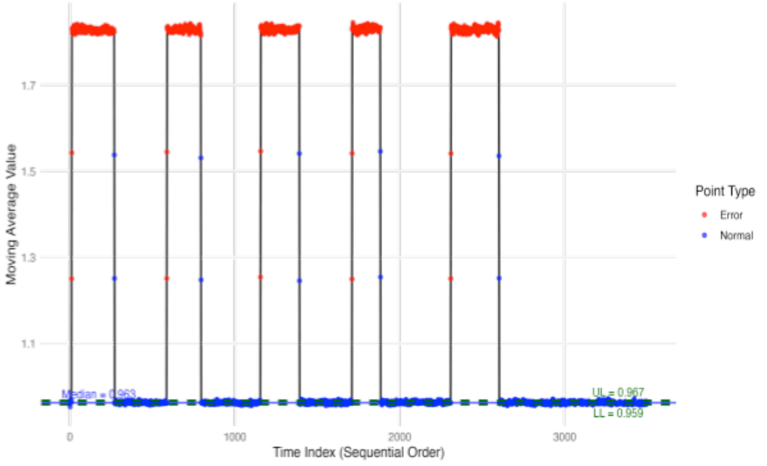

TT_outputMQ - Training (p1-p10)

Moving Quantile Control Chart p1 - Training Set: error_decrease_10
(Window= 3 , QL= 0.6 , a= 1.64 , b= 1.96 , TL= 0 , CP= 10)

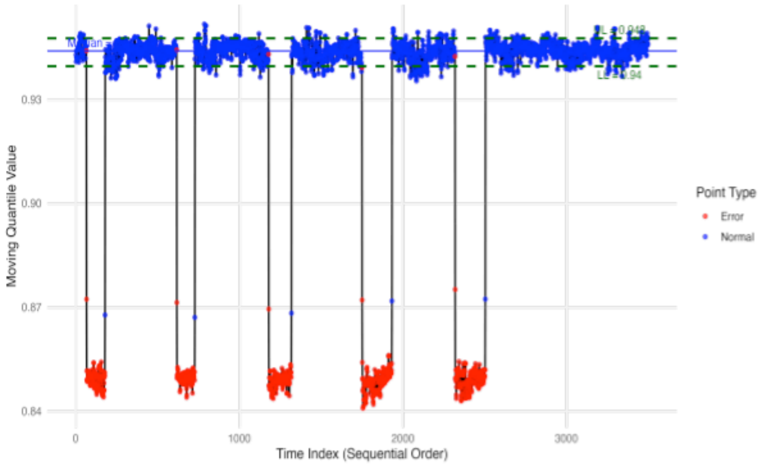

Moving Quantile Control Chart p6 - Training Set: error_increase_10
(Window= 3 , QL= 0.6 , a= 1.64 , b= 1.96 , TL= 0 , CP= 10)

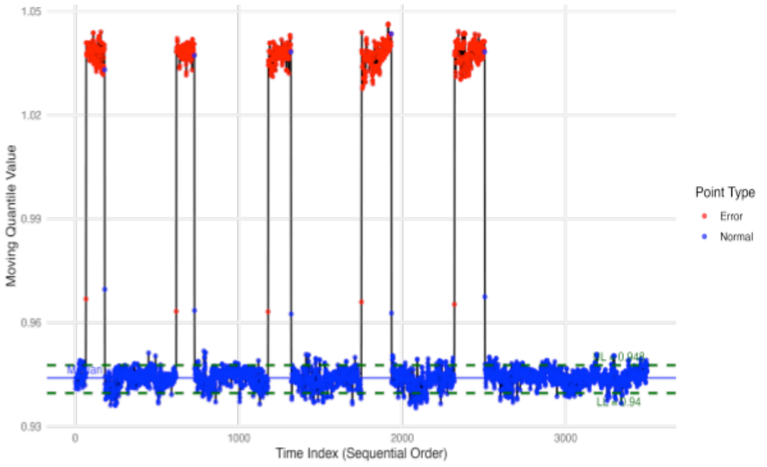

Moving Quantile Control Chart p2 - Training Set: error_decrease_30
(Window= 3 , QL= 0.6 , a= 1.64 , b= 1.96 , TL= 0 , CP= 10)

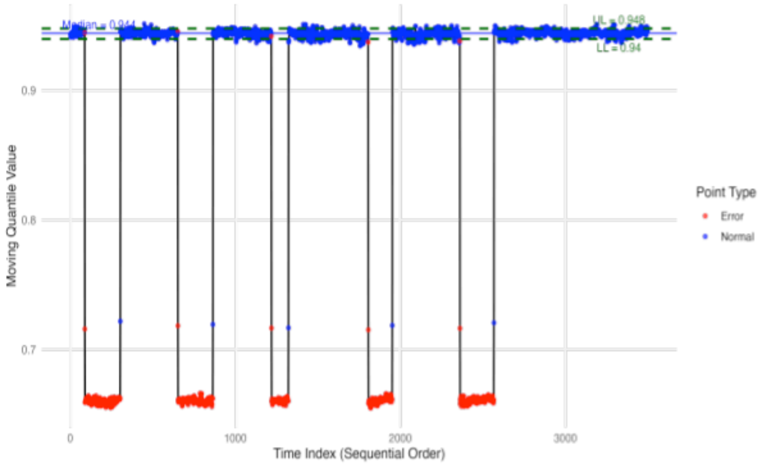

Moving Quantile Control Chart p7 - Training Set: error_increase_30
(Window= 3 , QL= 0.6 , a= 1.64 , b= 1.96 , TL= 0 , CP= 10)

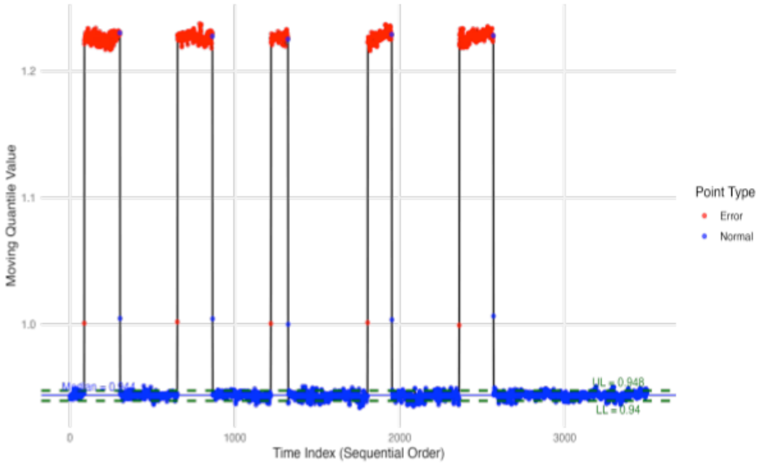

Moving Quantile Control Chart p3 - Training Set: error_decrease_50
(Window= 3 , QL= 0.6 , a= 1.64 , b= 1.96 , TL= 0 , CP= 10)

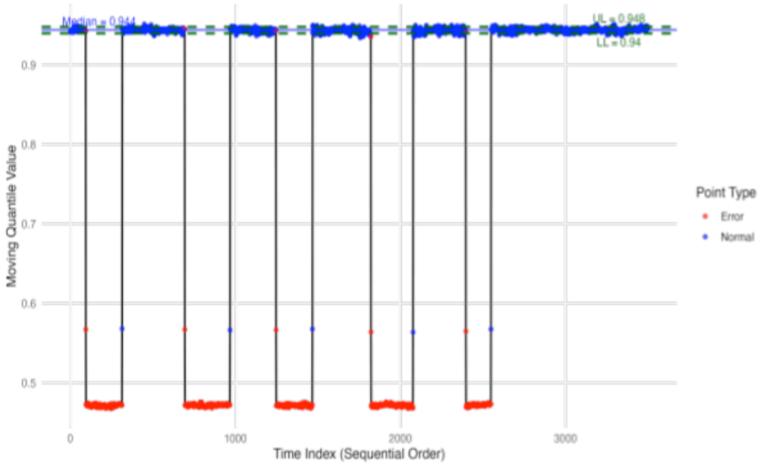

Moving Quantile Control Chart p8 - Training Set: error_increase_50
(Window= 3 , QL= 0.6 , a= 1.64 , b= 1.96 , TL= 0 , CP= 10)

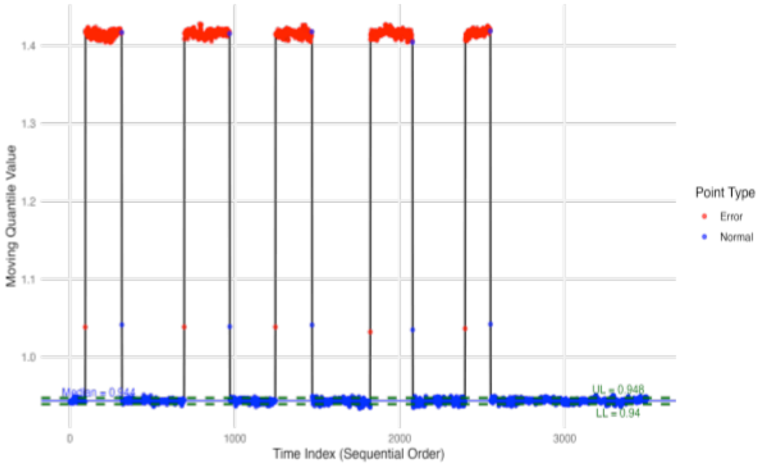

Moving Quantile Control Chart p4 - Training Set: error_decrease_70
(Window= 3 , QL= 0.6 , a= 1.64 , b= 1.96 , TL= 0 , CP= 10)

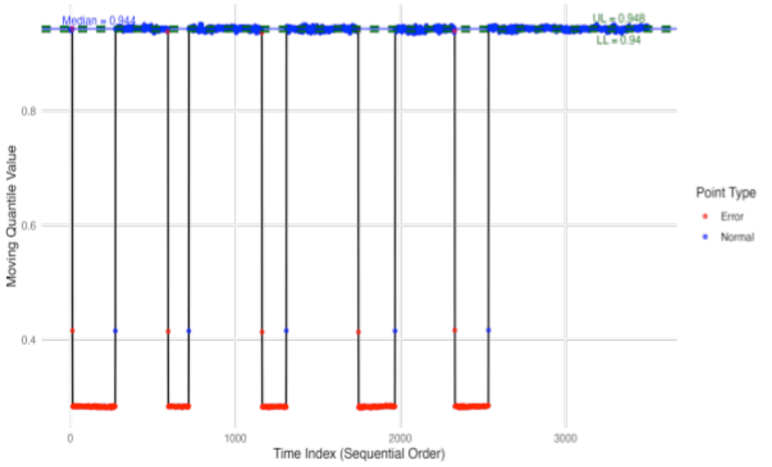

Moving Quantile Control Chart p9 - Training Set: error_increase_70
(Window= 3 , QL= 0.6 , a= 1.64 , b= 1.96 , TL= 0 , CP= 10)

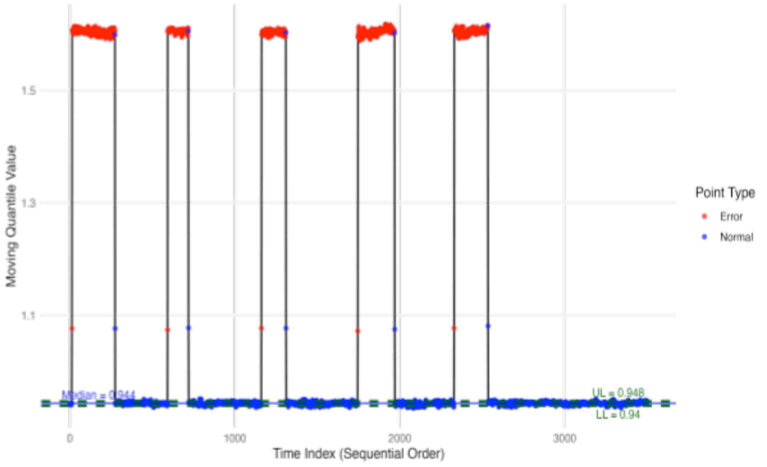

Moving Quantile Control Chart p5 - Training Set: error_decrease_90
(Window= 3 , QL= 0.6 , a= 1.64 , b= 1.96 , TL= 0 , CP= 10)

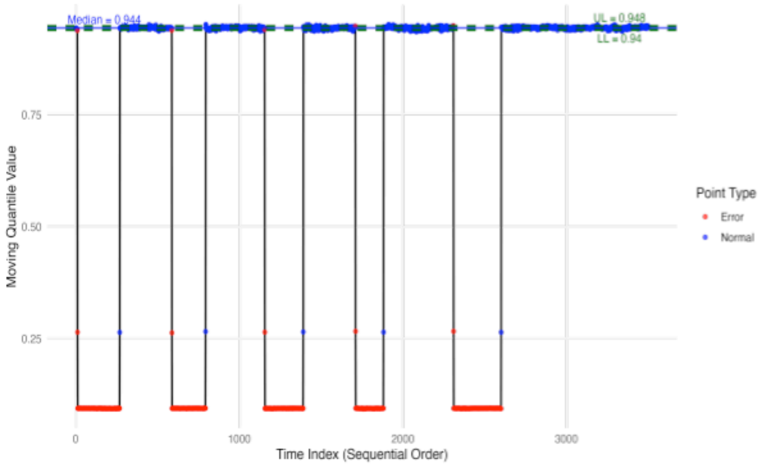

Moving Quantile Control Chart p10 - Training Set: error_increase_90
(Window= 3 , QL= 0.6 , a= 1.64 , b= 1.96 , TL= 0 , CP= 10)

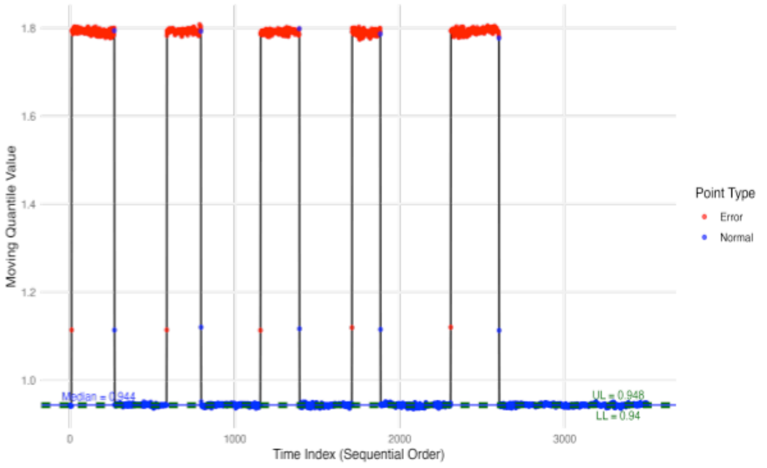

TT_outputMQ - Test (p11-p20)

Moving Quantile Control Chart p11 - Test Set: error_decrease_10
(Window= 3 , QL= 0.6 , a= 1.64 , b= 1.96 , TL= 0 , CP= 10)

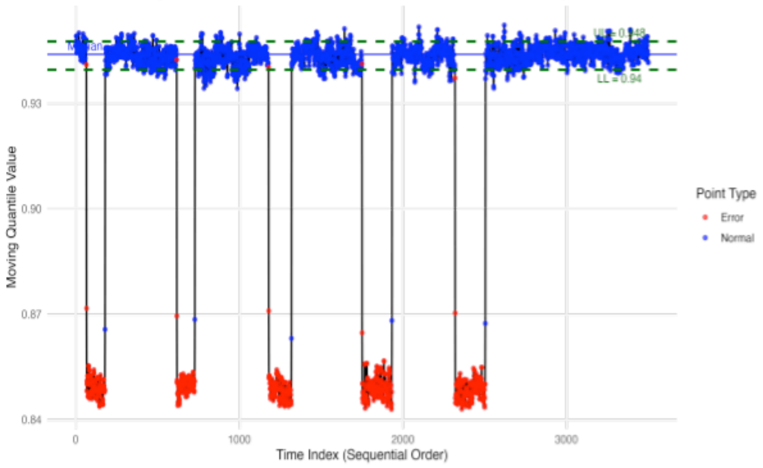

Moving Quantile Control Chart p12 - Test Set: error_decrease_30
(Window= 3 , QL= 0.6 , a= 1.64 , b= 1.96 , TL= 0 , CP= 10)

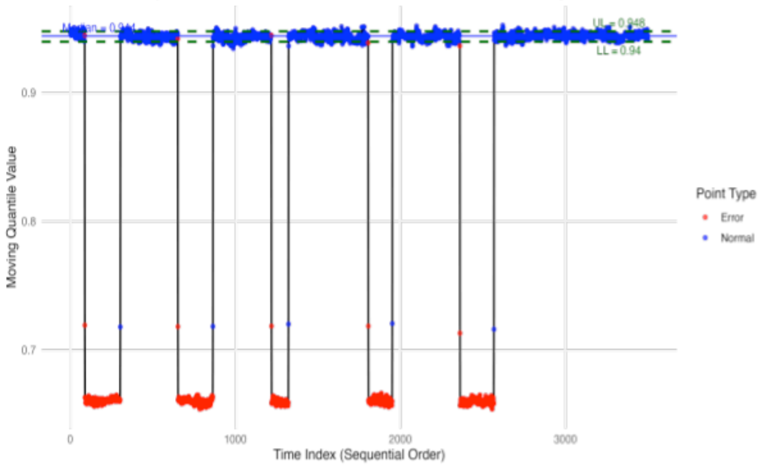

Moving Quantile Control Chart p13 - Test Set: error_decrease_50
(Window= 3 , QL= 0.6 , a= 1.64 , b= 1.96 , TL= 0 , CP= 10)

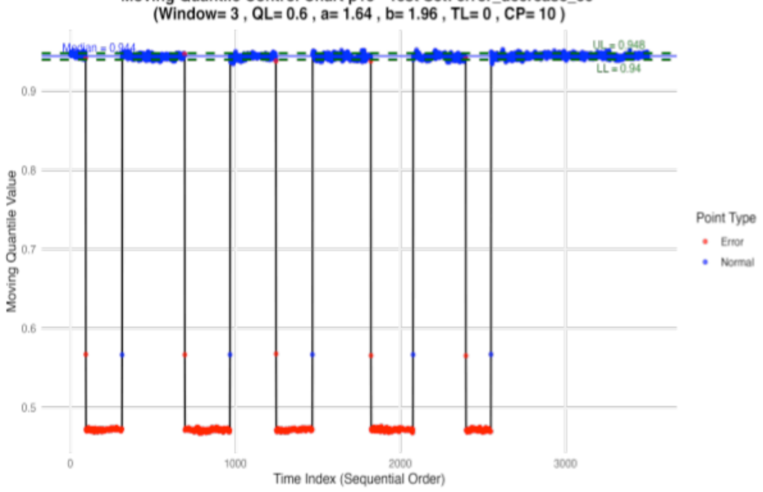

Moving Quantile Control Chart p14 - Test Set: error_decrease_70
(Window= 3 , QL= 0.6 , a= 1.64 , b= 1.96 , TL= 0 , CP= 10)

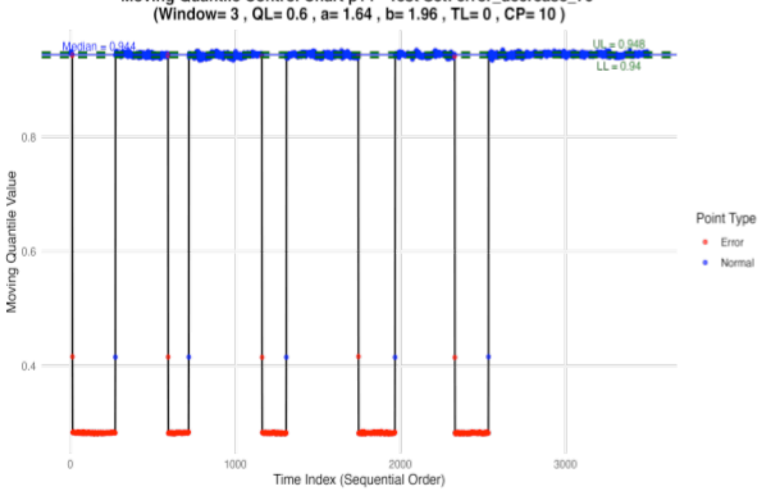

Moving Quantile Control Chart p15 - Test Set: error_decrease_90
(Window= 3 , QL= 0.6 , a= 1.64 , b= 1.96 , TL= 0 , CP= 10)

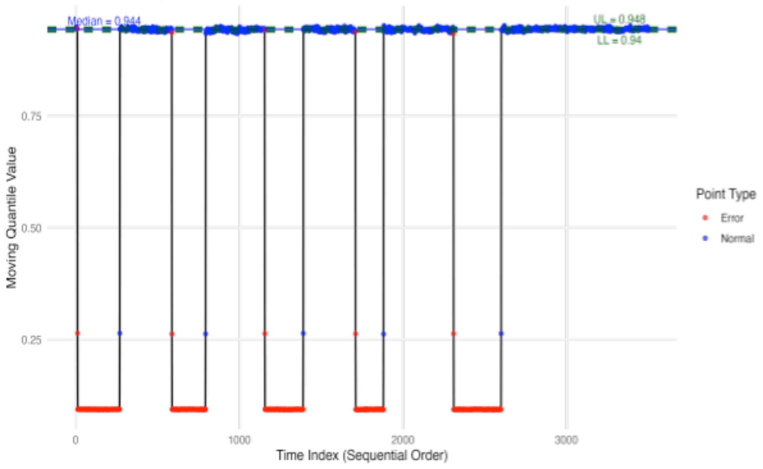

Moving Quantile Control Chart p16 - Test Set: error_increase_10
(Window= 3 , QL= 0.6 , a= 1.64 , b= 1.96 , TL= 0 , CP= 10)

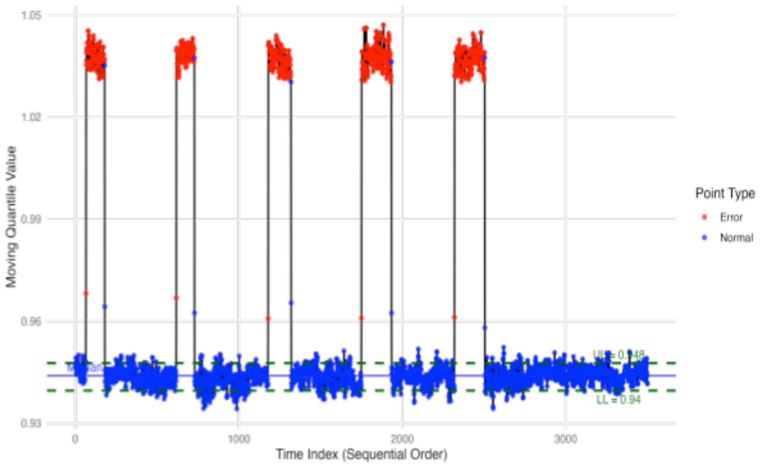

Moving Quantile Control Chart p17 - Test Set: error_increase_30
(Window= 3 , QL= 0.6 , a= 1.64 , b= 1.96 , TL= 0 , CP= 10)

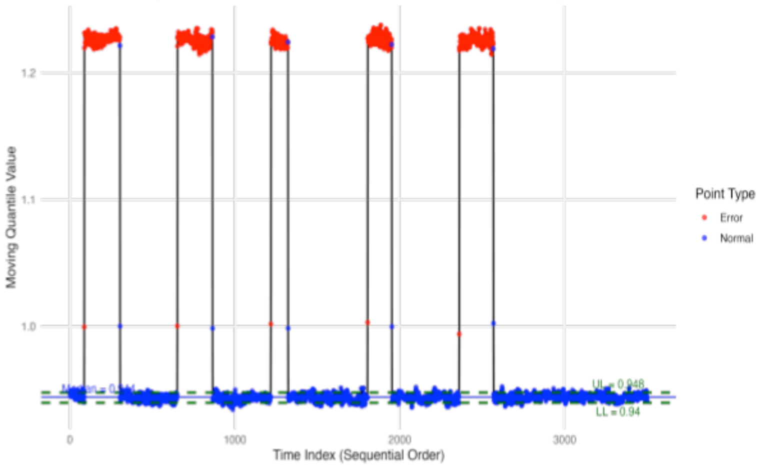

Moving Quantile Control Chart p18 - Test Set: error_increase_50
(Window= 3 , QL= 0.6 , a= 1.64 , b= 1.96 , TL= 0 , CP= 10)

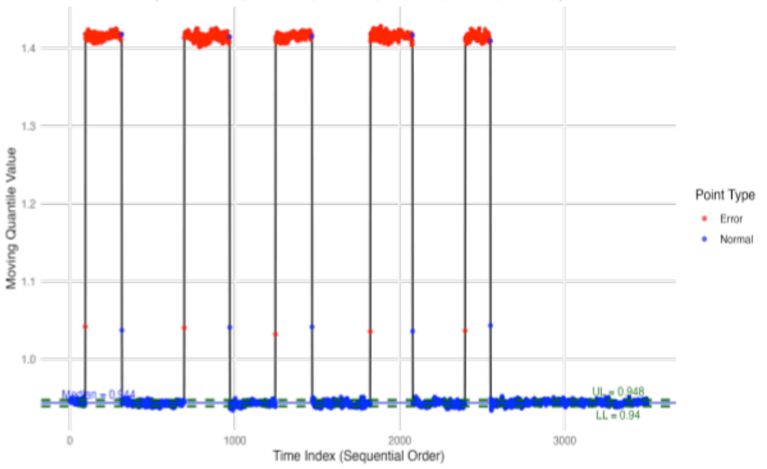

Moving Quantile Control Chart p19 - Test Set: error_increase_70
(Window= 3 , QL= 0.6 , a= 1.64 , b= 1.96 , TL= 0 , CP= 10)

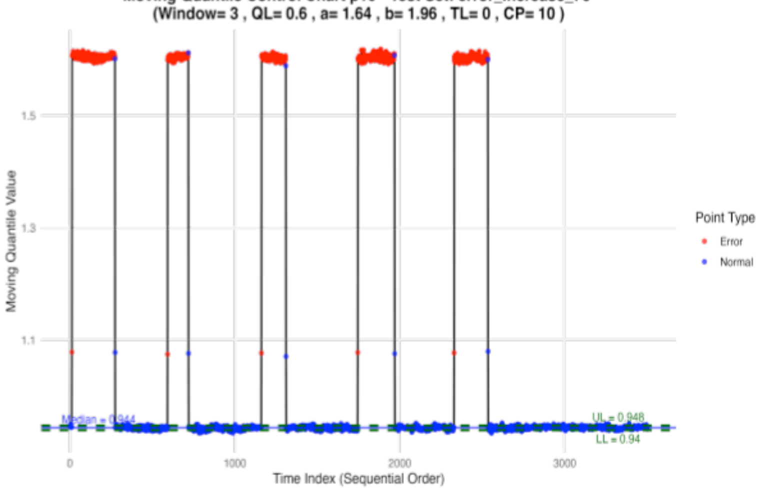

Moving Quantile Control Chart p20 - Test Set: error_increase_90
(Window= 3 , QL= 0.6 , a= 1.64 , b= 1.96 , TL= 0 , CP= 10)

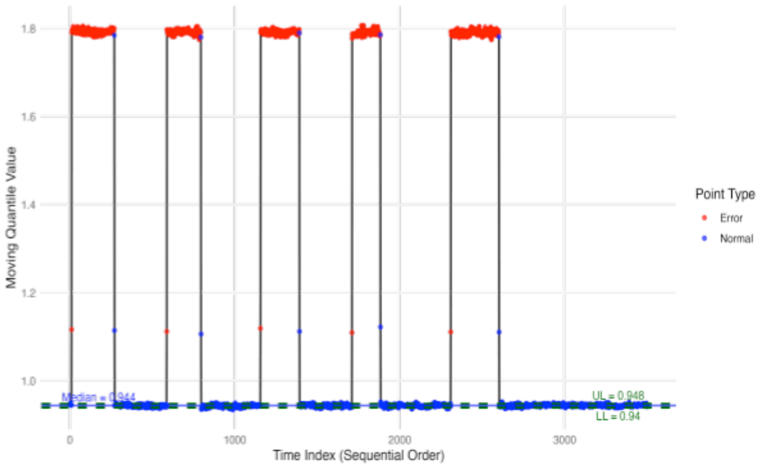

TT_outputEWMA - Training (p1-p10)

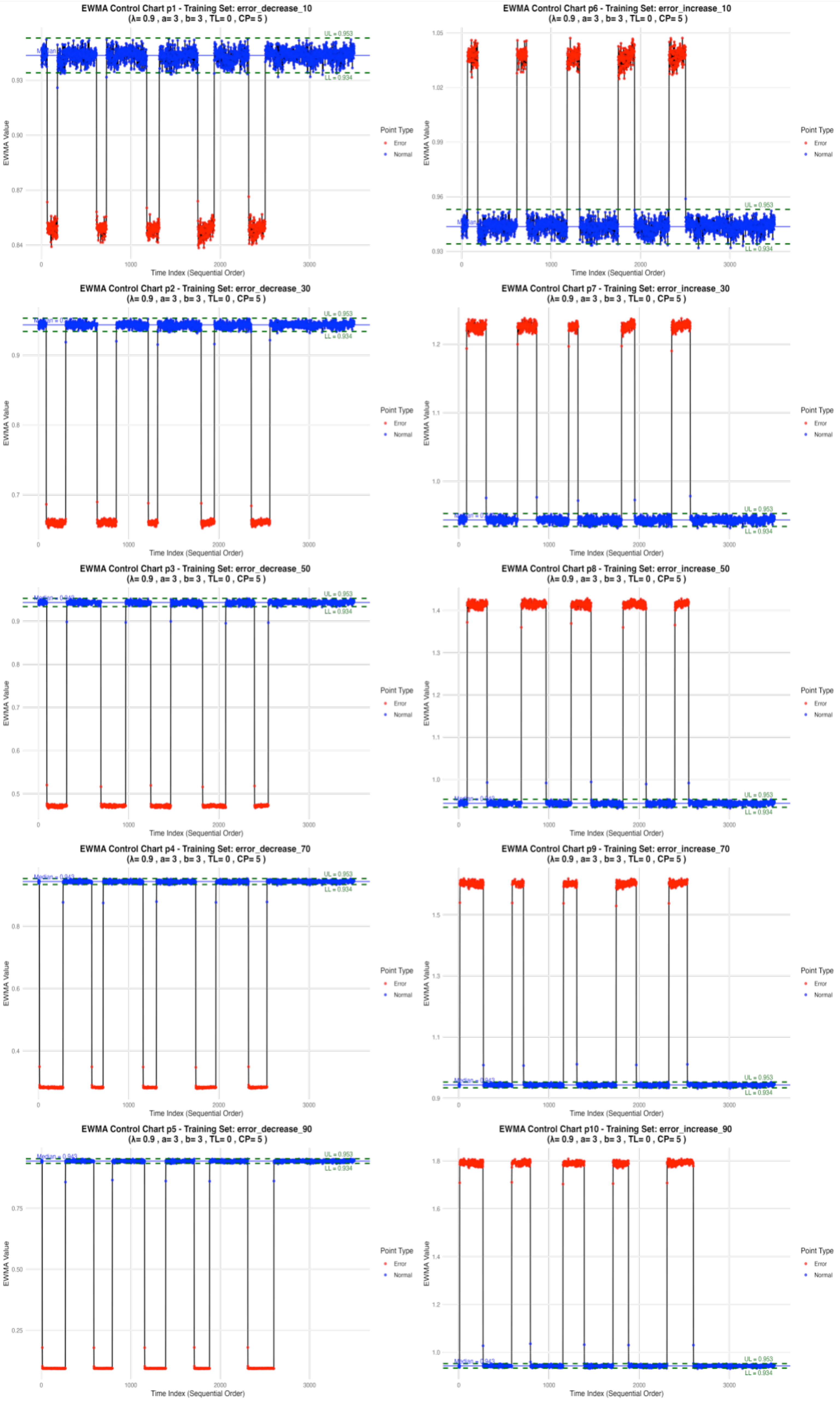

TT_outputEWMA - Test (p11-p20)

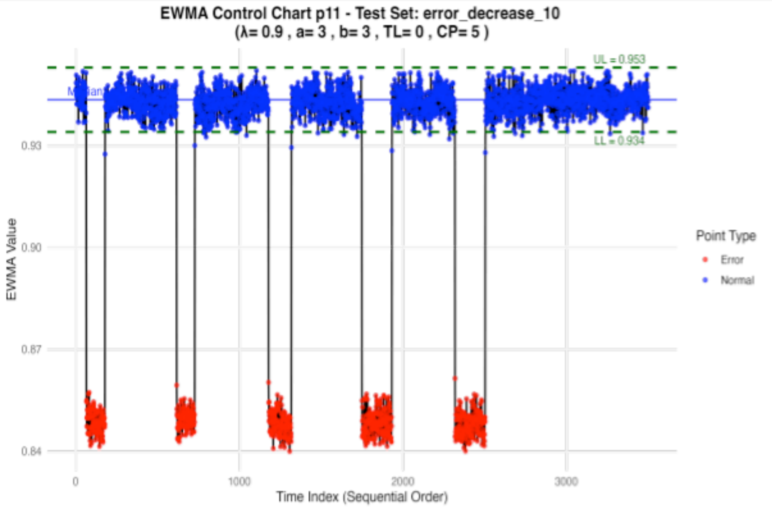

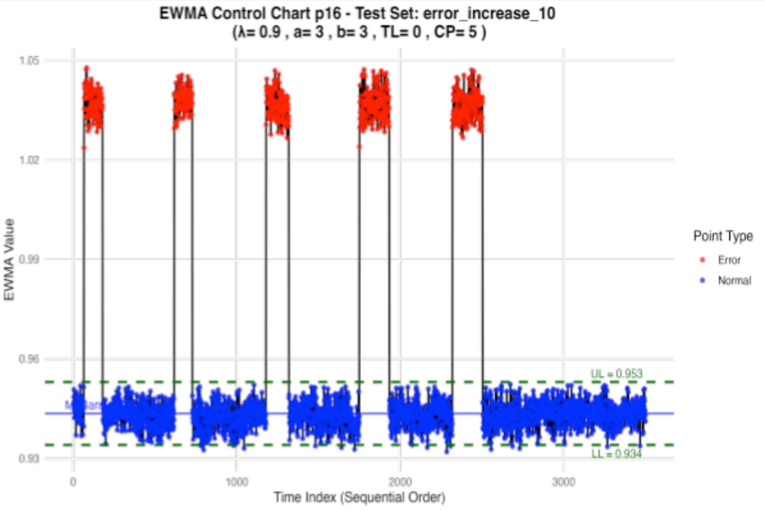

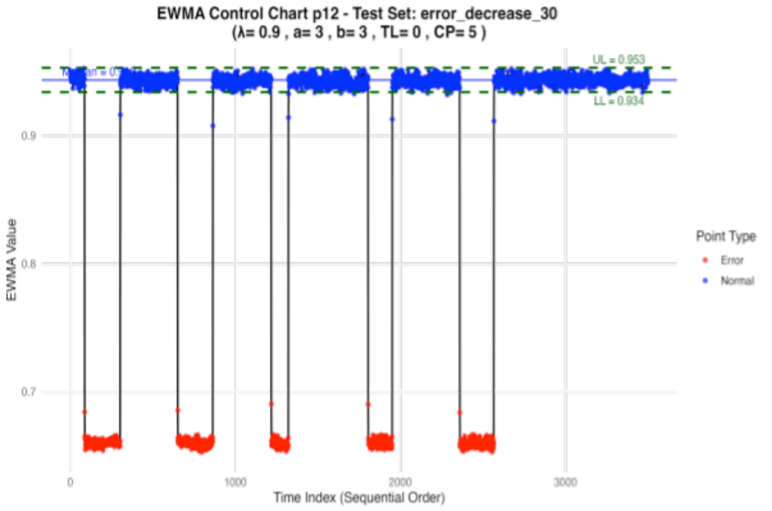

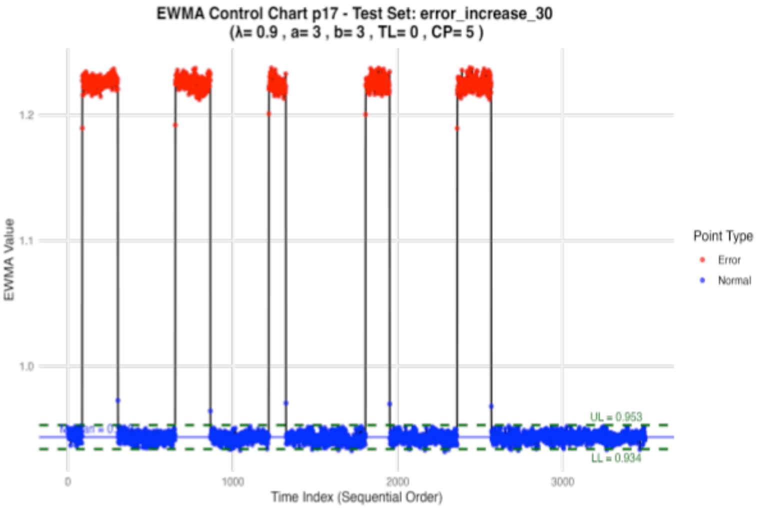

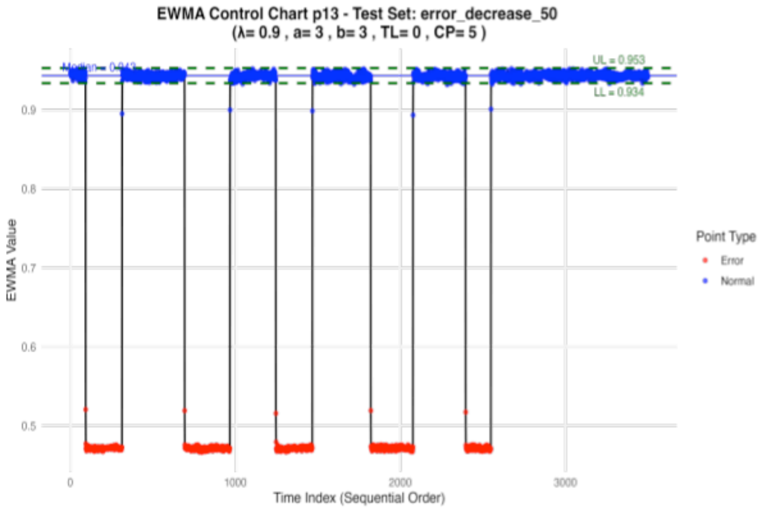

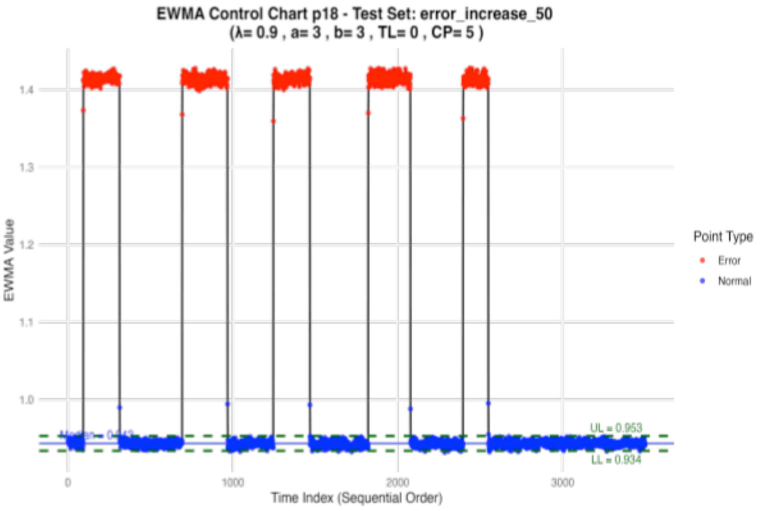

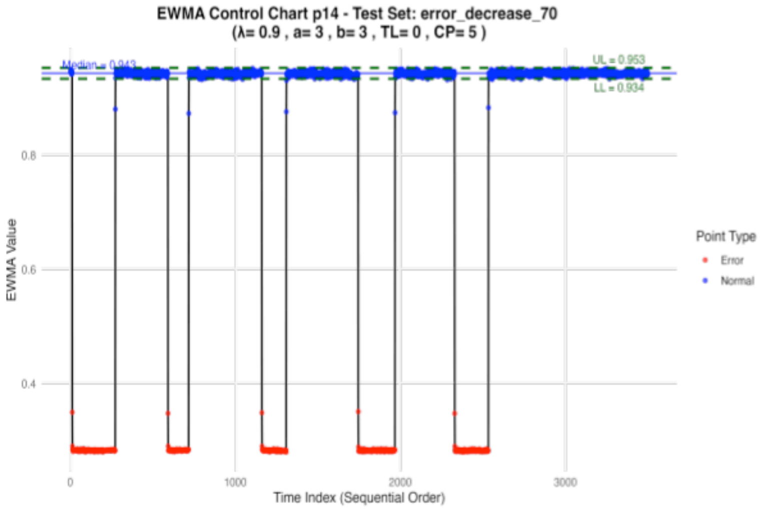

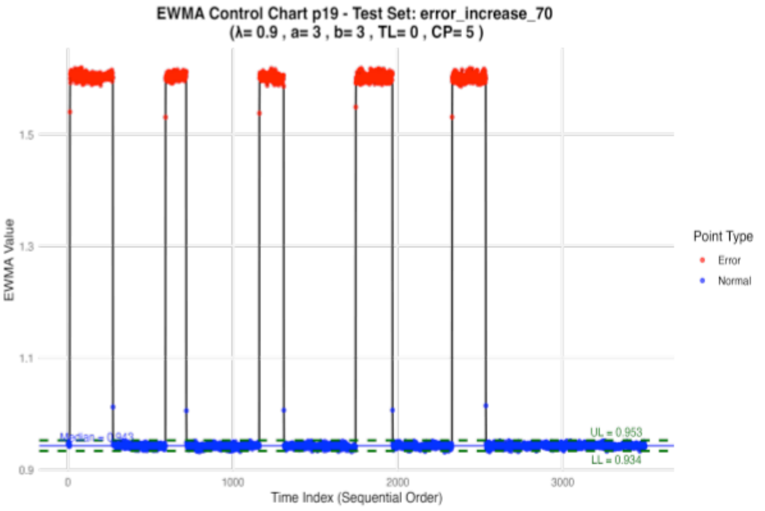

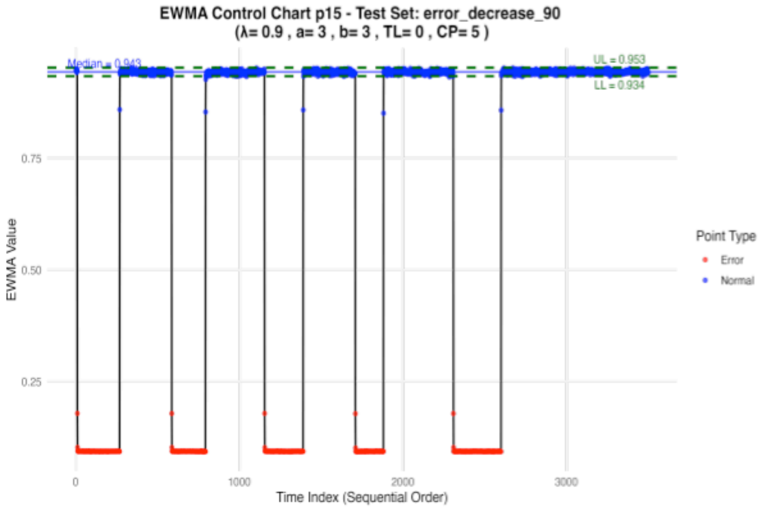

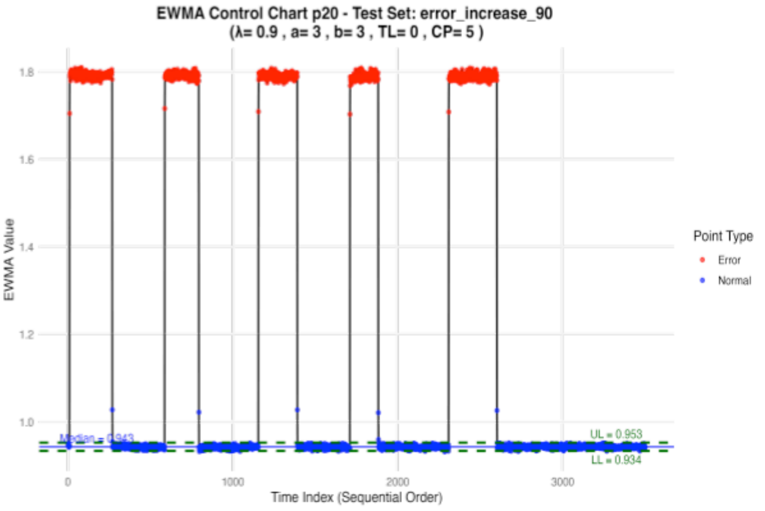

TT_outputMA - Training (p1-p10)

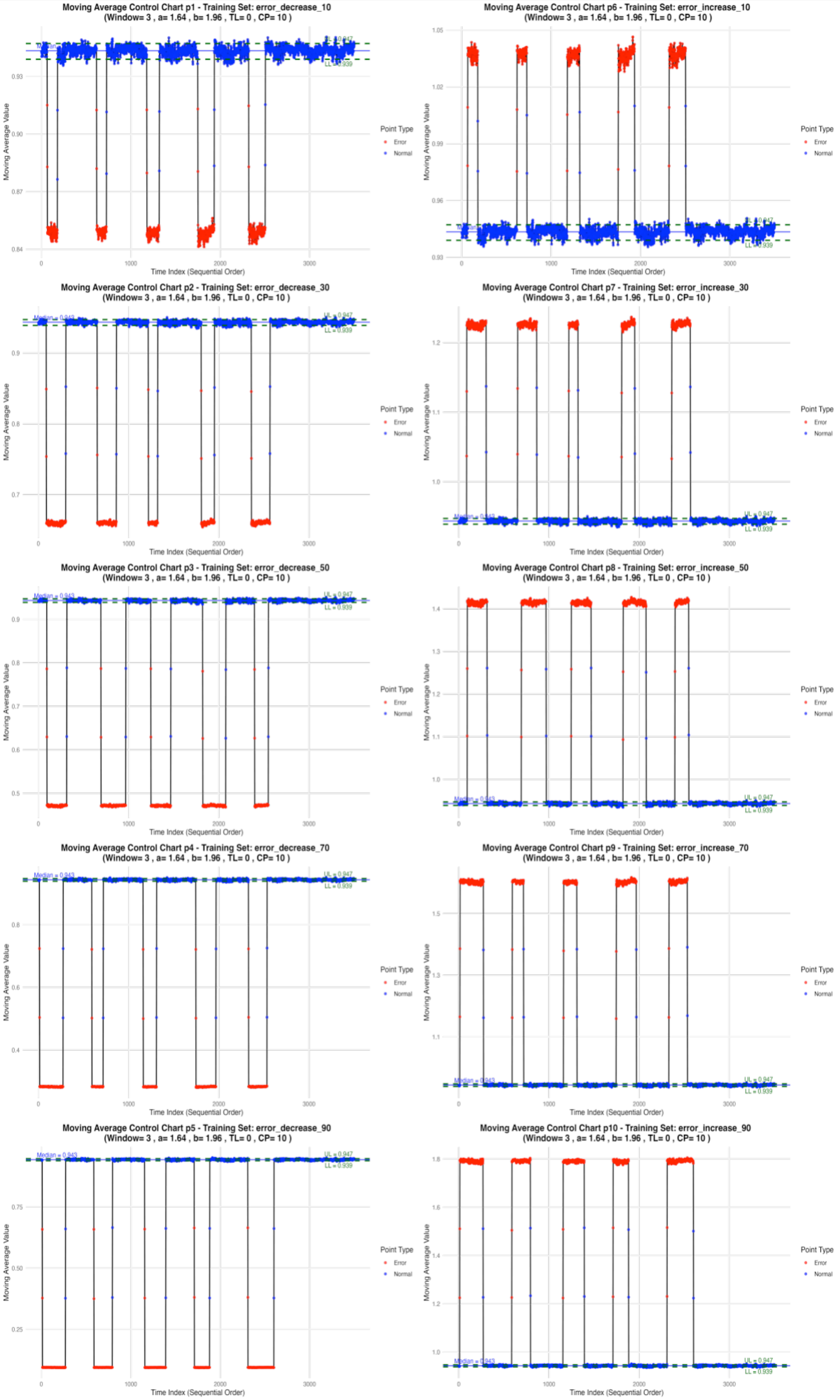

TT_outputMA - Test (p11-p20)

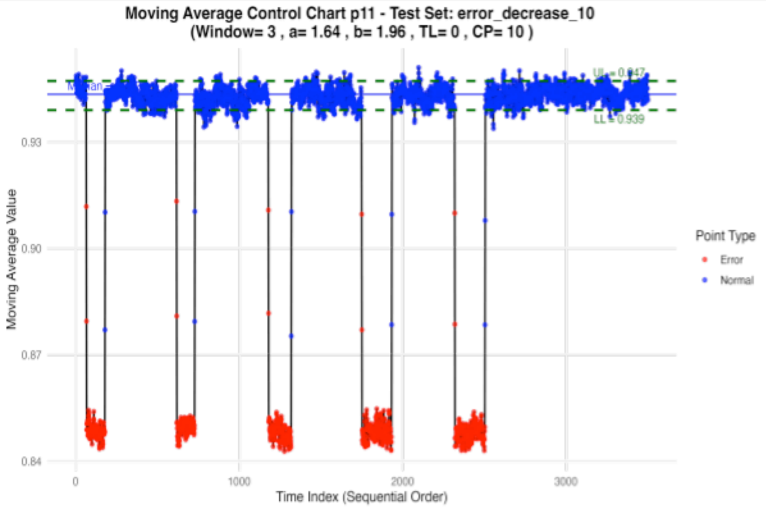

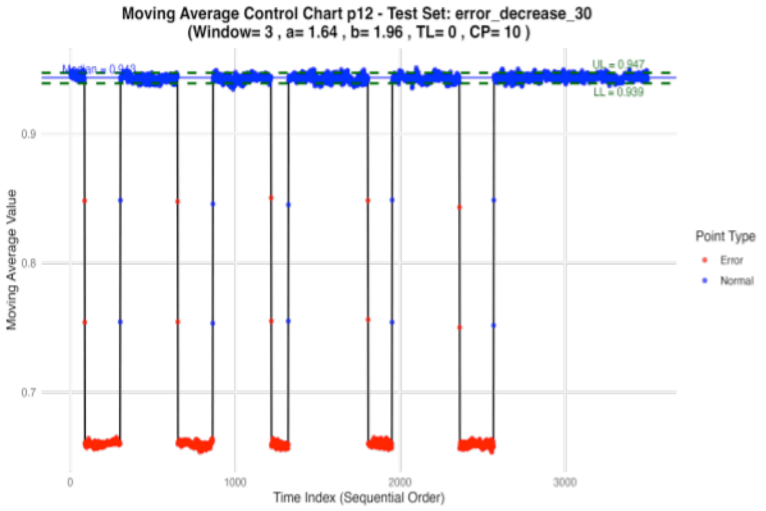

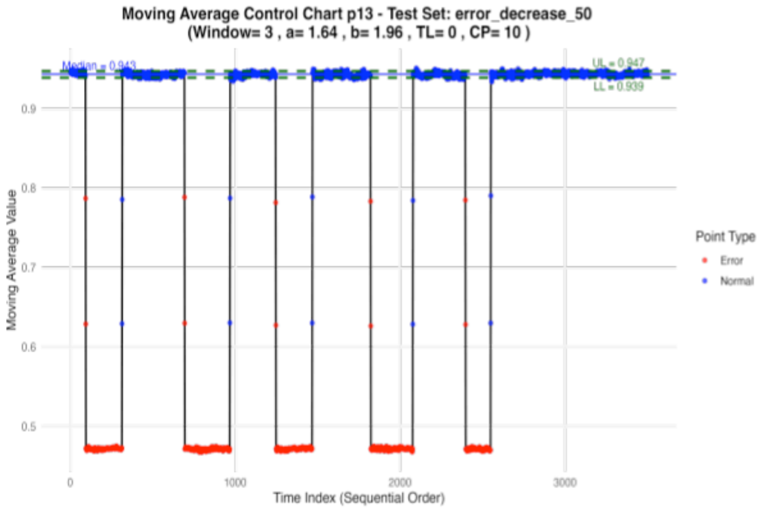

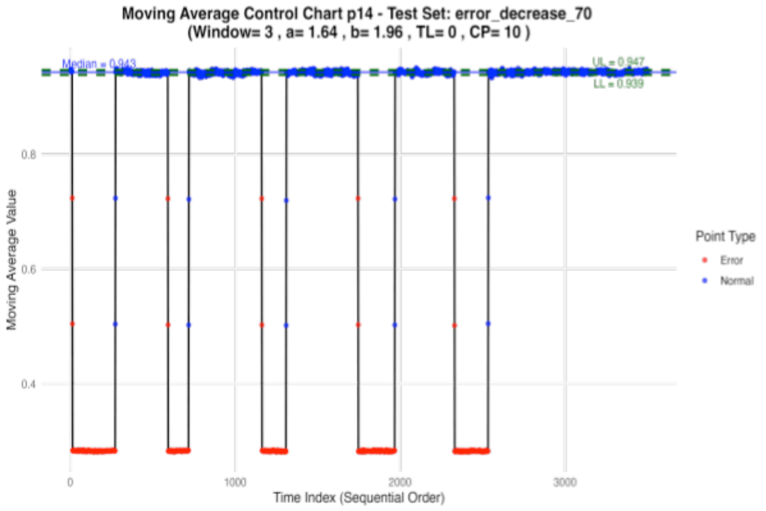

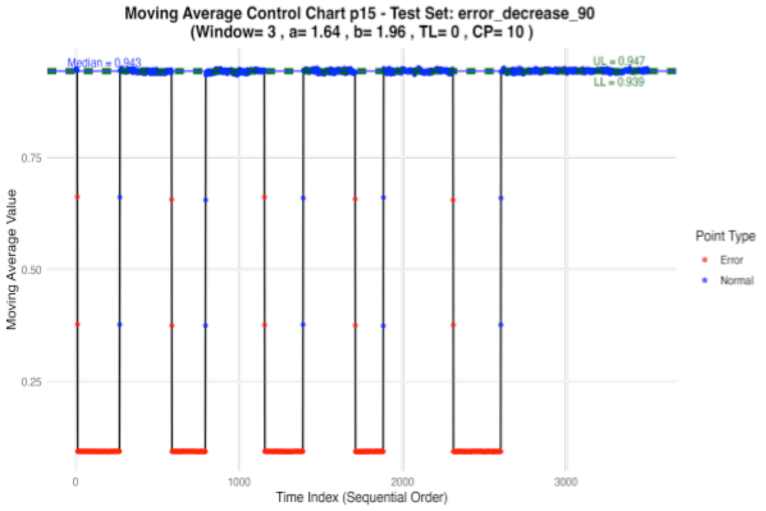

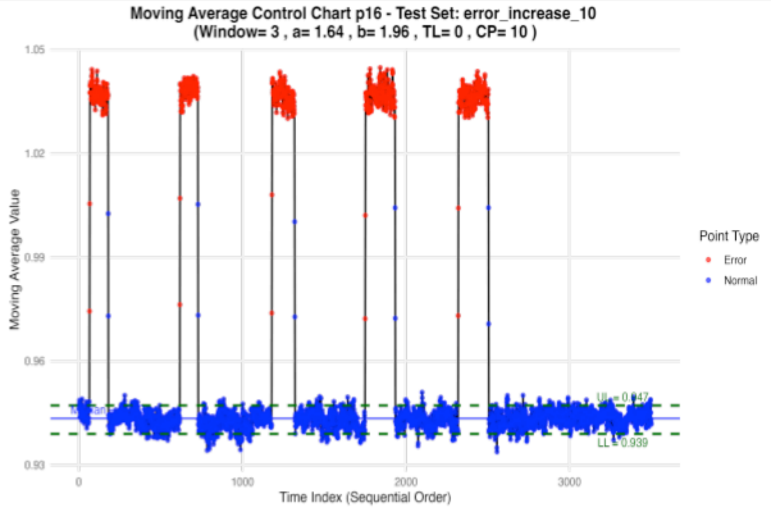

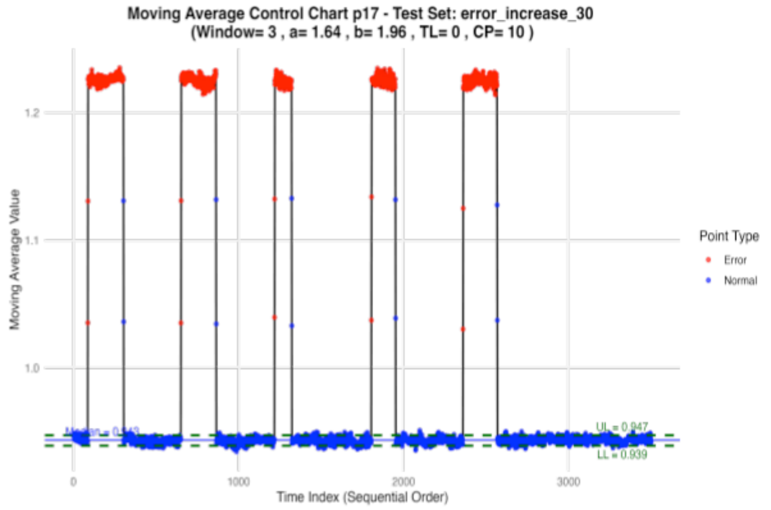

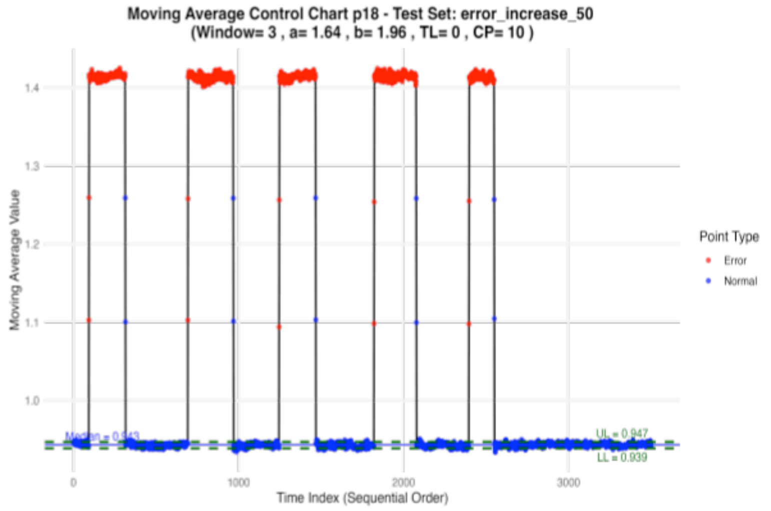

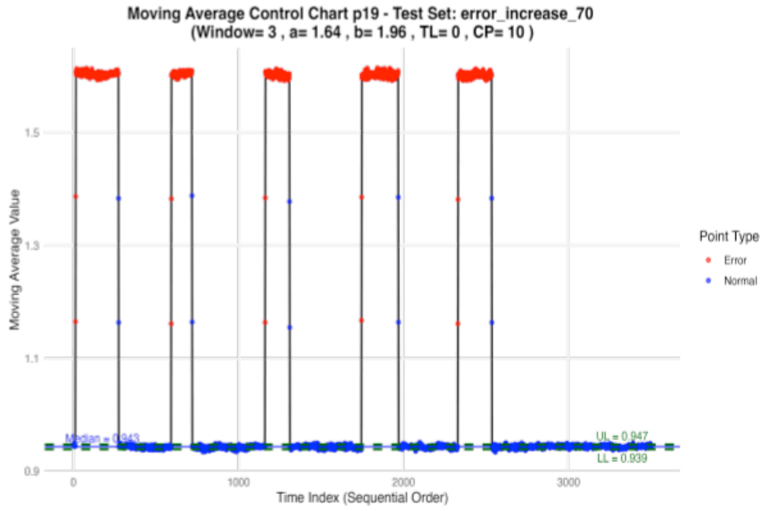

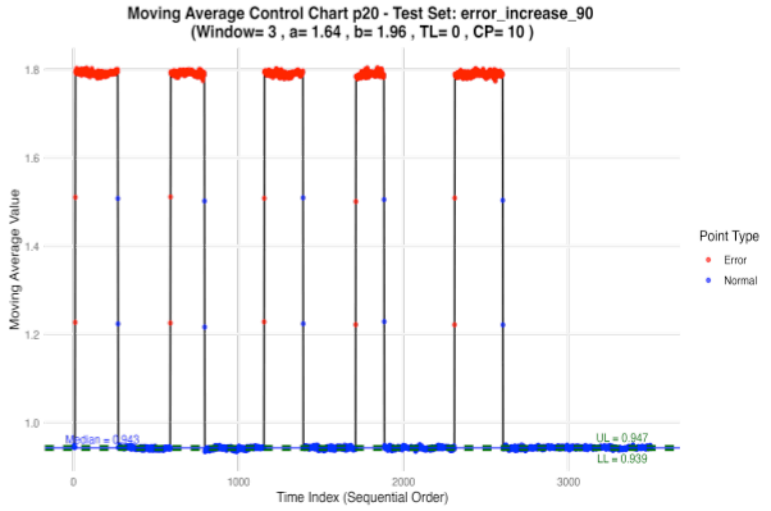

Supplement: Supplementary file 1 [file diagnostics-16-00288-s001.zip › Suppmental Figures S2-S36.pdf]
