# Supplementary material for: Comparison of EWMA, MA, and MQ Under a Unified PBRTQC Framework for Thyroid and Coagulation Tests
Source: Diagnostics (Basel). 2026 Jan 16;16(2):288. doi: 10.3390/diagnostics16020288 (PMC12839619; doi:10.3390/diagnostics16020288)

FT3 - Sensitivity Plots

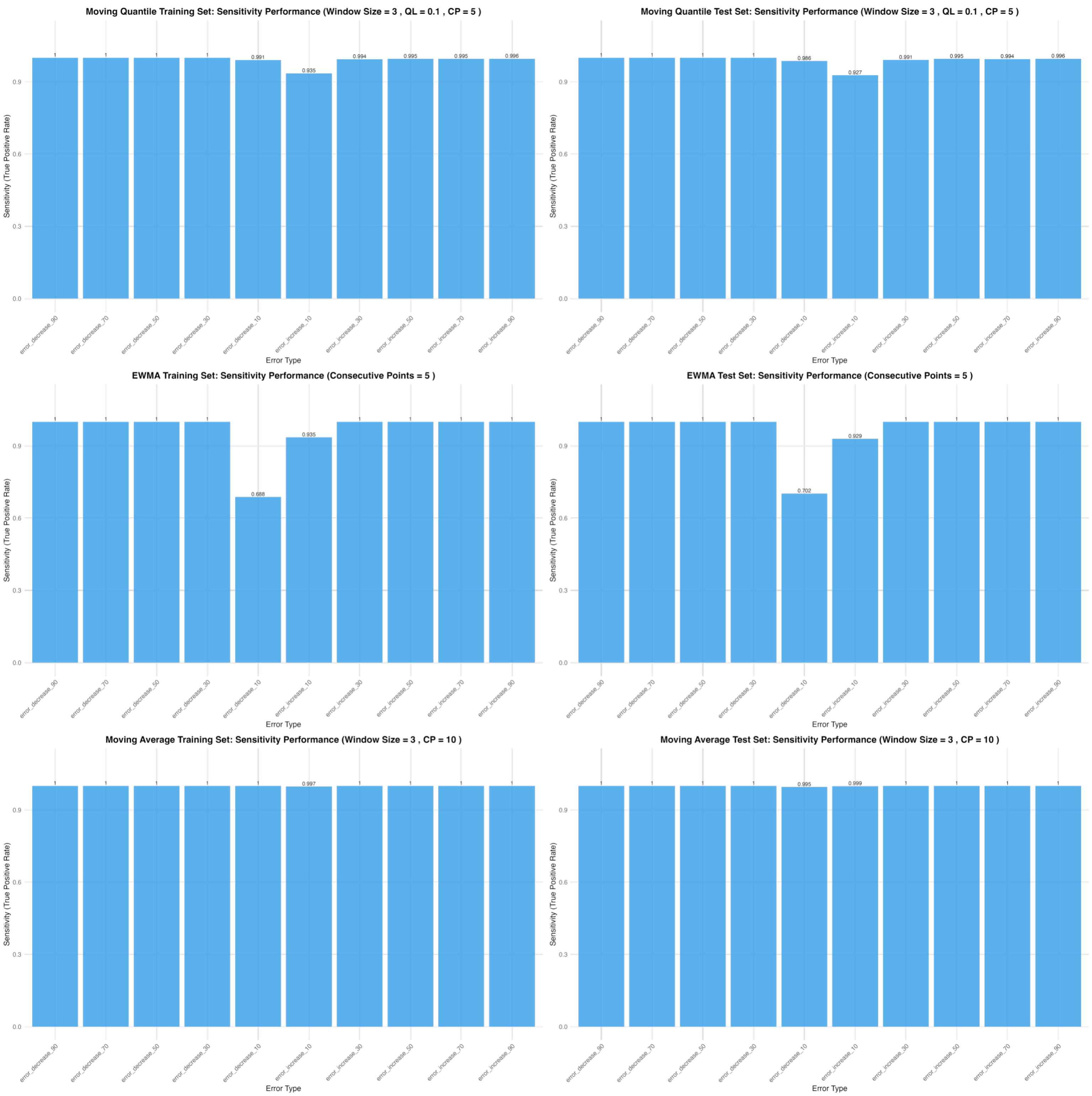

FT4 - Sensitivity Plots

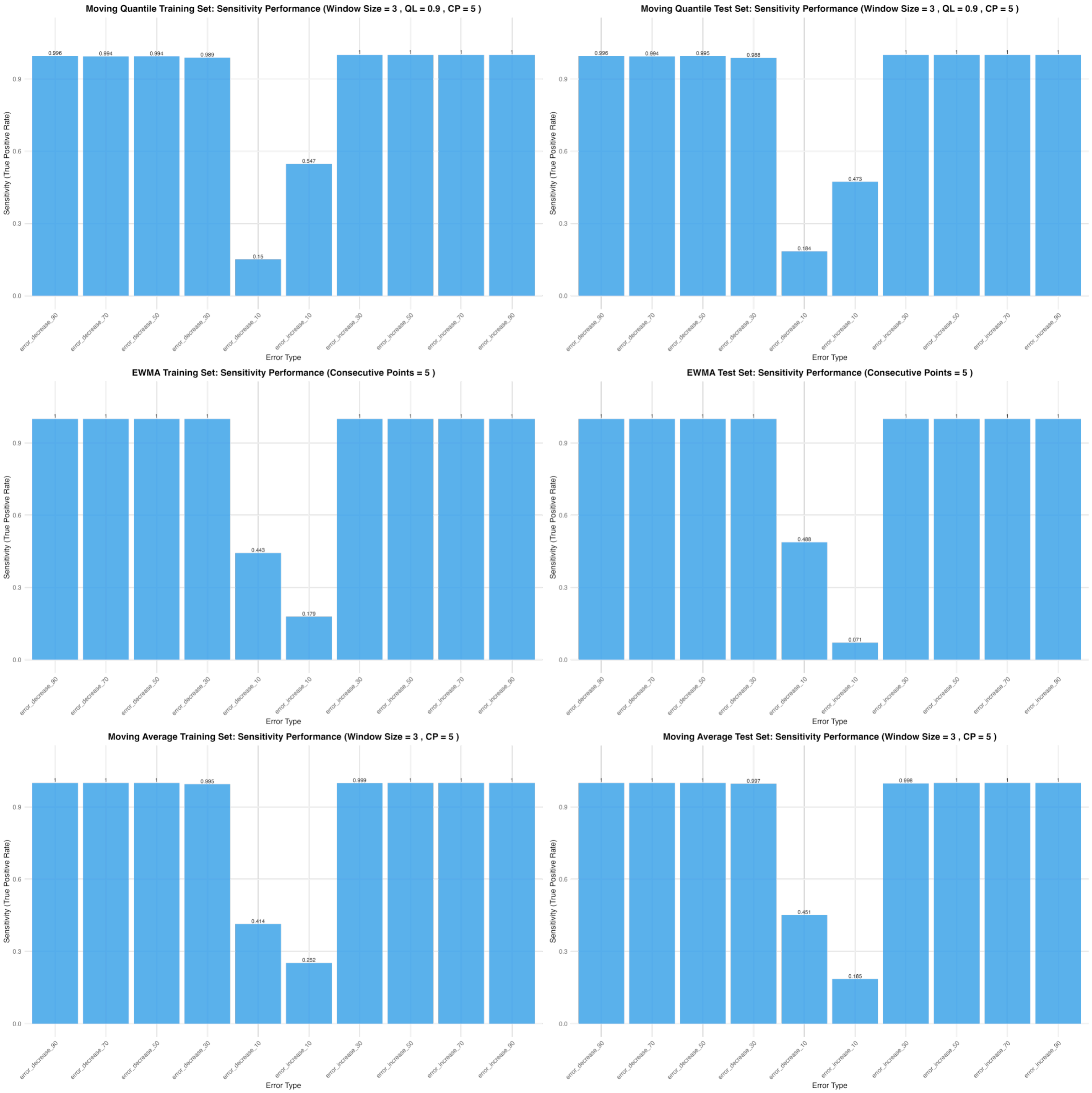

PT - Sensitivity Plots

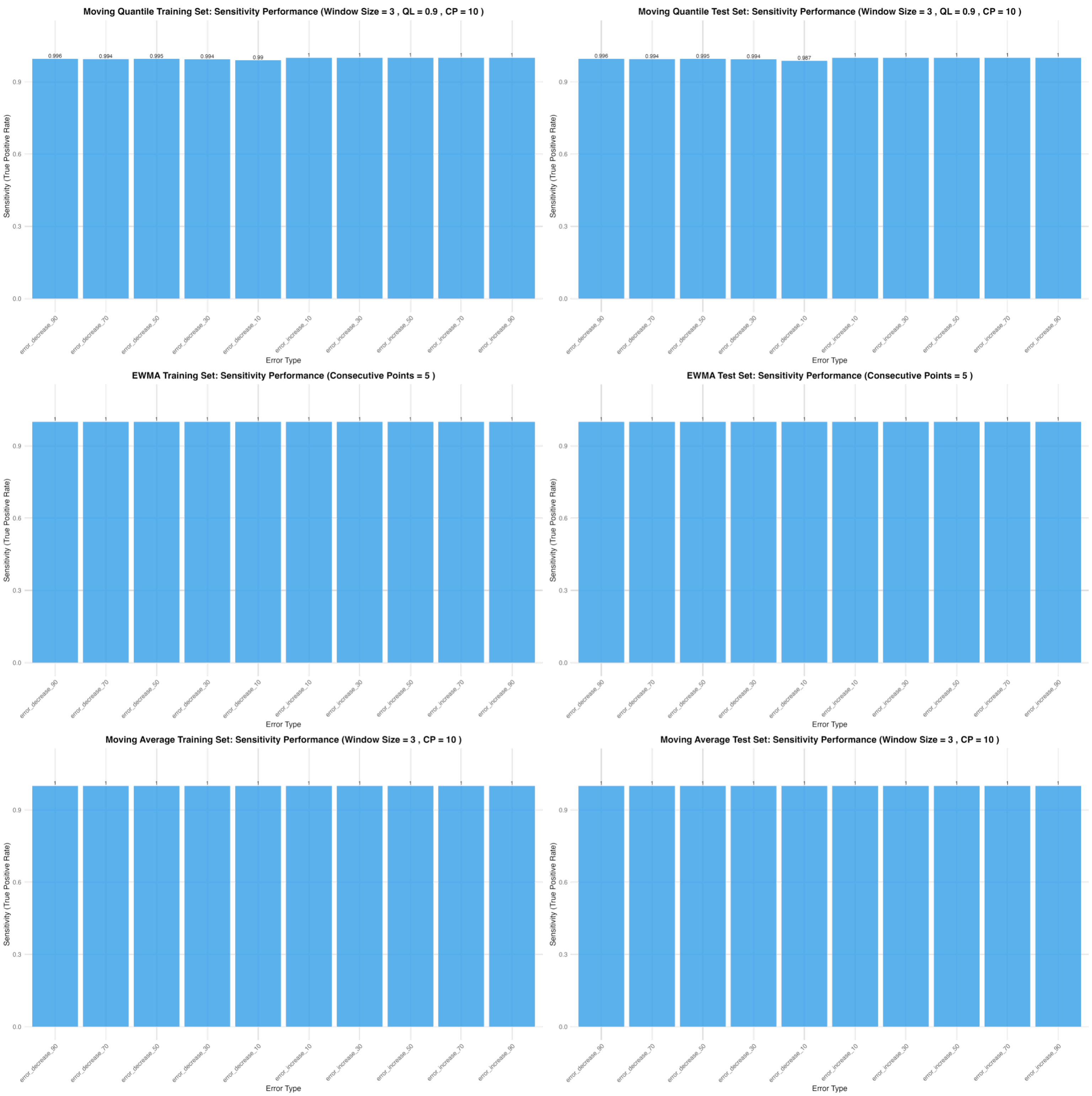

APTT - Sensitivity Plots

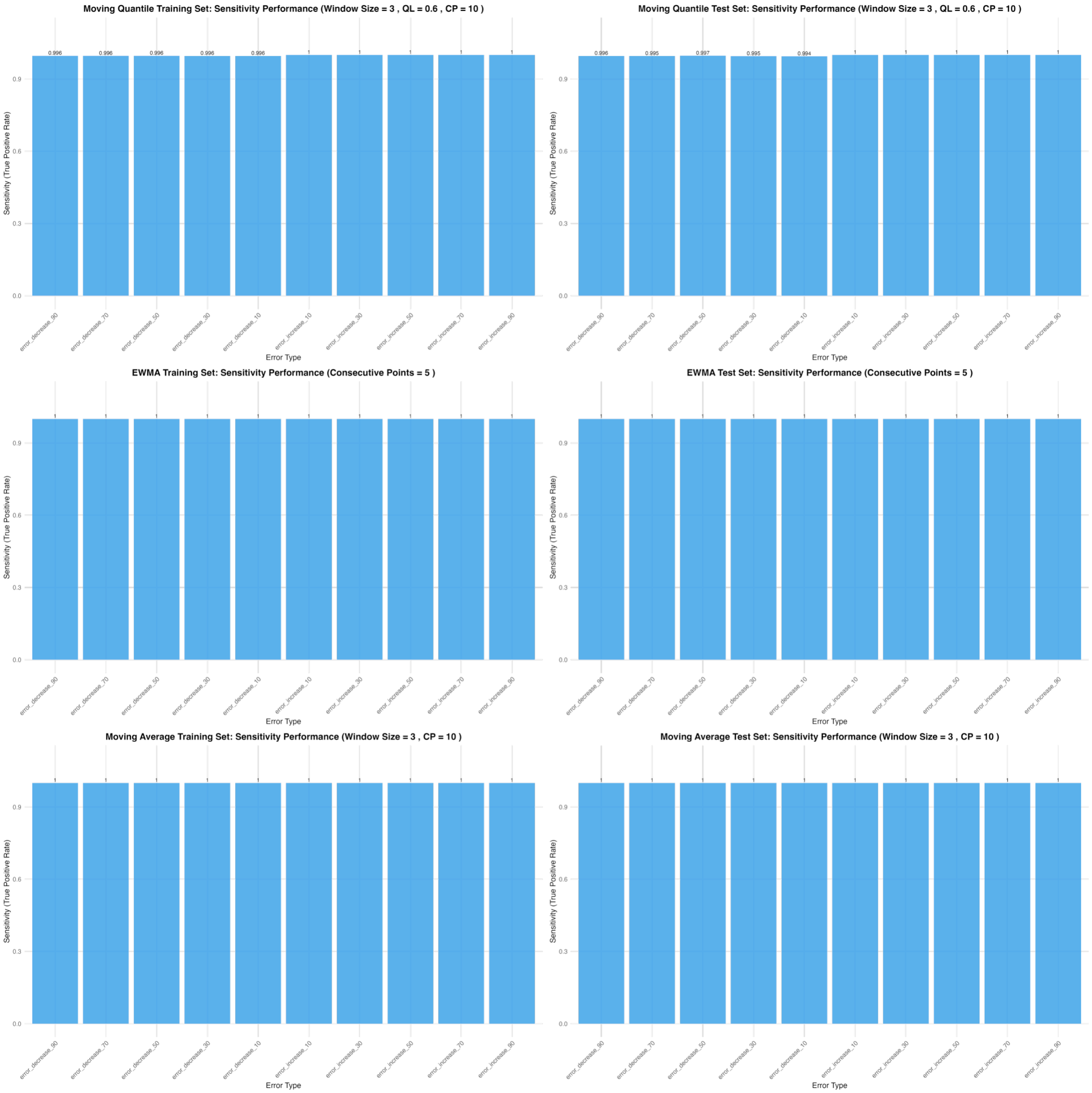

TT - Sensitivity Plots

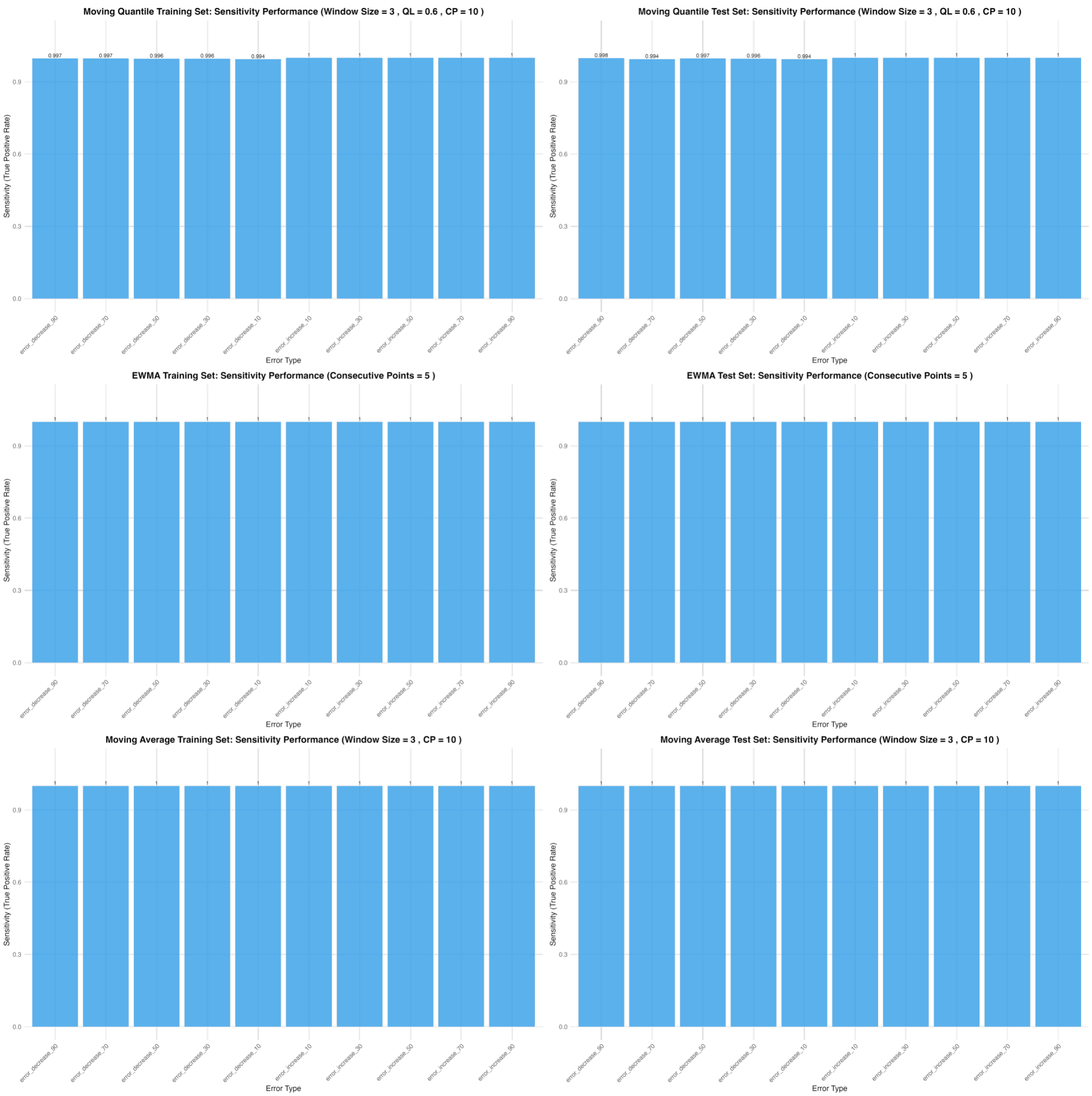

Supplement: Supplementary file 1 [file diagnostics-16-00288-s001.zip › Suppmental Figures S37-S41.pdf]
