# Supplementary material for: Comparison of EWMA, MA, and MQ Under a Unified PBRTQC Framework for Thyroid and Coagulation Tests
Source: Diagnostics (Basel). 2026 Jan 16;16(2):288. doi: 10.3390/diagnostics16020288 (PMC12839619; doi:10.3390/diagnostics16020288)
Supplement: Supplementary file 1 [file diagnostics-16-00288-s001.zip › Suppmental Figures S42-S46.pdf]

# FT3 - FPR Plots

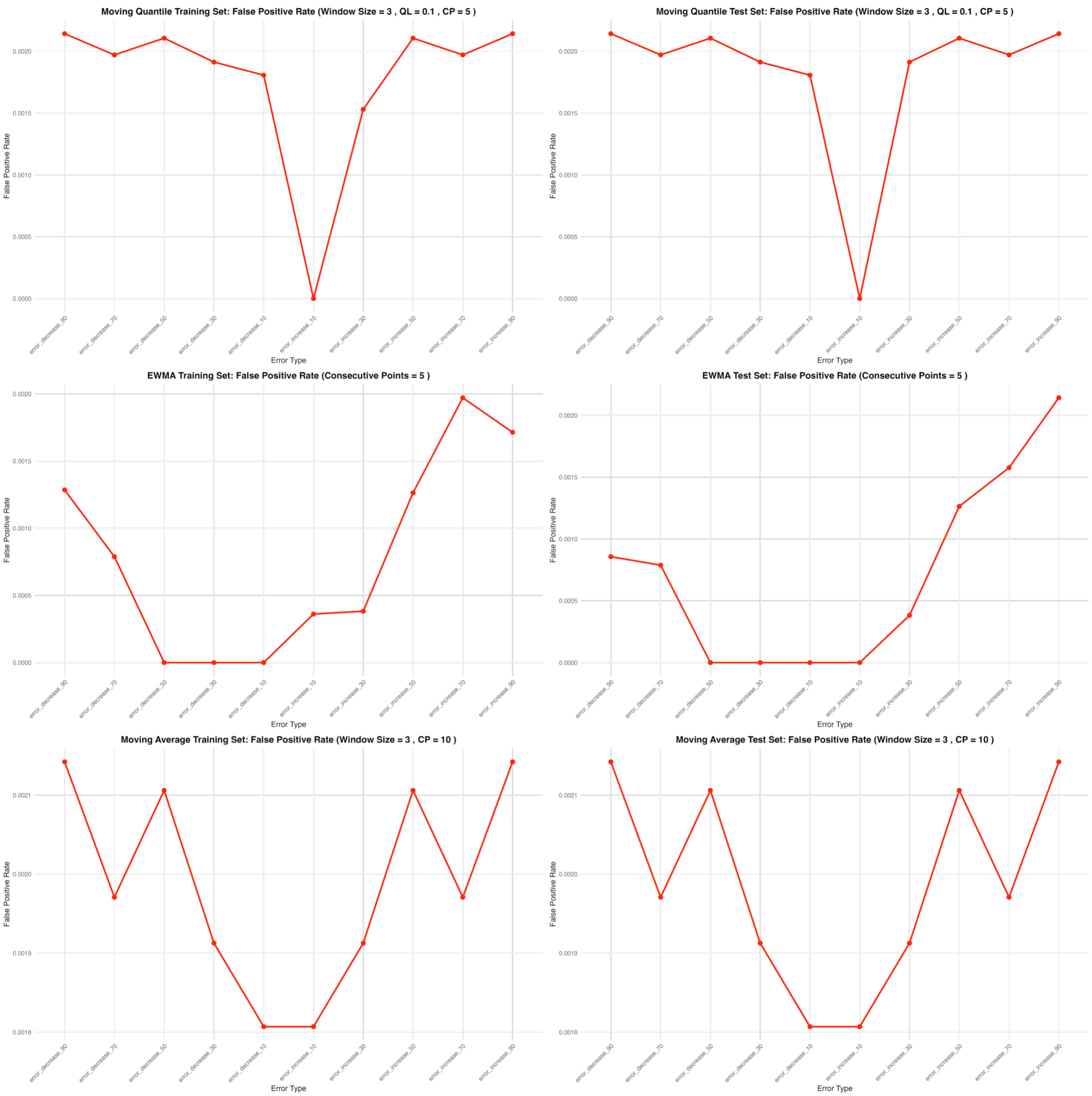

**FT4 - FPR Plots**

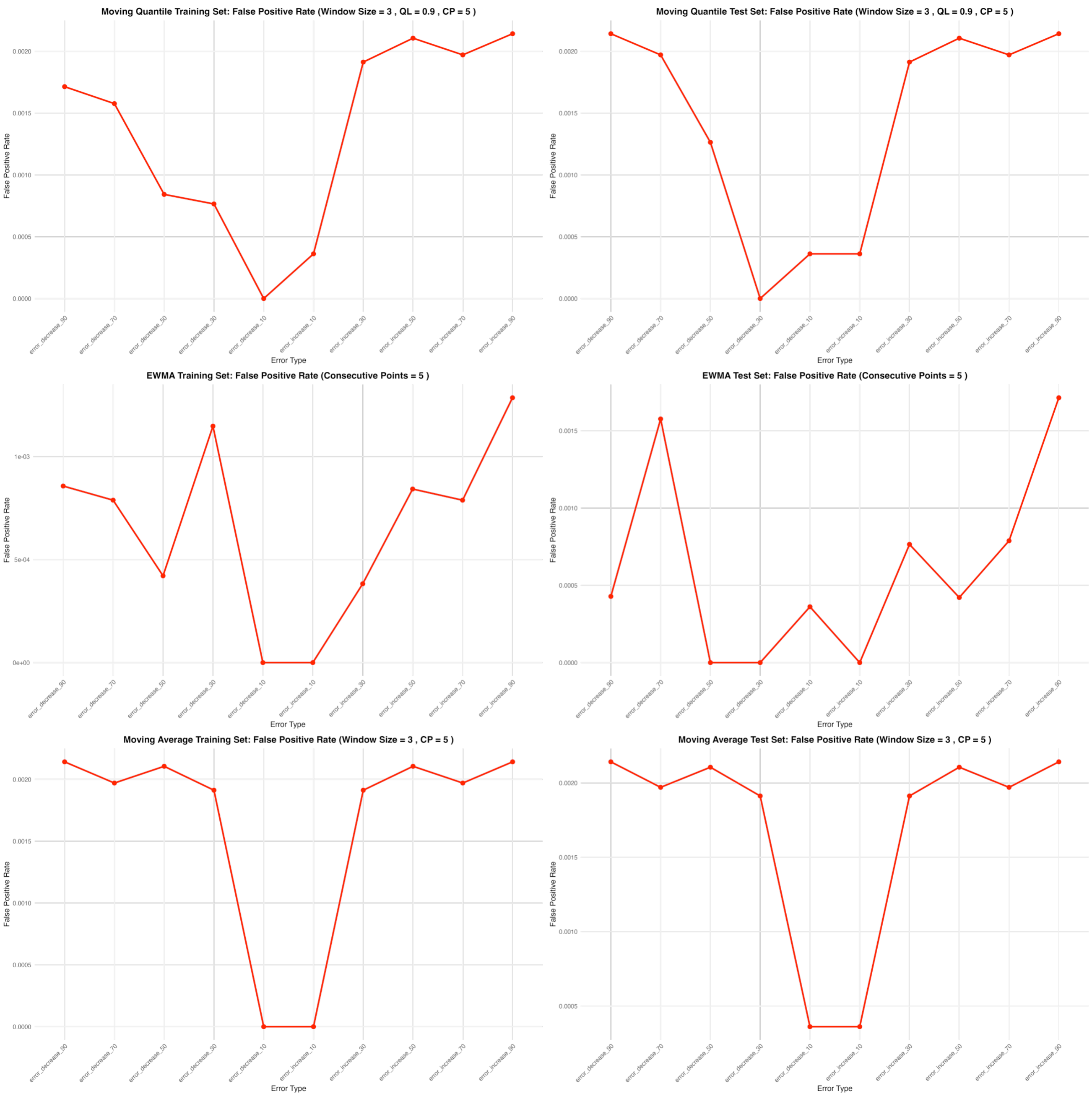

**PT - FPR Plots**

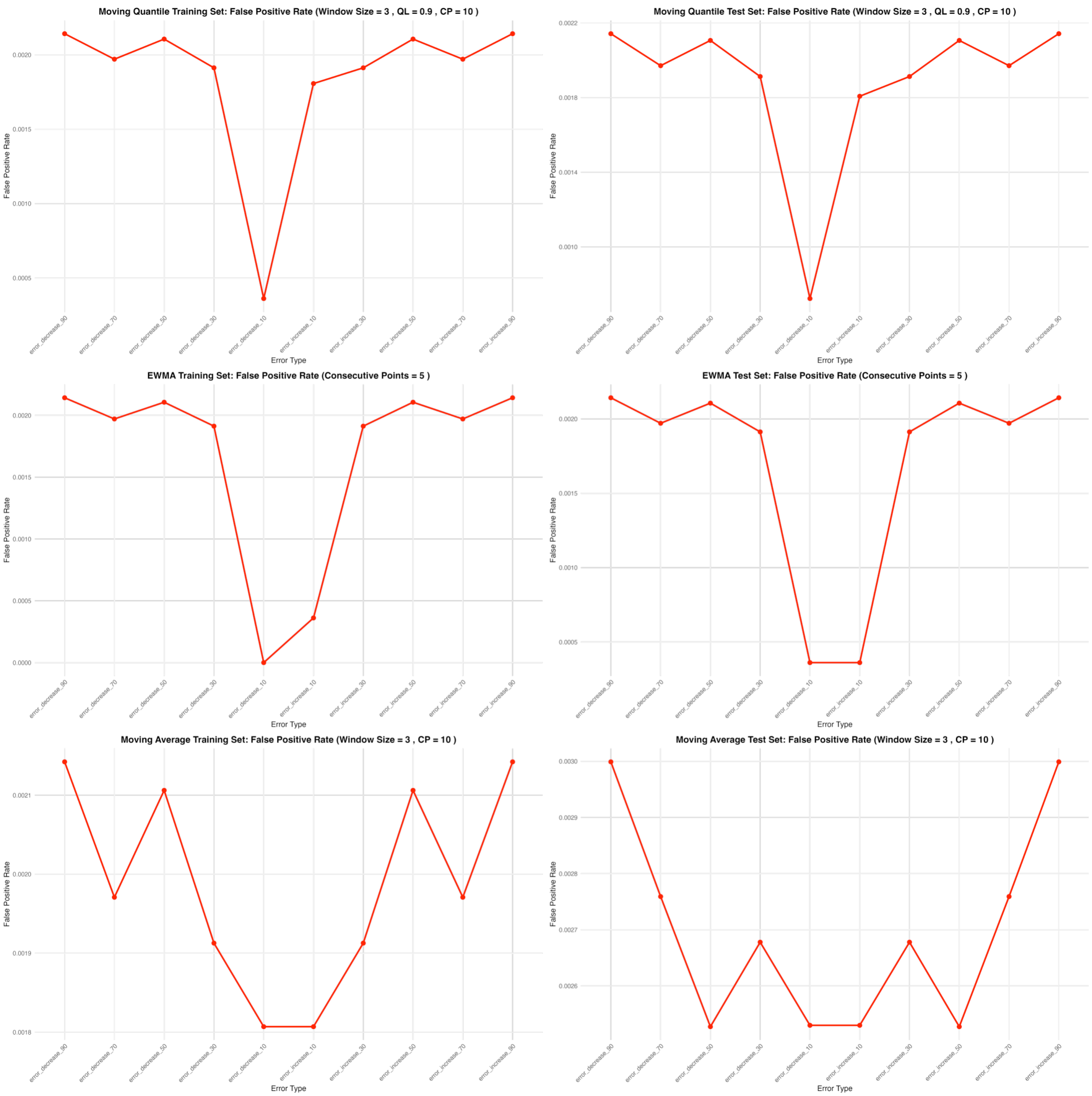
Moving Quantile Training Set: False Positive Rate (Window Size = 3 , QL = 0.9 , CP = 10 )
Moving Quantile Test Set: False Positive Rate (Window Size = 3 , QL = 0.9 , CP = 10 )
EWMA Training Set: False Positive Rate (Consecutive Points = 5 )
EWMA Test Set: False Positive Rate (Consecutive Points = 5 )
Moving Average Training Set: False Positive Rate (Window Size = 3 , CP = 10 )
Moving Average Test Set: False Positive Rate (Window Size = 3 , CP = 10 )
False Positive Rate
Error Type
error_decrease_90
error_decrease_70
error_decrease_50
error_decrease_30
error_decrease_10
error_increase_10
error_increase_30
error_increase_50
error_increase_70
error_increase_90

## APTT - FPR Plots

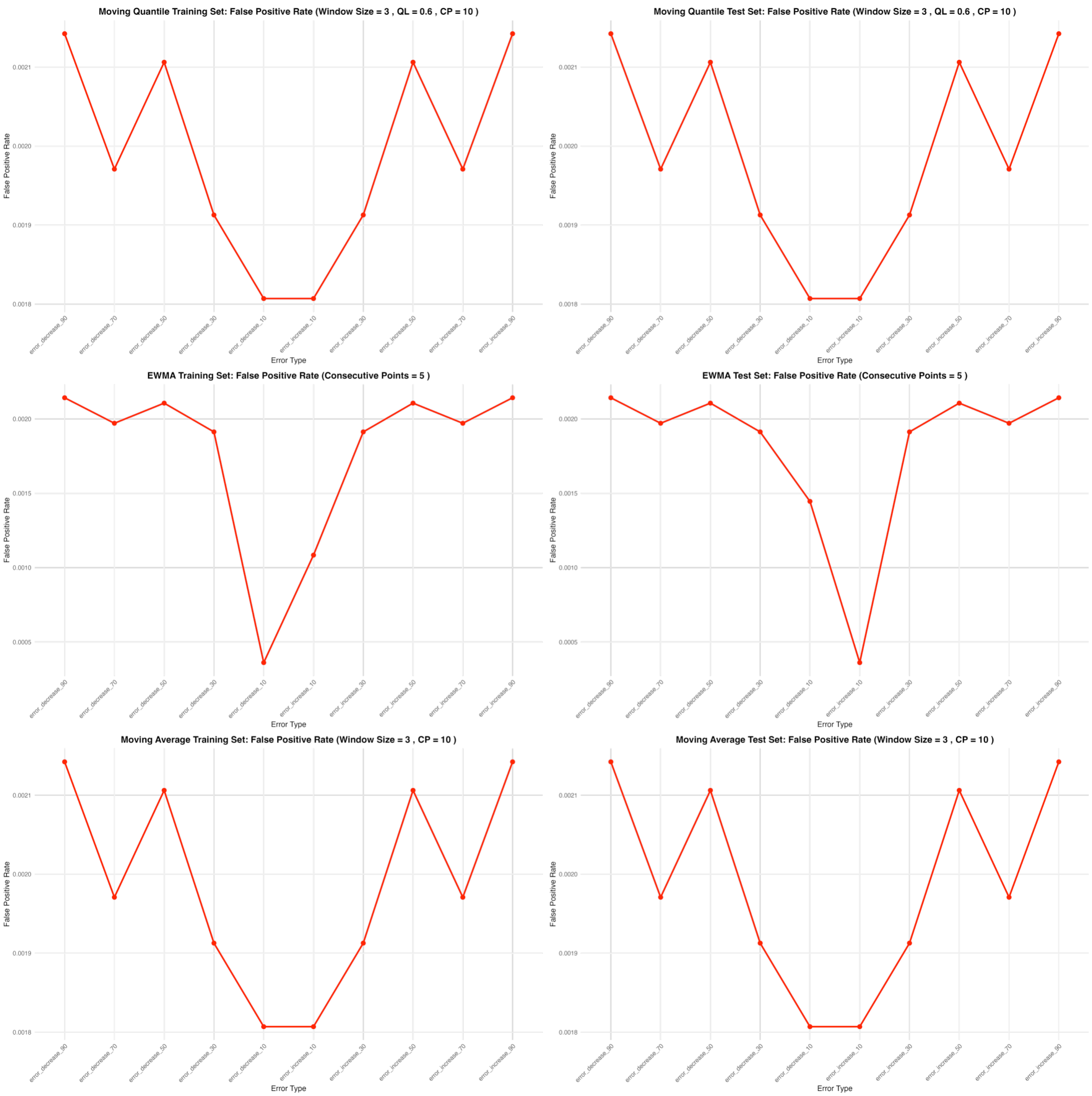

## TT - FPR Plots

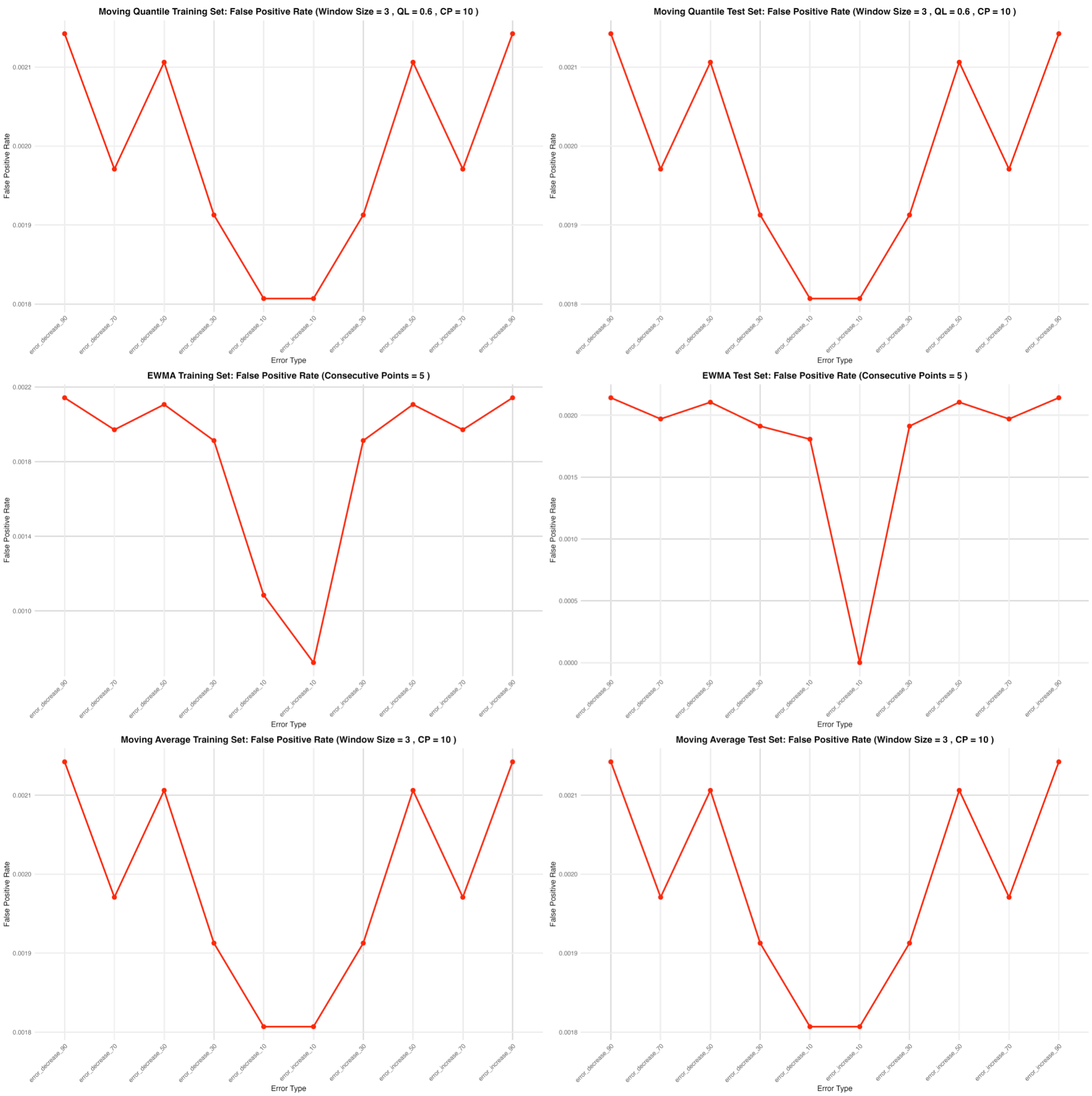
Moving Quantile Training Set: False Positive Rate (Window Size = 3 , QL = 0.6 , CP = 10 )
Moving Quantile Test Set: False Positive Rate (Window Size = 3 , QL = 0.6 , CP = 10 )
EWMA Training Set: False Positive Rate (Consecutive Points = 5 )
EWMA Test Set: False Positive Rate (Consecutive Points = 5 )
Moving Average Training Set: False Positive Rate (Window Size = 3 , CP = 10 )
Moving Average Test Set: False Positive Rate (Window Size = 3 , CP = 10 )
False Positive Rate
Error Type
0.0018
0.0019
0.0020
0.0021
0.0010
0.0014
0.0022
0.0000
0.0005
0.0015
error_decrease_90
error_decrease_70
error_decrease_50
error_decrease_30
error_decrease_10
error_increase_10
error_increase_30
error_increase_50
error_increase_70
error_increase_90
