# Supplementary figures and images for: Comparison of EWMA, MA, and MQ Under a Unified PBRTQC Framework for Thyroid and Coagulation Tests
Source: Diagnostics (Basel). 2026 Jan 16;16(2):288. doi: 10.3390/diagnostics16020288 (PMC12839619; doi:10.3390/diagnostics16020288)

TSH - MNPED Plots

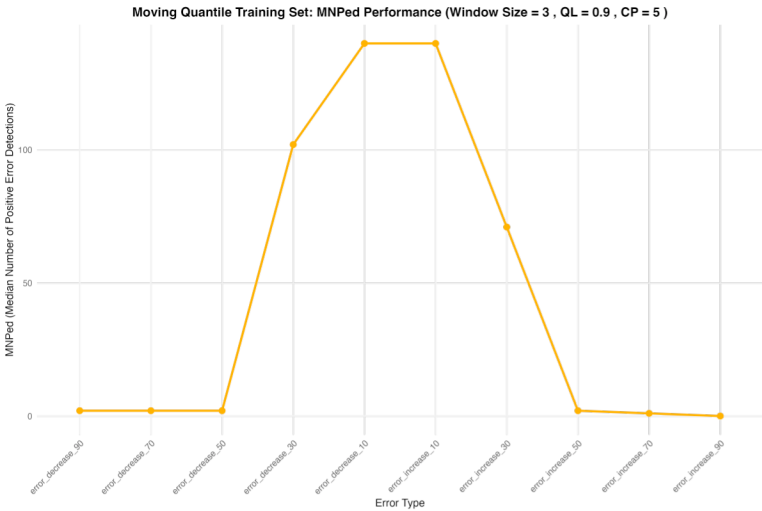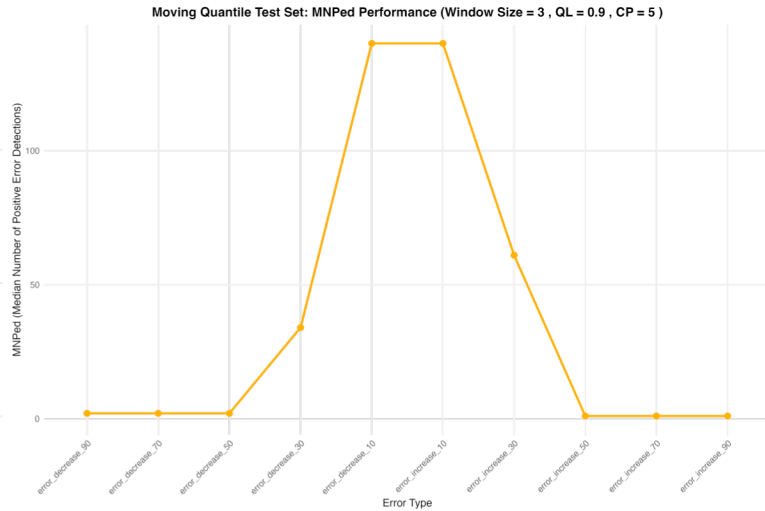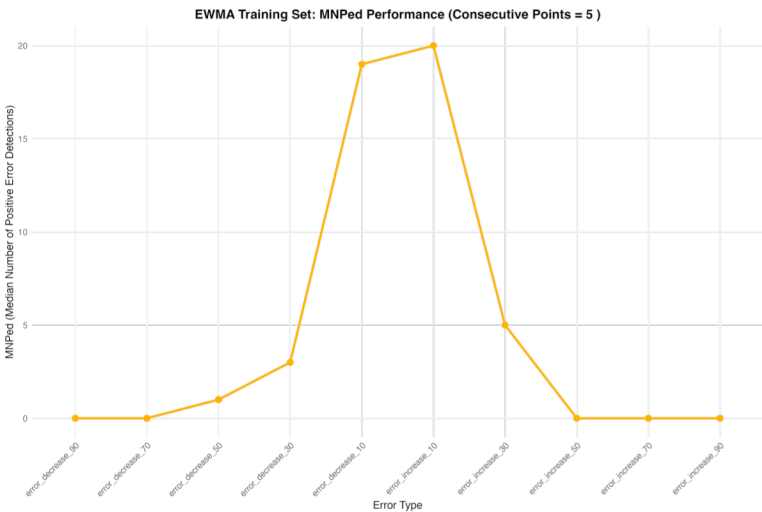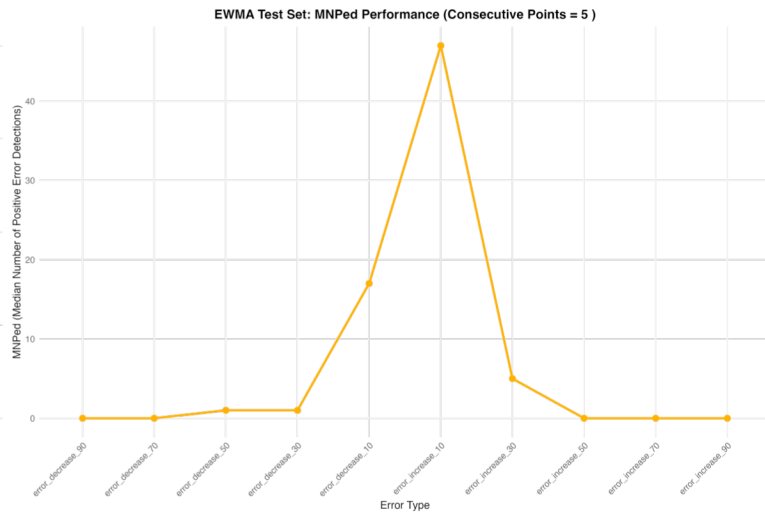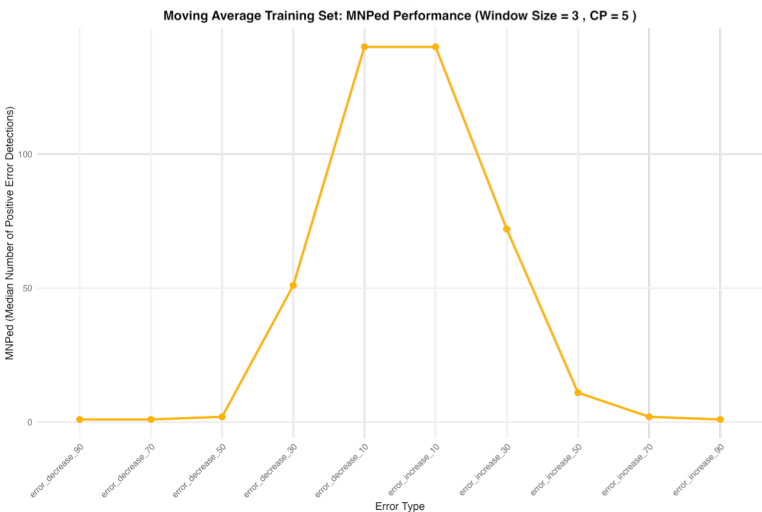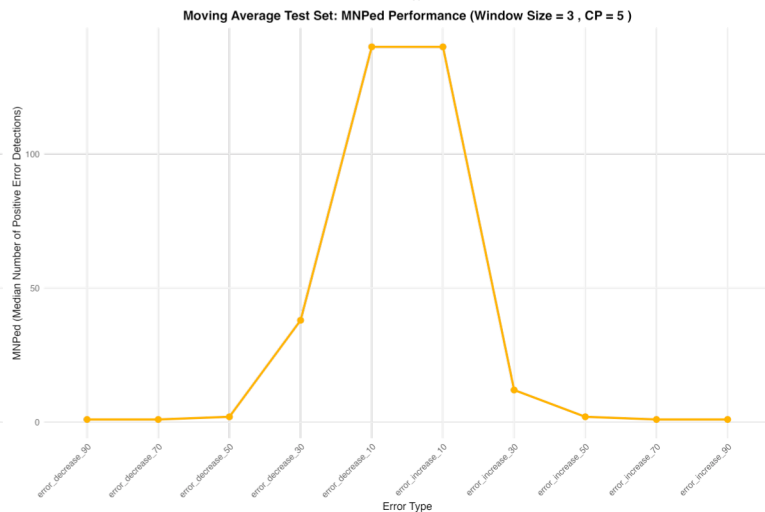

FT3 - MNPED Plots

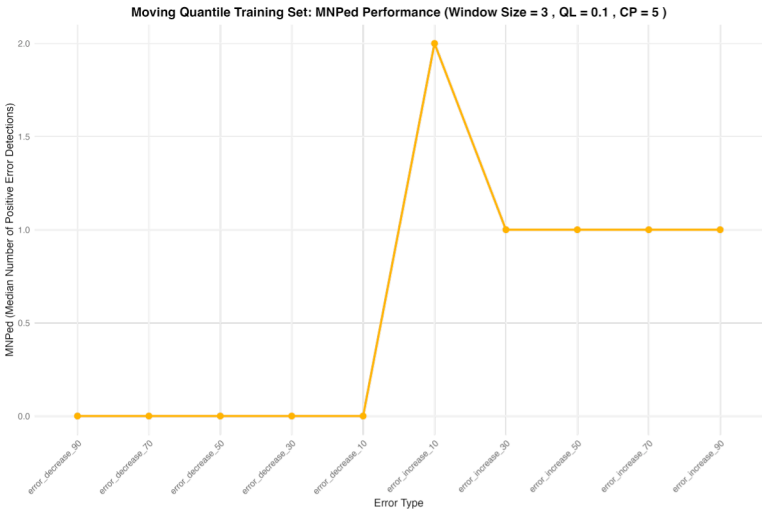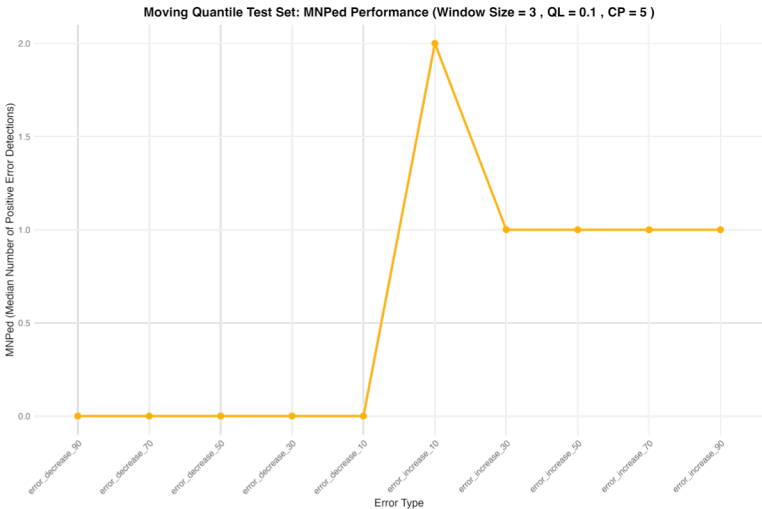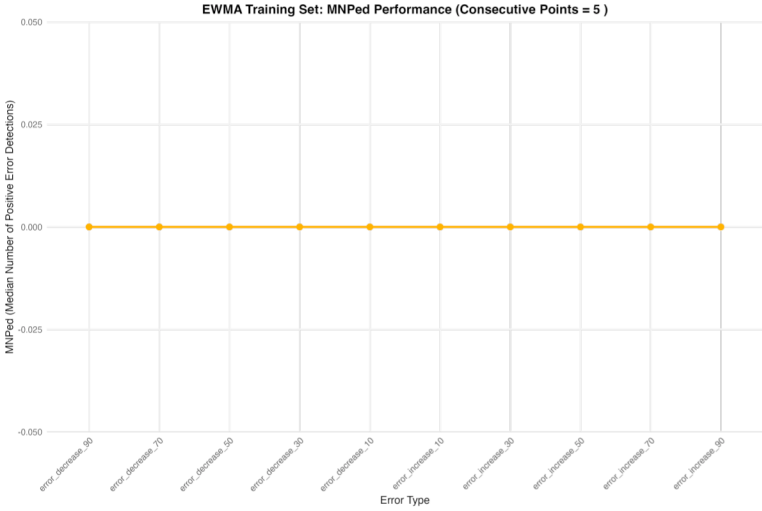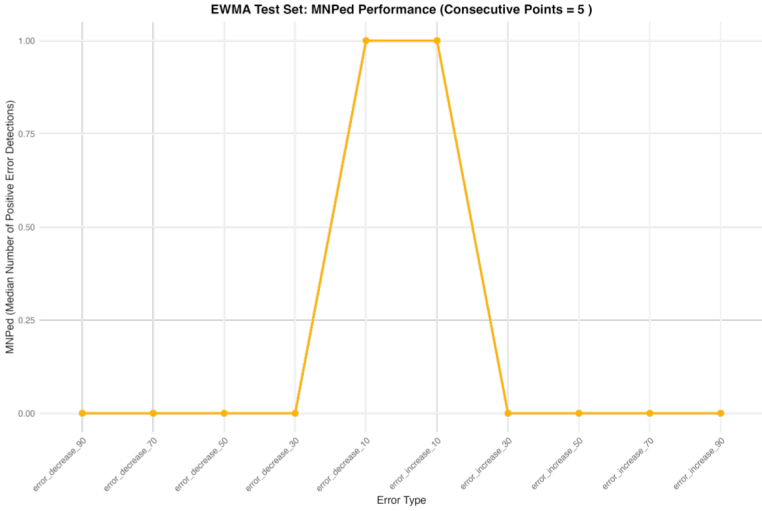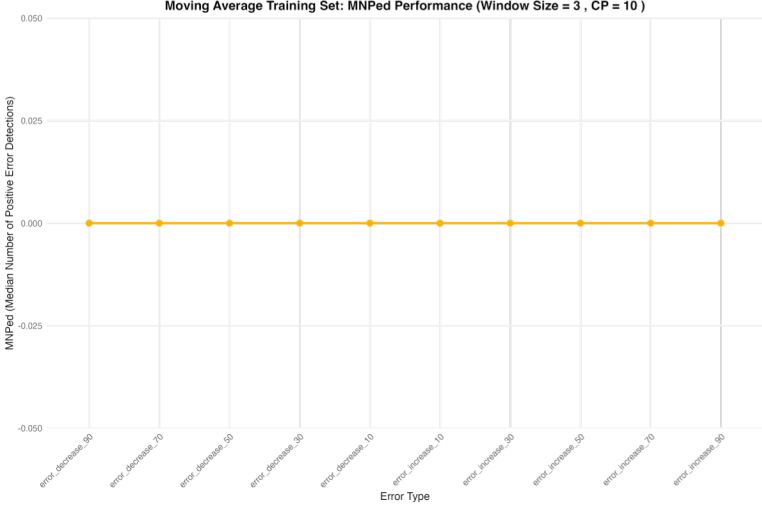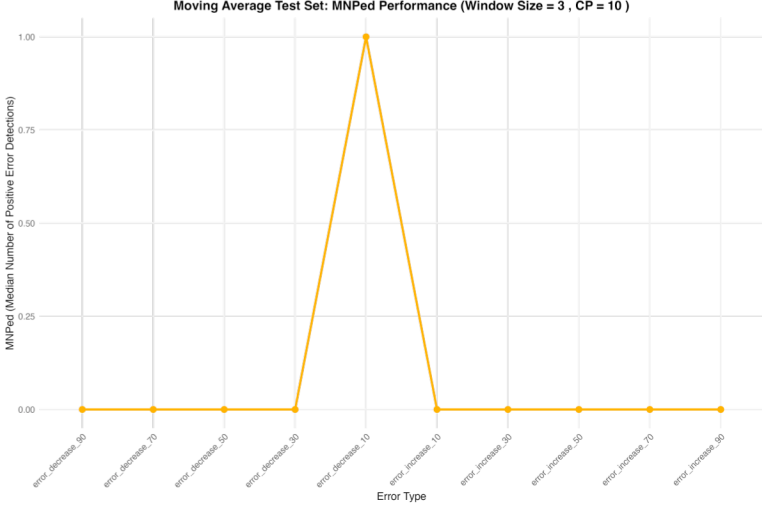

FT4 - MNPED Plots

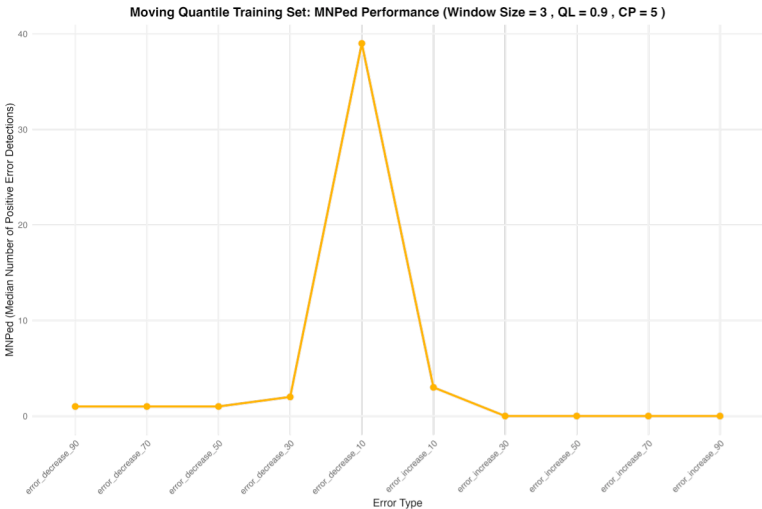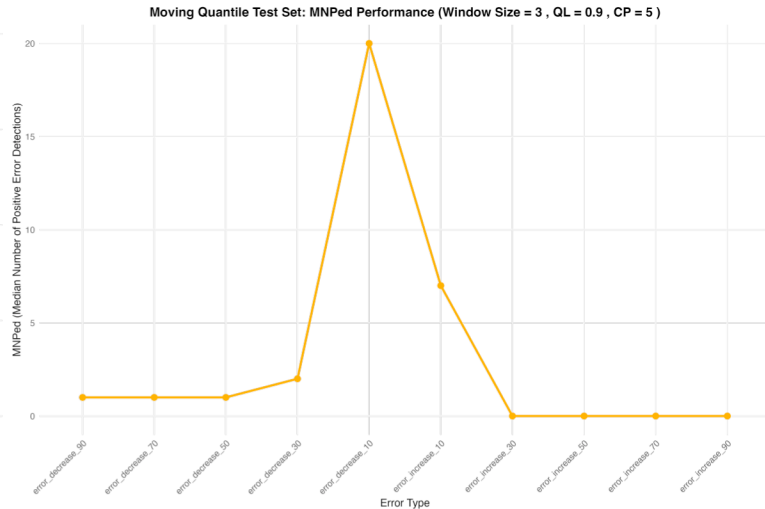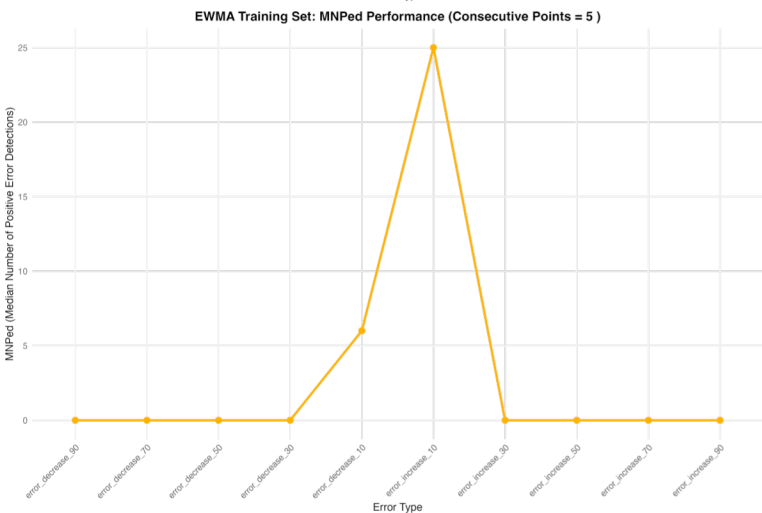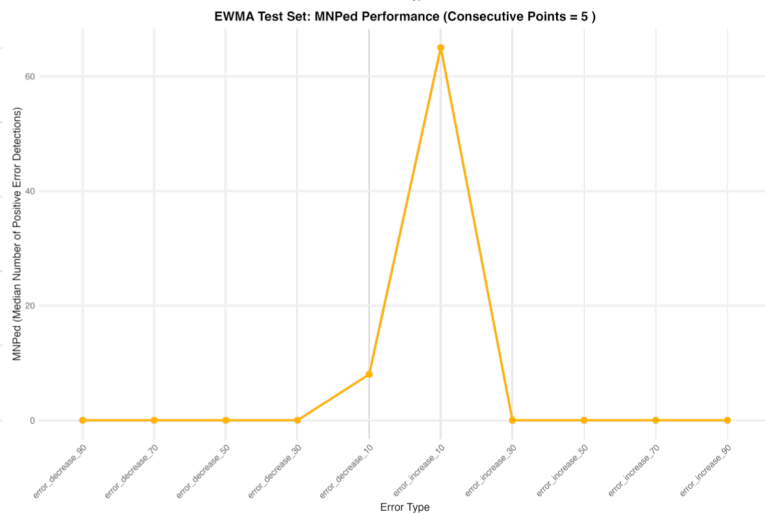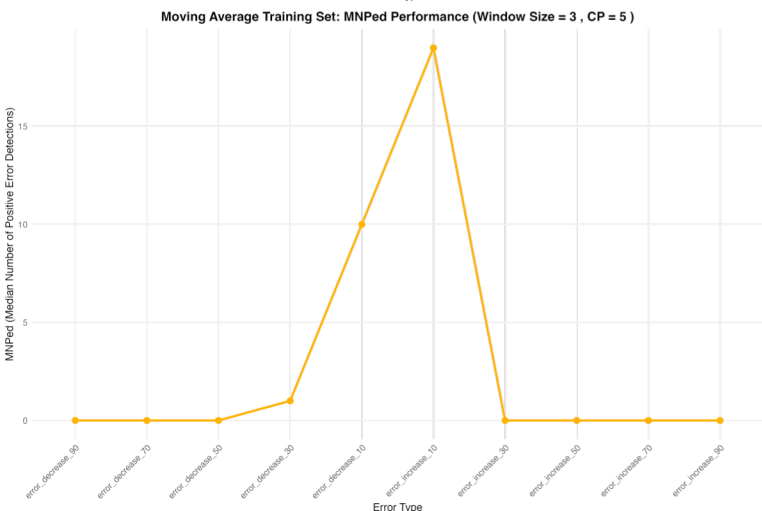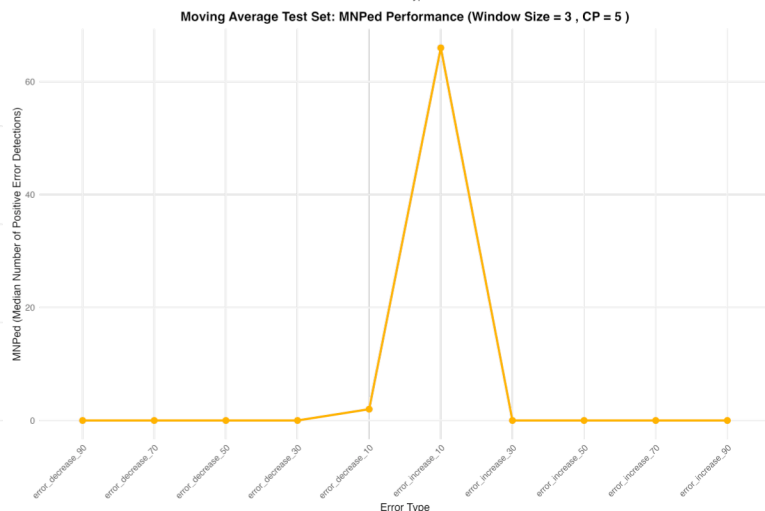

# PT - MNPED Plots

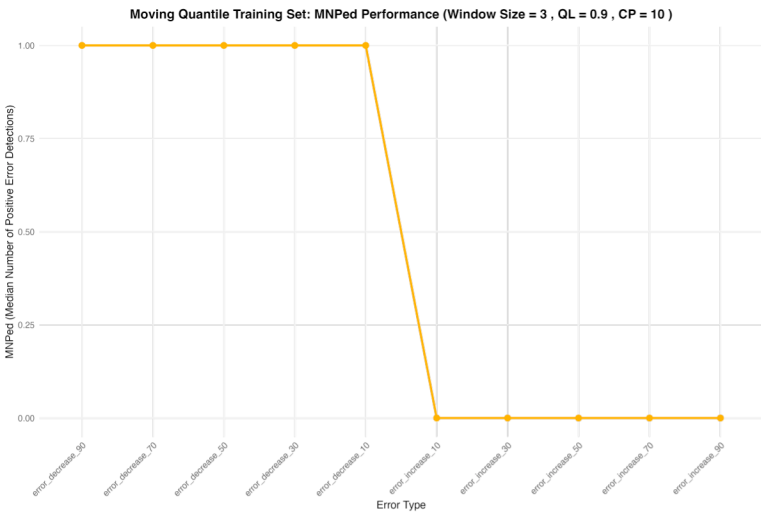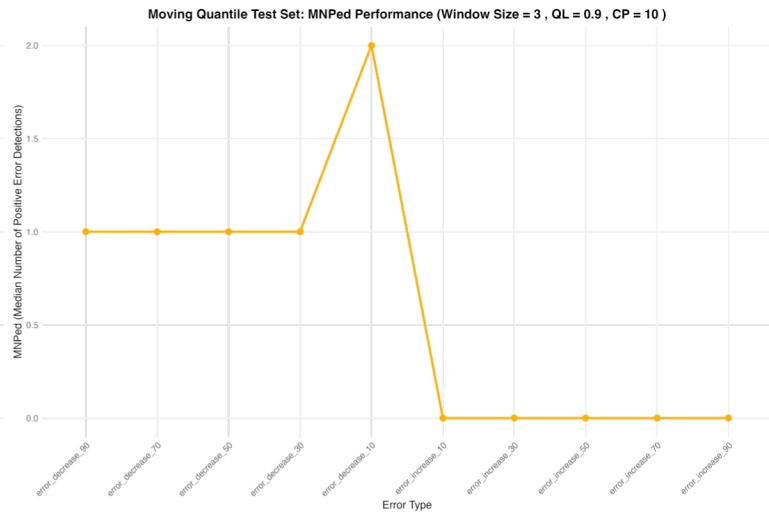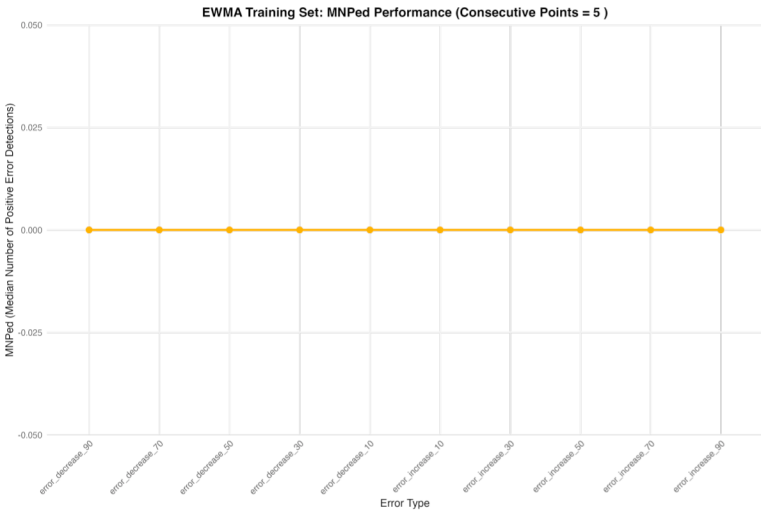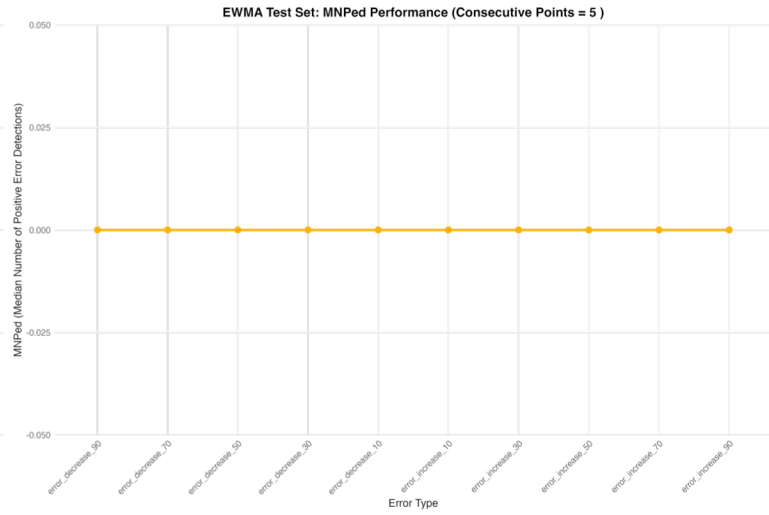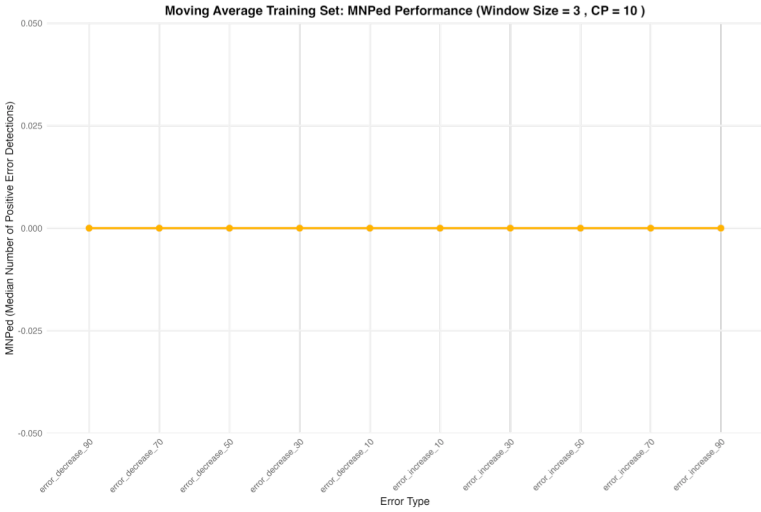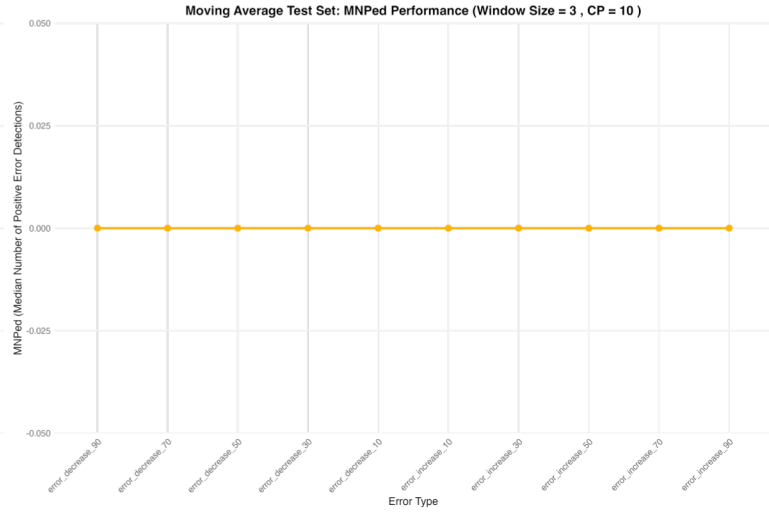

# APTT - MNPED Plots

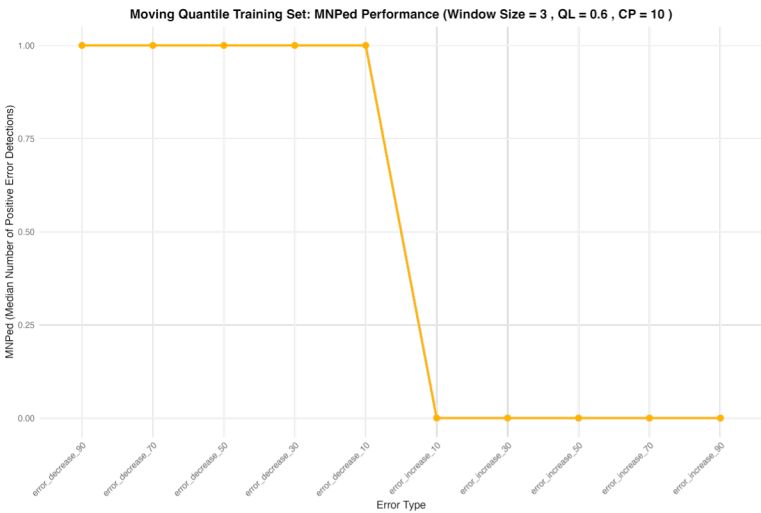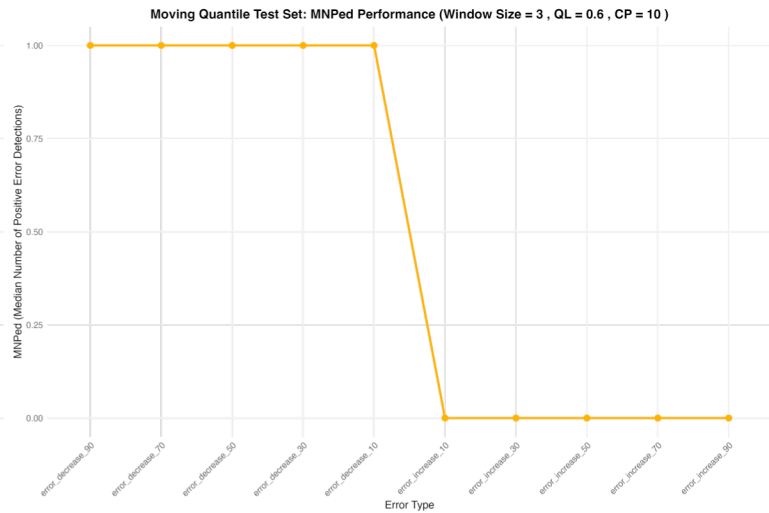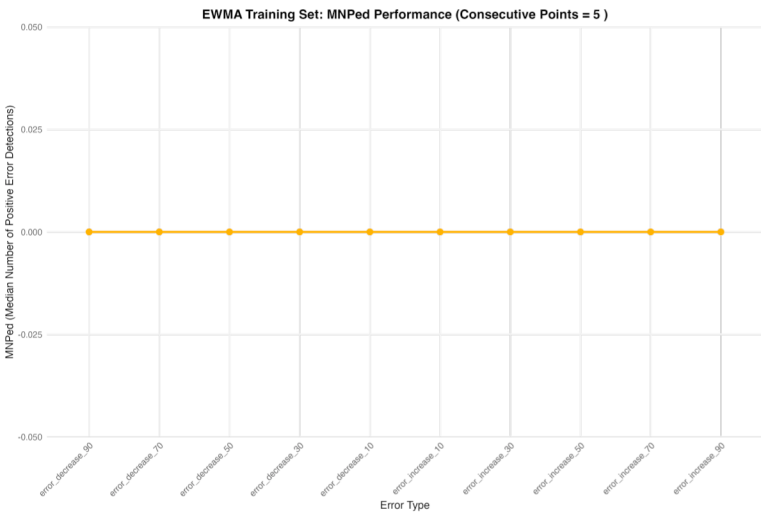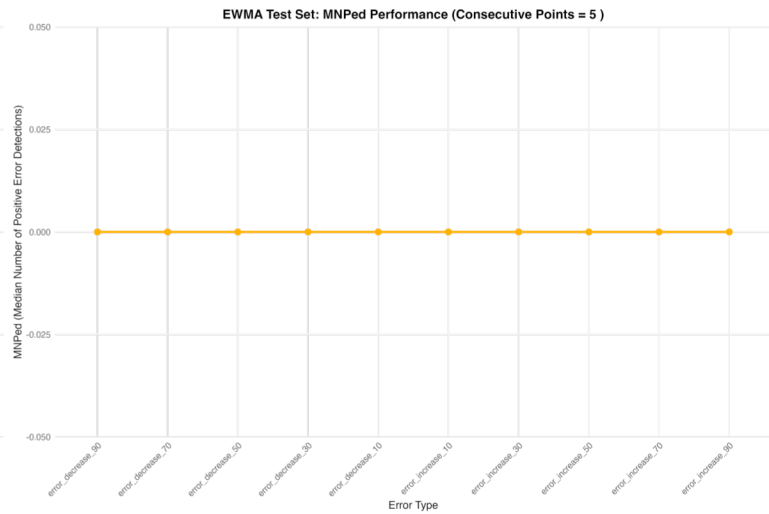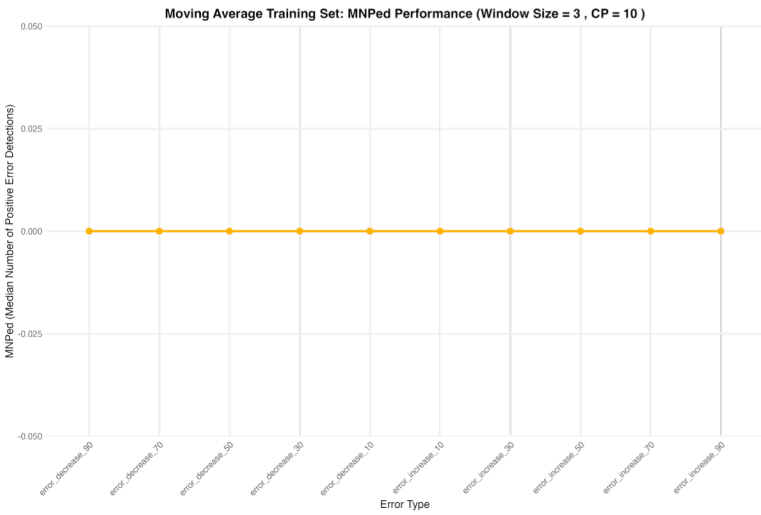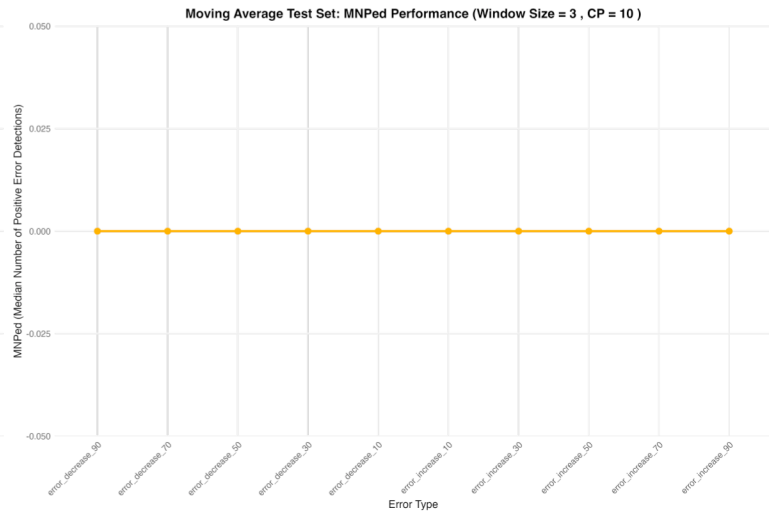

# TT - MNPED Plots

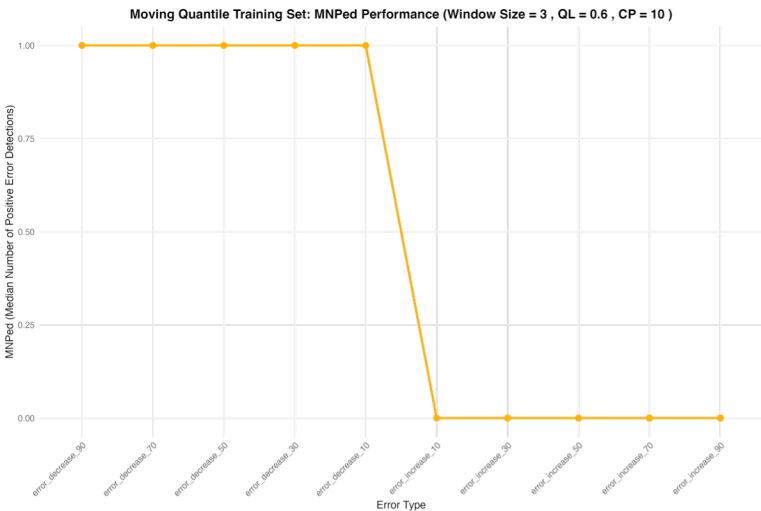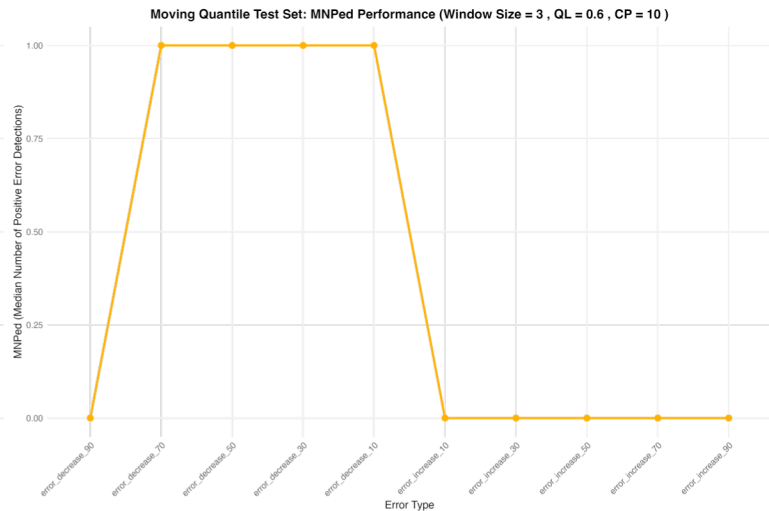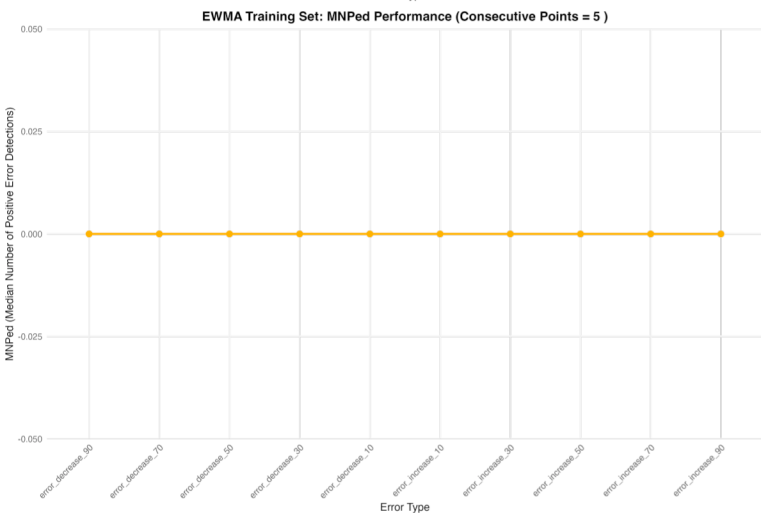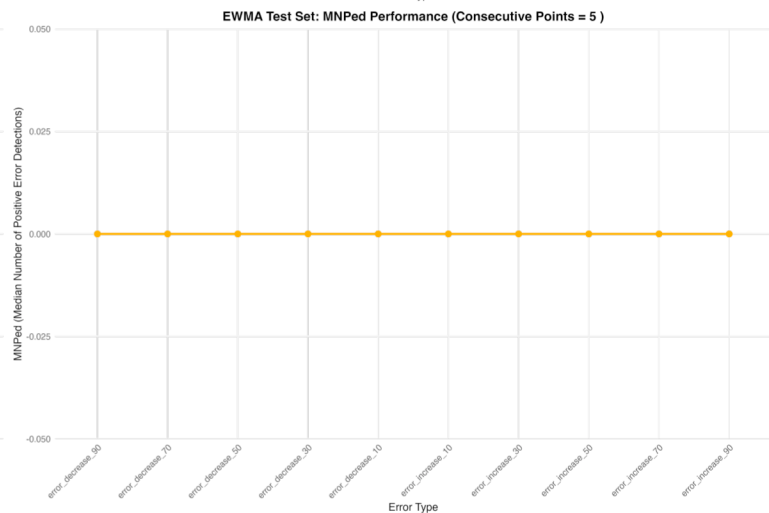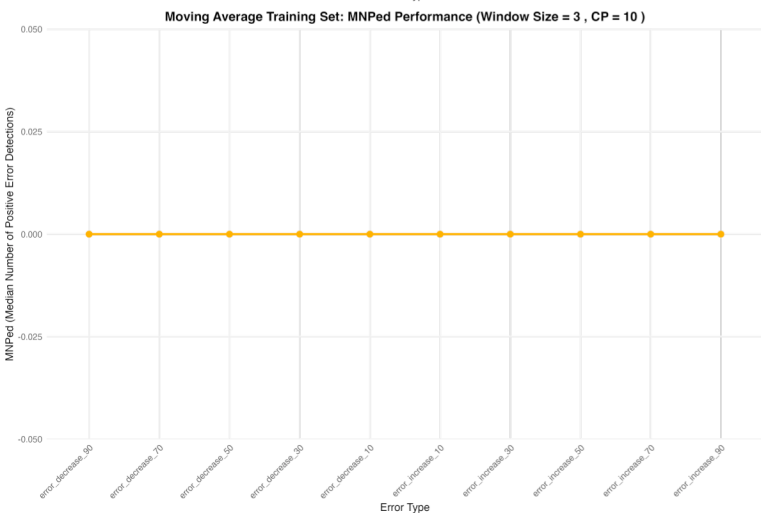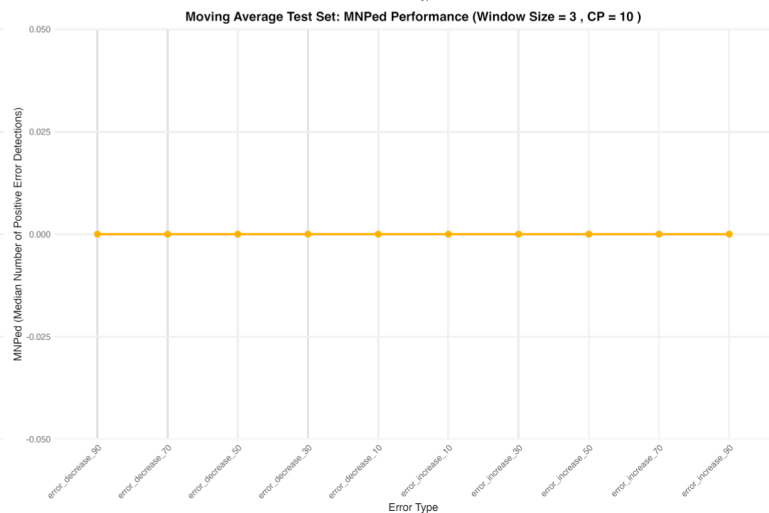

Supplement: Supplementary file 1 [file diagnostics-16-00288-s001.zip › Suppmental Figures S47-S51.pdf]
